# MPLID (Membrane Protein–Lipid Interaction Database): A Large-Scale Experimental Resource of Residue-Level Protein–Lipid Contacts

**DOI:** 10.1093/gigascience/giag080

**Published:** 2026-07-27

**Authors:** Folorunsho Bright Omage, Goran Neshich

**Affiliations:** Department of Computer Science, University of Oxford, Wolfson Building, Parks Road, Oxford OX1 3QD, UK; The Computational Biology Research Group, Embrapa Digital Agriculture, Av. André Tosello 209, Campinas, SP 13083-886, Brazil; Biological Chemistry Laboratory, Department of Organic Chemistry, Institute of Chemistry, University of Campinas (UNICAMP), R. Monteiro Lobato 270, Campinas, SP 13083-970, Brazil; The Computational Biology Research Group, Embrapa Digital Agriculture, Av. André Tosello 209, Campinas, SP 13083-886, Brazil

**Keywords:** membrane proteins, lipid–protein interactions, protein structure, machine learning dataset, structural biology, experimentally resolved contacts, residue-level classification, FAIR data

## Abstract

Membrane proteins constitute approximately 20–30% of all proteomes and represent over 60% of current drug targets. Although protein–lipid interactions play important structural and regulatory roles in membrane-associated proteins, most existing structural resources focus on identifying whether a residue lies within a membrane region, typically inferred from computational hydrophobicity-based positioning algorithms. This approach does not directly address a distinct biological question: which residues at the protein surface make direct physical contact with lipid molecules? Answering this question from experimental data is critical for understanding lipid-mediated allostery, designing lipid-mimetic therapeutics, and training accurate machine learning models for lipid-binding-site prediction. We present MPLID (Membrane Protein–Lipid Interaction Database), a curated residue-level dataset comprising 4,704 membrane proteins representing 813 sequence clusters at 30% identity, 8,055,325 residues, and 80,439 structurally observed lipid-contact annotations (1.00% observed positive rate). Labels are derived exclusively from experimentally resolved lipid molecules in experimentally determined Protein Data Bank structures using a 4.0 Å all-atom heavy-atom distance cutoff. Because many native lipid interactions are lost or remain unresolved during purification and structure determination, this observed rate represents a lower bound, and the non-contact class inevitably contains false negatives. The dataset uses a curated list of 117 candidate lipid identifiers across ten functional categories, including 90 PDB-derived ligand codes audited against the RCSB Chemical Component Dictionary and 27 CHARMM-style lipid identifiers encountered in cryo-EM depositions. These identifiers span phospholipids, cardiolipin, sphingolipids, sterols, fatty acids, glycerolipids, detergent mimetics, lipid A components, and CHARMM simulation nomenclature. To minimize data leakage, proteins are clustered at 30% sequence identity using MMseqs2, yielding 813 clusters partitioned into training (2,578), validation (1,051), and test (1,075) splits. Amino acid composition analysis reveals biologically consistent enrichment at lipid-contact sites: tryptophan (1.88×), arginine (1.44×), glycine (1.36×), lysine (1.33×), and phenylalanine (1.23×) are enriched, whereas proline (0.51×), isoleucine (0.57×), and aspartate (0.59×) are depleted. MPLID addresses a distinct biological question from existing resources such as OPM, MemBlob, and BioDolphin/PLIP by identifying residues that directly contact experimentally resolved lipid molecules rather than residues positioned within computationally defined membrane boundaries. With 4,704 proteins and more than 8 million annotated residues, MPLID provides the scale needed for training deep-learning models for lipid-contact prediction, with applications in structure-guided drug design and membrane-protein engineering. The dataset adheres to FAIR principles and is freely available under a CC0 public-domain dedication. Structurally resolved contacts represent only a subset of biological protein–lipid interactions, and MPLID is intended as an experimentally grounded resource rather than a complete catalogue of lipid-binding sites.

## Background

Membrane proteins are central to virtually all cellular processes, mediating transport of ions and solutes, signal transduction, energy conversion, and cell adhesion [1]. Genomic analyses consistently estimate that 20–30% of open reading frames in any given proteome encode membrane proteins [1], and pharmacological surveys have established that these proteins account for more than 60% of all current drug targets [2, 3]. Despite their biomedical importance, membrane proteins remain among the most challenging targets for structural and computational biology, in large part because their native functional context, the lipid bilayer, is difficult to recapitulate experimentally and model computationally.

A growing body of evidence demonstrates that lipid–protein interactions are not passive consequences of membrane embedding but rather active modulators of protein structure, dynamics, and function [4, 5]. Specific lipid binding events regulate ion channel gating [6], G protein-coupled receptor (GPCR) signaling [4], and transporter conformational cycling [7]. Cholesterol, for example, occupies defined binding sites on GPCRs that are structurally resolved at atomic resolution [8], and cardiolipin binding is essential for the function of mitochondrial respiratory chain complexes [9]. The diversity of lipid species in biological membranes, encompassing phospholipids, sphingolipids, sterols, and glycolipids [10, 11], creates a complex interaction landscape that shapes the behavior of membrane-embedded proteins.

### Membrane zone versus lipid contact: two distinct questions

A fundamental distinction exists between two related but different biological questions in membrane protein structural biology. The first question, “Is this residue located within the membrane zone?,” has been addressed by computational databases such as the Orientations of Proteins in Membranes (OPM) database [12, 13]. OPM positions membrane proteins in a hydrophobic slab model using energy minimization against experimentally calibrated transfer free energy parameters, producing membrane boundaries for over 15,000 protein entries. This approach is invaluable for understanding membrane protein topology, but the resulting labels reflect a coarse spatial localization: typically 20–40% of residues in a transmembrane protein fall within the OPM-defined membrane zone.

The second question, “Does this residue make direct physical contact with a lipid molecule?,” requires experimental structural evidence. Experimentally determined PDB structures frequently resolve lipid molecules at specific protein surface sites, revealing native lipid interaction partners at atomic resolution. These experimental contacts represent a far more stringent and specific annotation: in our dataset, only 1.00% of all residues in membrane proteins (including both surface-exposed and buried residues) are observed to make lipid contacts within 4.0 Å (using an all-atom distance criterion). However, this rate represents a lower bound on the true frequency of lipid contacts in biological membranes, because only a subset of native lipid interactions survive protein purification and are resolved in experimentally determined PDB structures. Nonetheless, even as a lower bound, this rate is approximately 20- to 40-fold lower than the membrane zone positive rate, reflecting the much lower observed frequency of structurally resolved lipid contacts.

This distinction matters for both biological interpretation and machine learning model development (Table 1). Membrane zone labels identify the general region of a protein that is embedded in the bilayer, while lipid contact labels pinpoint specific binding sites that may mediate functionally important lipid–protein interactions. For drug discovery, the latter is far more actionable: lipid contact sites represent potential targets for allosteric modulation by lipid-mimetic compounds [14], while membrane zone annotations provide no such site-level resolution. We emphasize that no OPM-derived residue labels were used at any stage of the MPLID construction pipeline; all residue annotations derive exclusively from experimentally resolved lipid molecules in PDB structures. OPM is discussed here solely to distinguish the complementary biological questions of membrane positioning versus direct lipid contact.

**Table 1:** Comparison of membrane zone and lipid contact prediction tasks.

Aspect	Membrane zone	Lipid contact
Label source	Computational (OPM)	Experimental (PDB)
Biological question	In membrane slab?	Contacts lipid?
Observed positive rate	20–40%	$\sim$ 1.00%^a^
Spatial resolution	Zone-level ($\sim$30 Å)	Atomic (4.0 Å)
Evidence type	In silico energy model	X-ray/cryo-EM/NMR
Key application	Topology prediction	Binding site identification

^a^Lower bound; the true lipid contact rate in vivo is likely substantially higher due to lipid loss during purification and incomplete resolution.

These represent fundamentally different biological questions with distinct label sources, positive rates, and downstream applications.

### Existing resources and their limitations

Several resources provide information about membrane protein–lipid interactions, each with specific scope and limitations. The OPM database [12] remains the definitive resource for membrane protein positioning, providing computationally derived membrane boundaries for transmembrane and peripheral proteins. However, OPM labels represent theoretical membrane zones rather than experimentally resolved lipid interaction sites.

The MemProtMD database [15, 16] complements OPM by providing coarse-grained molecular dynamics (CG-MD) simulations of membrane proteins embedded in explicit phospholipid bilayers. While MemProtMD offers valuable insights into lipid organization around proteins, its contacts derive from simulations rather than experimental structures, and the database focuses on integral membrane proteins in a single lipid type (dipalmitoylphosphatidylcholine).

The MemBlob database and server [17] take a complementary approach by analyzing cryo-EM density maps to computationally identify membrane-facing regions of transmembrane proteins. MemBlob classifies residues as solvent-facing, lipid-facing, at the lipid interface, or buried, providing a membrane environment annotation that bridges between the coarse OPM slab model and direct experimental contact data. While MemBlob provides valuable residue-level environment classification, its labels are derived from density map analysis rather than from resolved lipid molecules, and the database is limited to proteins with available cryo-EM reconstructions.

BioDolphin [18] identifies and catalogs all protein–lipid interactions across the PDB using the PLIP (Protein-Ligand Interaction Profiler) framework [19], providing detailed interaction-type annotations (hydrogen bonds, hydrophobic contacts, salt bridges) at the residue and atom level. BioDolphin offers API access and covers a broad range of interaction types, making it useful for detailed mechanistic analysis of individual protein-lipid complexes. MPLID differs from BioDolphin in its focus on providing pre-computed, ML-ready binary contact labels with cluster-aware train/validation/test splits, continuous distance values, and FAIR-compliant dataset deposition, rather than on-demand interaction profiling.

The STING Relational DataBase (RDB) [20, 21] provides a comprehensive structural analysis platform that computes and stores inter-atomic contacts, surface accessibility, and residue-level descriptors for all PDB structures. While STING RDB does not specifically label lipid contacts, its contact calculation infrastructure and residue-level annotation framework informed the design philosophy of MPLID, particularly regarding the use of distance-based contact definitions and the importance of providing both binary labels and continuous distance values for downstream analysis.

On the prediction methodology side, the DREAMM method [22] demonstrated that ensemble machine learning can achieve a Matthews correlation coefficient (MCC) of 0.84 for predicting protein-membrane interfaces, using a curated set of 54 peripheral membrane proteins (later expanded to 65) with experimentally characterized membrane-penetrating residues. However, this training data was distributed as part of the prediction tool rather than as a standalone, documented dataset resource. More recently, PMIpred introduced a physics-informed transformer approach trained on molecular dynamics data for peptide–membrane interactions [23], and protein language model approaches have been explored using ProtTrans embeddings [24, 25]. While these studies have advanced prediction methodology, none provides a large-scale, publicly deposited, and documented dataset covering the full diversity of membrane protein classes.

### The need for large-scale experimental lipid contact data

The convergence of three trends motivates the creation of MPLID. First, the rapid growth of the Protein Data Bank [26, 27], which now contains more than 230,000 structures, means that thousands of membrane protein structures with resolved lipid molecules are available for systematic analysis. Second, advances in protein language models such as ESM-2 [28] and ProtTrans [25] have demonstrated that per-residue embeddings trained on large-scale protein sequence data can capture structural and functional properties, but training supervised models on top of these representations requires large labeled datasets. Third, structure-guided drug discovery increasingly recognizes lipid binding sites as druggable targets for allosteric modulation [6, 14], creating demand for systematic catalogs of structurally observed lipid interaction sites.

MPLID addresses these needs by providing structurally observed lipid contact annotations for 4,704 membrane proteins containing 8,055,325 residues. All labels derive from experimentally resolved lipid molecules in PDB structures, providing experimentally determined structural evidence at atomic resolution. While existing resources offer related information, including membrane environment classification from cryo-EM density (MemBlob [17]) and detailed interaction-type profiling (BioDolphin/PLIP [18, 19]), none provides a pre-computed, ML-ready residue-level dataset with cluster-aware evaluation splits and FAIR-compliant deposition. MPLID fills this gap by covering integral, peripheral, and single-pass membrane proteins across 813 sequence-diverse clusters.

## Data description

### Dataset overview

**Figure 1:**
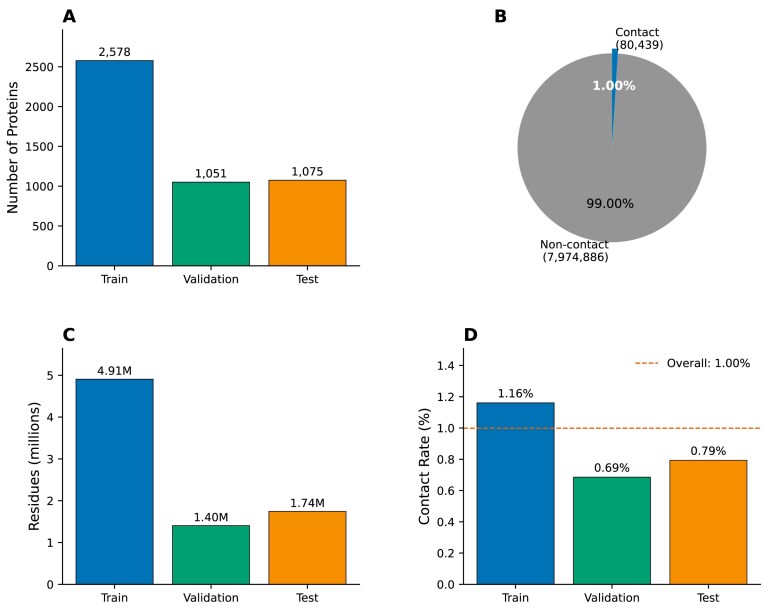
MPLID dataset overview. (A) Distribution of proteins across training (54.8%), validation (22.3%), and test (22.9%) splits. (B) Class distribution showing the imbalance between contact (1.00%) and non-contact (99.00%) residues. (C) Total residues per split. (D) Contact rates across splits, showing variation due to cluster-level stratification: training (1.16%), validation (0.69%), and test (0.79%).

MPLID provides residue-level binary annotations indicating whether each residue in a membrane protein makes direct physical contact with an experimentally resolved lipid molecule. The complete dataset encompasses 4,704 membrane proteins with a total of 8,055,325 residues, of which 80,439 (1.00%) are labeled as lipid contacts (Fig. 1). This observed positive rate should be interpreted as a lower bound on the true frequency of protein-lipid contacts in biological membranes: many native lipid interactions are lost during protein purification and crystallization, and most PDB structures resolve only a small number of tightly bound or structurally ordered lipids. Consequently, the non-contact class (99.00%) inevitably contains false negatives (that is, residues that may contact lipids in vivo but lack a resolved lipid partner in the experimentally determined structure). The remaining residues classified as non-contacts include solvent-exposed domains, protein-protein interfaces, interior regions of multi-subunit complexes, and an unknown fraction of unresolved lipid-binding sites.

Proteins in the dataset range from 22 to 15,280 residues (median 1,038; mean 1,712), reflecting the diversity of membrane protein sizes from small single-pass transmembrane peptides to large multi-subunit complexes. The number of lipid contacts per protein ranges from 1 to 4,820 (median 4; mean 17), with the highly skewed distribution reflecting that most proteins have few resolved lipid contacts while a small number of well-characterized complexes have extensive lipid interactions. The maximum value (4,820 contacts for PDB 6W4F, an NMR-resolved structure of the KRAS4B homodimer on a lipid bilayer nanodisc) arises because the current pipeline counts residue-lipid contacts across all NMR conformational models deposited in the PDB coordinate file; 6W4F contains 20 NMR models, each with approximately 240 contact residues. A total of 46 NMR multi-model structures in the dataset are affected by this counting convention, contributing approximately 29,000 excess contact annotations (see “Limitations” section). Every protein in the final dataset has at least one lipid contact, as proteins with zero contacts were removed during quality filtering (see “Methods” section). This removal excludes membrane proteins whose lipids were stripped during purification, meaning that MPLID does not represent the complete membrane proteome and cannot serve as a source of proteome-wide negative controls.

### Data schema and format

Each record in MPLID contains ten fields that provide complete information for each annotated residue. The pdb_id field contains the four-character PDB accession code, and chain_id specifies the polypeptide chain within the structure. The residue_number gives the residue’s position in the chain, while residue_name provides the three-letter amino acid code. The binary label is_contact takes a value of 1 for residues where any heavy atom is within 4.0 Å of any lipid heavy atom and 0 otherwise. The label_source field is set to “EXPERIMENTAL” for all entries, confirming that labels derive from experimentally resolved lipid molecules in PDB structures rather than computational predictions. The confidence field is currently set to “high” for all entries as a provenance label indicating an experimentally determined structural source; it should not be interpreted as a per-contact quality score. Future releases may incorporate structure resolution, occupancy, and B-factor information. The min_distance field records the minimum distance in Angstroms between any heavy atom of the residue and the nearest lipid heavy atom. Finally, cluster_id assigns each protein to a sequence cluster, and split indicates assignment to training, validation, or test partitions.

Data are distributed as gzip-compressed comma-separated values (CSV) files partitioned by split (train, validation, test), along with a protein-level metadata file containing per-protein summary statistics including residue counts, contact counts, contact rates, cluster assignments, and split assignments.

### Train/validation/test splits

The dataset is partitioned into training (2,578 proteins; 4,907,696 residues), validation (1,051 proteins; 1,403,838 residues), and test (1,075 proteins; 1,743,791 residues) splits (Fig. 1). Critically, splitting is performed at the protein level with cluster-aware stratification: proteins within the same sequence cluster are always assigned to the same split, preventing data leakage from sequence-similar proteins appearing in both training and evaluation partitions. Contact rates vary across splits (training 1.16%, validation 0.69%, test 0.79%) because stratification is performed at the cluster level rather than the residue level, and clusters with higher contact rates may be unevenly distributed. This variation is expected and should be accounted for when comparing model performance across splits. This design choice is essential for meaningful generalization assessment, as protein-level leakage can dramatically inflate performance estimates in structural bioinformatics tasks.

The cluster-aware splitting strategy uses MMseqs2 [29] with a 30% sequence identity threshold and 80% bidirectional alignment coverage, producing 813 clusters. Entire clusters are assigned to splits to ensure that no protein in the validation or test sets shares more than 30% sequence identity with any training protein. This threshold is substantially more conservative than the 40% identity commonly used in protein function prediction benchmarks [30], further reducing the risk of overestimating generalization performance.

### Sequence clustering analysis

**Figure 2:**
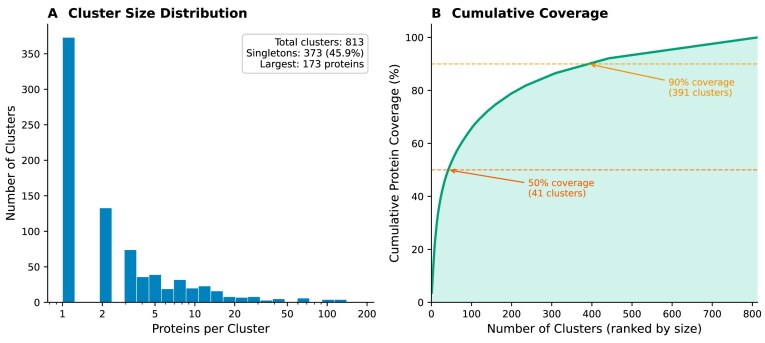
Sequence cluster analysis. (A) Distribution of cluster sizes at 30% sequence identity on a log scale, showing a highly skewed size distribution with many singletons and a small number of large clusters containing many homologous structures. (B) Cumulative protein coverage ranked by cluster size, illustrating the concentration of proteins in a few large families.

The 813 sequence clusters exhibit a highly skewed size distribution (Fig. 2), with 373 singletons (45.9%) and a largest cluster of 173 proteins, reflecting the sequence diversity of membrane proteins across different families and superfamilies. This clustering pattern indicates substantial structural and functional diversity in the dataset, as singleton clusters represent unique membrane protein families without close homologs.

## Analyses

### Amino acid composition at lipid contact sites

**Figure 3:**
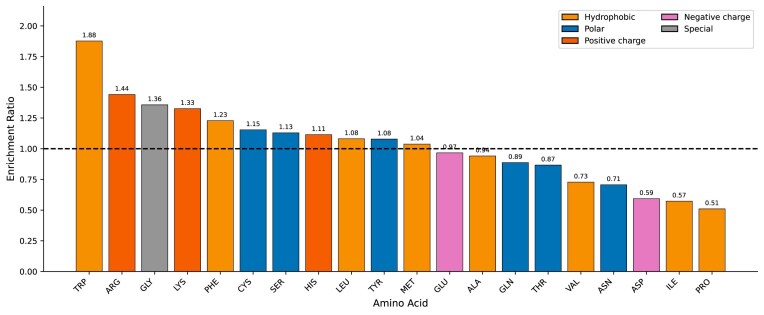
Amino acid enrichment at lipid contact sites. Enrichment ratios are calculated as the frequency of each amino acid among contact residues divided by its overall frequency in the dataset. Bars are grouped by physicochemical class, as indicated in the legend: hydrophobic, polar, positively charged, negatively charged, and special. The dashed line marks the expected frequency (1.0). Values above 1.0 indicate enrichment; values below 1.0 indicate depletion.

Analysis of amino acid enrichment at lipid contact sites reveals biologically meaningful and interpretable patterns (Fig. 3). Enrichment ratios were calculated as the fraction of each amino acid among contact residues divided by its fraction in the complete dataset. Tryptophan (1.88$\times$) shows the strongest enrichment, consistent with the well-established role of aromatic residues in anchoring transmembrane proteins at the membrane-water interface through favorable interactions with lipid headgroups via cation-$\pi$ and hydrogen bonding interactions [4]. Notably, positively charged residues are strongly enriched: arginine (1.44$\times$) and lysine (1.33$\times$) rank among the top five most enriched amino acids, consistent with the “positive-inside rule” [31] and with the electrostatic attraction of cationic side chains to anionic phospholipid headgroups. Glycine (1.36$\times$) is enriched, reflecting its prevalence in GxxxG motifs that mediate transmembrane helix-helix packing and create lipid-accessible surfaces at helix interfaces [32]. Phenylalanine (1.23$\times$) and tyrosine (1.08$\times$) are moderately enriched, contributing to the aromatic belt. Among other residues, cysteine (1.15$\times$), serine (1.13$\times$), and histidine (1.11$\times$) show mild enrichment, with histidine functioning as an interfacial anchoring residue through its amphipathic imidazole ring, and cysteine enrichment likely reflecting palmitoylation sites and disulfide-constrained loop regions at lipid interfaces. Leucine (1.08$\times$) is slightly enriched, reflecting its involvement in direct side-chain contacts with lipid acyl chains.

Among depleted residues, proline (0.51$\times$) and isoleucine (0.57$\times$) show the strongest depletion. Proline disrupts regular secondary structure and is underrepresented at smooth lipid-protein interfaces. The depletion of isoleucine, a $\beta$-branched hydrophobic residue, may reflect its preference for buried core positions rather than surface lipid contacts. Aspartate (0.59$\times$) is depleted, consistent with the electrostatic incompatibility of negatively charged side chains with the lipid bilayer interior. Asparagine (0.71$\times$) and valine (0.73$\times$) are moderately depleted. Threonine (0.87$\times$) and glutamine (0.89$\times$) show mild depletion, while alanine (0.94$\times$) and glutamate (0.97$\times$) are near neutral. Overall, the enrichment pattern strongly supports the biological validity of the all-atom contact definition: residues capable of electrostatic interactions with phospholipid headgroups (arginine, lysine) and aromatic anchoring at the interface (tryptophan, phenylalanine) are enriched, while conformationally constrained (proline) and $\beta$-branched (isoleucine, valine) residues are depleted. These patterns are consistent with previous analyses of cardiolipin binding site composition [33] and likely reflect, in part, the overrepresentation of anionic lipids (particularly cardiolipin and phosphatidylglycerol) among structurally resolved lipid species. Because anionic lipids are more readily co-purified and crystallized with membrane proteins than zwitterionic species such as phosphatidylcholine and phosphatidylethanolamine, the observed enrichment of positively charged residues may be amplified relative to the in vivo membrane environment, where the majority of lipids are zwitterionic. Given the large sample sizes (80,439 contact residues among 8,055,325 total residues), these enrichment and depletion patterns are robust and unlikely to arise from sampling variation, but their magnitude may be influenced by the lipid composition bias inherent to crystallographic datasets. A sensitivity analysis excluding all 46 NMR multi-model structures confirms that the core enrichment pattern is preserved (Pearson $r = 0.86$; Spearman $\rho = 0.80$, $p = 2.3 \times 10^{-5}$; Supplementary Analysis S2).

### Distance distribution analysis

**Figure 4:**
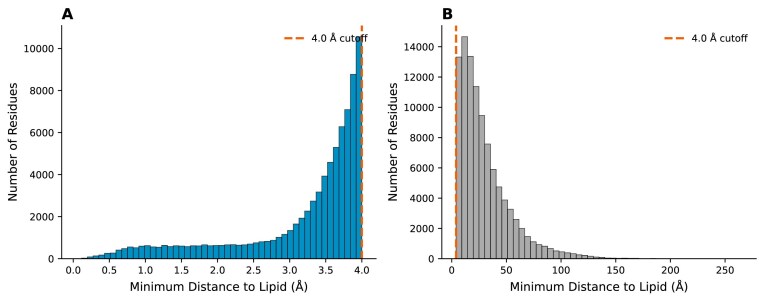
Distribution of minimum residue-to-lipid distances. (A) Contact residues ($\le$4.0 Å) show a peak near 3.5–4.0 Å, consistent with van der Waals contact distances. (B) Non-contact residues display a broad distribution with distances extending well beyond 30 Å, showing the separation imposed by the 4.0 Å operational cutoff.

The distribution of minimum distances between protein residues and lipid molecules characterizes the separation produced by the 4.0 Å contact cutoff (Fig. 4). Among contact residues, the distance distribution shows a clear peak at approximately 3.5–4.0 Å, corresponding to typical van der Waals contact distances between heavy atoms. The sharp boundary at 4.0 Å is imposed by the operational contact definition and should not be interpreted as independent validation of the threshold. For non-contact residues, the distribution is broadly shifted toward larger distances, with a peak near 8–12 Å and a long tail extending to distances greater than 30 Å for residues in soluble domains far from any lipid molecule.

### Protein size and contact density distributions

**Figure 5:**
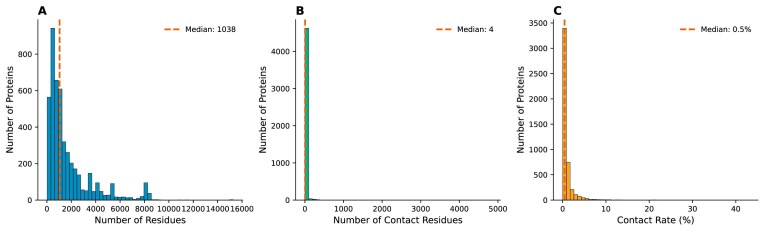
Protein-level statistics. Distribution of protein sizes and contact densities across the MPLID dataset, illustrating the diversity of membrane protein families represented.

The distribution of protein sizes in MPLID spans three orders of magnitude, reflecting the diversity of membrane protein families represented (Fig. 5). Contact density, defined as the fraction of residues making lipid contacts, varies substantially across proteins, with a per-protein median contact rate of 0.45% and a mean of 0.99%. The observed contact rate of 1.00% reflects both the sparse resolution of lipid molecules in PDB structures and the inherent specificity of direct van der Waals contacts. This rate should be interpreted as a lower bound; the severe class imbalance (approximately 100:1 negative-to-positive ratio) includes a substantial fraction of false negatives in the non-contact class, where residues that contact lipids in vivo lack a resolved lipid partner in the experimental structure. This imbalance requires specialized machine learning approaches such as class weighting or focal loss.

### Comparison with existing resources

**Figure 6:**
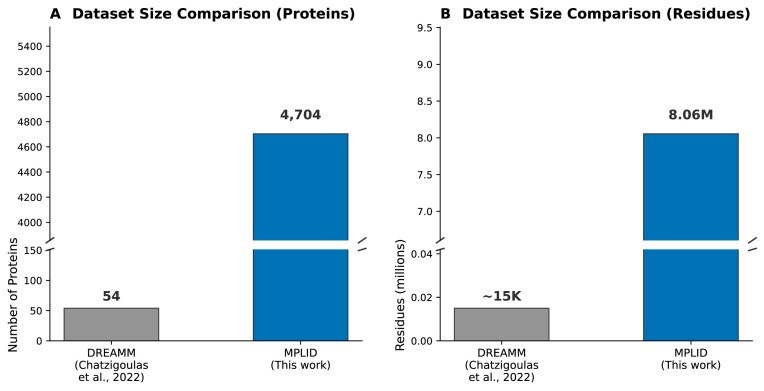
Scale comparison between MPLID and the training data used by the DREAMM prediction method [22], the largest prior collection of experimentally annotated membrane protein–lipid interactions. (A) Number of proteins (4,704 versus 54). (B) Number of annotated residues (8,055,325 versus $\sim$15,000). Note that these resources differ in scope: DREAMM focuses on peripheral membrane proteins, while MPLID covers all membrane protein classes.

Table 2 places MPLID in the context of existing membrane protein–lipid interaction resources (Fig. 6). Each resource addresses a different aspect of the problem. OPM [12] provides computationally derived membrane zone boundaries for topology analysis. MemProtMD [15] offers coarse-grained simulation data for lipid organization. The DREAMM prediction method [22] used a curated set of 54 peripheral membrane proteins with experimentally characterized membrane-penetrating residues as training data, though this data was not released as a standalone documented dataset. MPLID fills this gap by providing the first large-scale, publicly deposited, and fully documented dataset of experimental lipid contact labels, covering 4,704 proteins across all membrane protein classes with FAIR-compliant metadata, a data dictionary, and pre-defined evaluation splits. The diversity of membrane protein families represented in MPLID, spanning 813 sequence clusters at 30% identity, ensures broad coverage of the membrane protein structural landscape. These resources are complementary rather than competing, each addressing a different level of the membrane protein–lipid interaction hierarchy.

In practice, models trained on one type of label (e.g., OPM membrane zone) cannot be directly evaluated on another (e.g., MPLID lipid contact), and researchers should select the appropriate resource based on their specific biological question.

**Table 2:** Comparison of MPLID with existing membrane protein–lipid interaction resources.

Resource	Proteins	Residues	Label type	Label source
MPLID (this work)	4,704	8,055,325	Lipid contact	Experimental (PDB)
BioDolphin [18]^b^	PDB-wide	–	Interaction type	Experimental (PLIP)
MemBlob [17]^c^	$\sim$ 500	–	Membrane environment	Cryo-EM density
DREAMM [22]^a^	54	$\sim$ 15,000	Membrane interface	Experimental (curated)
OPM [12]	15,096	–	Membrane zone	Computational
MemProtMD [15]	$\sim$ 3,500	–	Lipid contact	Simulated (CG-MD)

^a^Training data distributed within the DREAMM prediction tool repository; not released as a standalone documented dataset. ^b^On-demand interaction profiling via PLIP; not pre-computed ML-ready dataset with splits. ^c^Residue-level membrane environment classification from cryo-EM density maps.

MPLID is the first large-scale, publicly deposited dataset providing structurally observed residue-level lipid contact labels with FAIR-compliant documentation.

### Lipid type diversity

MPLID uses a curated list of 117 candidate lipid identifiers organized into ten functional categories, comprising 90 PDB-derived ligand codes and 27 CHARMM-style lipid identifiers commonly found in cryo-EM depositions. All 90 PDB-derived codes were audited against the RCSB Chemical Component Dictionary API during revision; the complete lipid ontology with RCSB verification status, official chemical names, and category assignments is provided in Supplementary Table S1. Phospholipids include cardiolipin (CDL, found in 675 structures), phosphocholine variants (PCW, 196 structures), phosphoethanolamine (PEE, 273 structures), and phosphoglycerol (PGV, 144 structures). Sterols are represented by cholesterol (CLR, 1,119 structures) and derivatives including cholic acid (CHD, 150 structures; a bile acid sterol derivative) and cholesterol hemisuccinate (Y01). Fatty acids include palmitate (PLM, 734 structures), oleic acid (OLA, 379 structures), myristate (MYR, 426 structures), and stearate (STE, 94 structures). Sphingolipids include sphingosine (SPH, 102 structures) and sphingosine-1-phosphate (S1P, 16 structures). Detergent mimetics used in crystallization, which often occupy native lipid binding sites, include dodecyl $\beta$-D-maltoside (LMT, 677 structures), octyl $\beta$-D-glucopyranoside (BOG, 510 structures), and lauryldimethylamine oxide (LDA, 263 structures). The protein-level metadata includes an explicit has_detergent_only flag indicating whether all lipid contacts for a given protein derive exclusively from detergent mimetics rather than physiological lipid species, enabling users to filter detergent-only entries if desired.

Notably, several biologically important lipid classes are absent or underrepresented in the current MPLID release. Phosphatidic acid (PA), phosphatidylserine (PS), and phosphoinositides (PI, PIP, PIP$_2$, PIP$_3$) are not represented among the recognized lipid codes, despite their well-established roles in membrane protein regulation. This absence reflects both the difficulty of resolving these lipids crystallographically (owing to their flexible headgroups and transient binding modes) and the focus of MPLID on lipid species that are currently represented in PDB HETATM records. Similarly, polyunsaturated fatty acids (e.g., docosahexaenoic acid, arachidonic acid) are rarely resolved in crystal structures. Future releases will expand lipid coverage as more structures with these lipid types become available in the PDB.

### Structural visualization of lipid contacts

**Figure 7:**
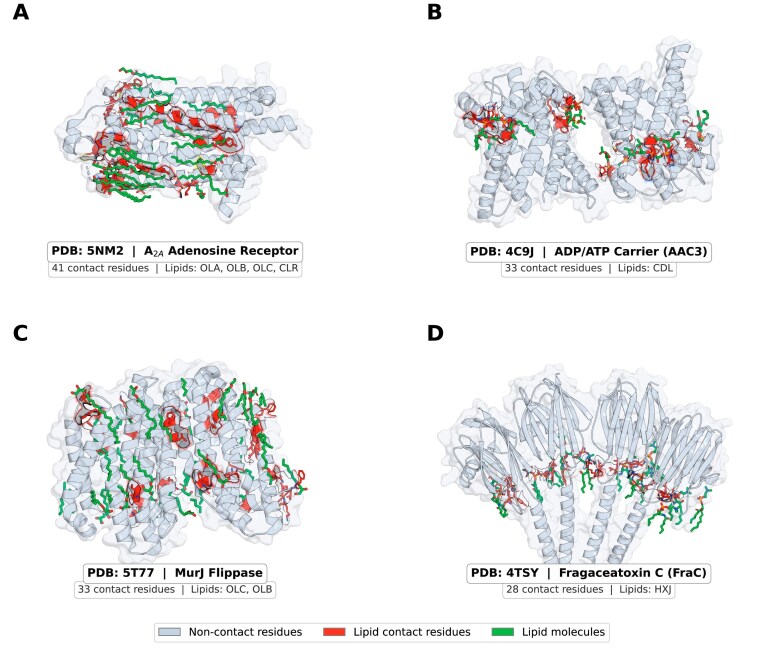
Structural visualization of MPLID lipid contact annotations across four representative membrane protein classes. (A) A_2A_ adenosine receptor (PDB: 5NM2), a GPCR with multiple lipid types including oleic acid and cholesterol. (B) ADP/ATP carrier AAC3 (PDB: 4C9J), a mitochondrial transporter with cardiolipin contact residues across two chains. (C) MurJ flippase (PDB: 5T77), a lipid II flippase with contacts distributed across its transmembrane helices. (D) Fragaceatoxin C (PDB: 4TSY), a tetrameric pore-forming toxin with sphingolipid contacts at the membrane insertion interface. These examples demonstrate that lipid contacts are site-specific rather than uniformly distributed across membrane-exposed surfaces.

To illustrate the nature of the lipid contact annotations in MPLID, representative membrane protein structures with their resolved lipid molecules are shown in Fig. 7. These examples demonstrate that lipid contacts occur at specific, well-defined sites on the protein surface rather than uniformly across the membrane-exposed region. Contact residues (highlighted in red/orange) are interspersed with non-contact residues even in transmembrane regions, confirming that lipid contact prediction is a more specific task than membrane zone classification.

### Computational label comparison studies

**Figure 8:**
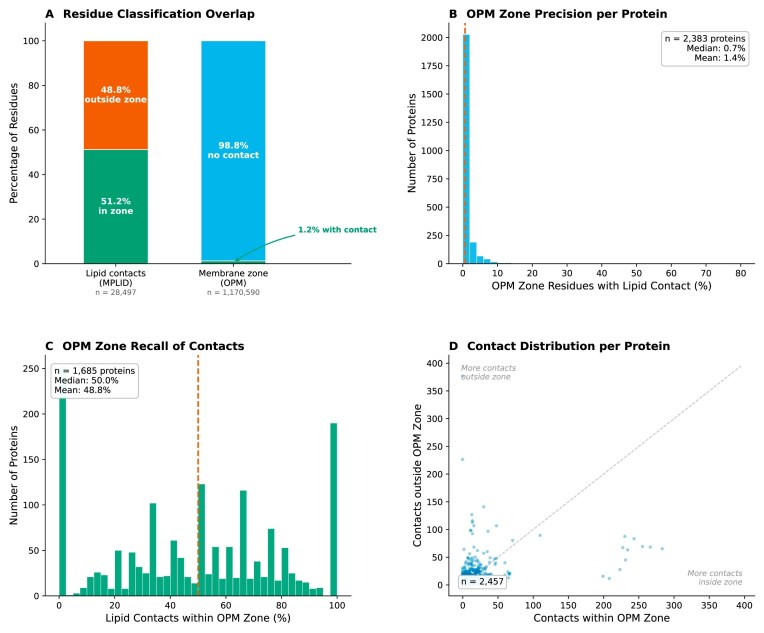
Quantitative comparison of MPLID lipid contacts versus OPM membrane zone annotations. (A) Residue classification overlap: 48.8% of lipid contacts ($n = 28{,}497$) fall outside the OPM membrane zone, while 98.8% of OPM zone residues ($n = 1{,}170{,}590$) lack direct lipid contact. (B) Per-protein OPM precision ($n = 2{,}383$ proteins): median 0.7% of membrane zone residues have lipid contacts. (C) Per-protein OPM recall ($n = 1{,}685$ proteins): the membrane zone captures a median of 50.0% of lipid contacts. (D) Per-protein scatter of contacts inside versus outside the OPM zone ($n = 2{,}457$ proteins). These results demonstrate that membrane zone classification and lipid contact annotation address fundamentally different biological questions.

The availability of MPLID alongside OPM membrane zone annotations enables systematic comparison of experimental lipid contacts with computational membrane boundaries. We compared MPLID contact labels with OPM-derived membrane zone assignments across proteins present in both resources (Fig. 8). The number of overlapping proteins varies by analysis: 2,383 proteins contributed to precision estimates, 1,685 to recall estimates, and 2,457 to contact distribution analysis, reflecting differences in the availability of OPM membrane zone annotations and non-zero lipid contacts across the shared set. Of all MPLID lipid contacts in the shared set ($n = 28{,}497$ residues), 51.2% fell within the OPM membrane zone while 48.8% were located outside the computationally defined boundaries, likely reflecting interfacial contacts, re-entrant loops, or amphipathic helices that extend beyond the hydrophobic core. Conversely, 98.8% of OPM membrane zone residues ($n = 1{,}170{,}590$) showed no direct lipid contact within 4.0 Å, and the per-protein median was only 0.7% of membrane zone residues having lipid contacts. The OPM membrane zone captured a median of 50.0% of lipid contacts per protein. These dataset-wide statistics highlight the fundamental difference between membrane zone classification and lipid contact prediction: being embedded in the membrane is neither sufficient nor necessary for direct lipid interaction. Researchers working with MemProtMD [15] simulations can similarly compare simulated lipid contacts with the experimental contacts provided by MPLID.

## Methods

### Data source integration

MPLID was constructed using a purely experimental approach with no dependence on computational membrane positioning databases. OPM data were not used at any stage of the MPLID construction pipeline; OPM is discussed in this paper solely to contextualize the complementary biological questions of membrane zone positioning versus direct lipid contact annotation. All residue annotations in MPLID derive exclusively from experimentally resolved lipid molecules in PDB structures. The RCSB Protein Data Bank [26, 27] was queried programmatically for all structures containing recognized lipid chemical components, identifying 8,221 candidate structures with lipid ligands. This query-first strategy ensures that every protein in the dataset has at least one experimentally resolved lipid molecule, providing broad structural coverage of experimentally resolved protein–lipid interactions across diverse membrane protein classes, while still reflecting the known experimental biases of structural biology datasets. The identified structures were downloaded as original PDB coordinate files (9,796 files total, as some entries required retrieval of biological assembly files or alternate depositions). After parsing, 5,723 unique proteins yielded valid structures with recognized HETATM lipid records; the remainder comprised duplicate chains, structures that failed parsing, or entries lacking standard amino acid residues. These were processed through the contact calculation and quality filtering pipeline described in the following subsections.

### Lipid molecule identification

Lipid molecules in PDB structures were identified using candidate ligand codes that were subsequently audited against the RCSB Chemical Component Dictionary. We compiled a list of 117 candidate lipid identifiers organized into ten functional categories: phospholipids (POV, PCW, PEE, PGV, and others), cardiolipin (CDL, C9V, 18W, LCL), sphingolipids (SPH, S1P, HXJ, and others), sterols (CLR, CHD, Y01, ERG), fatty acids (PLM, MYR, OLA, STE, DHA, and others), glycerolipids (TGL, DAG, MAG), detergent mimetics (LDA, LMT, BOG, OLC, DPC), lipid A components (LPA, KDO, 6LP), and CHARMM simulation nomenclature (POPC, POPE, POPG, and others commonly found in cryo-EM depositions). This list was developed iteratively by cross-referencing the PDB Chemical Component Dictionary with lipid classification databases and manually curating entries to exclude non-lipid small molecules that might share similar chemical codes. Detergent mimetics were included because they frequently occupy native lipid binding sites in membrane protein crystal structures and provide relevant information about lipid–protein interaction geometry [7].

### Structure processing and contact calculation

For each candidate protein, the original PDB coordinate file was downloaded from RCSB to preserve HETATM records containing lipid molecule coordinates. Structure parsing was performed using Biopython [34], extracting all standard amino acid residues (ATOM records) and all recognized lipid molecules (HETATM records). Non-standard residues, water molecules, metal ions, and non-lipid ligands were excluded.

Lipid contacts were defined using an all-atom distance criterion. For each protein residue *R* and each lipid molecule *L* in the structure, the minimum distance $d_{\min }(R, L)$ between any heavy (non-hydrogen) atom of *R* and any heavy atom of *L* was calculated. A residue was labeled as a lipid contact if:


(1) \begin{eqnarray*}
d_{\min }(R, L) = \min _{a \in R,\, b \in L} \Vert \mathbf {r}_a - \mathbf {r}_b \Vert \le 4.0~\mathrm{\mathring{\rm A}} ,
\end{eqnarray*}

where $\mathbf {r}_a$ denotes the Cartesian coordinates of heavy atom *a* in residue *R* and $\mathbf {r}_b$ denotes the coordinates of heavy atom *b* in lipid *L*. The use of all heavy atoms (backbone and side chain) as residue representatives captures direct van der Waals contacts that would be missed by a C$\alpha$-only criterion, particularly for residues whose side chains extend into lipid-binding pockets while their backbone remains distant from lipid molecules. The 4.0 Å cutoff was chosen to capture direct van der Waals contacts between heavy atoms, consistent with standard distance thresholds used in protein–ligand interaction analysis [19], protein contact databases [20], and previous membrane protein–lipid interaction studies [22]. Typical van der Waals radii for carbon, nitrogen, and oxygen atoms sum to contact distances of 3.2–3.8 Å, and the 4.0 Å threshold provides a modest margin to accommodate coordinate uncertainty and conformational variability while remaining well below the threshold for indirect (water-mediated or electrostatic) interactions. The distance distribution analysis (Fig. 4) characterizes this operational cutoff, showing a clear peak at 3.5–4.0 Å consistent with van der Waals separations. We note that this cutoff does not distinguish between contacts with lipid headgroups and acyl chains; both are captured by the all-atom criterion. Researchers interested in headgroup-specific or acyl-chain-specific contacts can use the continuous min_distance values together with the lipid atom identities available in the original PDB coordinate files. For each residue, the minimum all-atom distance to the nearest lipid heavy atom was recorded regardless of contact status, providing a continuous distance metric that enables researchers to evaluate alternative cutoff values.

### Quality filtering

Of the 5,723 proteins with parseable lipid-containing structures, 1,019 were removed because they contained only non-lipid molecules misannotated as lipids (such as HEPES buffer and bacteriochlorophyll) or had all lipid atoms beyond the 4.0 Å contact threshold, leaving 4,704 proteins with at least one genuine lipid contact. This filtering ensures that every protein in the final dataset has genuine, experimentally resolved lipid contacts.

### Sequence clustering

To prevent data leakage between training, validation, and test splits, all 4,704 proteins were clustered using MMseqs2 [29] with a 30% sequence identity threshold and 80% bidirectional alignment coverage. These stringent thresholds ensure that proteins assigned to different splits are sufficiently divergent in sequence to represent independent test cases. The clustering produced 813 clusters.

### Split strategy

Clusters were assigned to training, validation, and test splits using stratified random sampling at the cluster level, with stratification based on the average contact rate per cluster. This approach ensures that each split contains a representative distribution of high-contact and low-contact proteins. Entire clusters were assigned to a single split, guaranteeing that no two proteins sharing more than 30% sequence identity appear in different partitions. The resulting splits contain 2,578 training proteins (54.8%), 1,051 validation proteins (22.3%), and 1,075 test proteins (22.9%).

### Software and computational environment

The MPLID construction pipeline was implemented in Python 3.11.8 using Biopython 1.83 [34] for PDB structure parsing, pandas 2.2.2 [35] for data manipulation, NumPy 1.26.4 for numerical computation, SciPy 1.13 for distance calculations, and scikit-learn 1.7.2 [36] for data splitting. MMseqs2 version 15 [29] was used for sequence clustering. The RCSB PDB search API was used for programmatic identification of structures containing lipid chemical components.

## Discussion

MPLID represents the first large-scale, publicly deposited dataset of structurally observed residue-level lipid contacts for membrane proteins, complementing existing resources such as OPM (membrane zone positioning), MemBlob (membrane environment inference from cryo-EM density), and BioDolphin/PLIP (generalized structural interaction profiling). The dataset addresses a biological question that is fundamentally distinct from membrane zone classification: rather than identifying residues located within a computationally defined hydrophobic slab, MPLID annotates residues that make direct physical contact with experimentally resolved lipid molecules at atomic resolution. Importantly, MPLID captures only the subset of protein–lipid interactions that survive purification and are resolved in experimentally determined PDB structures; biological protein–lipid interactions are therefore broader than the structurally resolved set. The analyses presented here validate this annotation approach through multiple independent lines of evidence. The amino acid enrichment patterns at lipid contact sites are biologically consistent, with aromatic anchoring residues (tryptophan, phenylalanine) and positively charged residues (arginine, lysine) enriched at contact sites, while conformationally constrained (proline) and negatively charged (aspartate) residues are depleted, consistent with independent analyses of cardiolipin binding sites [33]. The distance distribution analysis confirms that the 4.0 Å cutoff effectively captures the van der Waals contact boundary. The quantitative comparison with OPM membrane zone annotations demonstrates that nearly half of experimentally observed lipid contacts fall outside computationally predicted membrane boundaries, reinforcing the complementary nature of these resources.

The scale of MPLID, comprising 4,704 proteins across 813 sequence clusters, provides substantially greater coverage than prior experimental lipid contact datasets. The DREAMM training set [22], the largest previously available collection, contained 54 peripheral membrane proteins. MPLID expands this by nearly two orders of magnitude while covering all membrane protein classes, including integral, peripheral, and single-pass proteins. The cluster-aware splitting strategy, using a conservative 30% sequence identity threshold, ensures that evaluation on the test set reflects genuine generalization rather than sequence memorization.

### Limitations

Several limitations should be noted when using MPLID.

#### False negatives in the non-contact class

Lipid contact labels depend on the crystallographic or cryo-EM resolution and completeness of resolved lipid molecules; many native lipid binding events are not captured because lipids are disordered or stripped during purification and crystallization. Consequently, the false-negative rate in the non-contact class is likely substantial, although its true magnitude cannot presently be estimated. The true frequency of lipid contacts in membrane proteins in vivo is almost certainly higher than the 1.00% observed in MPLID. Moreover, many structures contain only one or a few resolved lipid molecules, meaning that the observed contacts represent a sparse snapshot of the full lipid-binding landscape rather than a comprehensive map of all possible interaction sites. Users training machine learning models on MPLID should be aware that the non-contact class is noisy and that model performance may be limited by false negative labels rather than by model capacity.

#### Detergent mimetics

The inclusion of detergent mimetics as proxy lipids introduces potential noise, as detergent binding sites may not perfectly recapitulate native lipid interactions. Analysis of HETATM records across all 4,704 processed PDB files shows that 1,039 proteins (22.1%) contain exclusively detergent mimetics (e.g., dodecyl maltoside, octyl glucoside, lauryldimethylamine oxide) without any physiological lipid species, while an additional 685 proteins (14.6%) contain both detergent and physiological lipids. This means that for roughly one in five proteins, all contact labels derive from crystallization detergents rather than native lipid species. The protein-level metadata includes an explicit detergent-only flag to enable filtering. These compounds were retained because they frequently occupy membrane-associated binding environments and preserve experimentally observable lipid–protein interaction geometries, even when they do not represent native physiological lipids. The cholesterol derivative CHD (cholic acid, a bile acid sterol derivative) and Y01 (cholesterol hemisuccinate) are distinct compounds with different PDB Chemical Component Dictionary identities (C$_{24}$H$_{40}$O$_5$ and C$_{31}$H$_{50}$O$_4$, respectively), though both are classified as sterols in MPLID. Users should note that cholesterol hemisuccinate is an artificial detergent commonly added during membrane protein solubilization and is not a native membrane lipid.

#### Lipid code verification

During revision, all 90 PDB-derived candidate ligand codes were audited against the RCSB Chemical Component Dictionary API (Supplementary Table S1). This audit confirmed 45 codes as correctly classified, identified 34 codes with incorrect or ambiguous category assignments, and found 11 candidate codes absent from the dictionary. Of the 34 misclassified codes, 15 appear in PDB HETATM records within the dataset, but most have low occurrence. The codes with the largest impact are CHD (cholic acid, present in 150 structures; name corrected from “cholesteryl hemisuccinate”), CRT (spirilloxanthin, a bacterial carotenoid incorrectly classified as ceramide, present in 22 structures), and ARA (alpha-L-arabinopyranose, a sugar incorrectly classified as arachidonic acid, present in 18 structures). These misclassified contacts collectively represent fewer than 300 protein entries and do not materially alter the dataset statistics. The three codes specifically flagged by reviewers (LNS, LNN, GLF) are confirmed as misclassified but do not appear in any PDB structure represented in MPLID and therefore do not affect residue-level contact annotations. The complete verification results are provided in Supplementary Table S1 for transparency.

#### NMR multi-model structures

The current pipeline counts residue-lipid contacts across all conformational models in NMR-derived PDB coordinate files rather than extracting contacts from a single representative model. Empirical detection of duplicated residue positions identified 46 multi-model structures in the dataset, contributing approximately 144,000 excess residue entries (1.8% of total residues) and approximately 29,000 excess contact annotations. Although substantial in relative terms ($\sim$36% of all contact annotations), these excess contacts derive from a small subset of multi-model structures and can be readily excluded through metadata filtering. The most extreme case is PDB 6W4F (KRAS4B homodimer on a lipid bilayer nanodisc), which contains 20 NMR models and reports 4,820 contacts across all models, compared to approximately 240 unique contact residues per individual model. A sensitivity analysis excluding all 46 multi-model structures confirms that the principal enrichment patterns (aromatic belt, arginine, glycine, proline depletion) are preserved (Spearman $\rho = 0.80$, $p = 2.3 \times 10^{-5}$), although lysine enrichment is sensitive to NMR inclusion (Supplementary Analysis S2). The multi-model convention also introduces artifactual sub-2.0 Å contact distances from cross-model atom overlaps; these effectively vanish upon NMR exclusion (Supplementary Analysis S2). Users working with NMR structures should be aware of this multi-model counting convention. Future releases will adopt a single-model extraction strategy for NMR structures.

#### PDB and lipid composition biases

The dataset inherits biases from the PDB toward well-studied, readily crystallizable membrane protein families, leaving many therapeutically relevant but structurally uncharacterized families underrepresented. As detailed in the Lipid type diversity section, several biologically important lipid classes (PA, PS, phosphoinositides, polyunsaturated fatty acids) are absent, reflecting the difficulty of resolving these lipids crystallographically rather than a deliberate exclusion. Anionic lipids (particularly cardiolipin and phosphatidylglycerol) are overrepresented among structurally resolved lipid contacts relative to their abundance in biological membranes, which has implications for machine learning models trained on MPLID: such models may partly reflect structural detectability biases alongside biological lipid-binding preferences. Additionally, the removal of 1,019 proteins with zero contacts during quality filtering (see “Dataset overview” section) further limits proteome-wide negative control inference.

#### Distance cutoff and static snapshots

The 4.0 Å distance cutoff is a single threshold that may not be optimal for all lipid types; researchers are encouraged to explore alternative cutoffs using the continuous distance values provided in the dataset. As noted in Methods, the cutoff does not distinguish between headgroup and acyl-chain contacts. Because MPLID annotations are derived from static crystallographic and cryo-EM snapshots, they may not fully represent the dynamic nature of protein–lipid interactions in native membrane environments, where lipids undergo lateral diffusion and exchange on nanosecond-to-microsecond timescales. Crystal packing artifacts may also contribute false positive contacts, as lipid molecules resolved in crystal structures can occupy lattice contact interfaces that do not reflect native membrane binding.

#### Additional technical notes

No structure resolution cutoff was applied during dataset construction: all PDB structures with recognized lipid molecules were included regardless of crystallographic resolution. While this maximizes dataset size, low-resolution structures (worse than 3.5 Å) may have less reliable lipid positions, potentially introducing noise into contact labels. Researchers working with resolution-sensitive applications are encouraged to filter by PDB resolution using the provided pdb_id field. The current release does not include per-residue lipid chemical component codes, meaning that users cannot directly determine which lipid type contacts a given residue; however, the pdb_id field allows users to retrieve lipid identities from the original PDB structures. Lipid type stratification at the residue level is planned for a future release. Four selenocysteine (SEC) residues in the dataset are all annotated as lipid contacts (4/4, 100% contact rate); given the extremely small sample size, this observation is not statistically meaningful and these residues were excluded from enrichment ratio analysis.

Cross-validation of MPLID labels against BioDolphin [18] interaction annotations and the curated dataset used by the DREAMM prediction method [22] is planned as future work to assess concordance between independently derived annotation approaches.

## Conclusions

In summary, MPLID provides a large-scale, experimentally grounded resource for membrane protein structural biology. By distinguishing direct lipid contact from membrane zone localization, and by positioning itself as complementary to existing resources including OPM, MemBlob, and BioDolphin/PLIP, the dataset enables residue-level prediction tasks with direct relevance to drug discovery, protein engineering, and mechanistic understanding of lipid-mediated regulation. Users should interpret MPLID annotations as representing structurally resolved lipid contacts rather than a complete inventory of biological protein–lipid interactions, and should account for the substantial false negative rate inherent to crystallographic and cryo-EM datasets. The dataset adheres to FAIR principles and is freely available under a CC0 public domain dedication. As the PDB continues to grow and cryo-EM structures increasingly resolve lipid molecules at higher resolution, future releases of MPLID are expected to expand both the coverage and the annotation granularity of this resource, including NMR single-model extraction, per-residue lipid type annotations, and expanded lipid ontology coverage.

## Potential implications

MPLID is designed to serve as a large-scale training and evaluation resource for multiple research applications in structural bioinformatics, drug discovery, and membrane biology.

### Machine learning for lipid contact prediction

The principal application of MPLID is as a training and evaluation dataset for developing machine learning models that predict lipid contact residues from protein sequence or structure. The pre-defined, cluster-aware train/validation/test splits enable standardized model comparison, and the class imbalance (1.00% observed positive rate, approximately 100:1 negative-to-positive ratio) presents a realistic and challenging binary classification task that requires specialized approaches such as class weighting, oversampling (SMOTE) [37], or focal loss functions. Recent advances in geometric deep learning for protein interaction site prediction [38, 39] and end-to-end protein interactome modeling [40] demonstrate the growing potential of graph-based and attention-based architectures for residue-level prediction tasks. While these methods have been developed primarily for protein–protein interaction sites, their hierarchical geometric representations and multiscale attention mechanisms are architecturally suitable for the protein-lipid contact prediction task addressed by MPLID, particularly given the spatial nature of lipid binding sites. The scale of the dataset is sufficient for training deep neural networks, including architectures that combine structural descriptors with protein language model embeddings from ESM-2 [28] or ProtTrans [25]. We note that the MCC should be used as the primary evaluation metric for this task due to the substantial class imbalance, as accuracy and even F1 scores can be misleading when the negative class dominates. Users should also be aware that the substantial false negative rate in the non-contact class (see “Limitations” section) may impose an inherent ceiling on achievable performance metrics.

### Drug discovery applications

Lipid binding sites on membrane proteins represent an emerging class of druggable targets. General anesthetics, neurosteroids, and endocannabinoids modulate ion channels and GPCRs through specific lipid binding sites [6, 41], and cholesterol binding sites on GPCRs are increasingly recognized as allosteric modulatory sites [8, 42]. This interpretation is consistent with recent work highlighting exosites and allosteric site-forming residues as therapeutically actionable protein-surface regions [43–45]. MPLID can inform the identification of lipid-mimetic drug binding sites by providing a catalog of structurally observed lipid interaction residues across thousands of membrane proteins. Predicted lipid contact sites on therapeutically relevant proteins can guide the design of lipid-conjugated prodrugs, membrane-anchored therapeutics, or allosteric modulators that exploit lipid binding pockets.

### Structural biology and protein engineering

Understanding which residues contact lipids is essential for rational membrane protein engineering. Mutations at lipid contact sites can destabilize membrane proteins, alter their trafficking, or modify their functional properties. MPLID provides data to train models that predict which residues are critical for lipid-mediated stability, enabling more informed design of stabilizing mutations for membrane protein crystallization, cryo-EM, or functional studies. Additionally, the dataset supports comparative analysis of lipid contact patterns across protein families, enabling identification of conserved lipid binding motifs and family-specific interaction signatures. These interpretations are based on static structural snapshots. Incorporation of dynamic information may alter residue–lipid proximity patterns, as conformational fluctuations could transiently modify contact definitions.

### Integration with protein language models

The residue-level format of MPLID is directly compatible with per-residue embeddings from protein language models. Each residue’s 1,280-dimensional ESM-2 embedding [28] or 1,024-dimensional ProtTrans embedding [25] can be concatenated with structural descriptors and used as input to supervised classifiers. The 813 sequence clusters provide sufficient diversity to evaluate whether language model representations generalize across membrane protein families with limited sequence similarity. Alternatively, MPLID can be combined with interpretable structural descriptor frameworks, such as STING RDB-derived nanoenvironment descriptors [20, 21, 43, 44]. In this setting, residue-level lipid contacts may be analyzed in terms of explicit physicochemical and geometric properties, enabling mechanistic interpretation of the nanoenvironment characteristics associated with lipid interaction. This dual compatibility supports both high-capacity representation learning approaches and descriptor-based, interpretable modeling strategies.

## Availability of source code and requirements

The source code implementing the complete MPLID construction and analysis pipeline is openly available, with project details as follows:

- Project name: MPLID
- Project home page: https://github.com/omagebright/MPLID
- License: MIT license
- Operating system(s): Linux, macOS
- Programming language: Python 3.11
- Other requirements: Biopython 1.83, pandas 2.2.2, NumPy 1.26.4, SciPy 1.13, scikit-learn 1.7.2, MMseqs2 v15
- RRID:SCR_028506
- biotools: MPLID
- WorkflowHub: https://workflowhub.eu/workflows/2176

The repository includes the complete processing pipeline (structure downloading, lipid identification, contact calculation, quality filtering, and split generation), the analysis scripts that generate the figures and tables in this article together with their underlying numerical data, and documentation of the data schema and methodology.

## Additional files

**Supplementary Table S1**.

## Abbreviations

CDL: cardiolipin; CG-MD: coarse-grained molecular dynamics; CLR: cholesterol; CSV: comma-separated values; DREAMM: detection of residues on exposed areas of membrane-associated macromolecules; ESM-2: Evolutionary Scale Modeling 2; FAIR: Findable, Accessible, Interoperable, Reusable; GPCR: G protein-coupled receptor; MCC: Matthews correlation coefficient; MPLID: membrane protein–lipid interaction database; NMR: nuclear magnetic resonance; OPM: orientations of proteins in membranes; PDB: Protein Data Bank; PLIP: protein–ligand interaction profiler; PLM: palmitic acid; RCSB: Research Collaboratory for Structural Bioinformatics; SMOTE: Synthetic Minority Over-sampling Technique.

## Ethics approval and consent to participate

Not applicable. This study uses publicly available structural data from the Protein Data Bank and does not involve human subjects, animal experiments, or clinical data.

## Supplementary Material

giag080_Supplemental_File

giag080_Authors_Response_To_Reviewer_Comments_Original_Submission

giag080_GIGA-D-26-00059_original_submission

giag080_GIGA-D-26-00059_revision_1

giag080_Reviewer_1_Report_Original_Submission Reviewer 1 -- 3/19/2026

giag080_Reviewer_1_Report_Revision_1 Reviewer 1 -- 6/10/2026

giag080_Reviewer_2_Report_Original_Submission Reviewer 2 -- 4/29/2026

giag080_Reviewer_2_Report_Revision_1 Reviewer 2 -- 6/22/2026

giag080_Reviewer_3_Report_Original_Submission Reviewer 3 -- 4/30/2026

## Data Availability

The complete MPLID dataset is deposited at Zenodo under a CC0 public domain dedication [46]. The Zenodo archive contains the full residue-level annotations (train, validation, and test splits as gzip-compressed CSV files), protein-level metadata, amino acid composition statistics, the underlying numerical data for Figs. 2, 4, 5, and 8, and a data dictionary describing all fields. Snapshots of the data and code are additionally available in the GitHub repository described in the Availability of Source Code and Requirements section. The dataset and code follow the FAIR principles [47], with data released under a CC0 public domain dedication and code under the MIT license.
