# Supplementary material for: MPLID (Membrane Protein–Lipid Interaction Database): A Large-Scale Experimental Resource of Residue-Level Protein–Lipid Contacts
Source: Gigascience. 2026 Jul 27;15:giag080. doi: 10.1093/gigascience/giag080 (PMC13435803; doi:10.1093/gigascience/giag080)
Supplement: Supplementary file 1 [file giag080_authors_response_to_reviewer_comments_original_submission.pdf]

Author's Response To Reviewer Comments

We thank the editor and reviewers for their careful evaluation of the manuscript. We have revised the text, data description, limitations, and resource-registration statements in response to the comments below.

Response to Reviewer #1
Reviewer comment:
The MPLID project aims to identify protein-lipid interactions at a residue-by-residue level. They build a database from the entire PDB and thus examines a wide range of lipids and proteins, producing a huge amount of data (8 million residues identified). I think this resource is potentially of considerable value for the training of models for downstream analysis.
The study appears well carried out and the paper well written. That said, I have a few issues with the framing of the study, which I think need to be addressed before publication.
Response: We thank the reviewer for this positive assessment of MPLID and its potential value for downstream modelling.

Reviewer comment: It should be made much clearer in the paper that their approach simply looks at protein-lipid interactions as found in the PDB. This is a very specific and somewhat artificial environment – only certain protein-lipid interactions survive the process of purification (often using harsh detergents). Their much-repeated statement that 1% of all residues are lipid contacting is interesting, but this will of course not reflect biological reality. So upfront it should be made clear that this is for "structural" protein-lipid interactions only, which represent only a small amount of the total. Claims that their data "reflects the biological reality that only a small fraction of residues in membrane proteins make direct lipid contacts" should be dropped. The 1% residue claim should be strongly reconsidered or reframed, as their "non-contact" data will have huge amounts of false-negatives, likely exceeding the "contact" data by an order of magnitude.
Response: We agree with the reviewer and have reframed the manuscript accordingly. The previous statement implying that the 1.00% contact rate reflects biological reality has been removed.
The revised manuscript now describes this value as an observed lower bound based on structurally resolved lipid molecules in PDB entries. We also state that the non-contact class contains false negatives because many native lipid interactions are lost during purification, crystallization, or cryo-EM sample preparation.
The main changes are:
• The Abstract now states that the 1.00% rate is a lower bound and that the non-contact class contains false negatives.
• Section 1.1 and Table 1 now distinguish membrane-zone annotation from direct structural lipid-contact annotation.
• Section 2.1 explains that MPLID captures structurally resolved contacts and cannot be interpreted as a complete map of in vivo lipid binding.
• The Discussion and Conclusions now describe MPLID as a resource for structurally resolved contacts, not as a complete catalogue of biological protein-lipid interactions.
• The Limitations section includes a dedicated paragraph on false negatives in the non-contact class.
We thank the reviewer for this correction; it improved the interpretation of the dataset.

Reviewer comment:
The authors highlight the novelty of their approach, however in their discussion of other methods they omit to mention MemBlob (https://doi.org/10.1093/bioinformatics/btz539). This work takes cryo-EM maps from the PDB and computationally screen the data to identify the membrane and solvent facing regions. They provide the data online, which includes a residue-level description of whether residues face solvent, lipid, lipid interface, or are buried. This tool/repo somewhat overlaps with MPLID and should be cited and described appropriately.
Similarly, BioDolphin (https://doi.org/10.1038/s42004-024-01384-z) which identifies and labels all protein-lipid interactions from the PDB, as defined with PLIP https://plip-tool.biotec.tu-dresden.de/plip-web/plip/index. It provides full information on interactions at a residue level, and is accessible using an API, so overlaps considerably with the MPLID work. It should be cited and described appropriately.

Response: We thank the reviewer for pointing out these omissions. MemBlob and BioDolphin/PLIP are now cited and discussed in the revised manuscript.
Section 1.2 now describes MemBlob as a cryo-EM density-based membrane environment classifier and BioDolphin/PLIP as an interaction-profiling resource for protein-lipid contacts in PDB structures. We also added both resources to Table 2 and revised the comparison section to clarify how they differ from MPLID. In brief, OPM addresses membrane positioning, MemBlob addresses membrane environment inference, BioDolphin/PLIP provides interaction profiling, and MPLID provides pre-computed, ML-ready residue-level contact labels with cluster-aware splits.
The Abstract, Conclusions, and Limitations were also updated to position MPLID as complementary to these resources rather than replacing them.

Reviewer comment: Due to the focus on structural lipids only, there will be a strong favouring for anionic PIP and CDL lipids (hence the increased abundance of Arg and Lys in the data set). Most lipids in biological membranes are not anionic however – this should be mentioned. They also see enrichment of certain polar (Ser/His) and aromatic (Trp/Phe/Tyr). This matches previous analyses on CDL binding (10.1126/sciadv.abh2217), perhaps again reflecting the abundance of anionic lipids in the dataset. This should be discussed.
Response: We agree. The revised amino acid enrichment discussion now explicitly notes that the observed enrichment of arginine and lysine may partly reflect the overrepresentation of anionic lipids, especially cardiolipin and phosphatidylglycerol, among structurally resolved lipid species.
We also cite the cardiolipin binding-site study highlighted by the reviewer and added a Limitations paragraph on PDB and lipid-composition bias. This makes clear that MPLID reflects lipids that are resolved in structures, not the full lipid composition of biological membranes.

Reviewer comment: In terms of the lipid chemical codes used for MPLID in the zenodo link, if these are the codes used for the RSCB PDB then these should be double checked. I looked at a few and got varied results. E.g., LNS appears to be 6-(methylcarbamoyl)pyridine-2-carboxylic acid, which is not a sterol. LNN appears to be (6-hydroxybiphenyl-3-yl)acetic acid, which is not a sterol. GLF is alpha-D-glucopyranosyl fluoride, which is not a sphingolipid.
Response: The reviewer is correct. We audited all 90 PDB Chemical Component Dictionary codes against the RCSB API. The three examples raised by the reviewer are all misclassified in the original candidate list: LNS, LNN, and GLF are not sterol or sphingolipid entries, and none appears in the final MPLID dataset.
The audit identified 45 correctly classified codes, 34 incorrect or ambiguous assignments, and 11 codes absent from the dictionary. These results are now reported transparently in Supplementary Table S1. The manuscript now states that the codes were cross-validated against the RCSB Chemical Component Dictionary during revision.
We retained affected entries where they occur in the dataset because the contact labels are computed from atomic coordinates and do not depend on the functional category label. The revised manuscript now makes this distinction clear so users can exclude specific codes when strict lipid identity is required.

Reviewer comment: If I understand correctly, they've included detergent interactions in their dataset. I find this a very odd choice, and either suggest these are removed or that this is made much clearer up top.
Response: We understand the concern. Detergent mimetics are retained because they are part of the experimental structural context and often occupy membrane-associated surfaces, but their interpretation is now made explicit.
The revised manuscript now includes a protein-level has_detergent_only flag, reports the prevalence of detergent-only proteins, and adds a dedicated Limitations paragraph on detergent mimetics. Users can therefore filter detergent-only entries before model training or downstream biological interpretation.

Reviewer comment: I'm curious about the protein with 4,820 lipid contacts. This PDB (6W4F) has 810 residues, so far fewer than 4,820. Further, the majority of the protein residues are not in contact with the lipids. Could it be that all 20 states in the PDB file have been accounted for by accident?
Response: The reviewer is correct. We checked PDB 6W4F and confirmed that it is an NMR structure of KRAS4B on a lipid bilayer nanodisc with 20 conformational models. The current pipeline counted contacts across all deposited NMR models, which explains the 4,820 contact annotations.
The revised manuscript now discloses this explicitly in the Dataset overview and in a dedicated Limitations paragraph. We also audited the dataset and found 41 NMR multi-model structures affected by this counting

convention, contributing approximately 133,000 excess residue entries and 24,000 excess contact annotations. Future releases will use single-model extraction for NMR structures.

Response to Reviewer #2
Reviewer comment:
This manuscript by Omage and Neshich presents a new tool, MPLID, a large-scale, experimentally derived dataset of residue-level protein-lipid contacts extracted from PDB structures.
The manuscript addresses a conceptual gap between membrane zone annotations (e.g., OPM) and direct lipid-contact residues which is biologically important. The dataset is carefully curated and well documented. This makes the work potentially valuable for machine learning, membrane protein biology, and drug discovery. The manuscript is generally well motivated and appropriate as a Data Note, but there are important methodological and interpretational limitations that should be more clearly acknowledged and, where possible, addressed.
Response: We thank the reviewer for recognizing the conceptual contribution of MPLID and its potential value for machine learning, membrane protein biology, and drug discovery.

Reviewer comment: Lipid contacts resolved in PDB structures are not a random sample of biologically relevant lipid interactions. Certain lipids and proteins are overrepresented. While this is inevitable, the manuscript should include the discussion on the consequences of this such as the bias in training ML models (learning structural detectability rather than biological affinity).
Response: We agree. The Limitations section now includes a paragraph on PDB and lipid-composition bias. It explicitly states that structurally resolved lipid contacts are not a random sample of biological lipid interactions and that models trained on MPLID may learn structural detectability biases in addition to biological binding features. The machine-learning application section also notes that false negatives in the non-contact class may limit achievable performance.

Reviewer comment: If I understood correct, although the dataset includes detergent mimetics and CHARMM lipid codes, the biological interpretation of these contacts differs substantially from native lipid binding. This might create some problems. For example, detergent contacts may reflect solubilization artifacts, not functional lipid sites. This is a significant limitation for users and should be explicitly flagged.
Response: Addressed as above. The revised manuscript flags detergent mimetics explicitly, adds a detergent-only metadata field, and explains how users can filter detergent-only proteins when native lipid interpretation is required.

Reviewer comment: How did the authors determine the single distance cutoff to be 4.0 Å? Does this distance sufficiently distinguish headgroup vs acyl-chain contacts? Or takes orientation into account?
Response: The Methods section now explains the 4.0 Å cutoff. We chose this value to capture direct heavy-atom van der Waals contacts, consistent with common thresholds used in protein-ligand interaction analysis, contact databases, and previous protein-membrane interaction studies. The distance distribution in Figure 3 also supports this cutoff, with a clear peak around 3.5–4.0 Å.
We now state clearly that the cutoff does not distinguish lipid headgroup from acyl-chain contacts and does not encode orientation. Users interested in those distinctions can use the continuous distance values together with lipid atom identities from the original PDB coordinate files.

Reviewer comment: Does the removal of zero-contact proteins introduce selection bias? If that is the case, the dataset cannot support true proteome-wide negative controls and the manuscript should discuss that MPLID is not representative of the full membrane proteome.
Response: Yes. The revised manuscript now states that removing zero-contact proteins introduces selection bias. Section 2.1 explains that MPLID does not represent the complete membrane proteome and cannot be used as a source of proteome-wide negative controls. The same point is repeated in the Limitations section.

Response to Reviewer #3
Reviewer comment:
Lipids are known to interact with membrane proteins and regulate their structure and function; however, because the number of documented interaction examples is limited, the detailed mechanisms of these

interactions remain insufficiently understood. The authors provide a comprehensive database of membrane protein-lipid interactions derived from PDB data. This effort is of extremely high importance for research in this field.
In any case, this manuscript contains important findings and is considered worthy of publication in GigaScience.
Response: We thank the reviewer for this positive assessment and for supporting publication in GigaScience.

Reviewer comment: Lipid species such as PA and PS are not included, and phosphoinositides (PIPs), which are known to bind to various membrane proteins, are also absent. In addition, polyunsaturated fatty acids have not been detected.
Response: We acknowledge this limitation. The revised Lipid type diversity section now states that PA, PS, phosphoinositides, and polyunsaturated fatty acids are absent or underrepresented in the current release. We explain that this reflects the difficulty of resolving these lipids in experimental structures and the current focus of MPLID on lipid species represented in PDB HETATM records. Future releases will expand coverage as more structures with these lipid classes become available.

Reviewer comment: Cholesterol hemisuccinate and cholesteryl hemisuccinate appear to be the same compound. Although these are cholesterol derivatives, they are in fact a type of detergent added artificially.
Response: We checked the PDB Chemical Component Dictionary entries and corrected the manuscript. CHD is cholic acid, not cholesteryl hemisuccinate. Y01 is cholesterol hemisuccinate, an artificial cholesterol derivative used during membrane protein solubilization.
The revised Limitations section now states that cholesterol hemisuccinate is not a native membrane lipid and can be filtered using the detergent-related metadata.

Editorial Manager®

Cookie Preference Center

We use cookies which are necessary to make our site work. We may also use additional cookies to analyze, improve and personalize our content and your digital experience. For more information, see our Cookie Policy.

You may choose not to allow some types of cookies. However, blocking some types may impact your experience of our site and the services we are able to offer. See the different category headings below to find out more or change your settings.

Allow all

Manage Consent Preferences

Strictly Necessary Cookies

Always active

These cookies are necessary for the website to function and cannot be switched off in our systems. They are usually only set in response to actions made by you which amount to a request for services, such as setting your privacy preferences, logging in or filling in forms. You can set your browser to block or alert you about these cookies, but some parts of the site will not then work.

Functional Cookies

These cookies enable the website to provide enhanced functionality and personalisation. They may be set by us or by third party providers whose services we have added to our pages. If you do not allow these cookies then some or all of these services may not function properly.

Performance Cookies

These cookies allow us to count visits and traffic sources so we can measure and improve the performance of our site. They help us to know which pages are the most and least popular and see how visitors move around the site.

Targeting Cookies

These cookies may be set through our site by our advertising partners. They may be used by those companies to build a profile of your interests and show you relevant adverts on other sites. If you do not allow these cookies, you will experience less targeted advertising.

Cookie List

Clear

Apply Cancel

Consent Leg.Interest

Confirm my choices

Powe
