# Supplementary material for: MPLID (Membrane Protein–Lipid Interaction Database): A Large-Scale Experimental Resource of Residue-Level Protein–Lipid Contacts
Source: Gigascience. 2026 Jul 27;15:giag080. doi: 10.1093/gigascience/giag080 (PMC13435803; doi:10.1093/gigascience/giag080)
Supplement: Supplementary file 2 [file giag080_giga-d-26-00059_original_submission.pdf]

GigaScience

MPLID (Membrane Protein-Lipid Interaction Database): A Large-Scale Experimental Resource of Residue-Level Protein-Lipid Contacts

--Manuscript Draft--

**Manuscript Number:**	GIGA-D-26-00059
**Full Title:**	MPLID (Membrane Protein-Lipid Interaction Database): A Large-Scale Experimental Resource of Residue-Level Protein-Lipid Contacts
**Article Type:**	Data Note
**Funding Information:**	Fundação de Amparo à Pesquisa do Estado de São Paulo (2023/02691-2, 2025/23708-6) — Dr Folorunsho Bright Omage
**Abstract:**	see below
**Corresponding Author:**	Goran Neshich<br>Embrapa Informática Agropecuária: Embrapa Agricultura Digital

Abstract:

Background

Membrane proteins constitute approximately 20–30% of all proteomes and represent over 60% of current drug targets. Although protein–lipid interactions play important structural and regulatory roles in membrane-associated proteins, most existing structural resources focus on identifying whether a residue lies within a membrane region, typically inferred from computational hydrophobicity-based positioning algorithms. This approach does not directly address a distinct biological question: which residues at the protein surface make direct physical contact with lipid molecules? Answering this question from experimental data is critical for understanding lipid-mediated allostery, designing lipid-mimetic therapeutics, and training accurate machine learning models for lipid binding site prediction.

Findings

We present MPLID (Membrane Protein-Lipid Interaction Database), a curated residue-level dataset comprising 4,704 membrane proteins representing 813 sequence clusters at 30% identity, 8,055,325 residues, and 80,439 experimentally validated lipid contact annotations (1.00% positive rate). Labels are derived exclusively from crystallized lipid molecules resolved in Protein Data Bank structures, using a 4.0 ˚A all-atom heavy-atom distance cutoff: a residue is labeled as a contact if any of its heavy atoms lies within 4.0 ˚A of any lipid heavy atom, capturing direct van der Waals interactions including side-chain contacts. The dataset recognizes 117 lipid codes spanning phospholipids, cardiolipin, sphingolipids, sterols, fatty acids, glycerolipids, detergent mimetics, lipid A components, and CHARMM simulation nomenclature, covering the full diversity of lipid species encountered in experimental membrane protein structures. To prevent data leakage, proteins are clustered at 30% sequence identity using MMseqs2, yielding 813 clusters that are partitioned into training (2,578 proteins), validation (1,051 proteins), and test (1,075 proteins) splits. Amino acid composition analysis reveals biologically consistent enrichment at lipid contact sites, with tryptophan (1.88×), arginine (1.44×), glycine (1.36×), lysine (1.33×), and phenylalanine (1.23×) showing the strongest enrichment, while negatively charged and β-branched residues are depleted (proline 0.51×, isoleucine 0.57×, aspartate 0.59×).

Conclusions

MPLID addresses a distinct biological question compared to existing membrane zone datasets: identifying residues that directly contact lipid molecules rather than those positioned within the membrane hydrophobic slab. With 4,704 proteins and over 8 million annotated residues, MPLID provides the scale and diversity needed for training deep learning models for lipid contact prediction, a task with direct applications in structure-guided drug design and membrane protein engineering. The dataset adheres to FAIR principles and is freely available under a CC0 public domain dedication. MPLID is intended as a ground-truth resource for residue-level lipid contact annotation and does not aim, by itself, to provide an optimized predictive solution to the full experimental-only classification problem.

Powered by Editorial Manager® and ProduXion Manager® from Aries Systems Corporation

	Campinas, BRAZIL
**Corresponding Author Secondary Information:**	
**Corresponding Author's Institution:**	Embrapa Informática Agropecuária: Embrapa Agricultura Digital
**Corresponding Author's Secondary Institution:**	
**First Author:**	Folorunsho Bright Omage, Ph.D.
**First Author Secondary Information:**	
**Order of Authors:**	Folorunsho Bright Omage, Ph.D.
	Goran Neshich, Ph.D.
**Order of Authors Secondary Information:**	
**Additional Information:**	
**Question**	**Response**
Are you submitting this manuscript to a special series or article collection?	No
**Experimental design and statistics**<br><br>Full details of the experimental design and statistical methods used should be given in the Methods section, as detailed in our Minimum Standards Reporting Checklist. Information essential to interpreting the data presented should be made available in the figure legends.<br><br>Have you included all the information requested in your manuscript?	Yes
**Resources**<br><br>A description of all resources used, including antibodies, cell lines, animals and software tools, with enough information to allow them to be uniquely identified, should be included in the Methods section. Authors are strongly encouraged to cite Research Resource Identifiers (RRIDs) for antibodies, model organisms and tools, where possible.<br><br>Have you included the information requested as detailed in our Minimum Standards Reporting Checklist?	Yes

Powered by Editorial Manager® and ProduXion Manager® from Aries Systems Corporation

**Availability of data and materials**<br><br>All datasets and code on which the conclusions of the paper rely must be either included in your submission or deposited in publicly available repositories (where available and ethically appropriate), referencing such data using a unique identifier in the references and in the “Availability of Data and Materials” section of your manuscript.<br><br>Have you have met the above requirement as detailed in our Minimum Standards Reporting Checklist?	Yes
GigaScience has policies and guidelines in place for the use of generative AI-writing tools such as ChatGPT. If you have used such writing tools to assist with writing the manuscript this must be declared and cited in the text. Authors should not list AI-writing tools and other AI-assisted technologies as an author or co-author and should acknowledge that they are fully responsible for text generated or refined by AI-writing tools.<p><br><br>A summary of use (particularly in the introduction or among methods) needs to be included at the end of the paper, and the outputs should also be included as a supplementary file hosted in GigaDB or other open repositories. Please read our guidelines for more information. <p><br><br>By submitting to GigaScience, you are aware of the journal's AI-writing tools policy, and if you have declared use of such tools below, you have acknowledged this where appropriate in your manuscript and have made a summary of use and outputs available. <p><br><b>AI-assisted writing tools have been used in the preparation of this manuscript?	No

Manuscript

Click here to access/download;Manuscript;main_gigascience_FINAL.pdf

GigaScience, 2026, pp. 1–14

doi:

Data Note

DATA NOTE

MPLID (Membrane Protein–Lipid Interaction Database): A Large-Scale Experimental Resource of Residue-Level Protein–Lipid Contacts

Folorunsho Bright Omage[1,2,*] and Goran Neshich[1,*]

[1]Computational Biology Research Group, Embrapa Digital Agriculture, Av. André Tosello 209, 13083-886, Campinas, São Paulo, Brazil and [2]Biological Chemistry Laboratory, Department of Organic Chemistry, Institute of Chemistry, University of Campinas (UNICAMP), R. Monteiro Lobato 270, 13083-970, Campinas, São Paulo, Brazil

[*]Corresponding authors. omagefolorunsho@gmail.com; goran.neshich@embrapa.br

Abstract

Background: Membrane proteins constitute approximately 20–30% of all proteomes and represent over 60% of current drug targets. Although protein–lipid interactions play important structural and regulatory roles in membrane-associated proteins, most existing structural resources focus on identifying whether a residue lies within a membrane region, typically inferred from computational hydrophobicity-based positioning algorithms. This approach does not directly address a distinct biological question: which residues at the protein surface make direct physical contact with lipid molecules? Answering this question from experimental data is critical for understanding lipid-mediated allostery, designing lipid-mimetic therapeutics, and training accurate machine learning models for lipid binding site prediction. **Findings:** We present MPLID (Membrane Protein-Lipid Interaction Database), a curated residue-level dataset comprising 4,704 membrane proteins representing 813 sequence clusters at 30% identity, 8,055,325 residues, and 80,439 experimentally validated lipid contact annotations (1.00% positive rate). Labels are derived exclusively from crystallized lipid molecules resolved in Protein Data Bank structures, using a 4.0 Å all-atom heavy-atom distance cutoff: a residue is labeled as a contact if any of its heavy atoms lies within 4.0 Å of any lipid heavy atom, capturing direct van der Waals interactions including side-chain contacts. The dataset recognizes 117 lipid codes spanning phospholipids, cardiolipin, sphingolipids, sterols, fatty acids, glycerolipids, detergent mimetics, lipid A components, and CHARMM simulation nomenclature, covering the full diversity of lipid species encountered in experimental membrane protein structures. To prevent data leakage, proteins are clustered at 30% sequence identity using MMseqs2, yielding 813 clusters that are partitioned into training (2,578 proteins), validation (1,051 proteins), and test (1,075 proteins) splits. Amino acid composition analysis reveals biologically consistent enrichment at lipid contact sites, with tryptophan (1.88×), arginine (1.44×), glycine (1.36×), lysine (1.33×), and phenylalanine (1.23×) showing the strongest enrichment, while negatively charged and β-branched residues are depleted (proline 0.51×, isoleucine 0.57×, aspartate 0.59×). **Conclusions:** MPLID addresses a distinct biological question compared to existing membrane zone datasets: identifying residues that directly contact lipid molecules rather than those positioned within the membrane hydrophobic slab. With 4,704 proteins and over 8 million annotated residues, MPLID provides the scale and diversity needed for training deep learning models for lipid contact prediction, a task with direct applications in structure-guided drug design and membrane protein engineering. The dataset adheres to FAIR principles and is freely available under a CC0 public domain dedication. MPLID is intended as a ground-truth resource for residue-level lipid contact annotation and does not aim, by itself, to provide an optimized predictive solution to the full experimental-only classification problem.

Key words: membrane proteins, lipid-protein interactions, protein structure, machine learning dataset, structural biology, experimental validation, residue-level classification, FAIR data

Background

Membrane proteins are central to virtually all cellular processes, mediating transport of ions and solutes, signal transduction, energy conversion, and cell adhesion Wallin and von Heijne [1998]. Genomic analyses consistently estimate that 20–30% of open reading frames in any given proteome encode membrane proteins Wallin and von Heijne [1998], and pharmacological surveys have established that these proteins account for more than 60% of all current drug targets Overington et al. [2006], Santos et al. [2017]. Despite their biomedical importance, membrane proteins remain among the

© The Author 2026. Published by Oxford University Press. All rights reserved. For permissions, please e-mail: journals.permissions@oup.com

most challenging targets for structural and computational biology, in large part because their native functional context, the lipid bilayer, is difficult to recapitulate experimentally and model computationally.

A growing body of evidence demonstrates that lipid-protein interactions are not passive consequences of membrane embedding but rather active modulators of protein structure, dynamics, and function Corradi et al. [2019], Laganowsky et al. [2014]. Specific lipid binding events regulate ion channel gating Duncan et al. [2020], G protein-coupled receptor (GPCR) signaling Corradi et al. [2019], and transporter conformational cycling Lee [2004]. Cholesterol, for example, occupies defined binding sites on GPCRs that are structurally resolved at atomic resolution Hanson et al. [2008], and cardiolipin binding is essential for the function of mitochondrial respiratory chain complexes Contreras et al. [2011]. The diversity of lipid species in biological membranes, encompassing phospholipids, sphingolipids, sterols, and glycolipids Harayama and Riezman [2018], van Meer et al. [2008], creates a complex interaction landscape that shapes the behavior of membrane-embedded proteins.

Membrane zone versus lipid contact: two distinct questions

A fundamental distinction exists between two related but different biological questions in membrane protein structural biology. The first question, “Is this residue located within the membrane zone?”, has been addressed by computational databases such as the Orientations of Proteins in Membranes (OPM) database Lomize et al. [2012, 2006]. OPM positions membrane proteins in a hydrophobic slab model using energy minimization against experimentally calibrated transfer free energy parameters, producing membrane boundaries for over 15,000 protein entries. This approach is invaluable for understanding membrane protein topology, but the resulting labels reflect a coarse spatial localization: typically 20–40% of residues in a transmembrane protein fall within the OPM-defined membrane zone.

The second question, “Does this residue make direct physical contact with a lipid molecule?”, requires experimental structural evidence. Crystallographic and cryo-electron microscopy structures frequently resolve lipid molecules at specific protein surface sites, revealing native lipid interaction partners at atomic resolution. These experimental contacts represent a far more stringent and specific annotation: in our dataset, only 1.00% of all residues in membrane proteins (including both surface-exposed and buried residues) make lipid contacts within 4.0 Å (using an all-atom distance criterion), a rate approximately 20- to 40-fold lower than the membrane zone positive rate.

This distinction has profound consequences for both biological interpretation and machine learning model development (Table 1). Membrane zone labels identify the general region of a protein that is embedded in the bilayer, while lipid contact labels pinpoint specific binding sites that may mediate functionally important lipid-protein interactions. For drug discovery, the latter is far more actionable: lipid contact sites represent potential targets for allosteric modulation by lipid-mimetic compounds Chatzigoulas and Cournia [2022a], while membrane zone annotations provide no such site-level resolution. We emphasize that no OPM-derived residue labels were used at any stage of the MPLID construction pipeline; all residue annotations derive exclusively from

Table 1. Comparison of membrane zone and lipid contact prediction tasks. These represent fundamentally different biological questions with distinct label sources, positive rates, and downstream applications.

Aspect	Membrane zone	Lipid contact
Label source	Computational (OPM)	Experimental (PDB)
Biological question	In membrane slab?	Contacts lipid?
Positive rate	20–40%	∼1.00%
Spatial resolution	Zone-level (∼30 Å)	Atomic (4.0 Å)
Validation	In silico energy	X-ray/cryo-EM
Key application	Topology prediction	Binding site identification

experimentally resolved lipid molecules in PDB structures. OPM is discussed here solely to distinguish the complementary biological questions of membrane positioning versus direct lipid contact.

Existing resources and their limitations

Several resources provide information about membrane protein-lipid interactions, each with specific scope and limitations. The OPM database Lomize et al. [2012] remains the definitive resource for membrane protein positioning, providing computationally derived membrane boundaries for transmembrane and peripheral proteins. However, OPM labels represent theoretical membrane zones rather than experimentally resolved lipid interaction sites.

The MemProtMD database Newport et al. [2019], Stansfeld et al. [2015] complements OPM by providing coarse-grained molecular dynamics (CG-MD) simulations of membrane proteins embedded in explicit phospholipid bilayers. While MemProtMD offers valuable insights into lipid organization around proteins, its contacts derive from simulations rather than experimental structures, and the database focuses on integral membrane proteins in a single lipid type (dipalmitoylphosphatidylcholine).

The STING Relational DataBase (RDB) Neshich et al. [2003, 2006] provides a comprehensive structural analysis platform that computes and stores inter-atomic contacts, surface accessibility, and residue-level descriptors for all PDB structures. While STING RDB does not specifically label lipid contacts, its contact calculation infrastructure and residue-level annotation framework informed the design philosophy of MPLID, particularly regarding the use of distance-based contact definitions and the importance of providing both binary labels and continuous distance values for downstream analysis.

On the prediction methodology side, the DREAMM method Chatzigoulas and Cournia [2022b] demonstrated that ensemble machine learning can achieve a Matthews correlation coefficient (MCC) of 0.84 for predicting protein-membrane interfaces, using a curated set of 54 peripheral membrane proteins (later expanded to 65) with experimentally characterized membrane-penetrating residues. However, this training data was distributed as part of the prediction tool rather than as a standalone, documented dataset resource. More recently, PMIpred introduced a physics-informed transformer approach trained on molecular dynamics data for peptide-membrane interactions van Hilten et al. [2024], and protein language model approaches have been explored using ProtTrans embeddings Paranou et al. [2024], Elnaggar et al. [2022]. While these studies

have advanced prediction methodology, none provides a large-scale, publicly deposited, and documented dataset covering the full diversity of membrane protein classes.

The need for large-scale experimental lipid contact data

The convergence of three trends motivates the creation of MPLID. First, the rapid growth of the Protein Data Bank Berman et al. [2000], Burley et al. [2023], which now contains more than 230,000 structures, means that thousands of membrane protein structures with resolved lipid molecules are available for systematic analysis. Second, advances in protein language models such as ESM-2 Lin et al. [2023] and ProtTrans Elnaggar et al. [2022] have demonstrated that per-residue embeddings trained on large-scale protein sequence data can capture structural and functional properties, but training supervised models on top of these representations requires large labeled datasets. Third, structure-guided drug discovery increasingly recognizes lipid binding sites as druggable targets for allosteric modulation Chatzigoulas and Cournia [2022a], Duncan et al. [2020], creating demand for systematic catalogs of experimentally validated lipid interaction sites.

MPLID addresses these needs by providing experimentally validated lipid contact annotations for 4,704 membrane proteins containing 8,055,325 residues. All labels derive from crystallized lipid molecules in PDB structures, ensuring experimental ground truth at atomic resolution. Unlike prior resources that are limited in scale or scope, MPLID covers integral, peripheral, and single-pass membrane proteins across 813 sequence-diverse clusters.

Data Description

Dataset overview

MPLID provides residue-level binary annotations indicating whether each residue in a membrane protein makes direct physical contact with a crystallized lipid molecule. The complete dataset encompasses 4,704 membrane proteins with a total of 8,055,325 residues, of which 80,439 (1.00%) are labeled as lipid contacts (Figure 1). This positive rate reflects the biological reality that only a small fraction of residues in membrane proteins make direct lipid contacts within 4.0 Å, primarily at protein surfaces, interfacial regions, and lipid-binding pockets. The remaining residues are located in solvent-exposed domains, protein-protein interfaces, or interior regions of multi-subunit complexes.

Proteins in the dataset range from 22 to 15,280 residues (median 1,038; mean 1,712), reflecting the diversity of membrane protein sizes from small single-pass transmembrane peptides to large multi-subunit complexes. The number of lipid contacts per protein ranges from 1 to 4,820 (median 4; mean 17), with the highly skewed distribution reflecting that most proteins have few resolved lipid contacts while a small number of well-characterized complexes have extensive lipid interactions. Every protein in the final dataset has at least one lipid contact, as proteins with zero contacts were removed during quality filtering (see Methods).

Data schema and format

Each record in MPLID contains ten fields that provide complete information for each annotated residue. The `pdb_id` field contains the four-character PDB accession code, and `chain_id` specifies the polypeptide chain within the structure. The `residue_number` gives the residue's position in the chain, while `residue_name` provides the three-letter amino acid code. The binary label `is_contact` takes a value of 1 for residues where any heavy atom is within 4.0 Å of any lipid heavy atom and 0 otherwise. The `label_source` field is set to "EXPERIMENTAL" for all entries, confirming that labels derive from experimentally resolved lipid molecules in PDB structures rather than computational predictions. The `confidence` field is set to "high" for all entries in the current release because all labels derive from experimentally resolved structures; this field is retained as a placeholder for future quality scoring that may incorporate structure resolution and B-factor data. The `min_distance` field records the minimum distance in Angstroms between any heavy atom of the residue and the nearest lipid heavy atom. Finally, `cluster_id` assigns each protein to a sequence cluster, and `split` indicates assignment to training, validation, or test partitions.

Data are distributed as gzip-compressed comma-separated values (CSV) files partitioned by split (train, validation, test), along with a protein-level metadata file containing per-protein summary statistics including residue counts, contact counts, contact rates, cluster assignments, and split assignments.

Train/validation/test splits

The dataset is partitioned into training (2,578 proteins; 4,907,696 residues), validation (1,051 proteins; 1,403,838 residues), and test (1,075 proteins; 1,743,791 residues) splits (Figure 1). Critically, splitting is performed at the protein level with cluster-aware stratification: proteins within the same sequence cluster are always assigned to the same split, preventing data leakage from sequence-similar proteins appearing in both training and evaluation partitions. Contact rates vary across splits (training 1.16%, validation 0.69%, test 0.79%) because stratification is performed at the cluster level rather than the residue level, and clusters with higher contact rates may be unevenly distributed. This variation is expected and should be accounted for when comparing model performance across splits. This design choice is essential for meaningful generalization assessment, as protein-level leakage can dramatically inflate performance estimates in structural bioinformatics tasks.

The cluster-aware splitting strategy uses MMseqs2 Steinegger and Söding [2017] with a 30% sequence identity threshold and 80% alignment coverage, producing 813 clusters. Entire clusters are assigned to splits to ensure that no protein in the validation or test sets shares more than 30% sequence identity with any training protein. This threshold is substantially more conservative than the 40% identity commonly used in protein function prediction benchmarks Sillitoe et al. [2021], further reducing the risk of overestimating generalization performance.

Sequence clustering analysis

The 813 sequence clusters exhibit a highly skewed size distribution (Figure 2), with 373 singletons (45.9%) and a largest cluster of 173 proteins, reflecting the sequence diversity of membrane proteins across different families and superfamilies. This clustering pattern indicates substantial structural and functional diversity in the dataset, as singleton clusters represent unique membrane protein families without close homologs.

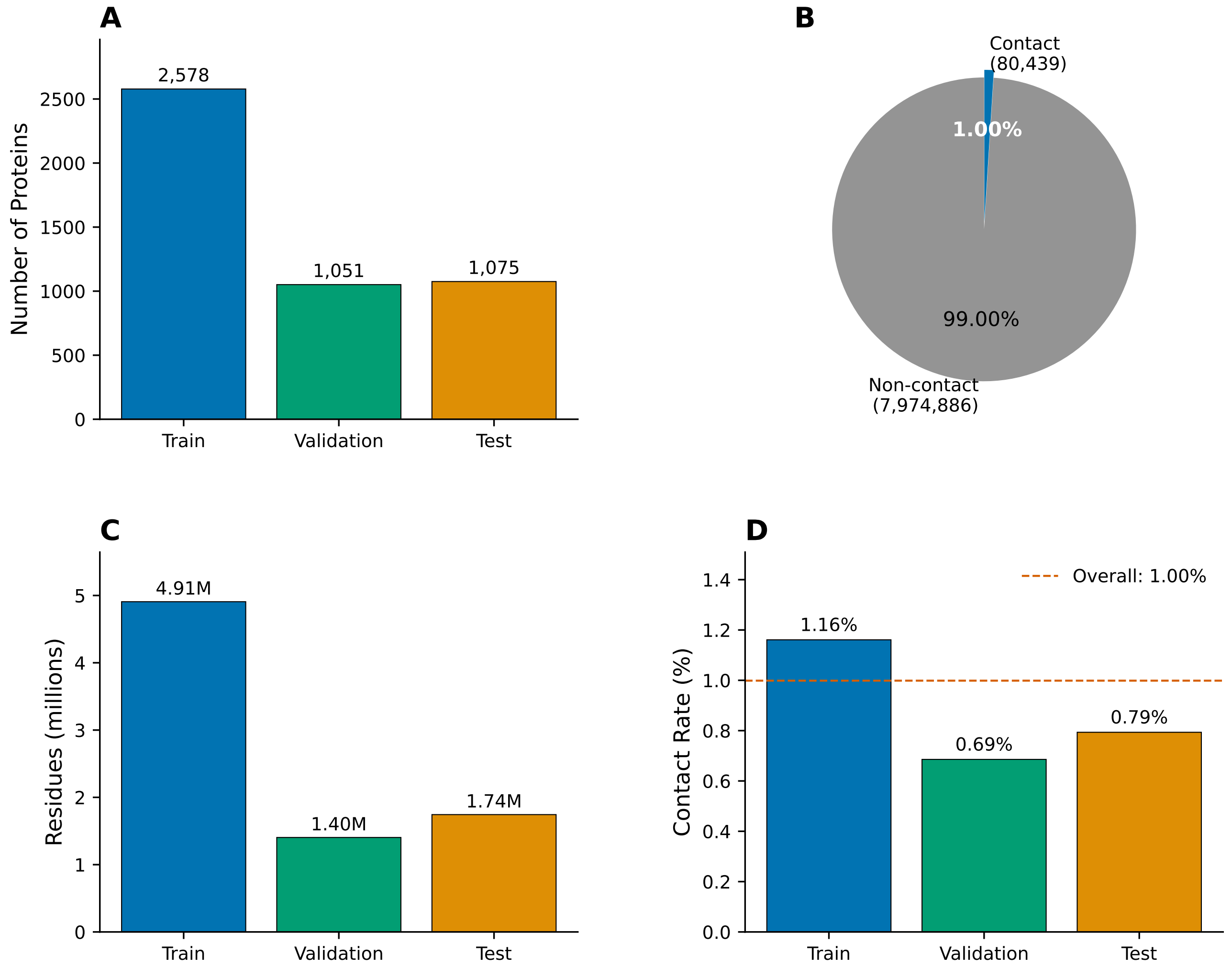

Fig. 1. MPLID dataset overview. (A) Distribution of proteins across training (54.8%), validation (22.3%), and test (22.9%) splits. (B) Class distribution showing the imbalance between contact (1.00%) and non-contact (99.00%) residues. (C) Total residues per split. (D) Contact rates across splits, showing variation due to cluster-level stratification: training (1.16%), validation (0.69%), and test (0.79%).

Analyses

Amino acid composition at lipid contact sites

Analysis of amino acid enrichment at lipid contact sites reveals biologically meaningful and interpretable patterns (Figure 3). Enrichment ratios were calculated as the fraction of each amino acid among contact residues divided by its fraction in the complete dataset. Tryptophan (1.88×) shows the strongest enrichment, consistent with the well-established role of aromatic residues in anchoring transmembrane proteins at the membrane-water interface through favorable interactions with lipid headgroups via cation-π and hydrogen bonding interactions Corradi et al. [2019]. Notably, positively charged residues are strongly enriched: arginine (1.44×) and lysine (1.33×) rank among the top five most enriched amino acids, consistent with the "positive-inside rule" von Heijne [1992] and with the electrostatic attraction of cationic side chains to anionic phospholipid headgroups. Glycine (1.36×) is enriched, reflecting its prevalence in GxxxG motifs that mediate transmembrane helix-helix packing and create lipid-accessible surfaces at helix interfaces Russ and Engelman [2000]. Phenylalanine (1.23×) and tyrosine (1.08×) are moderately enriched, contributing to the aromatic belt. Among other residues, cysteine (1.15×), serine (1.13×), and histidine (1.11×) show mild enrichment, with histidine functioning as an interfacial anchoring residue through its amphipathic imidazole ring, and cysteine enrichment likely reflecting palmitoylation sites and disulfide-constrained loop regions at lipid interfaces. Leucine (1.08×) is slightly enriched, reflecting its involvement in direct side-chain contacts with lipid acyl chains.

Among depleted residues, proline (0.51×) and isoleucine (0.57×) show the strongest depletion. Proline disrupts regular secondary structure and is underrepresented at smooth lipid-protein interfaces. The depletion of isoleucine, a β-branched hydrophobic residue, may reflect its preference for buried core positions rather than surface lipid contacts. Aspartate (0.59×) is depleted, consistent with the electrostatic incompatibility of negatively charged side chains with the lipid bilayer interior. Asparagine (0.71×) and valine (0.73×) are moderately depleted. Threonine (0.87×) and glutamine (0.89×) show mild depletion, while alanine (0.94×) and glutamate (0.97×) are near neutral. Overall, the enrichment

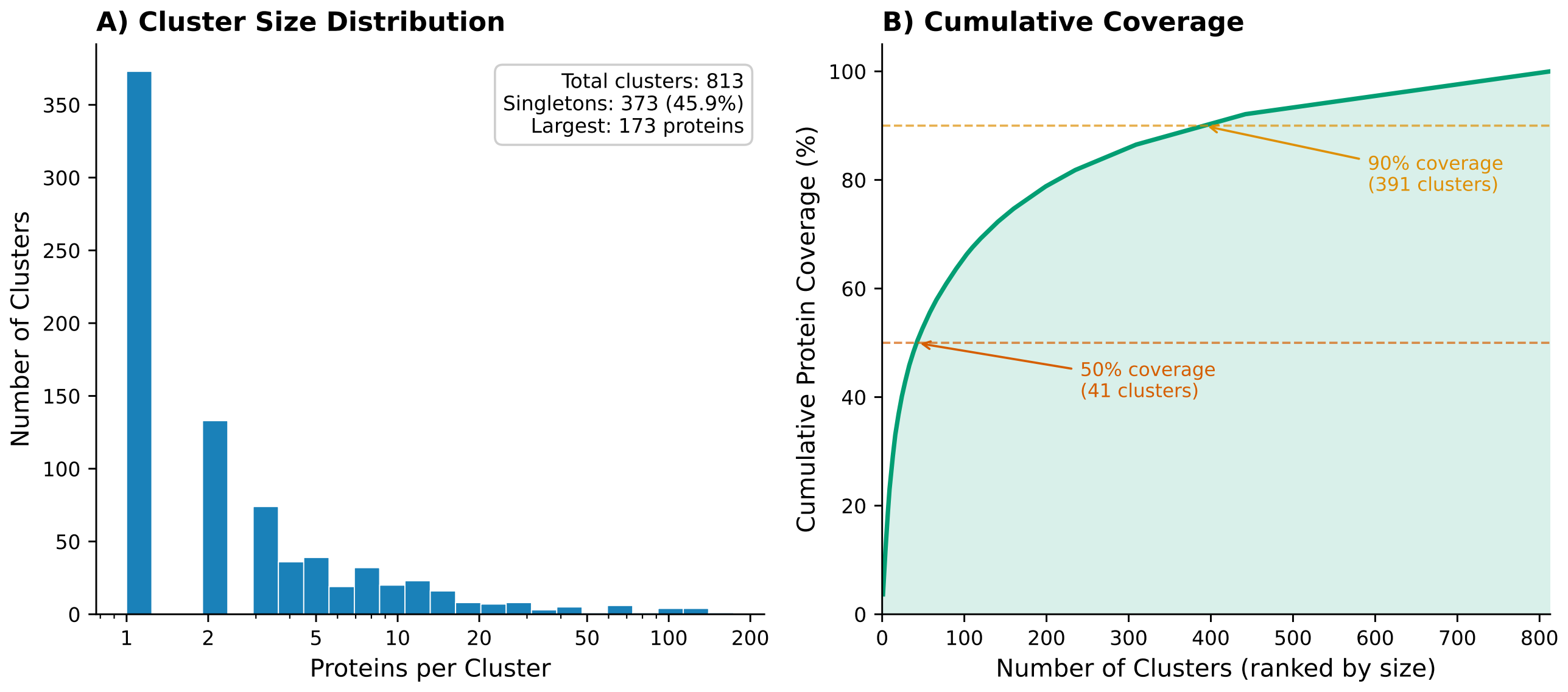

Fig. 2. Sequence cluster analysis. (A) Distribution of cluster sizes at 30% sequence identity on a log scale, showing a highly skewed size distribution with many singletons and a small number of large clusters containing many homologous structures. (B) Cumulative protein coverage ranked by cluster size, illustrating the concentration of proteins in a few large families.

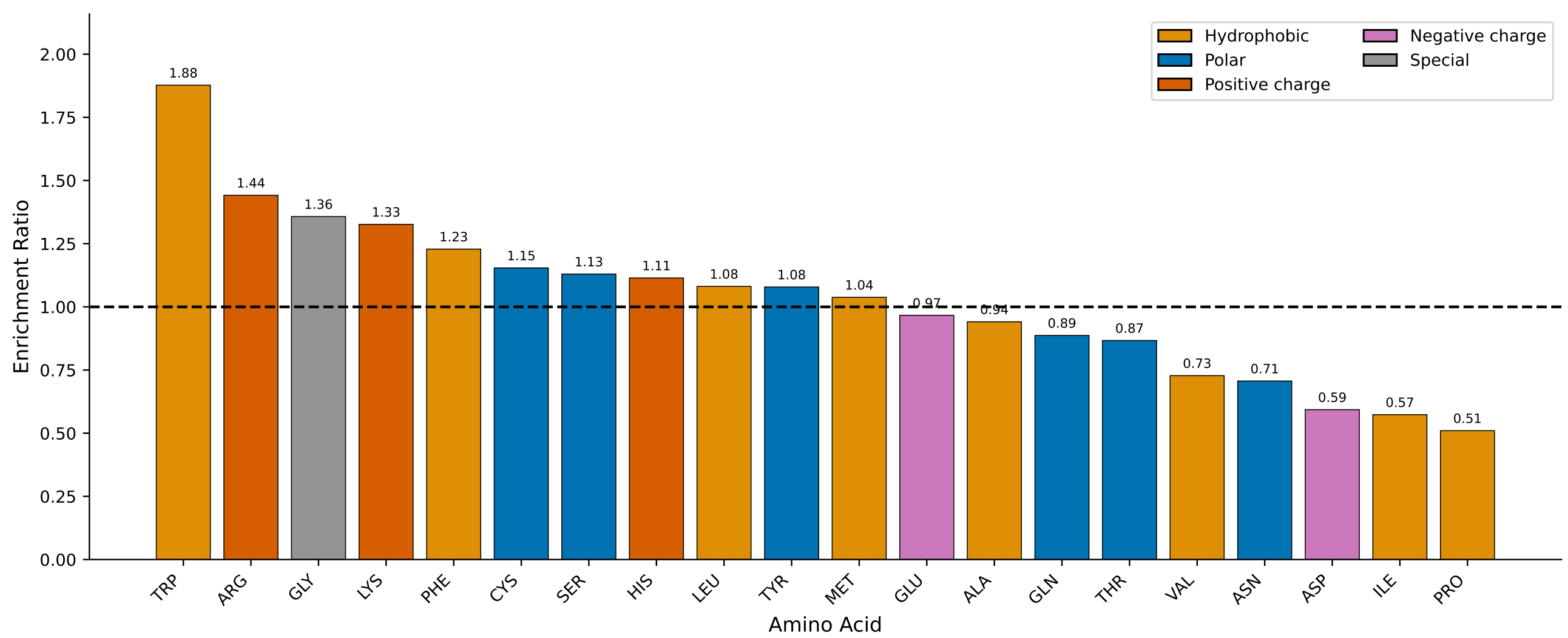

Fig. 3. Amino acid enrichment at lipid contact sites. Enrichment ratios are calculated as the frequency of each amino acid among contact residues divided by its overall frequency in the dataset. Colors indicate amino acid physicochemical properties: hydrophobic (orange), polar (blue), positive charge (red), negative charge (purple), and special (gray). The dashed line marks the expected frequency (1.0). Values above 1.0 indicate enrichment; values below 1.0 indicate depletion.

pattern strongly supports the biological validity of the all-atom contact definition: residues capable of electrostatic interactions with phospholipid headgroups (arginine, lysine) and aromatic anchoring at the interface (tryptophan, phenylalanine) are enriched, while conformationally constrained (proline) and β-branched (isoleucine, valine) residues are depleted. Given the large sample sizes (80,439 contact residues among 8,055,325 total residues), these enrichment and depletion patterns are robust and unlikely to arise from sampling variation.

Distance distribution analysis

The distribution of minimum distances between protein residues and lipid molecules provides independent validation of the 4.0 Å contact cutoff (Figure 4). Among contact residues, the distance distribution shows a clear peak at approximately 3.5–4.0 Å, corresponding to typical van der Waals contact distances between heavy atoms. The sharp boundary at 4.0 Å indicates that the cutoff effectively captures the transition from direct physical contact to non-contact. For non-contact residues, the distribution is broadly shifted toward larger distances, with a peak near 8–12 Å and a long tail extending to distances greater

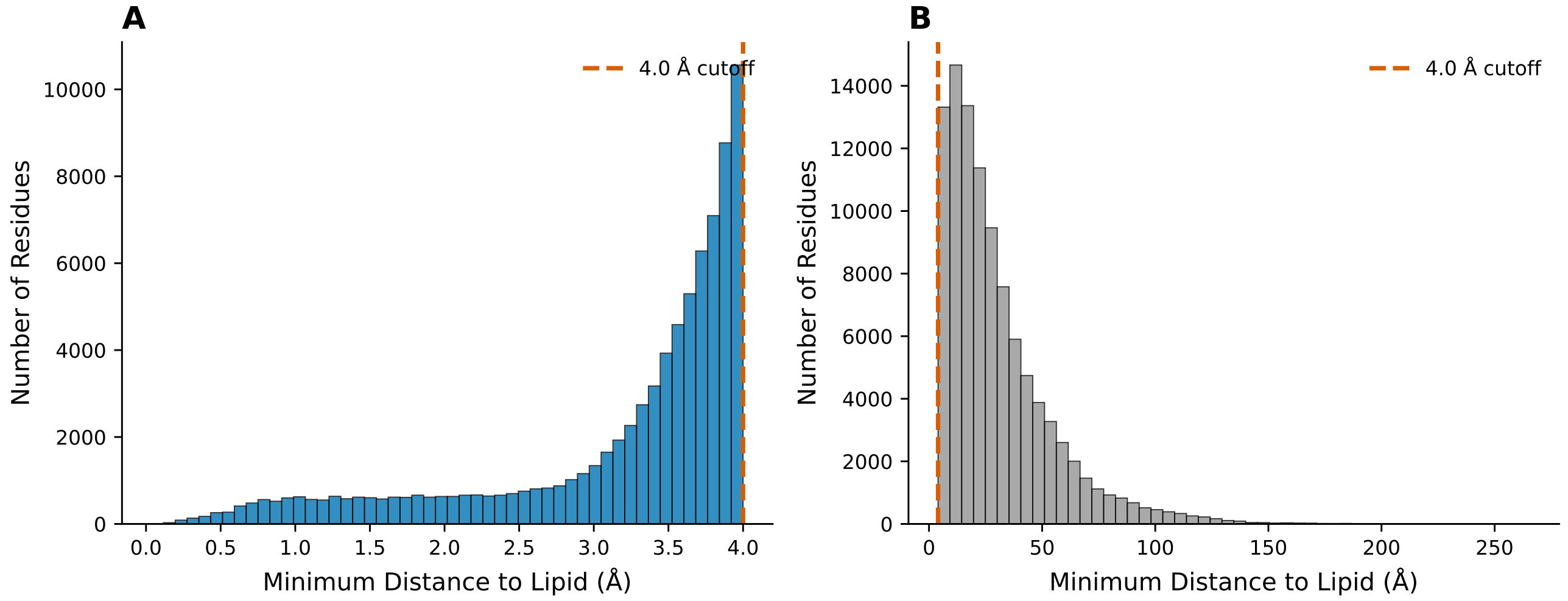

Fig. 4. Distribution of minimum residue-to-lipid distances. (A) Contact residues (≤4.0 Å) show a peak near 3.5–4.0 Å, consistent with van der Waals contact distances. (B) Non-contact residues display a broad distribution with distances extending well beyond 30 Å, confirming clear separation between contacting and non-contacting populations.

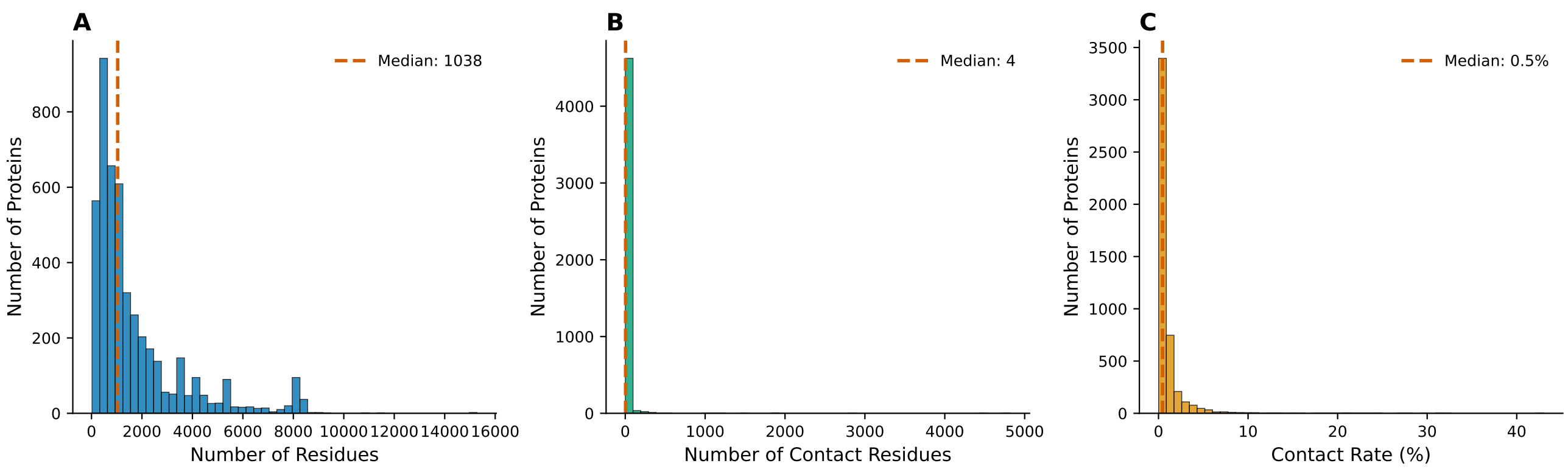

Fig. 5. Protein-level statistics. Distribution of protein sizes and contact densities across the MPLID dataset, illustrating the diversity of membrane protein families represented.

than 30 Å for residues in soluble domains far from any lipid molecule.

Protein size and contact density distributions

The distribution of protein sizes in MPLID spans three orders of magnitude, reflecting the diversity of membrane protein families represented (Figure 5). Contact density, defined as the fraction of residues making lipid contacts, varies substantially across proteins, with a per-protein median contact rate of 0.45% and a mean of 0.99%. The overall contact rate of 1.00% reflects a severe class imbalance (approximately 100:1 negative-to-positive ratio) inherent to this prediction task, which requires specialized machine learning approaches such as class weighting or focal loss.

Comparison with existing resources

Table 2 places MPLID in the context of existing membrane protein-lipid interaction resources (Figure 6). Each resource addresses a different aspect of the problem. OPM Lomize et al. [2012] provides computationally derived membrane zone boundaries for topology analysis. MemProtMD Newport et al. [2019] offers coarse-grained simulation data for lipid organization. The DREAMM prediction method Chatzigoulas and Cournia [2022b] used a curated set of 54 peripheral membrane proteins with experimentally characterized membrane-penetrating residues as training data, though this data was not released as a standalone documented dataset. MPLID fills this gap by providing the first large-scale, publicly deposited, and fully documented dataset of experimental lipid contact labels, covering 4,704 proteins across all membrane protein classes with FAIR-compliant metadata, a data dictionary, and pre-defined evaluation splits. The diversity of membrane protein families represented in MPLID, spanning 813 sequence clusters at 30% identity, ensures broad coverage of the membrane protein structural landscape. These resources are complementary rather than competing: researchers should select the appropriate resource based on their specific biological question, and models trained on one type of label may not transfer directly to another without domain adaptation.

MPLID and OPM address different biological questions and should be viewed as complementary rather than competing resources. OPM provides membrane zone annotations that are useful for topology prediction and membrane protein

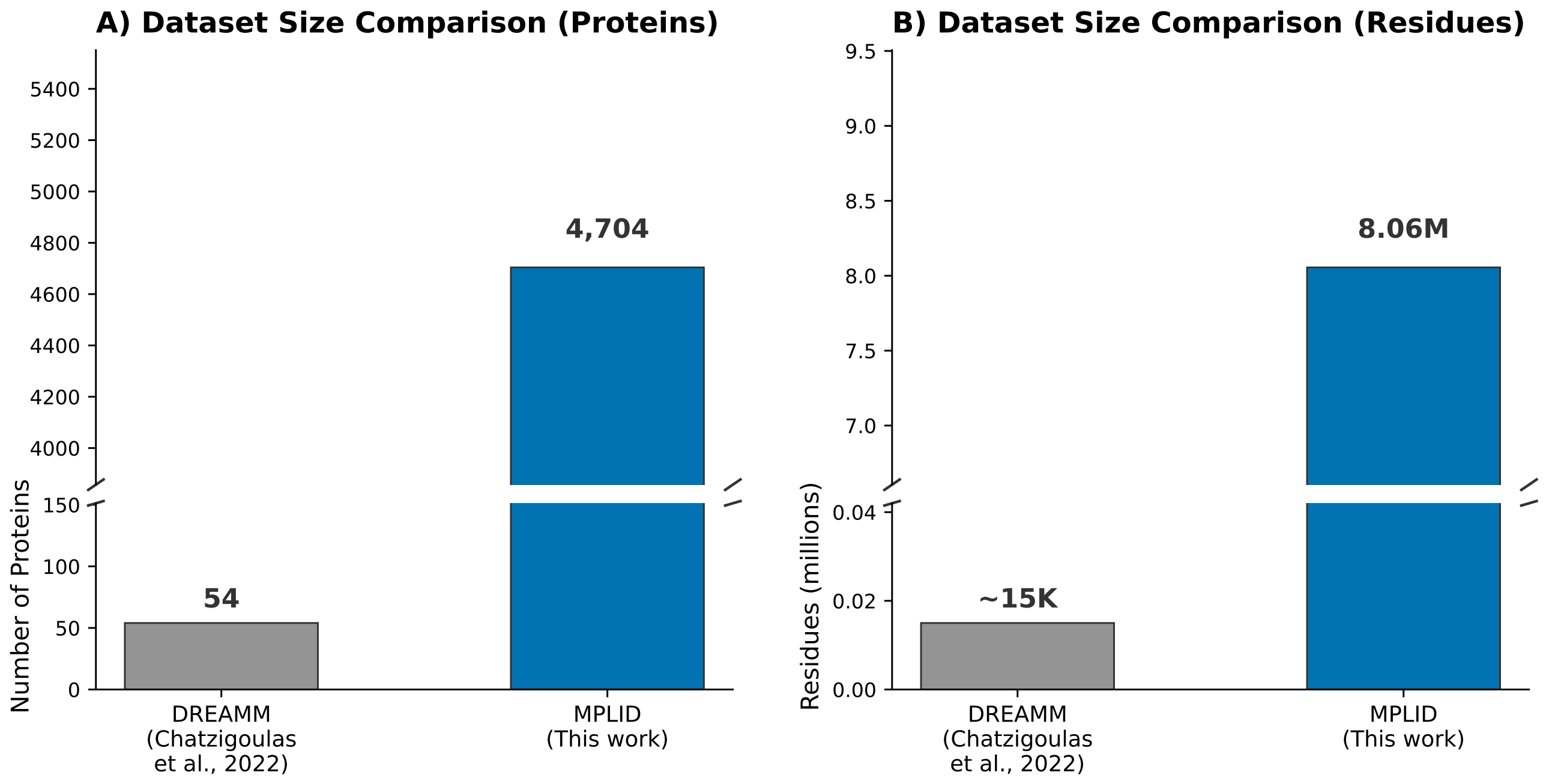

Fig. 6. Scale comparison between MPLID and the training data used by the DREAMM prediction method Chatzigoulas and Cournia [2022b], the largest prior collection of experimentally annotated membrane protein-lipid interactions. (A) Number of proteins (4,704 versus 54). (B) Number of annotated residues (8,055,325 versus ∼15,000). Note that these resources differ in scope: DREAMM focuses on peripheral membrane proteins, while MPLID covers all membrane protein classes.

Table 2. Comparison of MPLID with existing membrane protein-lipid interaction resources. MPLID is the first large-scale, publicly deposited dataset providing experimentally validated residue-level lipid contact labels with FAIR-compliant documentation.

Resource	Proteins	Residues	Label type	Label source
MPLID (this work)	4,704	8,055,325	Lipid contact	Experimental (PDB)
DREAMM Chatzigoulas and Cournia [2022b][a]	54	∼15,000	Membrane interface	Experimental (curated)
OPM Lomize et al. [2012]	15,096	–	Membrane zone	Computational
MemProtMD Newport et al. [2019]	∼3,500	–	Lipid contact	Simulated (CG-MD)

[a]Training data distributed within the DREAMM prediction tool repository; not released as a standalone documented dataset.

classification, while MPLID provides lipid contact annotations that are useful for binding site identification and lipid-mediated function analysis. Models trained on one type of label cannot be directly evaluated on the other, and researchers should select the appropriate dataset based on their specific biological question.

Lipid type diversity

MPLID recognizes 117 lipid codes organized into ten functional categories, comprising 90 PDB Chemical Component Dictionary codes and 27 CHARMM simulation lipid identifiers commonly found in cryo-EM depositions. Phospholipids include cardiolipin (CDL, found in 675 structures), phosphocholine variants (PCW, 196 structures), phosphoethanolamine (PEE, 273 structures), and phosphoglycerol (PGV, 144 structures). Sterols are represented by cholesterol (CLR, 1,119 structures) and derivatives including cholesteryl hemisuccinate (CHD, 151 structures) and cholesterol hemisuccinate (Y01). Fatty acids include palmitate (PLM, 734 structures), oleic acid (OLA, 379 structures), myristate (MYR, 426 structures), and stearate (STE, 94 structures). Sphingolipids include sphingosine (SPH, 102 structures) and sphingosine-1-phosphate (S1P, 16 structures). Detergent mimetics used in crystallization, which often occupy native lipid binding sites, include lauryl maltose neopentyl glycol (LMT, 677 structures), octyl β-D-glucopyranoside (BOG, 510 structures), and lauryldimethylamine oxide (LDA, 263 structures). This comprehensive lipid recognition ensures that MPLID captures the full diversity of protein-lipid interactions observed in experimental structures.

Structural visualization of lipid contacts

To illustrate the nature of the lipid contact annotations in MPLID, representative membrane protein structures with their resolved lipid molecules are shown in Figure 7. These examples demonstrate that lipid contacts occur at specific, well-defined sites on the protein surface rather than uniformly across the membrane-exposed region. Contact residues (highlighted in red/orange) are interspersed with non-contact residues even in transmembrane regions, confirming that lipid contact prediction is a more specific task than membrane zone classification.

Computational label comparison studies

The availability of MPLID alongside OPM membrane zone annotations enables systematic comparison of experimental

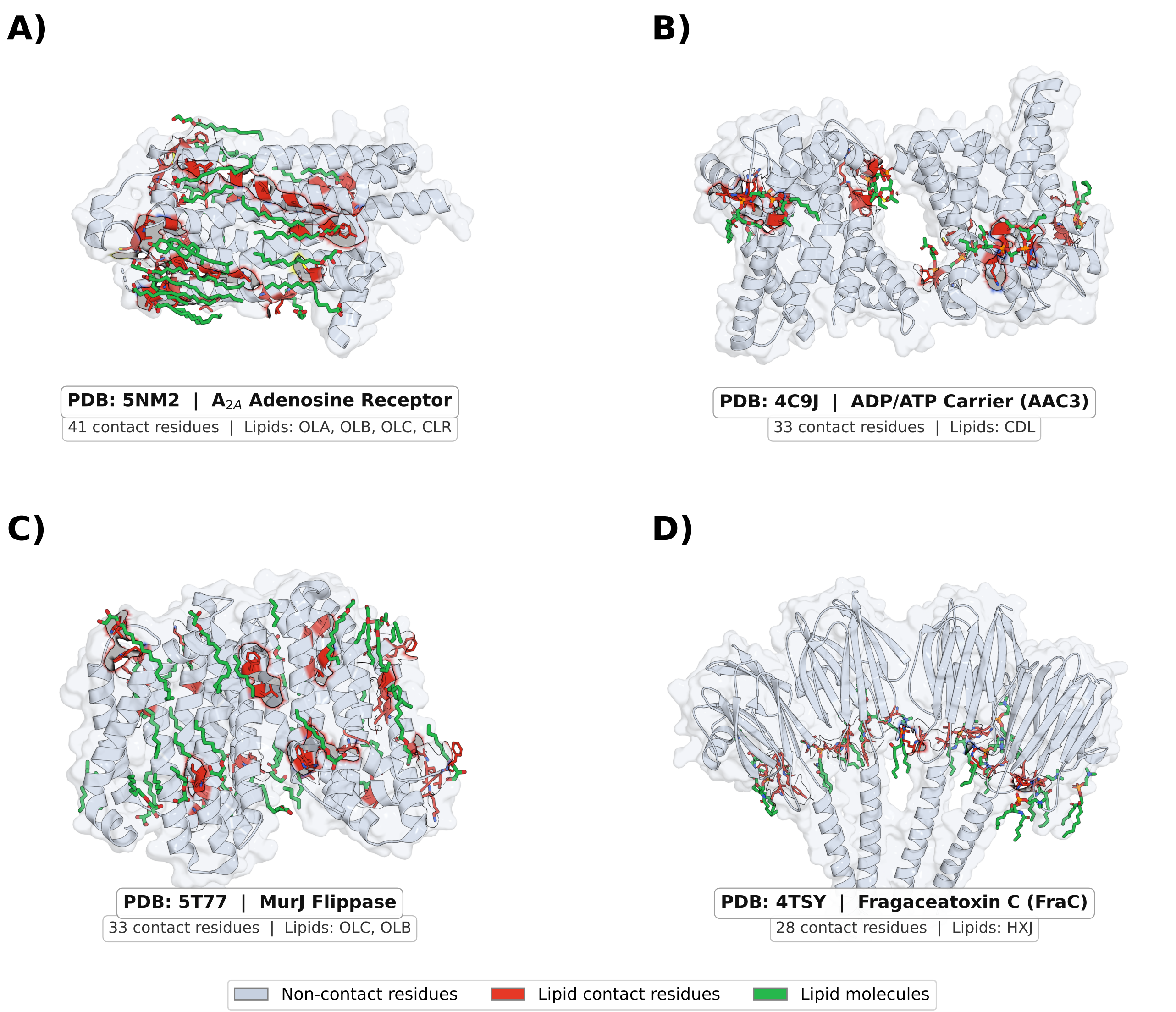

Fig. 7. Structural visualization of MPLID lipid contact annotations across four representative membrane protein classes. (A) A_{2A} adenosine receptor (PDB: 5NM2), a GPCR with multiple lipid types including oleic acid and cholesterol. (B) ADP/ATP carrier AAC3 (PDB: 4C9J), a mitochondrial transporter with cardiolipin contact residues across two chains. (C) MurJ flippase (PDB: 5T77), a lipid II flippase with contacts distributed across its transmembrane helices. (D) Fragaceatoxin C (PDB: 4TSY), a tetrameric pore-forming toxin with sphingolipid contacts at the membrane insertion interface. These examples demonstrate that lipid contacts are site-specific rather than uniformly distributed across membrane-exposed surfaces.

lipid contacts with computational membrane boundaries. We compared MPLID contact labels with OPM-derived membrane zone assignments across proteins present in both resources (Figure 8). The number of overlapping proteins varies by analysis: 2,383 proteins contributed to precision estimates, 1,685 to recall estimates, and 2,457 to contact distribution analysis, reflecting differences in the availability of OPM membrane zone annotations and non-zero lipid contacts across the shared set. Of all MPLID lipid contacts in the shared set (n = 28,497 residues), 51.2% fell within the OPM membrane zone while 48.8% were located outside the computationally defined boundaries, likely reflecting interfacial contacts, re-entrant loops, or amphipathic helices that extend beyond the hydrophobic core. Conversely, 98.8% of OPM membrane zone residues (n = 1,170,590) showed no direct lipid contact within 4.0 Å, and the per-protein median was only 0.7% of membrane zone residues having lipid contacts. The OPM membrane zone captured a median of 50.0% of lipid contacts per protein. These dataset-wide statistics highlight the fundamental difference between membrane zone classification and lipid contact prediction: being embedded in the membrane is neither sufficient nor necessary for direct lipid interaction. Researchers working with MemProtMD Newport et al. [2019] simulations can similarly compare simulated lipid contacts with the experimental contacts provided by MPLID.

Methods

Data source integration

MPLID was constructed using a purely experimental approach with no dependence on computational membrane positioning databases. OPM data were not used at any stage of the

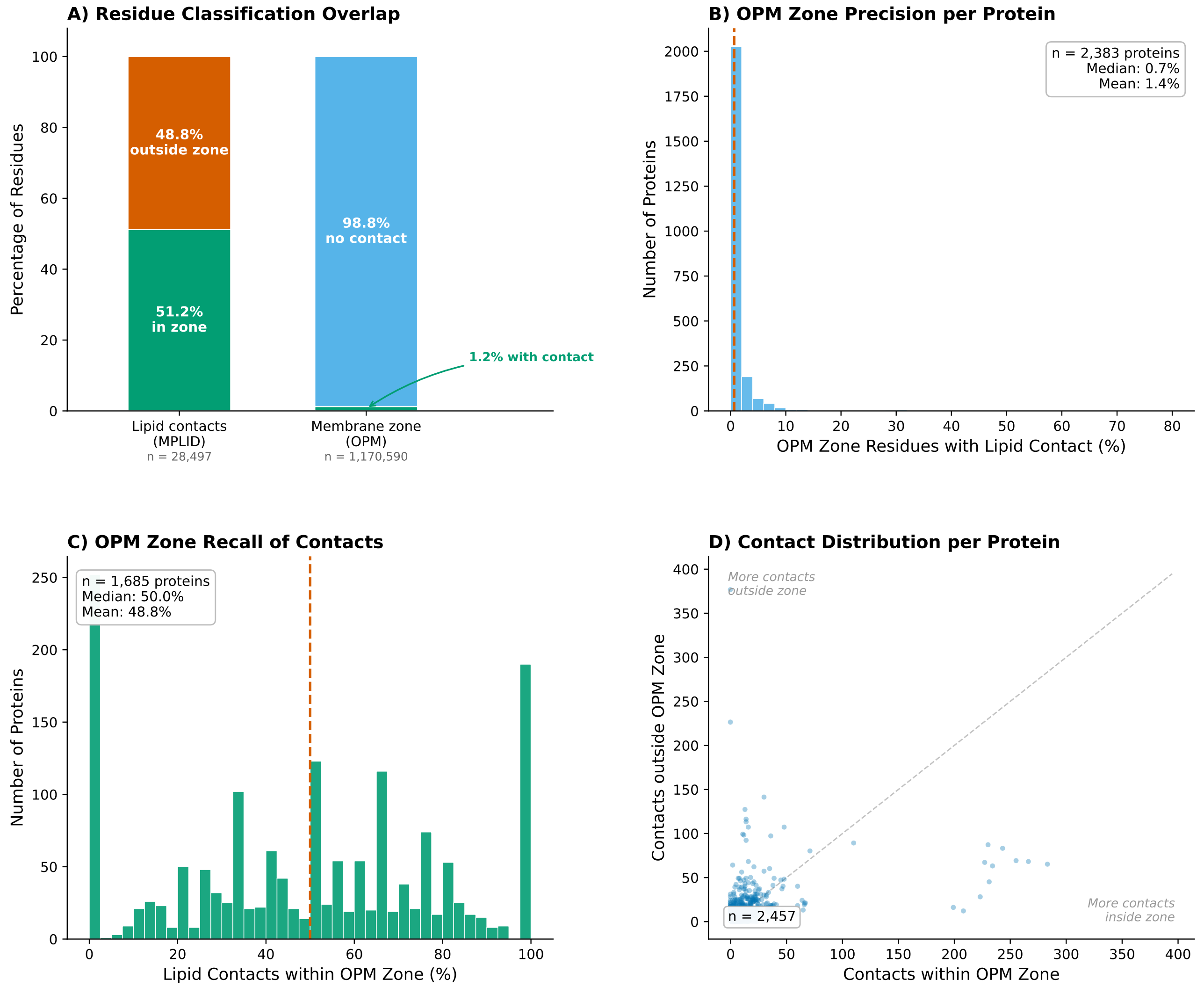

Fig. 8. Quantitative comparison of MPLID lipid contacts versus OPM membrane zone annotations. (A) Residue classification overlap: 48.8% of lipid contacts (n = 28,497) fall outside the OPM membrane zone, while 98.8% of OPM zone residues (n = 1,170,590) lack direct lipid contact. (B) Per-protein OPM precision (n = 2,383 proteins): median 0.7% of membrane zone residues have lipid contacts. (C) Per-protein OPM recall (n = 1,685 proteins): the membrane zone captures a median of 50.0% of lipid contacts. (D) Per-protein scatter of contacts inside versus outside the OPM zone (n = 2,457 proteins). These results demonstrate that membrane zone classification and lipid contact annotation address fundamentally different biological questions.

MPLID construction pipeline; OPM is discussed in this paper solely to contextualize the complementary biological questions of membrane zone positioning versus direct lipid contact annotation. All residue annotations in MPLID derive exclusively from experimentally resolved lipid molecules in PDB structures. The RCSB Protein Data Bank Berman et al. [2000], Burley et al. [2023] was queried programmatically for all structures containing recognized lipid chemical components, identifying 8,221 candidate structures with lipid ligands. This query-first strategy ensures that every protein in the dataset has at least one experimentally resolved lipid molecule, providing an unbiased sampling of protein-lipid interactions across all structural classes. The identified structures were downloaded as original PDB coordinate files (9,796 files total, as some entries required retrieval of biological assembly files or alternate depositions). After parsing, 5,723 unique proteins yielded valid structures with recognized HETATM lipid records; the remainder comprised duplicate chains, structures that failed parsing, or entries lacking standard amino acid residues. These were processed through the contact calculation and quality filtering pipeline described in the following subsections.

Lipid molecule identification

Lipid molecules in PDB structures were identified using their Chemical Component Dictionary codes. We compiled a list of 117 lipid codes organized into ten functional categories: phospholipids (CDL, POV, PCW, PEE, PGV, and others), cardiolipin (CDL, C9V, 18W, LCL), sphingolipids (SPH, S1P, HXJ, CRT), sterols (CLR, CHD, Y01, ERG), fatty acids (PLM, MYR, OLA, STE, ARA, DHA), glycerolipids (TGL, DAG, MAG), detergent mimetics (LDA, LMT, BOG, OLC, DPC), lipid A components (LPA, KDO, 6LP), and CHARMM simulation nomenclature (POPC, POPE, POPG, and others commonly found in cryo-EM depositions). This list was developed iteratively by cross-referencing the PDB Chemical

Component Dictionary with lipid classification databases and manually curating entries to exclude non-lipid small molecules that might share similar chemical codes. Detergent mimetics were included because they frequently occupy native lipid binding sites in membrane protein crystal structures and provide relevant information about lipid-protein interaction geometry Lee [2004].

Structure processing and contact calculation

For each candidate protein, the original PDB coordinate file was downloaded from RCSB to preserve HETATM records containing lipid molecule coordinates. Structure parsing was performed using BioPython Cock et al. [2009], extracting all standard amino acid residues (ATOM records) and all recognized lipid molecules (HETATM records). Non-standard residues, water molecules, metal ions, and non-lipid ligands were excluded.

Lipid contacts were defined using an all-atom distance criterion. For each protein residue R and each lipid molecule L in the structure, the minimum distance $d_{\min}(R, L)$ between any heavy (non-hydrogen) atom of R and any heavy atom of L was calculated. A residue was labeled as a lipid contact if:

$$d_{\min}(R, L) = \min_{a \in R,\, b \in L} \|\mathbf{r}_a - \mathbf{r}_b\| \leq 4.0 \text{ Å} \tag{1}$$

where $\mathbf{r}_a$ denotes the Cartesian coordinates of heavy atom a in residue R and $\mathbf{r}_b$ denotes the coordinates of heavy atom b in lipid L. The use of all heavy atoms (backbone and side chain) as residue representatives captures direct van der Waals contacts that would be missed by a Cα-only criterion, particularly for residues whose side chains extend into lipid-binding pockets while their backbone remains distant from lipid molecules. The 4.0 Å cutoff was chosen to capture van der Waals contacts, consistent with standard distance thresholds in protein-ligand interaction analysis. For each residue, the minimum all-atom distance to the nearest lipid heavy atom was recorded regardless of contact status, providing a continuous distance metric that enables researchers to evaluate alternative cutoff values.

Quality filtering

Of the 5,723 proteins with parseable lipid-containing structures, 1,019 were removed because they contained only non-lipid molecules misannotated as lipids (such as HEPES buffer and bacteriochlorophyll) or had all lipid atoms beyond the 4.0 Å contact threshold, leaving 4,704 proteins with at least one genuine lipid contact. This filtering ensures that every protein in the final dataset has genuine, experimentally resolved lipid contacts.

Sequence clustering

To prevent data leakage between training, validation, and test splits, all 4,704 proteins were clustered using MMseqs2 Steinegger and Söding [2017] with a 30% sequence identity threshold and 80% bidirectional alignment coverage. These stringent thresholds ensure that proteins assigned to different splits are sufficiently divergent in sequence to represent independent test cases. The clustering produced 813 clusters.

Split strategy

Clusters were assigned to training, validation, and test splits using stratified random sampling at the cluster level, with stratification based on the average contact rate per cluster. This approach ensures that each split contains a representative distribution of high-contact and low-contact proteins. Entire clusters were assigned to a single split, guaranteeing that no two proteins sharing more than 30% sequence identity appear in different partitions. The resulting splits contain 2,578 training proteins (54.8%), 1,051 validation proteins (22.3%), and 1,075 test proteins (22.9%).

Software and computational environment

The MPLID construction pipeline was implemented in Python 3.11.8 using BioPython 1.83 Cock et al. [2009] for PDB structure parsing, pandas 2.2.2 McKinney [2010] for data manipulation, NumPy 1.26.4 for numerical computation, SciPy 1.13 for distance calculations, and scikit-learn 1.7.2 Pedregosa et al. [2011] for data splitting. MMseqs2 version 15 Steinegger and Söding [2017] was used for sequence clustering. The RCSB PDB search API was used for programmatic identification of structures containing lipid chemical components.

Discussion

MPLID represents the first large-scale, publicly deposited dataset of experimentally validated residue-level lipid contacts for membrane proteins. The dataset addresses a biological question that is fundamentally distinct from membrane zone classification: rather than identifying residues located within a computationally defined hydrophobic slab, MPLID annotates residues that make direct physical contact with crystallized lipid molecules at atomic resolution. The analyses presented here validate this annotation approach through multiple independent lines of evidence. The amino acid enrichment patterns at lipid contact sites are biologically consistent, with aromatic anchoring residues (tryptophan, phenylalanine) and positively charged residues (arginine, lysine) enriched at contact sites, while conformationally constrained (proline) and negatively charged (aspartate) residues are depleted. The distance distribution analysis confirms that the 4.0 Å cutoff effectively captures the van der Waals contact boundary. The quantitative comparison with OPM membrane zone annotations demonstrates that nearly half of experimentally observed lipid contacts fall outside computationally predicted membrane boundaries, reinforcing the complementary nature of these two resources.

The scale of MPLID, comprising 4,704 proteins across 813 sequence clusters, provides substantially greater coverage than prior experimental lipid contact datasets. The DREAMM training set Chatzigoulas and Cournia [2022b], the largest previously available collection, contained 54 peripheral membrane proteins. MPLID expands this by nearly two orders of magnitude while covering all membrane protein classes, including integral, peripheral, and single-pass proteins. The cluster-aware splitting strategy, using a conservative 30% sequence identity threshold, ensures that evaluation on the test set reflects genuine generalization rather than sequence memorization.

Limitations

Several limitations should be noted when using MPLID. First, lipid contact labels depend on the crystallographic or cryo-EM resolution and completeness of resolved lipid molecules; many native lipid binding events are not captured because lipids are disordered or stripped during purification and crystallization. Consequently, the false negative rate is likely

substantial, and the true positive rate of lipid contacts in membrane proteins is probably higher than the 1.00% observed in MPLID. Moreover, many structures contain only one or a few resolved lipid molecules, meaning that the observed contacts represent a sparse snapshot of the full lipid-binding landscape rather than a comprehensive map of all possible interaction sites. Second, the inclusion of detergent mimetics as proxy lipids introduces potential noise, as detergent binding sites may not perfectly recapitulate native lipid interactions. Analysis of HETATM records across all processed PDB files shows that approximately 17% of proteins with recognized lipid molecules contain exclusively detergent mimetics (e.g., dodecyl maltoside, octyl glucoside, lauryldimethylamine oxide) without any physiological lipid species, while an additional 10% contain both detergent and physiological lipids. This means that for roughly one in six proteins, all contact labels derive from crystallization detergents rather than native lipid species. Third, the dataset inherits biases from the PDB toward well-studied, readily crystallizable membrane protein families, leaving many therapeutically relevant but structurally uncharacterized families underrepresented. Finally, the 4.0 Å distance cutoff is a single threshold that may not be optimal for all lipid types; researchers are encouraged to explore alternative cutoffs using the continuous distance values provided in the dataset. Additionally, because MPLID annotations are derived from static crystallographic and cryo-EM snapshots, they may not fully represent the dynamic nature of protein–lipid interactions in native membrane environments, where lipids undergo lateral diffusion and exchange on nanosecond-to-microsecond timescales. Crystal packing artifacts may also contribute false positive contacts, as lipid molecules resolved in crystal structures can occupy lattice contact interfaces that do not reflect native membrane binding.

No structure resolution cutoff was applied during dataset construction: all PDB structures with recognized lipid molecules were included regardless of crystallographic resolution. While this maximizes dataset size, low-resolution structures (worse than 3.5 Å) may have less reliable lipid positions, potentially introducing noise into contact labels. Researchers working with resolution-sensitive applications are encouraged to filter by PDB resolution using the provided `pdb_id` field.

The current release does not include per-residue lipid chemical component codes, meaning that users cannot directly determine which lipid type (e.g., cholesterol versus cardiolipin) contacts a given residue. However, the continuous `min_distance` values enable evaluation of alternative distance cutoffs, and the `pdb_id` field allows users to retrieve lipid identities from the original PDB structures. Lipid type stratification at the residue level is planned for a future release.

Four selenocysteine (SEC) residues in the dataset are all annotated as lipid contacts (4/4, 100% contact rate). Given the extremely small sample size, this observation is not statistically meaningful and these residues were excluded from enrichment ratio analysis.

Cross-validation of MPLID labels against the curated dataset used by the DREAMM prediction method Chatzigoulas and Cournia [2022b] is planned as future work to assess concordance between the two independently derived annotation approaches.

Conclusions

In summary, MPLID provides a large-scale, experimentally grounded resource that fills a critical gap in the membrane protein structural biology landscape. By distinguishing direct lipid contact from membrane zone localization, the dataset enables a new class of residue-level prediction tasks with direct relevance to drug discovery, protein engineering, and mechanistic understanding of lipid-mediated regulation. The dataset adheres to FAIR principles and is freely available under a CC0 public domain dedication. MPLID is intended as a ground-truth resource for residue-level lipid contact annotation and does not aim, by itself, to provide an optimized predictive solution to the full experimental-only classification problem. As the PDB continues to grow and cryo-EM structures increasingly resolve lipid molecules at higher resolution, future releases of MPLID will expand both the coverage and the annotation granularity of this resource.

Potential implications

MPLID is designed to serve as a large-scale training and evaluation resource for multiple research applications in structural bioinformatics, drug discovery, and membrane biology.

Machine learning for lipid contact prediction

The principal application of MPLID is as a training and evaluation dataset for developing machine learning models that predict lipid contact residues from protein sequence or structure. The pre-defined, cluster-aware train/validation/test splits enable standardized model comparison, and the class imbalance (1.00% positive rate, approximately 100:1 negative-to-positive ratio) presents a realistic and challenging binary classification task that requires specialized approaches such as class weighting, oversampling (SMOTE) Chawla et al. [2002], or focal loss functions. The scale of the dataset is sufficient for training deep neural networks, including architectures that combine structural descriptors with protein language model embeddings from ESM-2 Lin et al. [2023] or ProtTrans Elnaggar et al. [2022]. We note that the Matthews correlation coefficient should be used as the primary evaluation metric for this task due to the substantial class imbalance, as accuracy and even F1 scores can be misleading when the negative class dominates.

Drug discovery applications

Lipid binding sites on membrane proteins represent an emerging class of druggable targets. General anesthetics, neurosteroids, and endocannabinoids modulate ion channels and GPCRs through specific lipid binding sites Duncan et al. [2020], Brannigan et al. [2008], and cholesterol binding sites on GPCRs are increasingly recognized as allosteric modulatory sites Hanson et al. [2008], Fantini and Barrantes [2013]. MPLID can inform the identification of lipid-mimetic drug binding sites by providing a catalog of experimentally validated lipid interaction residues across thousands of membrane proteins. Predicted lipid contact sites on therapeutically relevant proteins can guide the design of lipid-conjugated prodrugs, membrane-anchored therapeutics, or allosteric modulators that exploit lipid binding pockets.

Structural biology and protein engineering

Understanding which residues contact lipids is essential for rational membrane protein engineering. Mutations at lipid contact sites can destabilize membrane proteins, alter their trafficking, or modify their functional properties. MPLID

provides data to train models that predict which residues are critical for lipid-mediated stability, enabling more informed design of stabilizing mutations for membrane protein crystallization, cryo-EM, or functional studies. Additionally, the dataset supports comparative analysis of lipid contact patterns across protein families, enabling identification of conserved lipid binding motifs and family-specific interaction signatures. Future work incorporating cavity-based structural analysis could further support the observation that many residues located within the membrane region do not form direct lipid contacts, reinforcing the conceptual separation between membrane localization and residue-level interaction. These interpretations are based on static structural snapshots. Incorporation of dynamic information may alter residue–lipid proximity patterns, as conformational fluctuations could transiently modify contact definitions.

Integration with protein language models

The residue-level format of MPLID is directly compatible with per-residue embeddings from protein language models. Each residue's 1,280-dimensional ESM-2 embedding Lin et al. [2023] or 1,024-dimensional ProtTrans embedding Elnaggar et al. [2022] can be concatenated with structural descriptors and used as input to supervised classifiers. The 813 sequence clusters provide sufficient diversity to evaluate whether language model representations generalize across membrane protein families with limited sequence similarity. Alternatively, MPLID can be combined with interpretable structural descriptor frameworks, such as STING RDB-derived nanoenvironment descriptors Neshich et al. [2003, 2006]. In this setting, residue-level lipid contacts may be analyzed in terms of explicit physicochemical and geometric properties, enabling mechanistic interpretation of the nanoenvironment characteristics associated with lipid interaction. This dual compatibility supports both high-capacity representation learning approaches and descriptor-based, interpretable modeling strategies.

Availability of supporting source code and data

The complete MPLID dataset is deposited at Zenodo under a CC0 public domain dedication with persistent DOI: `https://doi.org/10.5281/zenodo.18487584` Omage and Neshich [2026]. Upon acceptance, the dataset will also be deposited in GigaDB in accordance with journal policy. The Zenodo archive contains the full residue-level annotations (train, validation, and test splits as gzip-compressed CSV files), protein-level metadata, amino acid composition statistics, and a data dictionary describing all fields.

All code for reproducing the dataset from raw PDB structures is available in a public GitHub repository: `https://github.com/omagebright/MPLID`. The repository includes the complete processing pipeline (structure downloading, lipid identification, contact calculation, quality filtering, and split generation), analysis scripts for generating figures and tables, and documentation of the data schema and methodology.

The dataset and code are organized following FAIR principles Wilkinson et al. [2016]: data are Findable via persistent DOIs and indexed metadata; Accessible through standard HTTPS download from Zenodo and GitHub; Interoperable using standard CSV format with documented column definitions; and Reusable under permissive open licenses (CC0 for data, MIT for code).

Conflicts of interest

The authors declare that they have no competing interests.

Funding

This research was funded by the São Paulo Research Foundation (FAPESP) (grant nos. 2023/02691-2, 2025/23708-6). The Article Processing Charge for the publication of this research was funded by the Coordenação de Aperfeiçoamento de Pessoal de Nível Superior (CAPES) (ROR identifier: 00x0ma614). For the purpose of open access, the authors have applied a Creative Commons Attribution (CC BY) license to any Author Accepted Manuscript version arising.

Data availability

The data underlying this article are available in Zenodo at `https://doi.org/10.5281/zenodo.18487584`, and in a public GitHub repository at `https://github.com/omagebright/MPLID`.

Author contributions statement

Author contributions follow the CRediT taxonomy. F.B.O.: Conceptualization, Methodology, Software, Formal Analysis, Data Curation, Writing (Original Draft), Visualization. G.N.: Conceptualization (dataset definition and biological framework), Supervision, Writing (Original Draft, Review & Editing), Funding Acquisition. Both authors read and approved the final manuscript.

Ethics approval and consent to participate

Not applicable. This study uses publicly available structural data from the Protein Data Bank and does not involve human subjects, animal experiments, or clinical data.

Acknowledgments

The authors thank Ivan Mazoni and Inácio Henrique Yano for their contributions to data validation and resource management during the early stages of this work. The authors thank Embrapa Digital Agriculture (Embrapa Agricultura Digital) for computational resources and infrastructure support. The authors acknowledge the RCSB Protein Data Bank Berman et al. [2000], Burley et al. [2023] and the OPM database Lomize et al. [2012] teams for maintaining the essential structural biology resources upon which this work depends. The authors thank the developers of MMseqs2 Steinegger and Söding [2017], BioPython Cock et al. [2009], and the broader open-source scientific computing community for the software tools used in this study.

Abbreviations

MPLID: Membrane Protein-Lipid Interaction Database; **PDB**: Protein Data Bank; **OPM**: Orientations of Proteins in Membranes; **GPCR**: G protein-coupled receptor; **MCC**: Matthews correlation coefficient; **CG-MD**: Coarse-grained molecular dynamics; **FAIR**: Findable, Accessible, Interoperable, Reusable; **CDL**: Cardiolipin; **CLR**: Cholesterol; **PLM**: Palmitic acid; **DREAMM**: Detection of Residues on Exposed Areas of Membrane-associated Macromolecules; **ESM-2**:

Evolutionary Scale Modeling 2; **SMOTE**: Synthetic Minority Over-sampling Technique; **RCSB**: Research Collaboratory for Structural Bioinformatics; **CSV**: Comma-separated values.

References

Erik Wallin and Gunnar von Heijne. Genome-wide analysis of integral membrane proteins from eubacterial, archaean, and eukaryotic organisms. *Protein Science*, 7(4):1029–1038, 1998. doi: 10.1002/pro.5560070420.

John P Overington, Bissan Al-Lazikani, and Andrew L Hopkins. How many drug targets are there? *Nature Reviews Drug Discovery*, 5(12):993–996, 2006. doi: 10.1038/nrd2199.

Rita Santos, Oleg Ursu, Anna Gaulton, et al. A comprehensive map of molecular drug targets. *Nature Reviews Drug Discovery*, 16(1):19–34, 2017. doi: 10.1038/nrd.2016.230.

Valentina Corradi, Besian I Sejdiu, Haydee Mesa-Galloso, et al. Emerging diversity in lipid–protein interactions. *Chemical Reviews*, 119(9):5775–5848, 2019. doi: 10.1021/acs.chemrev.8b00451.

Arthur Laganowsky, Eamonn Reading, Timothy M Allison, et al. Membrane proteins bind lipids selectively to modulate their structure and function. *Nature*, 510(7503):172–175, 2014. doi: 10.1038/nature13419.

Anna L Duncan, Wanling Song, and Mark SP Sansom. Lipid-dependent regulation of ion channels and G protein-coupled receptors: insights from structures and simulations. *Annual Review of Pharmacology and Toxicology*, 60:31–50, 2020. doi: 10.1146/annurev-pharmtox-010919-023411.

Anthony G Lee. How lipids affect the activities of integral membrane proteins. *Biochimica et Biophysica Acta - Biomembranes*, 1666(1-2):62–87, 2004. doi: 10.1016/j.bbamem.2004.05.012.

Michael A Hanson, Vadim Cherezov, Mark T Griffith, et al. A specific cholesterol binding site is established by the 2.8 å structure of the human β2-adrenergic receptor. *Structure*, 16 (6):897–905, 2008. doi: 10.1016/j.str.2008.05.001.

Felix-Xavier Contreras, Andreas M Ernst, Felix Wieland, and Britta Brügger. Specificity of intramembrane protein–lipid interactions. *Cold Spring Harbor Perspectives in Biology*, 3 (6):a004705, 2011. doi: 10.1101/cshperspect.a004705.

Takeshi Harayama and Howard Riezman. Understanding the diversity of membrane lipid composition. *Nature Reviews Molecular Cell Biology*, 19(5):281–296, 2018. doi: 10.1038/nrm.2017.138.

Gerrit van Meer, Dennis R Voelker, and Gerald W Feigenson. Membrane lipids: where they are and how they behave. *Nature Reviews Molecular Cell Biology*, 9(2):112–124, 2008. doi: 10.1038/nrm2330.

Mikhail A Lomize, Irina D Pogozheva, Hyeon Joo, Henry I Mosberg, and Andrei L Lomize. OPM database and PPM web server: resources for positioning of proteins in membranes. *Nucleic Acids Research*, 40(D1):D370–D376, 2012. doi: 10.1093/nar/gkr703.

Andrei L Lomize, Irina D Pogozheva, Mikhail A Lomize, and Henry I Mosberg. Positioning of proteins in membranes: a computational approach. *Protein Science*, 15(6):1318–1333, 2006. doi: 10.1110/ps.062126106.

Alexios Chatzigoulas and Zoe Cournia. DREAMM: a web-based server for drugging protein-membrane interfaces as a novel workflow for targeted drug design. *Bioinformatics*, 38(24): 5449–5451, 2022a. doi: 10.1093/bioinformatics/btac680.

Thomas D Newport, Mark SP Sansom, and Phillip J Stansfeld. The MemProtMD database: a resource for membrane-embedded protein structures and their lipid interactions. *Nucleic Acids Research*, 47(D1):D390–D397, 2019. doi: 10.1093/nar/gky1047.

Phillip J Stansfeld, Joseph E Goose, Martin Caffrey, et al. MemProtMD: automated insertion of membrane protein structures into explicit lipid membranes. *Structure*, 23(7): 1350–1361, 2015. doi: 10.1016/j.str.2015.05.006.

Goran Neshich, Roberto C Togawa, Adauto L Mancini, Paula R Kuser, Michel EB Yamagishi, Georgios Pappas Jr, Wandré V Torres, Thalita Fonseca e Campo, Leandro L Ferreira, Fabio M Luna, et al. STING Millennium: a web-based suite of programs for comprehensive and simultaneous analysis of protein structure and sequence. *Nucleic Acids Research*, 31 (13):3386–3392, 2003. doi: 10.1093/nar/gkg578.

Goran Neshich, Ivan Mazoni, Sandro Roberto Oliveira, Michel EB Yamagishi, Paula R Kuser-Falcão, Leandro C Borro, Dag H Morita, Katlin Rabelo Souza, Vagner M Gonçalves-Almeida, et al. STING Report: convenient web-based application for graphic and tabular presentations of protein sequence, structure and function descriptors from the STING database. *Nucleic Acids Research*, 34(suppl_2): W209–W213, 2006. doi: 10.1093/nar/gkl226.

Alexios Chatzigoulas and Zoe Cournia. Predicting protein–membrane interfaces of peripheral membrane proteins using ensemble machine learning. *Briefings in Bioinformatics*, 23 (2):bbab518, 2022b. doi: 10.1093/bib/bbab518.

Niek van Hilten, Nino Verwei, Jeroen Methorst, Carsten Nase, Andrius Bernatavicius, and Herre Jelger Risselada. PMIpred: a physics-informed web server for quantitative protein–membrane interaction prediction. *Bioinformatics*, 40(2):btae069, 2024. doi: 10.1093/bioinformatics/btae069.

Dimitra Paranou, Alexios Chatzigoulas, and Zoe Cournia. Using deep learning and large protein language models to predict protein–membrane interfaces of peripheral membrane proteins. *Bioinformatics Advances*, 4(1):vbae078, 2024. doi: 10.1093/bioadv/vbae078.

Ahmed Elnaggar, Michael Heinzinger, Christian Dallago, et al. ProtTrans: toward understanding the language of life through self-supervised learning. *IEEE Transactions on Pattern Analysis and Machine Intelligence*, 44(10):7112–7127, 2022. doi: 10.1109/TPAMI.2021.3095381.

Helen M Berman, John Westbrook, Zukang Feng, et al. The Protein Data Bank. *Nucleic Acids Research*, 28(1):235–242, 2000. doi: 10.1093/nar/28.1.235.

Stephen K Burley, Charmi Bhikadiya, Chunxiao Bi, et al. RCSB Protein Data Bank (rcsb.org): delivery of experimentally-determined PDB structures alongside one million computed structure models of proteins from artificial intelligence/machine learning. *Nucleic Acids Research*, 51 (D1):D488–D508, 2023. doi: 10.1093/nar/gkac1077.

Zeming Lin, Halil Akin, Roshan Rao, et al. Evolutionary-scale prediction of atomic-level protein structure with a language model. *Science*, 379(6637):1123–1130, 2023. doi: 10.1126/science.ade2574.

Martin Steinegger and Johannes Söding. MMseqs2 enables sensitive protein sequence searching for the analysis of massive data sets. *Nature Biotechnology*, 35(11):1026–1028, 2017. doi: 10.1038/nbt.3988.

Ian Sillitoe, Nicola Bordin, Natalie Dawson, et al. CATH: increased structural coverage of functional space. *Nucleic Acids Research*, 49(D1):D266–D273, 2021. doi: 10.1093/nar/gkaa1079.

Gunnar von Heijne. Membrane protein structure prediction: hydrophobicity analysis and the positive-inside rule. *Journal of Molecular Biology*, 225(2):487–494, 1992. doi: 10.1016/0022-2836(92)90934-C.

William P Russ and Donald M Engelman. The GxxxG motif: a framework for transmembrane helix-helix association. *Journal of Molecular Biology*, 296(3):911–919, 2000. doi: 10.1006/jmbi.1999.3489.

Peter JA Cock, Tiago Antao, Jeffrey T Chang, et al. Biopython: freely available Python tools for computational molecular biology and bioinformatics. *Bioinformatics*, 25 (11):1422–1423, 2009. doi: 10.1093/bioinformatics/btp163.

Wes McKinney. Data structures for statistical computing in Python. In *Proceedings of the 9th Python in Science Conference*, pages 56–61, 2010. doi: 10.25080/Majora-92bf1922-00a.

Fabian Pedregosa, Gaël Varoquaux, Alexandre Gramfort, et al. Scikit-learn: machine learning in Python. *Journal of Machine Learning Research*, 12:2825–2830, 2011.

Nitesh V Chawla, Kevin W Bowyer, Lawrence O Hall, and W Philip Kegelmeyer. SMOTE: synthetic minority oversampling technique. *Journal of Artificial Intelligence Research*, 16:321–357, 2002. doi: 10.1613/jair.953.

Grace Brannigan, Jérôme Hénin, Richard Law, et al. Embedded cholesterol in the nicotinic acetylcholine receptor. *Proceedings of the National Academy of Sciences*, 105(38): 14418–14423, 2008. doi: 10.1073/pnas.0803029105.

Jacques Fantini and Francisco J Barrantes. How cholesterol interacts with membrane proteins: an exploration of cholesterol-binding sites including CRAC, CARC, and tilted domains. *Frontiers in Physiology*, 4:31, 2013. doi: 10.3389/fphys.2013.00031.

Folorunsho Bright Omage and Goran Neshich. MPLID: Membrane protein-lipid interface dataset, 2026. URL `https://doi.org/10.5281/zenodo.18487584`. Version 2.0.0.

Mark D Wilkinson, Michel Dumontier, IJsbrand Jan Aalbersberg, et al. The FAIR guiding principles for scientific data management and stewardship. *Scientific Data*, 3(1): 160018, 2016. doi: 10.1038/sdata.2016.18.
