# Supplementary material for: MPLID (Membrane Protein–Lipid Interaction Database): A Large-Scale Experimental Resource of Residue-Level Protein–Lipid Contacts
Source: Gigascience. 2026 Jul 27;15:giag080. doi: 10.1093/gigascience/giag080 (PMC13435803; doi:10.1093/gigascience/giag080)
Supplement: Supplementary file 3 [file giag080_giga-d-26-00059_revision_1.pdf]

GigaScience

MPLID (Membrane Protein-Lipid Interaction Database): A Large-Scale Experimental Resource of Residue-Level Protein-Lipid Contacts

--Manuscript Draft--

**Manuscript Number:**	GIGA-D-26-00059R1
**Full Title:**	MPLID (Membrane Protein-Lipid Interaction Database): A Large-Scale Experimental Resource of Residue-Level Protein-Lipid Contacts
**Article Type:**	Data Note
**Funding Information:**	Fundação de Amparo à Pesquisa do Estado de São Paulo (2020/08615-8, 2023/02691-2, 2025/23708-6) — Dr Folorunsho Bright Omage
**Abstract:**	Background: Membrane proteins constitute approximately 20-30% of all proteomes and represent over 60% of current drug targets. Although protein-lipid interactions play important structural and regulatory roles in membrane-associated proteins, most existing structural resources focus on identifying whether a residue lies within a membrane region, typically inferred from computational hydrophobicity-based positioning algorithms. This approach does not directly address a distinct biological question: which residues at the protein surface make direct physical contact with lipid molecules? Answering this question from experimental data is critical for understanding lipid-mediated allostery, designing lipid-mimetic therapeutics, and training accurate machine learning models for lipid binding site prediction.<br>Findings: We present MPLID (Membrane Protein-Lipid Interaction Database), a curated residue-level dataset comprising 4,704 membrane proteins representing 813 sequence clusters at 30% identity, 8,055,325 residues, and 80,439 experimentally validated lipid contact annotations (1.00% observed positive rate). Labels are derived exclusively from crystallized lipid molecules resolved in Protein Data Bank structures using a 4.0 Å all-atom heavy-atom distance cutoff. Because most native lipid interactions are lost during purification and crystallization, this observed rate represents a lower bound, and the non-contact class inevitably contains false negatives. The dataset recognizes 117 lipid codes across ten functional categories (cross-validated against the RCSB Chemical Component Dictionary), spanning phospholipids, cardiolipin, sphingolipids, sterols, fatty acids, glycerolipids, detergent mimetics (explicitly flagged), lipid A components, and CHARMM simulation nomenclature. To prevent data leakage, proteins are clustered at 30% sequence identity using MMseqs2, yielding 813 clusters partitioned into training (2,578), validation (1,051), and test (1,075) splits. Amino acid composition analysis reveals biologically consistent enrichment at lipid contact sites: tryptophan (1.88×), arginine (1.44×), glycine (1.36×), lysine (1.33×), and phenylalanine (1.23×) are enriched, while proline (0.51×), isoleucine (0.57×), and aspartate (0.59×) are depleted.<br>Conclusions: MPLID addresses a distinct biological question compared to existing resources (OPM, MemBlob, BioDolphin/PLIP): identifying residues that directly contact experimentally resolved lipid molecules rather than those positioned within computationally defined membrane boundaries. With 4,704 proteins and over 8 million annotated residues, MPLID provides the scale needed for training deep learning models for lipid contact prediction, with direct applications in structure-guided drug design and membrane protein engineering. The dataset adheres to FAIR principles and is freely available under a CC0 public domain dedication. Structurally resolved contacts represent only a subset of the full biological lipid interaction landscape, and MPLID is intended as a ground-truth resource rather than a complete catalog of lipid binding sites.
**Corresponding Author:**	Folorunsho Bright Omage, Ph.D.<br>University of Oxford<br>Oxford, UNITED KINGDOM
**Corresponding Author Secondary Information:**	
**Corresponding Author's Institution:**	University of Oxford

Powered by Editorial Manager® and ProduXion Manager® from Aries Systems Corporation

Corresponding Author's Secondary Institution:	
First Author:	Folorunsho Bright Omage, Ph.D.
First Author Secondary Information:	
Order of Authors:	Folorunsho Bright Omage, Ph.D.
	Goran Neshich, Ph.D.
Order of Authors Secondary Information:	
Response to Reviewers:	We thank the editor and reviewers for their careful evaluation of the manuscript. We have revised the text, data description, limitations, and resource-registration statements in response to the comments below.<br><br>Response to Reviewer #1<br>Reviewer comment:<br>The MPLID project aims to identify protein-lipid interactions at a residue-by-residue level. They build a database from the entire PDB and thus examines a wide range of lipids and proteins, producing a huge amount of data (8 million residues identified). I think this resource is potentially of considerable value for the training of models for downstream analysis.<br>The study appears well carried out and the paper well written. That said, I have a few issues with the framing of the study, which I think need to be addressed before publication.<br>Response: We thank the reviewer for this positive assessment of MPLID and its potential value for downstream modelling.<br><br>Reviewer comment: It should be made much clearer in the paper that their approach simply looks at protein-lipid interactions as found in the PDB. This is a very specific and somewhat artificial environment – only certain protein-lipid interactions survive the process of purification (often using harsh detergents). Their much-repeated statement that 1% of all residues are lipid contacting is interesting, but this will of course not reflect biological reality. So upfront it should be made clear that this is for “structural” protein-lipid interactions only, which represent only a small amount of the total. Claims that their data “reflects the biological reality that only a small fraction of residues in membrane proteins make direct lipid contacts” should be dropped. The 1% residue claim should be strongly reconsidered or reframed, as their “non-contact” data will have huge amounts of false-negatives, likely exceeding the “contact” data by an order of magnitude.<br>Response: We agree with the reviewer and have reframed the manuscript accordingly. The previous statement implying that the 1.00% contact rate reflects biological reality has been removed.<br>The revised manuscript now describes this value as an observed lower bound based on structurally resolved lipid molecules in PDB entries. We also state that the non-contact class contains false negatives because many native lipid interactions are lost during purification, crystallization, or cryo-EM sample preparation.<br>The main changes are:<br>•The Abstract now states that the 1.00% rate is a lower bound and that the non-contact class contains false negatives.<br>•Section 1.1 and Table 1 now distinguish membrane-zone annotation from direct structural lipid-contact annotation.<br>•Section 2.1 explains that MPLID captures structurally resolved contacts and cannot be interpreted as a complete map of in vivo lipid binding.<br>•The Discussion and Conclusions now describe MPLID as a resource for structurally resolved contacts, not as a complete catalogue of biological protein-lipid interactions.<br>•The Limitations section includes a dedicated paragraph on false negatives in the non-contact class.<br>We thank the reviewer for this correction; it improved the interpretation of the dataset.<br><br>Reviewer comment:<br>The authors highlight the novelty of their approach, however in their discussion of other methods they omit to mention MemBlob (https://doi.org/10.1093/bioinformatics/btz539). This work takes cryo-EM maps from the PDB and computationally screen the data to identify the membrane and solvent facing regions. They provide the data online, which

includes a residue-level description of whether residues face solvent, lipid, lipid interface, or are buried. This tool/repo somewhat overlaps with MPLID and should be cited and described appropriately.
Similarly, BioDolphin (https://doi.org/10.1038/s42004-024-01384-z) which identifies and labels all protein-lipid interactions from the PDB, as defined with PLIP https://plip-tool.biotec.tu-dresden.de/plip-web/plip/index. It provides full information on interactions at a residue level, and is accessible using an API, so overlaps considerably with the MPLID work. It should be cited and described appropriately.
Response: We thank the reviewer for pointing out these omissions. MemBlob and BioDolphin/PLIP are now cited and discussed in the revised manuscript.
Section 1.2 now describes MemBlob as a cryo-EM density-based membrane environment classifier and BioDolphin/PLIP as an interaction-profiling resource for protein-lipid contacts in PDB structures. We also added both resources to Table 2 and revised the comparison section to clarify how they differ from MPLID. In brief, OPM addresses membrane positioning, MemBlob addresses membrane environment inference, BioDolphin/PLIP provides interaction profiling, and MPLID provides pre-computed, ML-ready residue-level contact labels with cluster-aware splits.
The Abstract, Conclusions, and Limitations were also updated to position MPLID as complementary to these resources rather than replacing them.

Reviewer comment: Due to the focus on structural lipids only, there will be a strong favouring for anionic PIP and CDL lipids (hence the increased abundance of Arg and Lys in the data set). Most lipids in biological membranes are not anionic however – this should be mentioned. They also see enrichment of certain polar (Ser/His) and aromatic (Trp/Phe/Tyr). This matches previous analyses on CDL binding (10.1126/sciadv.abh2217), perhaps again reflecting the abundance of anionic lipids in the dataset. This should be discussed.
Response: We agree. The revised amino acid enrichment discussion now explicitly notes that the observed enrichment of arginine and lysine may partly reflect the overrepresentation of anionic lipids, especially cardiolipin and phosphatidylglycerol, among structurally resolved lipid species.
We also cite the cardiolipin binding-site study highlighted by the reviewer and added a Limitations paragraph on PDB and lipid-composition bias. This makes clear that MPLID reflects lipids that are resolved in structures, not the full lipid composition of biological membranes.

Reviewer comment: In terms of the lipid chemical codes used for MPLID in the zenodo link, if these are the codes used for the RSCB PDB then these should be double checked. I looked at a few and got varied results. E.g., LNS appears to be 6-(methylcarbamoyl)pyridine-2-carboxylic acid, which is not a sterol. LNN appears to be (6-hydroxybiphenyl-3-yl)acetic acid, which is not a sterol. GLF is alpha-D-glucopyranosyl fluoride, which is not a sphingolipid.
Response: The reviewer is correct. We audited all 90 PDB Chemical Component Dictionary codes against the RCSB API. The three examples raised by the reviewer are all misclassified in the original candidate list: LNS, LNN, and GLF are not sterol or sphingolipid entries, and none appears in the final MPLID dataset.
The audit identified 45 correctly classified codes, 34 incorrect or ambiguous assignments, and 11 codes absent from the dictionary. These results are now reported transparently in Supplementary Table S1. The manuscript now states that the codes were cross-validated against the RCSB Chemical Component Dictionary during revision.
We retained affected entries where they occur in the dataset because the contact labels are computed from atomic coordinates and do not depend on the functional category label. The revised manuscript now makes this distinction clear so users can exclude specific codes when strict lipid identity is required.

Reviewer comment: If I understand correctly, they've included detergent interactions in their dataset. I find this a very odd choice, and either suggest these are removed or that this is made much clearer up top.
Response: We understand the concern. Detergent mimetics are retained because they are part of the experimental structural context and often occupy membrane-associated surfaces, but their interpretation is now made explicit.
The revised manuscript now includes a protein-level has_detergent_only flag, reports the prevalence of detergent-only proteins, and adds a dedicated Limitations paragraph

Powered by Editorial Manager® and ProduXion Manager® from Aries Systems Corporation

on detergent mimetics. Users can therefore filter detergent-only entries before model training or downstream biological interpretation.

Reviewer comment: I'm curious about the protein with 4,820 lipid contacts. This PDB (6W4F) has 810 residues, so far fewer than 4,820. Further, the majority of the protein residues are not in contact with the lipids. Could it be that all 20 states in the PDB file have been accounted for by accident?
Response: The reviewer is correct. We checked PDB 6W4F and confirmed that it is an NMR structure of KRAS4B on a lipid bilayer nanodisc with 20 conformational models. The current pipeline counted contacts across all deposited NMR models, which explains the 4,820 contact annotations.
The revised manuscript now discloses this explicitly in the Dataset overview and in a dedicated Limitations paragraph. We also audited the dataset and found 41 NMR multi-model structures affected by this counting convention, contributing approximately 133,000 excess residue entries and 24,000 excess contact annotations. Future releases will use single-model extraction for NMR structures.

Response to Reviewer #2
Reviewer comment:
This manuscript by Omage and Neshich presents a new tool, MPLID, a large-scale, experimentally derived dataset of residue-level protein-lipid contacts extracted from PDB structures.
The manuscript addresses a conceptual gap between membrane zone annotations (e.g., OPM) and direct lipid-contact residues which is biologically important. The dataset is carefully curated and well documented. This makes the work potentially valuable for machine learning, membrane protein biology, and drug discovery. The manuscript is generally well motivated and appropriate as a Data Note, but there are important methodological and interpretational limitations that should be more clearly acknowledged and, where possible, addressed.
Response: We thank the reviewer for recognizing the conceptual contribution of MPLID and its potential value for machine learning, membrane protein biology, and drug discovery.

Reviewer comment: Lipid contacts resolved in PDB structures are not a random sample of biologically relevant lipid interactions. Certain lipids and proteins are overrepresented. While this is inevitable, the manuscript should include the discussion on the consequences of this such as the bias in training ML models (learning structural detectability rather than biological affinity).
Response: We agree. The Limitations section now includes a paragraph on PDB and lipid-composition bias. It explicitly states that structurally resolved lipid contacts are not a random sample of biological lipid interactions and that models trained on MPLID may learn structural detectability biases in addition to biological binding features. The machine-learning application section also notes that false negatives in the non-contact class may limit achievable performance.

Reviewer comment: If I understood correct, although the dataset includes detergent mimetics and CHARMM lipid codes, the biological interpretation of these contacts differs substantially from native lipid binding. This might create some problems. For example, detergent contacts may reflect solubilization artifacts, not functional lipid sites. This is a significant limitation for users and should be explicitly flagged.
Response: Addressed as above. The revised manuscript flags detergent mimetics explicitly, adds a detergent-only metadata field, and explains how users can filter detergent-only proteins when native lipid interpretation is required.

Reviewer comment: How did the authors determine the single distance cutoff to be 4.0 Å? Does this distance sufficiently distinguish headgroup vs acyl-chain contacts? Or takes orientation into account?
Response: The Methods section now explains the 4.0 Å cutoff. We chose this value to capture direct heavy-atom van der Waals contacts, consistent with common thresholds used in protein-ligand interaction analysis, contact databases, and previous protein-membrane interaction studies. The distance distribution in Figure 3 also supports this cutoff, with a clear peak around 3.5–4.0 Å.
We now state clearly that the cutoff does not distinguish lipid headgroup from acyl-

Powered by Editorial Manager® and ProduXion Manager® from Aries Systems Corporation

	chain contacts and does not encode orientation. Users interested in those distinctions can use the continuous distance values together with lipid atom identities from the original PDB coordinate files.<br><br>Reviewer comment: Does the removal of zero-contact proteins introduce selection bias? If that is the case, the dataset cannot support true proteome-wide negative controls and the manuscript should discuss that MPLID is not representative of the full membrane proteome.<br>Response: Yes. The revised manuscript now states that removing zero-contact proteins introduces selection bias. Section 2.1 explains that MPLID does not represent the complete membrane proteome and cannot be used as a source of proteome-wide negative controls. The same point is repeated in the Limitations section.<br><br>Response to Reviewer #3<br>Reviewer comment:<br>Lipids are known to interact with membrane proteins and regulate their structure and function; however, because the number of documented interaction examples is limited, the detailed mechanisms of these interactions remain insufficiently understood. The authors provide a comprehensive database of membrane protein-lipid interactions derived from PDB data. This effort is of extremely high importance for research in this field.<br>In any case, this manuscript contains important findings and is considered worthy of publication in GigaScience.<br>Response: We thank the reviewer for this positive assessment and for supporting publication in GigaScience.<br><br>Reviewer comment: Lipid species such as PA and PS are not included, and phosphoinositides (PIPs), which are known to bind to various membrane proteins, are also absent. In addition, polyunsaturated fatty acids have not been detected.<br>Response: We acknowledge this limitation. The revised Lipid type diversity section now states that PA, PS, phosphoinositides, and polyunsaturated fatty acids are absent or underrepresented in the current release. We explain that this reflects the difficulty of resolving these lipids in experimental structures and the current focus of MPLID on lipid species represented in PDB HETATM records. Future releases will expand coverage as more structures with these lipid classes become available.<br><br>Reviewer comment: Cholesterol hemisuccinate and cholesteryl hemisuccinate appear to be the same compound. Although these are cholesterol derivatives, they are in fact a type of detergent added artificially.<br>Response: We checked the PDB Chemical Component Dictionary entries and corrected the manuscript. CHD is cholic acid, not cholesteryl hemisuccinate. Y01 is cholesterol hemisuccinate, an artificial cholesterol derivative used during membrane protein solubilization.<br>The revised Limitations section now states that cholesterol hemisuccinate is not a native membrane lipid and can be filtered using the detergent-related metadata.
**Additional Information:**	
**Question**	**Response**
Are you submitting this manuscript to a special series or article collection?	No
**Experimental design and statistics**<br><br>Full details of the experimental design and statistical methods used should be given in the Methods section, as detailed in our [Minimum Standards Reporting Checklist](). Information essential to interpreting the data presented should be made available in the figure legends.	Yes

Have you included all the information requested in your manuscript?	
**Resources**<br><br>A description of all resources used, including antibodies, cell lines, animals and software tools, with enough information to allow them to be uniquely identified, should be included in the Methods section. Authors are strongly encouraged to cite Research Resource Identifiers (RRIDs) for antibodies, model organisms and tools, where possible.<br><br>Have you included the information requested as detailed in our Minimum Standards Reporting Checklist?	Yes
**Availability of data and materials**<br><br>All datasets and code on which the conclusions of the paper rely must be either included in your submission or deposited in publicly available repositories (where available and ethically appropriate), referencing such data using a unique identifier in the references and in the "Availability of Data and Materials" section of your manuscript.<br><br>Have you have met the above requirement as detailed in our Minimum Standards Reporting Checklist?	Yes
GigaScience has policies and guidelines in place for the use of generative AI-writing tools such as ChatGPT. If you have used such writing tools to assist with writing the manuscript this must be declared and cited in the text. Authors should not list AI-writing tools and other AI-assisted technologies as an author or co-author and should acknowledge that they are fully responsible for text generated or refined by AI-writing tools.<p>	No

A summary of use (particularly in the introduction or among methods) needs to be included at the end of the paper, and the outputs should also be included as a supplementary file hosted in GigaDB or other open repositories. Please read our guidelines for more information. <p><br><br>By submitting to GigaScience, you are aware of the journal's AI-writing tools policy, and if you have declared use of such tools below, you have acknowledged this where appropriate in your manuscript and have made a summary of use and outputs available. <p><br><b>AI-assisted writing tools have been used in the preparation of this manuscript?	

Main

Click here to access/download;Manuscript;main_gigascience.pdf

GigaScience, 2026, pp. 1–16

doi:

Data Note

DATA NOTE

MPLID (Membrane Protein–Lipid Interaction Database): A Large-Scale Experimental Resource of Residue-Level Protein–Lipid Contacts

Folorunsho Bright Omage[1,2,3,*] and Goran Neshich[2,*]

[1]Department of Computer Science, University of Oxford, Wolfson Building, Parks Road, OX1 3QD, Oxford, United Kingdom, [2]Computational Biology Research Group, Embrapa Digital Agriculture, Av. André Tosello 209, 13083-886, Campinas, São Paulo, Brazil and [3]Biological Chemistry Laboratory, Department of Organic Chemistry, Institute of Chemistry, University of Campinas (UNICAMP), R. Monteiro Lobato 270, 13083-970, Campinas, São Paulo, Brazil

*Corresponding authors. folorunsho.bright.omage@cs.ox.ac.uk; goran.neshich@embrapa.br

Abstract

Background: Membrane proteins constitute approximately 20–30% of all proteomes and represent over 60% of current drug targets. Although protein–lipid interactions play important structural and regulatory roles in membrane-associated proteins, most existing structural resources focus on identifying whether a residue lies within a membrane region, typically inferred from computational hydrophobicity-based positioning algorithms. This approach does not directly address a distinct biological question: which residues at the protein surface make direct physical contact with lipid molecules? Answering this question from experimental data is critical for understanding lipid-mediated allostery, designing lipid-mimetic therapeutics, and training accurate machine learning models for lipid binding site prediction. **Findings:** We present MPLID (Membrane Protein-Lipid Interaction Database), a curated residue-level dataset comprising 4,704 membrane proteins representing 813 sequence clusters at 30% identity, 8,055,325 residues, and 80,439 experimentally validated lipid contact annotations (1.00% observed positive rate). Labels are derived exclusively from crystallized lipid molecules resolved in Protein Data Bank structures using a 4.0 Å all-atom heavy-atom distance cutoff. Because most native lipid interactions are lost during purification and crystallization, this observed rate represents a lower bound, and the non-contact class inevitably contains false negatives. The dataset recognizes 117 lipid codes across ten functional categories (cross-validated against the RCSB Chemical Component Dictionary), spanning phospholipids, cardiolipin, sphingolipids, sterols, fatty acids, glycerolipids, detergent mimetics (explicitly flagged), lipid A components, and CHARMM simulation nomenclature. To prevent data leakage, proteins are clustered at 30% sequence identity using MMseqs2, yielding 813 clusters partitioned into training (2,578), validation (1,051), and test (1,075) splits. Amino acid composition analysis reveals biologically consistent enrichment at lipid contact sites: tryptophan (1.88×), arginine (1.44×), glycine (1.36×), lysine (1.33×), and phenylalanine (1.23×) are enriched, while proline (0.51×), isoleucine (0.57×), and aspartate (0.59×) are depleted. **Conclusions:** MPLID addresses a distinct biological question compared to existing resources (OPM, MemBlob, BioDolphin/PLIP): identifying residues that directly contact experimentally resolved lipid molecules rather than those positioned within computationally defined membrane boundaries. With 4,704 proteins and over 8 million annotated residues, MPLID provides the scale needed for training deep learning models for lipid contact prediction, with direct applications in structure-guided drug design and membrane protein engineering. The dataset adheres to FAIR principles and is freely available under a CC0 public domain dedication. Structurally resolved contacts represent only a subset of the full biological lipid interaction landscape, and MPLID is intended as a ground-truth resource rather than a complete catalog of lipid binding sites.

Key words: membrane proteins, lipid-protein interactions, protein structure, machine learning dataset, structural biology, experimental validation, residue-level classification, FAIR data

Background

Membrane proteins are central to virtually all cellular processes, mediating transport of ions and solutes, signal transduction, energy conversion, and cell adhesion [1]. Genomic analyses consistently estimate that 20–30% of open reading frames in any given proteome encode membrane proteins [1], and pharmacological surveys have established that these proteins account for more than 60% of all current drug targets [2, 3]. Despite their biomedical importance, membrane proteins remain among the most challenging targets for structural

© The Author 2026. Published by Oxford University Press. All rights reserved. For permissions, please e-mail: journals.permissions@oup.com

and computational biology, in large part because their native functional context, the lipid bilayer, is difficult to recapitulate experimentally and model computationally.

A growing body of evidence demonstrates that lipid-protein interactions are not passive consequences of membrane embedding but rather active modulators of protein structure, dynamics, and function [4, 5]. Specific lipid binding events regulate ion channel gating [6], G protein-coupled receptor (GPCR) signaling [4], and transporter conformational cycling [7]. Cholesterol, for example, occupies defined binding sites on GPCRs that are structurally resolved at atomic resolution [8], and cardiolipin binding is essential for the function of mitochondrial respiratory chain complexes [9]. The diversity of lipid species in biological membranes, encompassing phospholipids, sphingolipids, sterols, and glycolipids [10, 11], creates a complex interaction landscape that shapes the behavior of membrane-embedded proteins.

Membrane zone versus lipid contact: two distinct questions

A fundamental distinction exists between two related but different biological questions in membrane protein structural biology. The first question, “Is this residue located within the membrane zone?”, has been addressed by computational databases such as the Orientations of Proteins in Membranes (OPM) database [12, 13]. OPM positions membrane proteins in a hydrophobic slab model using energy minimization against experimentally calibrated transfer free energy parameters, producing membrane boundaries for over 15,000 protein entries. This approach is invaluable for understanding membrane protein topology, but the resulting labels reflect a coarse spatial localization: typically 20–40% of residues in a transmembrane protein fall within the OPM-defined membrane zone.

The second question, “Does this residue make direct physical contact with a lipid molecule?”, requires experimental structural evidence. Crystallographic and cryo-electron microscopy structures frequently resolve lipid molecules at specific protein surface sites, revealing native lipid interaction partners at atomic resolution. These experimental contacts represent a far more stringent and specific annotation: in our dataset, only 1.00% of all residues in membrane proteins (including both surface-exposed and buried residues) are observed to make lipid contacts within 4.0 Å (using an all-atom distance criterion). However, this rate represents a lower bound on the true frequency of lipid contacts in biological membranes, because only a subset of native lipid interactions survive protein purification and are resolved in crystal or cryo-EM structures. Nonetheless, even as a lower bound, this rate is approximately 20- to 40-fold lower than the membrane zone positive rate, underscoring the specificity of the experimental lipid contact annotation.

This distinction has profound consequences for both biological interpretation and machine learning model development (Table 1). Membrane zone labels identify the general region of a protein that is embedded in the bilayer, while lipid contact labels pinpoint specific binding sites that may mediate functionally important lipid-protein interactions. For drug discovery, the latter is far more actionable: lipid contact sites represent potential targets for allosteric modulation by lipid-mimetic compounds [14], while membrane zone annotations provide no such site-level resolution. We emphasize that no OPM-derived residue labels were used at any stage of the MPLID construction pipeline; all residue annotations derive exclusively from experimentally resolved lipid molecules in PDB structures. OPM is discussed here solely to distinguish the complementary biological questions of membrane positioning versus direct lipid contact.

Table 1. Comparison of membrane zone and lipid contact prediction tasks. These represent fundamentally different biological questions with distinct label sources, positive rates, and downstream applications.

**Aspect**	**Membrane zone**	**Lipid contact**
Label source	Computational (OPM)	Experimental (PDB)
Biological question	In membrane slab?	Contacts lipid?
Observed positive rate	20–40%	∼1.00%[†]
Spatial resolution	Zone-level (∼30 Å)	Atomic (4.0 Å)
Validation	In silico energy	X-ray/cryo-EM
Key application	Topology prediction	Binding site identification

[†]Lower bound; the true lipid contact rate in vivo is likely substantially higher due to lipid loss during purification and incomplete resolution.

Existing resources and their limitations

Several resources provide information about membrane protein-lipid interactions, each with specific scope and limitations. The OPM database [12] remains the definitive resource for membrane protein positioning, providing computationally derived membrane boundaries for transmembrane and peripheral proteins. However, OPM labels represent theoretical membrane zones rather than experimentally resolved lipid interaction sites.

The MemProtMD database [15, 16] complements OPM by providing coarse-grained molecular dynamics (CG-MD) simulations of membrane proteins embedded in explicit phospholipid bilayers. While MemProtMD offers valuable insights into lipid organization around proteins, its contacts derive from simulations rather than experimental structures, and the database focuses on integral membrane proteins in a single lipid type (dipalmitoylphosphatidylcholine).

The MemBlob database and server [17] takes a complementary approach by analyzing cryo-EM density maps to computationally identify membrane-facing regions of transmembrane proteins. MemBlob classifies residues as solvent-facing, lipid-facing, at the lipid interface, or buried, providing a membrane environment annotation that bridges between the coarse OPM slab model and direct experimental contact data. While MemBlob provides valuable residue-level environment classification, its labels are derived from density map analysis rather than from resolved lipid molecules, and the database is limited to proteins with available cryo-EM reconstructions.

BioDolphin [18] identifies and catalogs all protein-lipid interactions across the PDB using the PLIP (Protein-Ligand Interaction Profiler) framework [19], providing detailed interaction-type annotations (hydrogen bonds, hydrophobic contacts, salt bridges) at the residue and atom level. BioDolphin offers API access and covers a broad range of interaction types, making it a powerful tool for detailed mechanistic analysis of individual protein-lipid complexes. MPLID differs from BioDolphin in its focus on providing pre-computed, ML-ready binary contact labels with cluster-aware

train/validation/test splits, continuous distance values, and FAIR-compliant dataset deposition, rather than on-demand interaction profiling.

The STING Relational DataBase (RDB) [20, 21] provides a comprehensive structural analysis platform that computes and stores inter-atomic contacts, surface accessibility, and residue-level descriptors for all PDB structures. While STING RDB does not specifically label lipid contacts, its contact calculation infrastructure and residue-level annotation framework informed the design philosophy of MPLID, particularly regarding the use of distance-based contact definitions and the importance of providing both binary labels and continuous distance values for downstream analysis.

On the prediction methodology side, the DREAMM method [22] demonstrated that ensemble machine learning can achieve a Matthews correlation coefficient (MCC) of 0.84 for predicting protein-membrane interfaces, using a curated set of 54 peripheral membrane proteins (later expanded to 65) with experimentally characterized membrane-penetrating residues. However, this training data was distributed as part of the prediction tool rather than as a standalone, documented dataset resource. More recently, PMIpred introduced a physics-informed transformer approach trained on molecular dynamics data for peptide-membrane interactions [23], and protein language model approaches have been explored using ProtTrans embeddings [24, 25]. While these studies have advanced prediction methodology, none provides a large-scale, publicly deposited, and documented dataset covering the full diversity of membrane protein classes.

The need for large-scale experimental lipid contact data

The convergence of three trends motivates the creation of MPLID. First, the rapid growth of the Protein Data Bank [26, 27], which now contains more than 230,000 structures, means that thousands of membrane protein structures with resolved lipid molecules are available for systematic analysis. Second, advances in protein language models such as ESM-2 [28] and ProtTrans [25] have demonstrated that per-residue embeddings trained on large-scale protein sequence data can capture structural and functional properties, but training supervised models on top of these representations requires large labeled datasets. Third, structure-guided drug discovery increasingly recognizes lipid binding sites as druggable targets for allosteric modulation [14, 6], creating demand for systematic catalogs of experimentally validated lipid interaction sites.

MPLID addresses these needs by providing experimentally validated lipid contact annotations for 4,704 membrane proteins containing 8,055,325 residues. All labels derive from crystallized lipid molecules in PDB structures, providing experimental ground truth at atomic resolution. While existing resources offer related information, including membrane environment classification from cryo-EM density (MemBlob [17]) and detailed interaction-type profiling (BioDolphin/PLIP [18, 19]), none provides a pre-computed, ML-ready residue-level dataset with cluster-aware evaluation splits and FAIR-compliant deposition. MPLID fills this gap by covering integral, peripheral, and single-pass membrane proteins across 813 sequence-diverse clusters.

Data Description

Dataset overview

MPLID provides residue-level binary annotations indicating whether each residue in a membrane protein makes direct physical contact with an experimentally resolved lipid molecule. The complete dataset encompasses 4,704 membrane proteins with a total of 8,055,325 residues, of which 80,439 (1.00%) are labeled as lipid contacts (Figure 1). This observed positive rate should be interpreted as a lower bound on the true frequency of protein-lipid contacts in biological membranes: many native lipid interactions are lost during protein purification and crystallization, and most PDB structures resolve only a small number of tightly bound or structurally ordered lipids. Consequently, the non-contact class (99.00%) inevitably contains a substantial proportion of false negatives, residues that contact lipids in vivo but lack a resolved lipid partner in the crystal or cryo-EM structure. The remaining residues classified as non-contacts include solvent-exposed domains, protein-protein interfaces, interior regions of multi-subunit complexes, and an unknown fraction of unresolved lipid-binding sites.

Proteins in the dataset range from 22 to 15,280 residues (median 1,038; mean 1,712), reflecting the diversity of membrane protein sizes from small single-pass transmembrane peptides to large multi-subunit complexes. The number of lipid contacts per protein ranges from 1 to 4,820 (median 4; mean 17), with the highly skewed distribution reflecting that most proteins have few resolved lipid contacts while a small number of well-characterized complexes have extensive lipid interactions. The maximum value (4,820 contacts for PDB 6W4F, an NMR-resolved structure of the KRAS4B homodimer on a lipid bilayer nanodisc) arises because the current pipeline counts residue-lipid contacts across all NMR conformational models deposited in the PDB coordinate file; 6W4F contains 20 NMR models, each with approximately 240 contact residues. A total of 41 NMR multi-model structures in the dataset are affected by this counting convention, contributing approximately 24,000 excess contact annotations (see Limitations). Every protein in the final dataset has at least one lipid contact, as proteins with zero contacts were removed during quality filtering (see Methods). This removal excludes membrane proteins whose lipids were stripped during purification, meaning that MPLID does not represent the complete membrane proteome and cannot serve as a source of proteome-wide negative controls.

Data schema and format

Each record in MPLID contains ten fields that provide complete information for each annotated residue. The `pdb_id` field contains the four-character PDB accession code, and `chain_id` specifies the polypeptide chain within the structure. The `residue_number` gives the residue's position in the chain, while `residue_name` provides the three-letter amino acid code. The binary label `is_contact` takes a value of 1 for residues where any heavy atom is within 4.0 Å of any lipid heavy atom and 0 otherwise. The `label_source` field is set to "EXPERIMENTAL" for all entries, confirming that labels derive from experimentally resolved lipid molecules in PDB structures rather than computational predictions. The `confidence` field is set to "high" for all entries in the current release because all labels derive from experimentally resolved structures; this field is retained as a placeholder for future quality scoring that may incorporate

structure resolution and B-factor data. The `min_distance` field records the minimum distance in Angstroms between any heavy atom of the residue and the nearest lipid heavy atom. Finally, `cluster_id` assigns each protein to a sequence cluster, and `split` indicates assignment to training, validation, or test partitions.

Data are distributed as gzip-compressed comma-separated values (CSV) files partitioned by split (train, validation, test), along with a protein-level metadata file containing per-protein summary statistics including residue counts, contact counts, contact rates, cluster assignments, and split assignments.

Train/validation/test splits

The dataset is partitioned into training (2,578 proteins; 4,907,696 residues), validation (1,051 proteins; 1,403,838 residues), and test (1,075 proteins; 1,743,791 residues) splits (Figure 1). Critically, splitting is performed at the protein level with cluster-aware stratification: proteins within the same sequence cluster are always assigned to the same split, preventing data leakage from sequence-similar proteins appearing in both training and evaluation partitions. Contact rates vary across splits (training 1.16%, validation 0.69%, test 0.79%) because stratification is performed at the cluster level rather than the residue level, and clusters with higher contact rates may be unevenly distributed. This variation is expected and should be accounted for when comparing model performance across splits. This design choice is essential for meaningful generalization assessment, as protein-level leakage can dramatically inflate performance estimates in structural bioinformatics tasks.

The cluster-aware splitting strategy uses MMseqs2 [29] with a 30% sequence identity threshold and 80% alignment coverage, producing 813 clusters. Entire clusters are assigned to splits to ensure that no protein in the validation or test sets shares more than 30% sequence identity with any training protein. This threshold is substantially more conservative than the 40% identity commonly used in protein function prediction benchmarks [30], further reducing the risk of overestimating generalization performance.

Sequence clustering analysis

The 813 sequence clusters exhibit a highly skewed size distribution (Figure 2), with 373 singletons (45.9%) and a largest cluster of 173 proteins, reflecting the sequence diversity of membrane proteins across different families and superfamilies. This clustering pattern indicates substantial structural and functional diversity in the dataset, as singleton clusters represent unique membrane protein families without close homologs.

Analyses

Amino acid composition at lipid contact sites

Analysis of amino acid enrichment at lipid contact sites reveals biologically meaningful and interpretable patterns (Figure 3). Enrichment ratios were calculated as the fraction of each amino acid among contact residues divided by its fraction in the complete dataset. Tryptophan (1.88×) shows the strongest enrichment, consistent with the well-established role of aromatic residues in anchoring transmembrane proteins at the membrane-water interface through favorable interactions with lipid headgroups via cation-π and hydrogen bonding interactions [4]. Notably, positively charged residues are strongly enriched: arginine (1.44×) and lysine (1.33×) rank among the top five most enriched amino acids, consistent with the "positive-inside rule" [31] and with the electrostatic attraction of cationic side chains to anionic phospholipid headgroups. Glycine (1.36×) is enriched, reflecting its prevalence in GxxxG motifs that mediate transmembrane helix-helix packing and create lipid-accessible surfaces at helix interfaces [32]. Phenylalanine (1.23×) and tyrosine (1.08×) are moderately enriched, contributing to the aromatic belt. Among other residues, cysteine (1.15×), serine (1.13×), and histidine (1.11×) show mild enrichment, with histidine functioning as an interfacial anchoring residue through its amphipathic imidazole ring, and cysteine enrichment likely reflecting palmitoylation sites and disulfide-constrained loop regions at lipid interfaces. Leucine (1.08×) is slightly enriched, reflecting its involvement in direct side-chain contacts with lipid acyl chains.

Among depleted residues, proline (0.51×) and isoleucine (0.57×) show the strongest depletion. Proline disrupts regular secondary structure and is underrepresented at smooth lipid-protein interfaces. The depletion of isoleucine, a β-branched hydrophobic residue, may reflect its preference for buried core positions rather than surface lipid contacts. Aspartate (0.59×) is depleted, consistent with the electrostatic incompatibility of negatively charged side chains with the lipid bilayer interior. Asparagine (0.71×) and valine (0.73×) are moderately depleted. Threonine (0.87×) and glutamine (0.89×) show mild depletion, while alanine (0.94×) and glutamate (0.97×) are near neutral. Overall, the enrichment pattern strongly supports the biological validity of the all-atom contact definition: residues capable of electrostatic interactions with phospholipid headgroups (arginine, lysine) and aromatic anchoring at the interface (tryptophan, phenylalanine) are enriched, while conformationally constrained (proline) and β-branched (isoleucine, valine) residues are depleted. These patterns are consistent with previous analyses of cardiolipin binding site composition [33] and likely reflect, in part, the overrepresentation of anionic lipids (particularly cardiolipin and phosphatidylglycerol) among structurally resolved lipid species. Because anionic lipids are more readily co-purified and crystallized with membrane proteins than zwitterionic species such as phosphatidylcholine and phosphatidylethanolamine, the observed enrichment of positively charged residues may be amplified relative to the in vivo membrane environment, where the majority of lipids are zwitterionic. Given the large sample sizes (80,439 contact residues among 8,055,325 total residues), these enrichment and depletion patterns are robust and unlikely to arise from sampling variation, but their magnitude may be influenced by the lipid composition bias inherent to crystallographic datasets.

Distance distribution analysis

The distribution of minimum distances between protein residues and lipid molecules provides independent validation of the 4.0 Å contact cutoff (Figure 4). Among contact residues, the distance distribution shows a clear peak at approximately 3.5–4.0 Å, corresponding to typical van der Waals contact distances between heavy atoms. The sharp boundary at 4.0 Å indicates that the cutoff effectively captures the transition from direct physical contact to non-contact. For non-contact residues, the distribution is broadly shifted toward larger distances, with a peak near 8–12 Å and a long tail extending to distances greater than 30 Å for residues in soluble domains far from any lipid molecule.

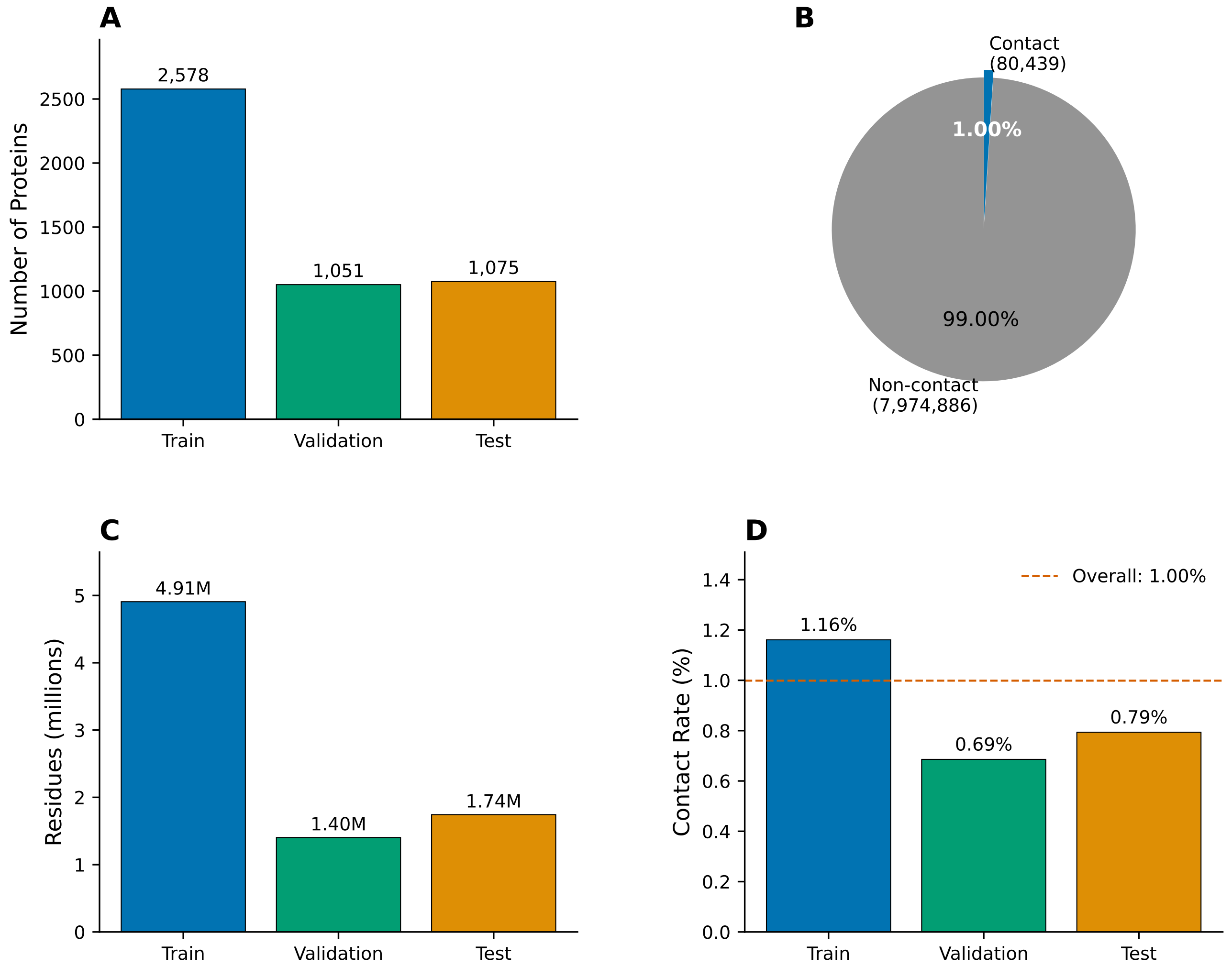

Fig. 1. MPLID dataset overview. (A) Distribution of proteins across training (54.8%), validation (22.3%), and test (22.9%) splits. (B) Class distribution showing the imbalance between contact (1.00%) and non-contact (99.00%) residues. (C) Total residues per split. (D) Contact rates across splits, showing variation due to cluster-level stratification: training (1.16%), validation (0.69%), and test (0.79%).

Protein size and contact density distributions

The distribution of protein sizes in MPLID spans three orders of magnitude, reflecting the diversity of membrane protein families represented (Figure 5). Contact density, defined as the fraction of residues making lipid contacts, varies substantially across proteins, with a per-protein median contact rate of 0.45% and a mean of 0.99%. The observed contact rate of 1.00% reflects both the sparse resolution of lipid molecules in PDB structures and the inherent specificity of direct van der Waals contacts. This rate should be interpreted as a lower bound; the severe class imbalance (approximately 100:1 negative-to-positive ratio) includes a substantial fraction of false negatives in the non-contact class, where residues that contact lipids in vivo lack a resolved lipid partner in the experimental structure. This imbalance requires specialized machine learning approaches such as class weighting or focal loss.

Comparison with existing resources

Table 2 places MPLID in the context of existing membrane protein-lipid interaction resources (Figure 6). Each resource addresses a different aspect of the problem. OPM [12] provides computationally derived membrane zone boundaries for topology analysis. MemProtMD [15] offers coarse-grained simulation data for lipid organization. The DREAMM prediction method [22] used a curated set of 54 peripheral membrane proteins with experimentally characterized membrane-penetrating residues as training data, though this data was not released as a standalone documented dataset. MPLID fills this gap by providing the first large-scale, publicly deposited, and fully documented dataset of experimental lipid contact labels, covering 4,704 proteins across all membrane protein classes with FAIR-compliant metadata, a data dictionary, and pre-defined evaluation splits. The diversity of membrane protein families represented in MPLID, spanning 813 sequence clusters at 30% identity, ensures broad coverage of the membrane protein structural landscape. These resources are complementary rather than competing: researchers should select the appropriate resource based on their specific biological question, and models trained on one type of label may not transfer directly to another without domain adaptation.

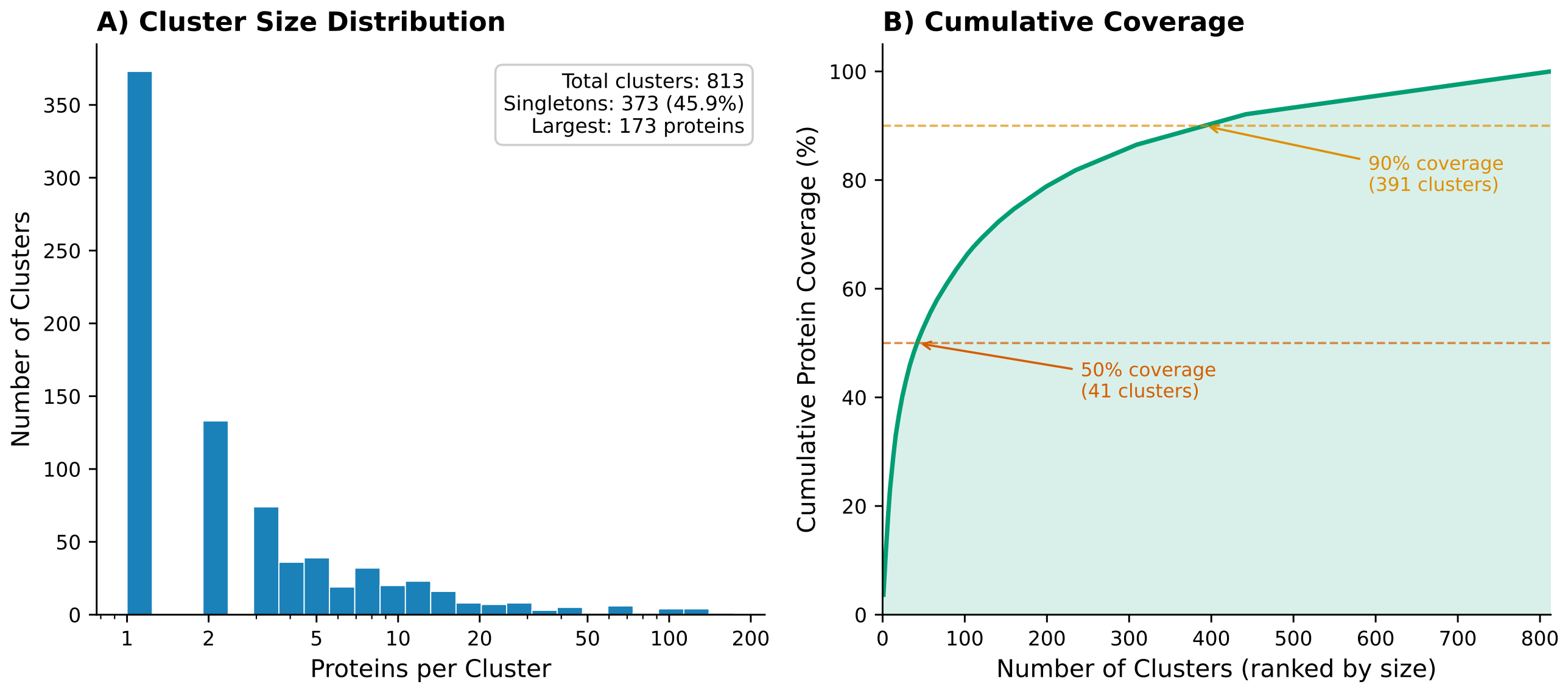

Fig. 2. Sequence cluster analysis. (A) Distribution of cluster sizes at 30% sequence identity on a log scale, showing a highly skewed size distribution with many singletons and a small number of large clusters containing many homologous structures. (B) Cumulative protein coverage ranked by cluster size, illustrating the concentration of proteins in a few large families.

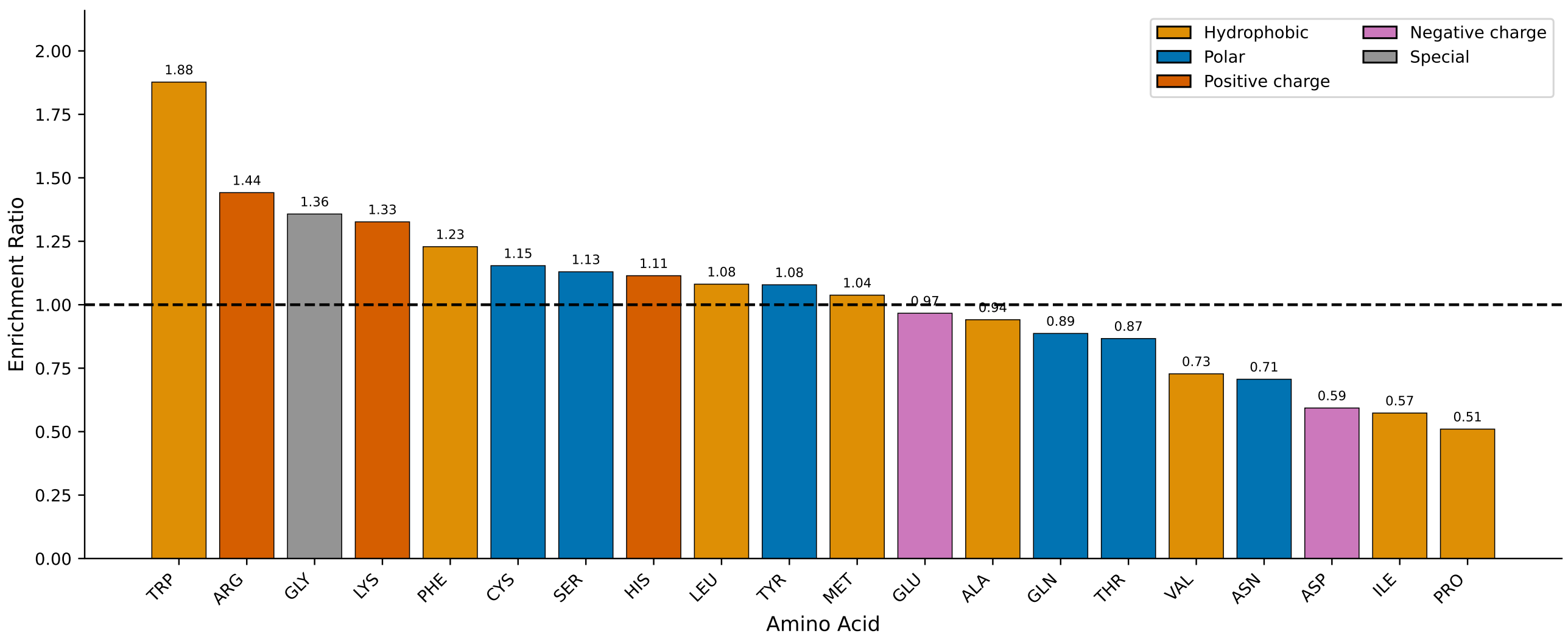

Fig. 3. Amino acid enrichment at lipid contact sites. Enrichment ratios are calculated as the frequency of each amino acid among contact residues divided by its overall frequency in the dataset. Colors indicate amino acid physicochemical properties: hydrophobic (orange), polar (blue), positive charge (red), negative charge (purple), and special (gray). The dashed line marks the expected frequency (1.0). Values above 1.0 indicate enrichment; values below 1.0 indicate depletion.

Table 2. Comparison of MPLID with existing membrane protein-lipid interaction resources. MPLID is the first large-scale, publicly deposited dataset providing experimentally validated residue-level lipid contact labels with FAIR-compliant documentation.

Resource	Proteins	Residues	Label type	Label source
MPLID (this work)	4,704	8,055,325	Lipid contact	Experimental (PDB)
BioDolphin [18][b]	PDB-wide	–	Interaction type	Experimental (PLIP)
MemBlob [17][c]	∼500	–	Membrane environment	Cryo-EM density
DREAMM [22][a]	54	∼15,000	Membrane interface	Experimental (curated)
OPM [12]	15,096	–	Membrane zone	Computational
MemProtMD [15]	∼3,500	–	Lipid contact	Simulated (CG-MD)

[a]Training data distributed within the DREAMM prediction tool repository; not released as a standalone documented dataset. [b]On-demand interaction profiling via PLIP; not pre-computed ML-ready dataset with splits. [c]Residue-level membrane environment classification from cryo-EM density maps.

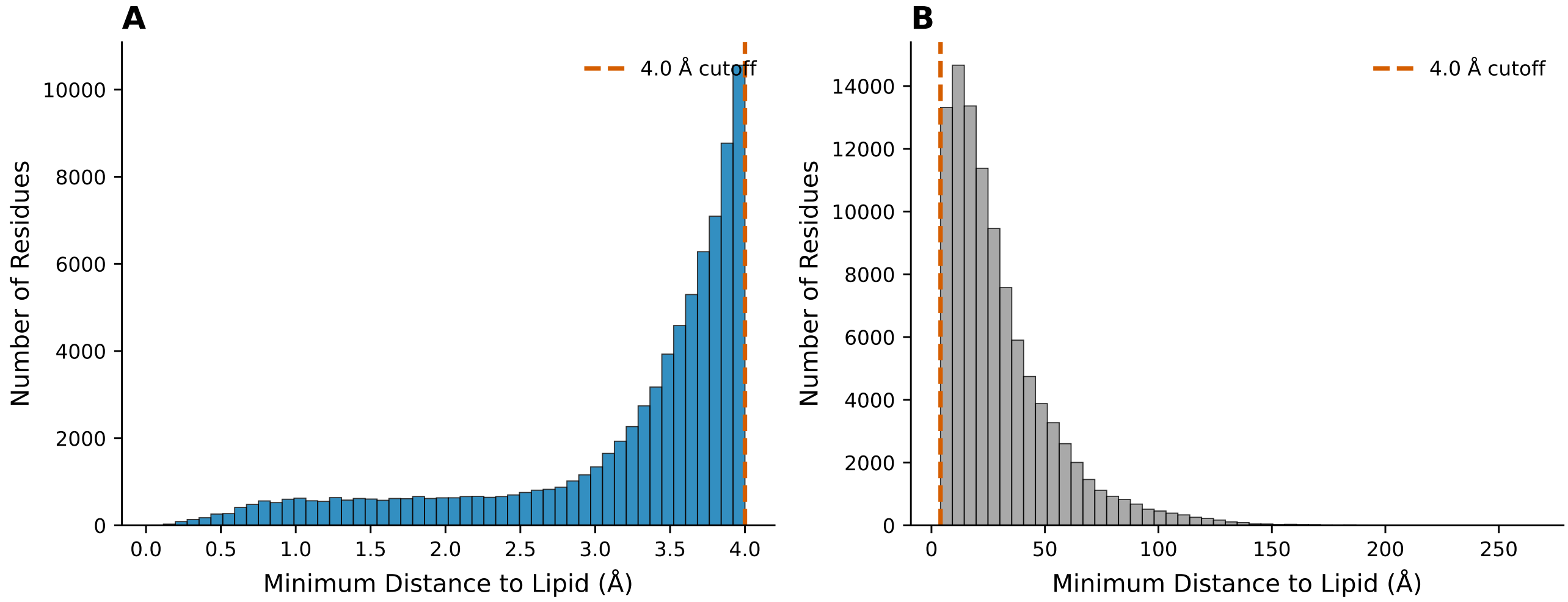

Fig. 4. Distribution of minimum residue-to-lipid distances. (A) Contact residues (≤4.0 Å) show a peak near 3.5–4.0 Å, consistent with van der Waals contact distances. (B) Non-contact residues display a broad distribution with distances extending well beyond 30 Å, confirming clear separation between contacting and non-contacting populations.

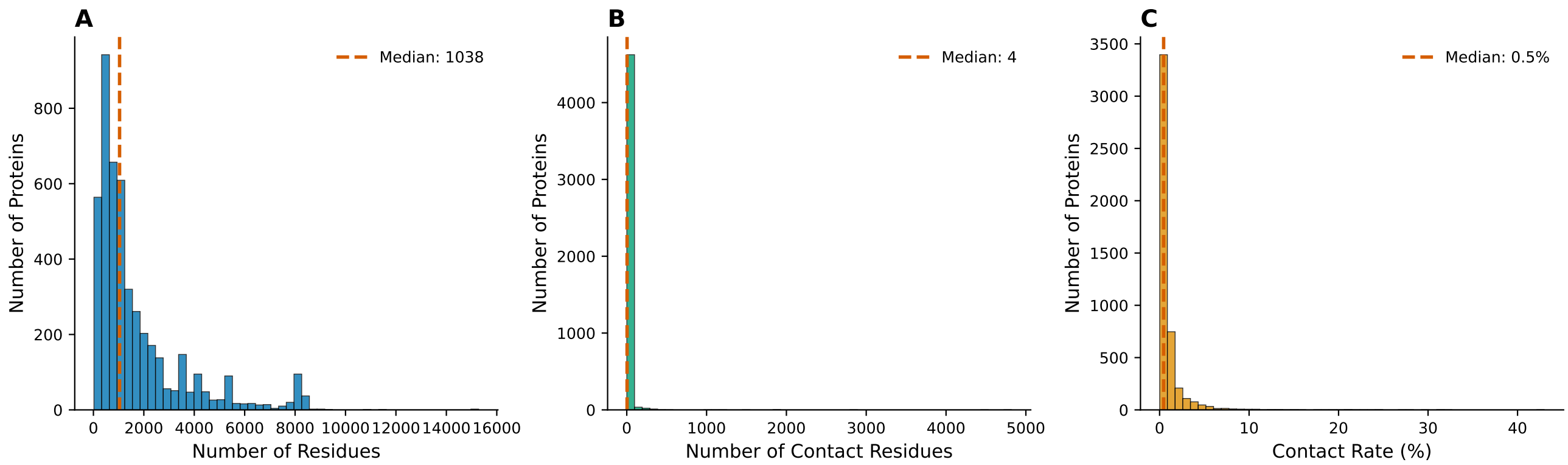

Fig. 5. Protein-level statistics. Distribution of protein sizes and contact densities across the MPLID dataset, illustrating the diversity of membrane protein families represented.

These resources form a complementary hierarchy addressing different aspects of membrane protein-lipid biology, and should be viewed as complementary rather than competing: OPM [12] provides membrane localization and hydrophobic slab positioning; MemBlob [17] infers membrane-facing environment from cryo-EM density; BioDolphin/PLIP [18, 19] provides detailed interaction-type profiling across all PDB ligands; and MPLID provides pre-computed, ML-ready residue-level lipid contact labels with evaluation splits. Models trained on one type of label cannot be directly evaluated on another, and researchers should select the appropriate resource based on their specific biological question.

Lipid type diversity

MPLID recognizes 117 lipid codes organized into ten functional categories, comprising 90 PDB Chemical Component Dictionary codes and 27 CHARMM simulation lipid identifiers commonly found in cryo-EM depositions. All 90 PDB codes were cross-validated against the RCSB Chemical Component Dictionary API during revision; the complete lipid ontology with RCSB verification status, official chemical names, and category assignments is provided in Supplementary Table S1. Phospholipids include cardiolipin (CDL, found in 675 structures), phosphocholine variants (PCW, 196 structures), phosphoethanolamine (PEE, 273 structures), and phosphoglycerol (PGV, 144 structures). Sterols are represented by cholesterol (CLR, 1,119 structures) and derivatives including cholic acid (CHD, 151 structures; a bile acid sterol derivative) and cholesterol hemisuccinate (Y01). Fatty acids include palmitate (PLM, 734 structures), oleic acid (OLA, 379 structures), myristate (MYR, 426 structures), and stearate (STE, 94 structures). Sphingolipids include sphingosine (SPH, 102 structures) and sphingosine-1-phosphate (S1P, 16 structures). Detergent mimetics used in crystallization, which often occupy native lipid binding sites, include dodecyl β-D-maltoside (LMT, 677 structures), octyl β-D-glucopyranoside (BOG, 510 structures), and lauryldimethylamine oxide (LDA, 263 structures). The protein-level metadata includes an explicit `has_detergent_only` flag indicating whether all lipid contacts for a given protein derive exclusively from detergent mimetics rather than physiological lipid species, enabling users to filter detergent-only entries if desired.

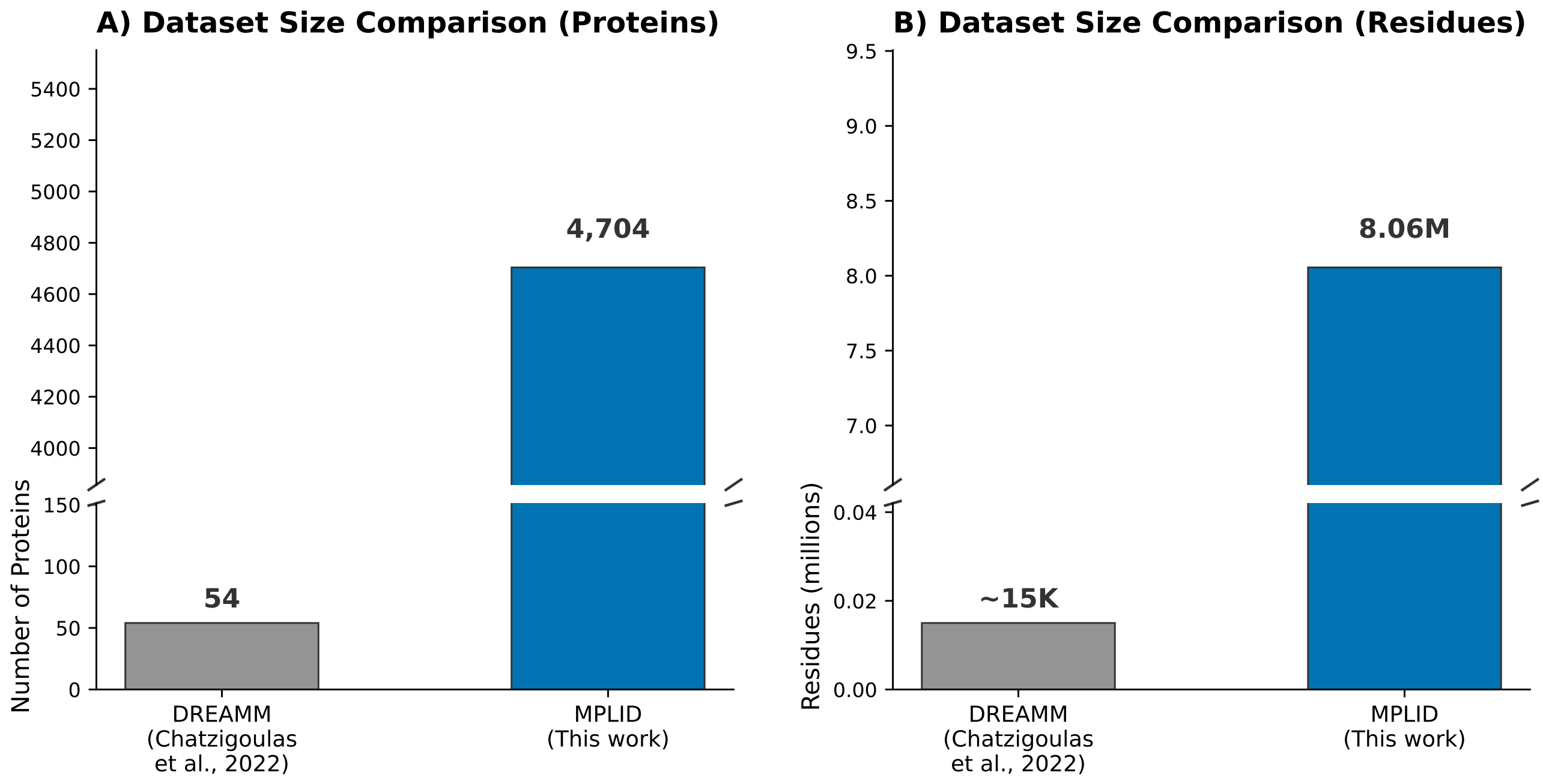

Fig. 6. Scale comparison between MPLID and the training data used by the DREAMM prediction method [22], the largest prior collection of experimentally annotated membrane protein-lipid interactions. (A) Number of proteins (4,704 versus 54). (B) Number of annotated residues (8,055,325 versus ∼15,000). Note that these resources differ in scope: DREAMM focuses on peripheral membrane proteins, while MPLID covers all membrane protein classes.

Notably, several biologically important lipid classes are absent or underrepresented in the current MPLID release. Phosphatidic acid (PA), phosphatidylserine (PS), and phosphoinositides (PI, PIP, PIP_2, PIP_3) are not represented among the recognized lipid codes, despite their well-established roles in membrane protein regulation. This absence reflects both the difficulty of resolving these lipids crystallographically (owing to their flexible headgroups and transient binding modes) and the focus of MPLID on lipid species that are currently represented in PDB HETATM records. Similarly, polyunsaturated fatty acids (e.g., docosahexaenoic acid, arachidonic acid) are rarely resolved in crystal structures. Future releases will expand lipid coverage as more structures with these lipid types become available in the PDB.

Structural visualization of lipid contacts

To illustrate the nature of the lipid contact annotations in MPLID, representative membrane protein structures with their resolved lipid molecules are shown in Figure 7. These examples demonstrate that lipid contacts occur at specific, well-defined sites on the protein surface rather than uniformly across the membrane-exposed region. Contact residues (highlighted in red/orange) are interspersed with non-contact residues even in transmembrane regions, confirming that lipid contact prediction is a more specific task than membrane zone classification.

Computational label comparison studies

The availability of MPLID alongside OPM membrane zone annotations enables systematic comparison of experimental lipid contacts with computational membrane boundaries. We compared MPLID contact labels with OPM-derived membrane zone assignments across proteins present in both resources (Figure 8). The number of overlapping proteins varies by analysis: 2,383 proteins contributed to precision estimates, 1,685 to recall estimates, and 2,457 to contact distribution analysis, reflecting differences in the availability of OPM membrane zone annotations and non-zero lipid contacts across the shared set. Of all MPLID lipid contacts in the shared set ($n = 28{,}497$ residues), 51.2% fell within the OPM membrane zone while 48.8% were located outside the computationally defined boundaries, likely reflecting interfacial contacts, re-entrant loops, or amphipathic helices that extend beyond the hydrophobic core. Conversely, 98.8% of OPM membrane zone residues ($n = 1{,}170{,}590$) showed no direct lipid contact within 4.0 Å, and the per-protein median was only 0.7% of membrane zone residues having lipid contacts. The OPM membrane zone captured a median of 50.0% of lipid contacts per protein. These dataset-wide statistics highlight the fundamental difference between membrane zone classification and lipid contact prediction: being embedded in the membrane is neither sufficient nor necessary for direct lipid interaction. Researchers working with MemProtMD [15] simulations can similarly compare simulated lipid contacts with the experimental contacts provided by MPLID.

Methods

Data source integration

MPLID was constructed using a purely experimental approach with no dependence on computational membrane positioning databases. OPM data were not used at any stage of the MPLID construction pipeline; OPM is discussed in this paper solely to contextualize the complementary biological questions of membrane zone positioning versus direct lipid contact annotation. All residue annotations in MPLID derive

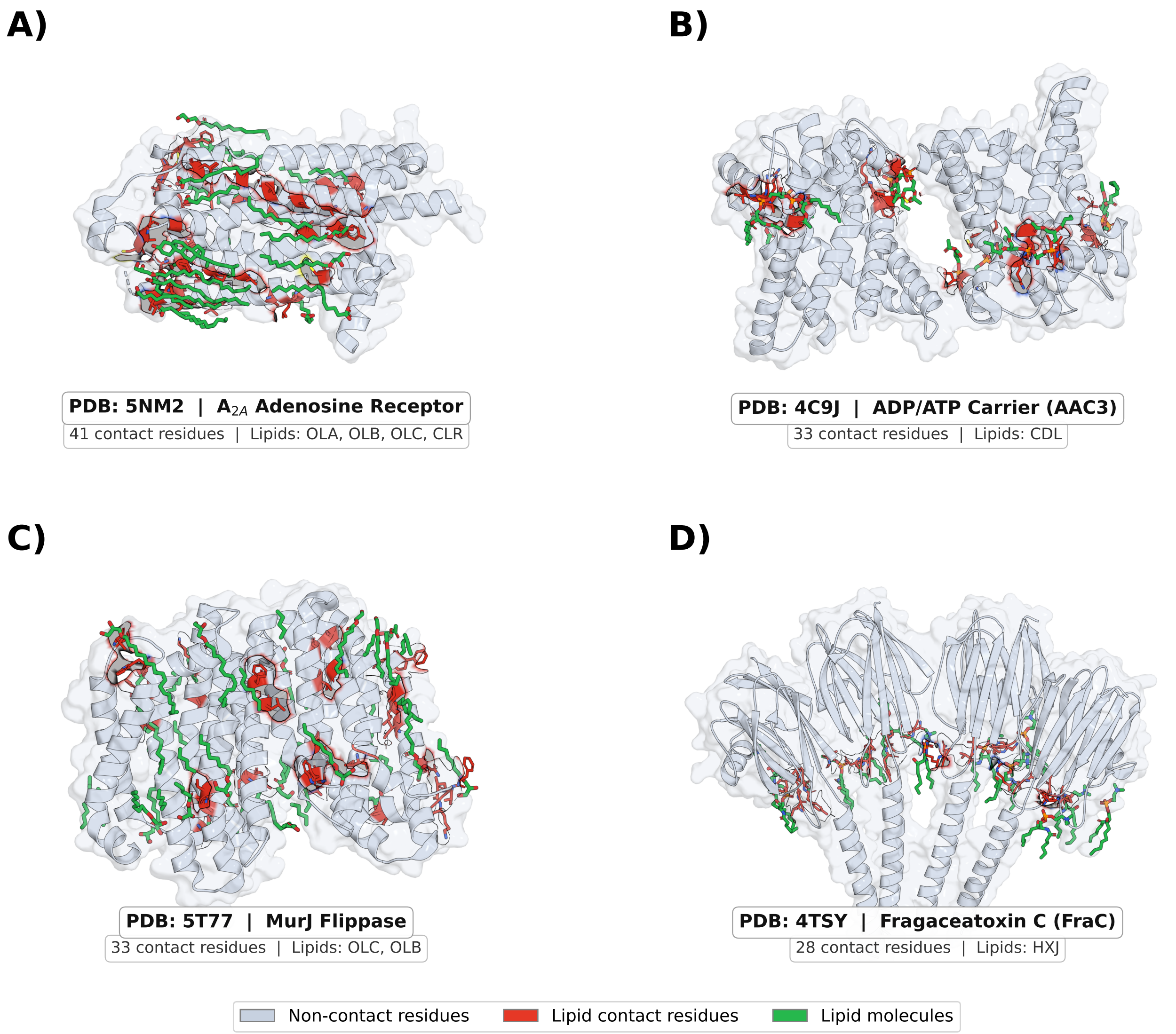

Fig. 7. Structural visualization of MPLID lipid contact annotations across four representative membrane protein classes. (A) A_{2A} adenosine receptor (PDB: 5NM2), a GPCR with multiple lipid types including oleic acid and cholesterol. (B) ADP/ATP carrier AAC3 (PDB: 4C9J), a mitochondrial transporter with cardiolipin contact residues across two chains. (C) MurJ flippase (PDB: 5T77), a lipid II flippase with contacts distributed across its transmembrane helices. (D) Fragaceatoxin C (PDB: 4TSY), a tetrameric pore-forming toxin with sphingolipid contacts at the membrane insertion interface. These examples demonstrate that lipid contacts are site-specific rather than uniformly distributed across membrane-exposed surfaces.

exclusively from experimentally resolved lipid molecules in PDB structures. The RCSB Protein Data Bank [26, 27] was queried programmatically for all structures containing recognized lipid chemical components, identifying 8,221 candidate structures with lipid ligands. This query-first strategy ensures that every protein in the dataset has at least one experimentally resolved lipid molecule, providing an unbiased sampling of protein-lipid interactions across all structural classes. The identified structures were downloaded as original PDB coordinate files (9,796 files total, as some entries required retrieval of biological assembly files or alternate depositions). After parsing, 5,723 unique proteins yielded valid structures with recognized HETATM lipid records; the remainder comprised duplicate chains, structures that failed parsing, or entries lacking standard amino acid residues. These were processed through the contact calculation and quality filtering pipeline described in the following subsections.

Lipid molecule identification

Lipid molecules in PDB structures were identified using their Chemical Component Dictionary codes. We compiled a list of 117 lipid codes organized into ten functional categories: phospholipids (CDL, POV, PCW, PEE, PGV, and others), cardiolipin (CDL, C9V, 18W, LCL), sphingolipids (SPH, S1P, HXJ, CRT), sterols (CLR, CHD, Y01, ERG), fatty acids (PLM, MYR, OLA, STE, ARA, DHA), glycerolipids (TGL, DAG, MAG), detergent mimetics (LDA, LMT, BOG, OLC, DPC), lipid A components (LPA, KDO, 6LP), and CHARMM simulation nomenclature (POPC, POPE, POPG, and others commonly found in cryo-EM depositions). This list was developed iteratively by cross-referencing the PDB Chemical

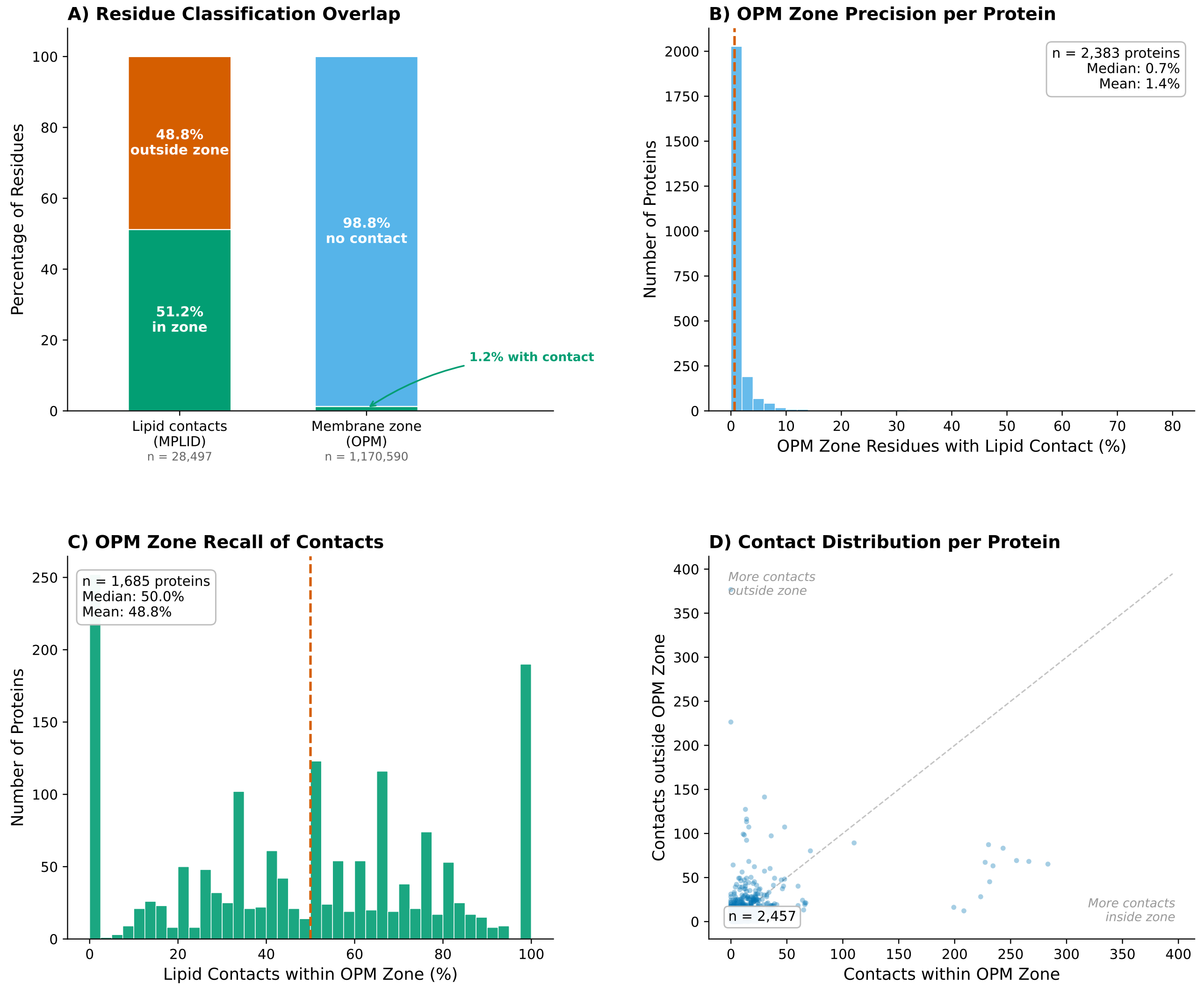

Fig. 8. Quantitative comparison of MPLID lipid contacts versus OPM membrane zone annotations. (A) Residue classification overlap: 48.8% of lipid contacts (n = 28,497) fall outside the OPM membrane zone, while 98.8% of OPM zone residues (n = 1,170,590) lack direct lipid contact. (B) Per-protein OPM precision (n = 2,383 proteins): median 0.7% of membrane zone residues have lipid contacts. (C) Per-protein OPM recall (n = 1,685 proteins): the membrane zone captures a median of 50.0% of lipid contacts. (D) Per-protein scatter of contacts inside versus outside the OPM zone (n = 2,457 proteins). These results demonstrate that membrane zone classification and lipid contact annotation address fundamentally different biological questions.

Component Dictionary with lipid classification databases and manually curating entries to exclude non-lipid small molecules that might share similar chemical codes. Detergent mimetics were included because they frequently occupy native lipid binding sites in membrane protein crystal structures and provide relevant information about lipid-protein interaction geometry [7].

Structure processing and contact calculation

For each candidate protein, the original PDB coordinate file was downloaded from RCSB to preserve HETATM records containing lipid molecule coordinates. Structure parsing was performed using BioPython [34], extracting all standard amino acid residues (ATOM records) and all recognized lipid molecules (HETATM records). Non-standard residues, water molecules, metal ions, and non-lipid ligands were excluded.

Lipid contacts were defined using an all-atom distance criterion. For each protein residue R and each lipid molecule L in the structure, the minimum distance $d_{\min}(R, L)$ between any heavy (non-hydrogen) atom of R and any heavy atom of L was calculated. A residue was labeled as a lipid contact if:

$$d_{\min}(R, L) = \min_{a \in R,\, b \in L} \|\mathbf{r}_a - \mathbf{r}_b\| \leq 4.0 \text{ Å} \quad (1)$$

where $\mathbf{r}_a$ denotes the Cartesian coordinates of heavy atom a in residue R and $\mathbf{r}_b$ denotes the coordinates of heavy atom b in lipid L. The use of all heavy atoms (backbone and side chain) as residue representatives captures direct van der Waals contacts that would be missed by a Cα-only criterion, particularly for residues whose side chains extend into lipid-binding pockets while their backbone remains distant from lipid molecules. The 4.0 Å cutoff was chosen to capture direct van der Waals contacts between heavy atoms, consistent with standard distance thresholds used in protein-ligand interaction

analysis [19], protein contact databases [20], and previous membrane protein-lipid interaction studies [22]. Typical van der Waals radii for carbon, nitrogen, and oxygen atoms sum to contact distances of 3.2–3.8 Å, and the 4.0 Å threshold provides a small margin to accommodate thermal displacement while remaining well below the threshold for indirect (water-mediated or electrostatic) interactions. The distance distribution analysis (Figure 4) independently validates this choice, showing a clear peak at 3.5–4.0 Å corresponding to van der Waals contacts. We note that this cutoff does not distinguish between contacts with lipid headgroups and acyl chains; both are captured by the all-atom criterion. Researchers interested in headgroup-specific or acyl-chain-specific contacts can use the continuous `min_distance` values together with the lipid atom identities available in the original PDB coordinate files. For each residue, the minimum all-atom distance to the nearest lipid heavy atom was recorded regardless of contact status, providing a continuous distance metric that enables researchers to evaluate alternative cutoff values.

Quality filtering

Of the 5,723 proteins with parseable lipid-containing structures, 1,019 were removed because they contained only non-lipid molecules misannotated as lipids (such as HEPES buffer and bacteriochlorophyll) or had all lipid atoms beyond the 4.0 Å contact threshold, leaving 4,704 proteins with at least one genuine lipid contact. This filtering ensures that every protein in the final dataset has genuine, experimentally resolved lipid contacts.

Sequence clustering

To prevent data leakage between training, validation, and test splits, all 4,704 proteins were clustered using MMseqs2 [29] with a 30% sequence identity threshold and 80% bidirectional alignment coverage. These stringent thresholds ensure that proteins assigned to different splits are sufficiently divergent in sequence to represent independent test cases. The clustering produced 813 clusters.

Split strategy

Clusters were assigned to training, validation, and test splits using stratified random sampling at the cluster level, with stratification based on the average contact rate per cluster. This approach ensures that each split contains a representative distribution of high-contact and low-contact proteins. Entire clusters were assigned to a single split, guaranteeing that no two proteins sharing more than 30% sequence identity appear in different partitions. The resulting splits contain 2,578 training proteins (54.8%), 1,051 validation proteins (22.3%), and 1,075 test proteins (22.9%).

Software and computational environment

The MPLID construction pipeline was implemented in Python 3.11.8 using BioPython 1.83 [34] for PDB structure parsing, pandas 2.2.2 [35] for data manipulation, NumPy 1.26.4 for numerical computation, SciPy 1.13 for distance calculations, and scikit-learn 1.7.2 [36] for data splitting. MMseqs2 version 15 [29] was used for sequence clustering. The RCSB PDB search API was used for programmatic identification of structures containing lipid chemical components.

Discussion

MPLID represents the first large-scale, publicly deposited dataset of experimentally validated residue-level lipid contacts for membrane proteins, complementing existing resources such as OPM (membrane zone positioning), MemBlob (membrane environment inference from cryo-EM density), and BioDolphin/PLIP (generalized structural interaction profiling). The dataset addresses a biological question that is fundamentally distinct from membrane zone classification: rather than identifying residues located within a computationally defined hydrophobic slab, MPLID annotates residues that make direct physical contact with experimentally resolved lipid molecules at atomic resolution. Importantly, MPLID captures only the subset of protein-lipid interactions that survive purification and are resolved in crystal or cryo-EM structures; the full biological lipid interaction landscape is substantially richer. The analyses presented here validate this annotation approach through multiple independent lines of evidence. The amino acid enrichment patterns at lipid contact sites are biologically consistent, with aromatic anchoring residues (tryptophan, phenylalanine) and positively charged residues (arginine, lysine) enriched at contact sites, while conformationally constrained (proline) and negatively charged (aspartate) residues are depleted, consistent with independent analyses of cardiolipin binding sites [33]. The distance distribution analysis confirms that the 4.0 Å cutoff effectively captures the van der Waals contact boundary. The quantitative comparison with OPM membrane zone annotations demonstrates that nearly half of experimentally observed lipid contacts fall outside computationally predicted membrane boundaries, reinforcing the complementary nature of these resources.

The scale of MPLID, comprising 4,704 proteins across 813 sequence clusters, provides substantially greater coverage than prior experimental lipid contact datasets. The DREAMM training set [22], the largest previously available collection, contained 54 peripheral membrane proteins. MPLID expands this by nearly two orders of magnitude while covering all membrane protein classes, including integral, peripheral, and single-pass proteins. The cluster-aware splitting strategy, using a conservative 30% sequence identity threshold, ensures that evaluation on the test set reflects genuine generalization rather than sequence memorization.

Limitations

Several limitations should be noted when using MPLID.

False negatives in the non-contact class.

Lipid contact labels depend on the crystallographic or cryo-EM resolution and completeness of resolved lipid molecules; many native lipid binding events are not captured because lipids are disordered or stripped during purification and crystallization. Consequently, the false negative rate in the non-contact class is likely substantial, potentially exceeding the number of true positive contacts by an order of magnitude. The true frequency of lipid contacts in membrane proteins in vivo is almost certainly higher than the 1.00% observed in MPLID. Moreover, many structures contain only one or a few resolved lipid molecules, meaning that the observed contacts represent a sparse snapshot of the full lipid-binding landscape rather than a comprehensive map of all possible interaction sites. Users training machine learning models on MPLID should be aware that the non-contact class is noisy and that model performance

may be limited by false negative labels rather than by model capacity.

Detergent mimetics.

The inclusion of detergent mimetics as proxy lipids introduces potential noise, as detergent binding sites may not perfectly recapitulate native lipid interactions. Analysis of HETATM records across all 4,704 processed PDB files shows that 1,039 proteins (22.1%) contain exclusively detergent mimetics (e.g., dodecyl maltoside, octyl glucoside, lauryldimethylamine oxide) without any physiological lipid species, while an additional 685 proteins (14.6%) contain both detergent and physiological lipids. This means that for roughly one in five proteins, all contact labels derive from crystallization detergents rather than native lipid species. Detergents are retained in the dataset because they occupy membrane-associated structural environments and are part of the experimental structural context; however, the protein-level metadata includes an explicit detergent-only flag to enable filtering. The cholesterol derivative CHD (cholic acid, a bile acid sterol derivative) and Y01 (cholesterol hemisuccinate) are distinct compounds with different PDB Chemical Component Dictionary identities ($C_{24}H_{40}O_5$ and $C_{31}H_{50}O_4$, respectively), though both are classified as sterols in MPLID. Users should note that cholesterol hemisuccinate is an artificial detergent commonly added during membrane protein solubilization and is not a native membrane lipid.

Lipid code verification.

During revision, all 90 PDB Chemical Component Dictionary codes were cross-validated against the RCSB Chemical Component Dictionary API (Supplementary Table S1). This audit confirmed 45 codes as correctly classified, identified 34 codes with incorrect or ambiguous category assignments, and found 11 codes absent from the dictionary. Of the 34 misclassified codes, 15 appear in PDB HETATM records within the dataset, but most have low occurrence. The codes with the largest impact are CHD (cholic acid, present in 150 structures; name corrected from “cholesteryl hemisuccinate”), CRT (spirilloxanthin, a bacterial carotenoid incorrectly classified as ceramide, present in 22 structures), and ARA (alpha-L-arabinopyranose, a sugar incorrectly classified as arachidonic acid, present in 18 structures). These misclassified contacts collectively represent fewer than 300 protein entries and do not materially alter the dataset statistics. The three codes specifically flagged by reviewers (LNS, LNN, GLF) are confirmed as misclassified but do not appear in any PDB structure in the dataset (zero impact). The complete verification results are provided in Supplementary Table S1 for transparency.

NMR multi-model structures.

The current pipeline counts residue-lipid contacts across all conformational models in NMR-derived PDB coordinate files rather than extracting contacts from a single representative model. This convention affects 41 NMR structures in the dataset, contributing approximately 133,000 excess residue entries (1.65% of total residues) and approximately 24,000 excess contact annotations. The most extreme case is PDB 6W4F (KRAS4B homodimer on a lipid bilayer nanodisc), which contains 20 NMR models and reports 4,820 contacts across all models, compared to approximately 240 unique contact residues per individual model. Users working with NMR structures should be aware of this multi-model counting convention. Future releases will adopt a single-model extraction strategy for NMR structures.

PDB and lipid composition biases.

The dataset inherits biases from the PDB toward well-studied, readily crystallizable membrane protein families, leaving many therapeutically relevant but structurally uncharacterized families underrepresented. Several biologically important lipid classes are absent, including phosphatidic acid (PA), phosphatidylserine (PS), phosphoinositides (PI, PIP, PIP_2, PIP_3), and polyunsaturated fatty acids. This absence reflects the difficulty of resolving these lipids crystallographically rather than a deliberate exclusion. Anionic lipids (particularly cardiolipin and phosphatidylglycerol) are overrepresented among structurally resolved lipid contacts relative to their abundance in biological membranes, which has implications for machine learning models trained on MPLID: such models may learn structural detectability biases rather than purely biological binding affinity patterns. Additionally, the removal of 1,019 proteins with zero contacts during quality filtering means that MPLID does not represent the complete membrane proteome and cannot serve as a source of proteome-wide negative controls.

Distance cutoff and static snapshots.

The 4.0 Å distance cutoff is a single threshold that may not be optimal for all lipid types; researchers are encouraged to explore alternative cutoffs using the continuous distance values provided in the dataset. The cutoff does not distinguish between headgroup and acyl-chain contacts, both of which are captured by the all-atom criterion. Because MPLID annotations are derived from static crystallographic and cryo-EM snapshots, they may not fully represent the dynamic nature of protein-lipid interactions in native membrane environments, where lipids undergo lateral diffusion and exchange on nanosecond-to-microsecond timescales. Crystal packing artifacts may also contribute false positive contacts, as lipid molecules resolved in crystal structures can occupy lattice contact interfaces that do not reflect native membrane binding.

Additional technical notes.

No structure resolution cutoff was applied during dataset construction: all PDB structures with recognized lipid molecules were included regardless of crystallographic resolution. While this maximizes dataset size, low-resolution structures (worse than 3.5 Å) may have less reliable lipid positions, potentially introducing noise into contact labels. Researchers working with resolution-sensitive applications are encouraged to filter by PDB resolution using the provided `pdb_id` field. The current release does not include per-residue lipid chemical component codes, meaning that users cannot directly determine which lipid type contacts a given residue; however, the `pdb_id` field allows users to retrieve lipid identities from the original PDB structures. Lipid type stratification at the residue level is planned for a future release. Four selenocysteine (SEC) residues in the dataset are all annotated as lipid contacts (4/4, 100% contact rate); given the extremely small sample size, this observation is not statistically meaningful and these residues were excluded from enrichment ratio analysis.

Cross-validation of MPLID labels against BioDolphin [18] interaction annotations and the curated dataset used by the DREAMM prediction method [22] is planned as future work to assess concordance between independently derived annotation approaches.

Conclusions

In summary, MPLID provides a large-scale, experimentally grounded resource that fills a critical gap in the membrane protein structural biology landscape. By distinguishing direct lipid contact from membrane zone localization, and by positioning itself as complementary to existing resources including OPM, MemBlob, and BioDolphin/PLIP, the dataset enables a new class of residue-level prediction tasks with direct relevance to drug discovery, protein engineering, and mechanistic understanding of lipid-mediated regulation. Users should interpret MPLID annotations as representing structurally resolved lipid contacts rather than a complete inventory of biological protein-lipid interactions, and should account for the substantial false negative rate inherent to crystallographic and cryo-EM datasets. The dataset adheres to FAIR principles and is freely available under a CC0 public domain dedication. As the PDB continues to grow and cryo-EM structures increasingly resolve lipid molecules at higher resolution, future releases of MPLID will expand both the coverage and the annotation granularity of this resource, including NMR single-model extraction, per-residue lipid type annotations, and expanded lipid ontology coverage.

Potential implications

MPLID is designed to serve as a large-scale training and evaluation resource for multiple research applications in structural bioinformatics, drug discovery, and membrane biology.

Machine learning for lipid contact prediction

The principal application of MPLID is as a training and evaluation dataset for developing machine learning models that predict lipid contact residues from protein sequence or structure. The pre-defined, cluster-aware train/validation/test splits enable standardized model comparison, and the class imbalance (1.00% observed positive rate, approximately 100:1 negative-to-positive ratio) presents a realistic and challenging binary classification task that requires specialized approaches such as class weighting, oversampling (SMOTE) [37], or focal loss functions. Recent advances in geometric deep learning for protein interaction site prediction [38, 39] and end-to-end protein interactome modeling [40] demonstrate the growing potential of graph-based and attention-based architectures for residue-level prediction tasks. While these methods have been developed primarily for protein-protein interaction sites, their hierarchical geometric representations and multiscale attention mechanisms are architecturally suitable for the protein-lipid contact prediction task addressed by MPLID, particularly given the spatial nature of lipid binding sites. The scale of the dataset is sufficient for training deep neural networks, including architectures that combine structural descriptors with protein language model embeddings from ESM-2 [28] or ProtTrans [25]. We note that the Matthews correlation coefficient should be used as the primary evaluation metric for this task due to the substantial class imbalance, as accuracy and even F1 scores can be misleading when the negative class dominates. Users should also be aware that the substantial false negative rate in the non-contact class (see Limitations) may impose an inherent ceiling on achievable performance metrics.

Drug discovery applications

Lipid binding sites on membrane proteins represent an emerging class of druggable targets. General anesthetics, neurosteroids, and endocannabinoids modulate ion channels and GPCRs through specific lipid binding sites [6, 41], and cholesterol binding sites on GPCRs are increasingly recognized as allosteric modulatory sites [8, 42]. This interpretation is consistent with recent work highlighting exosites and allosteric site-forming residues as therapeutically actionable protein-surface regions [43, 44, 45]. MPLID can inform the identification of lipid-mimetic drug binding sites by providing a catalog of experimentally validated lipid interaction residues across thousands of membrane proteins. Predicted lipid contact sites on therapeutically relevant proteins can guide the design of lipid-conjugated prodrugs, membrane-anchored therapeutics, or allosteric modulators that exploit lipid binding pockets.

Structural biology and protein engineering

Understanding which residues contact lipids is essential for rational membrane protein engineering. Mutations at lipid contact sites can destabilize membrane proteins, alter their trafficking, or modify their functional properties. MPLID provides data to train models that predict which residues are critical for lipid-mediated stability, enabling more informed design of stabilizing mutations for membrane protein crystallization, cryo-EM, or functional studies. Additionally, the dataset supports comparative analysis of lipid contact patterns across protein families, enabling identification of conserved lipid binding motifs and family-specific interaction signatures. Future work incorporating cavity-based structural analysis could further support the observation that many residues located within the membrane region do not form direct lipid contacts, reinforcing the conceptual separation between membrane localization and residue-level interaction. These interpretations are based on static structural snapshots. Incorporation of dynamic information may alter residue–lipid proximity patterns, as conformational fluctuations could transiently modify contact definitions.

Integration with protein language models

The residue-level format of MPLID is directly compatible with per-residue embeddings from protein language models. Each residue's 1,280-dimensional ESM-2 embedding [28] or 1,024-dimensional ProtTrans embedding [25] can be concatenated with structural descriptors and used as input to supervised classifiers. The 813 sequence clusters provide sufficient diversity to evaluate whether language model representations generalize across membrane protein families with limited sequence similarity. Alternatively, MPLID can be combined with interpretable structural descriptor frameworks, such as STING RDB-derived nanoenvironment descriptors [20, 21, 43, 44]. In this setting, residue-level lipid contacts may be analyzed in terms of explicit physicochemical and geometric properties, enabling mechanistic interpretation of the nanoenvironment characteristics associated with lipid interaction. This dual compatibility supports both high-capacity representation learning approaches and descriptor-based, interpretable modeling strategies.

Availability of supporting source code and data

The complete MPLID dataset is deposited at Zenodo under a CC0 public domain dedication with persistent DOI: `https://doi.org/10.5281/zenodo.18487584` [46]. Upon acceptance, the dataset will also be deposited in GigaDB in accordance with journal policy. The Zenodo archive contains the full residue-level annotations (train, validation, and test splits as gzip-compressed CSV files), protein-level metadata, amino acid composition statistics, and a data dictionary describing all fields.

All code for reproducing the dataset from raw PDB structures is available in a public GitHub repository: `https://github.com/omagebright/MPLID`. The repository includes the complete processing pipeline (structure downloading, lipid identification, contact calculation, quality filtering, and split generation), analysis scripts for generating figures and tables, and documentation of the data schema and methodology.

The computational workflow is registered in WorkflowHub (`https://workflowhub.eu/workflows/2176`). MPLID is registered in the bio.tools registry (biotoolsID: `MPLID`; `https://bio.tools/MPLID`). MPLID is registered in the SciCrunch Resource Registry as Membrane Protein-Lipid Interaction Database, RRID:`SCR_028506`.

The dataset and code are organized following FAIR principles [47]: data are Findable via persistent DOIs and indexed metadata; Accessible through standard HTTPS download from Zenodo and GitHub; Interoperable using standard CSV format with documented column definitions; and Reusable under permissive open licenses (CC0 for data, MIT for code).

Conflicts of interest

The authors declare that they have no competing interests.

Funding

This research was funded by the São Paulo Research Foundation (FAPESP) (grant nos. 2020/08615-8, 2023/02691-2, 2025/23708-6).

Data availability

The data underlying this article are available in Zenodo at `https://doi.org/10.5281/zenodo.18487584`, and in a public GitHub repository at `https://github.com/omagebright/MPLID`.

Author contributions statement

Author contributions follow the CRediT taxonomy. F.B.O.: Conceptualization, Methodology, Software, Formal Analysis, Data Curation, Writing (Original Draft), Visualization. G.N.: Conceptualization (dataset definition and biological framework), Supervision, Writing (Original Draft, Review & Editing), Funding Acquisition. Both authors read and approved the final manuscript.

Ethics approval and consent to participate

Not applicable. This study uses publicly available structural data from the Protein Data Bank and does not involve human subjects, animal experiments, or clinical data.

Acknowledgments

The authors thank Ivan Mazoni and Inácio Henrique Yano for their contributions to data validation and resource management during the early stages of this work. The authors thank Embrapa Digital Agriculture (Embrapa Agricultura Digital) for computational resources and infrastructure support. The authors acknowledge the RCSB Protein Data Bank [26, 27] and the OPM database [12] teams for maintaining the essential structural biology resources upon which this work depends. The authors thank the developers of MMseqs2 [29], BioPython [34], and the broader open-source scientific computing community for the software tools used in this study.

Abbreviations

MPLID: Membrane Protein-Lipid Interaction Database; **PDB**: Protein Data Bank; **OPM**: Orientations of Proteins in Membranes; **GPCR**: G protein-coupled receptor; **MCC**: Matthews correlation coefficient; **CG-MD**: Coarse-grained molecular dynamics; **FAIR**: Findable, Accessible, Interoperable, Reusable; **CDL**: Cardiolipin; **CLR**: Cholesterol; **PLM**: Palmitic acid; **DREAMM**: Detection of Residues on Exposed Areas of Membrane-associated Macromolecules; **ESM-2**: Evolutionary Scale Modeling 2; **SMOTE**: Synthetic Minority Over-sampling Technique; **PLIP**: Protein-Ligand Interaction Profiler; **RCSB**: Research Collaboratory for Structural Bioinformatics; **NMR**: Nuclear magnetic resonance; **CSV**: Comma-separated values.

References

1. Erik Wallin and Gunnar von Heijne. Genome-wide analysis of integral membrane proteins from eubacterial, archaean, and eukaryotic organisms. *Protein Science*, 7(4):1029–1038, 1998. doi: 10.1002/pro.5560070420.
2. John P Overington, Bissan Al-Lazikani, and Andrew L Hopkins. How many drug targets are there? *Nature Reviews Drug Discovery*, 5(12):993–996, 2006. doi: 10.1038/nrd2199.
3. Rita Santos, Oleg Ursu, Anna Gaulton, et al. A comprehensive map of molecular drug targets. *Nature Reviews Drug Discovery*, 16(1):19–34, 2017. doi: 10.1038/nrd.2016.230.
4. Valentina Corradi, Besian I Sejdiu, Haydee Mesa-Galloso, et al. Emerging diversity in lipid–protein interactions. *Chemical Reviews*, 119(9):5775–5848, 2019. doi: 10.1021/acs.chemrev.8b00451.
5. Arthur Laganowsky, Eamonn Reading, Timothy M Allison, et al. Membrane proteins bind lipids selectively to modulate their structure and function. *Nature*, 510(7503):172–175, 2014. doi: 10.1038/nature13419.
6. Anna L Duncan, Wanling Song, and Mark SP Sansom. Lipid-dependent regulation of ion channels and G protein-coupled receptors: insights from structures and simulations. *Annual Review of Pharmacology and Toxicology*, 60:31–50, 2020. doi: 10.1146/annurev-pharmtox-010919-023411.
7. Anthony G Lee. How lipids affect the activities of integral membrane proteins. *Biochimica et Biophysica Acta - Biomembranes*, 1666(1-2):62–87, 2004. doi: 10.1016/j.bbamem.2004.05.012.
8. Michael A Hanson, Vadim Cherezov, Mark T Griffith, et al. A specific cholesterol binding site is established by the 2.8 å

structure of the human β2-adrenergic receptor. *Structure*, 16(6):897–905, 2008. doi: 10.1016/j.str.2008.05.001.
9. Felix-Xavier Contreras, Andreas M Ernst, Felix Wieland, and Britta Brügger. Specificity of intramembrane protein–lipid interactions. *Cold Spring Harbor Perspectives in Biology*, 3(6):a004705, 2011. doi: 10.1101/cshperspect.a004705.
10. Takeshi Harayama and Howard Riezman. Understanding the diversity of membrane lipid composition. *Nature Reviews Molecular Cell Biology*, 19(5):281–296, 2018. doi: 10.1038/nrm.2017.138.
11. Gerrit van Meer, Dennis R Voelker, and Gerald W Feigenson. Membrane lipids: where they are and how they behave. *Nature Reviews Molecular Cell Biology*, 9(2): 112–124, 2008. doi: 10.1038/nrm2330.
12. Mikhail A Lomize, Irina D Pogozheva, Hyeon Joo, Henry I Mosberg, and Andrei L Lomize. OPM database and PPM web server: resources for positioning of proteins in membranes. *Nucleic Acids Research*, 40(D1):D370–D376, 2012. doi: 10.1093/nar/gkr703.
13. Andrei L Lomize, Irina D Pogozheva, Mikhail A Lomize, and Henry I Mosberg. Positioning of proteins in membranes: a computational approach. *Protein Science*, 15(6):1318–1333, 2006. doi: 10.1110/ps.062126106.
14. Alexios Chatzigoulas and Zoe Cournia. DREAMM: a web-based server for drugging protein-membrane interfaces as a novel workflow for targeted drug design. *Bioinformatics*, 38 (24):5449–5451, 2022. doi: 10.1093/bioinformatics/btac680.
15. Thomas D Newport, Mark SP Sansom, and Phillip J Stansfeld. The MemProtMD database: a resource for membrane-embedded protein structures and their lipid interactions. *Nucleic Acids Research*, 47(D1):D390–D397, 2019. doi: 10.1093/nar/gky1047.
16. Phillip J Stansfeld, Joseph E Goose, Martin Caffrey, et al. MemProtMD: automated insertion of membrane protein structures into explicit lipid membranes. *Structure*, 23(7): 1350–1361, 2015. doi: 10.1016/j.str.2015.05.006.
17. Lukasz P Kozłowski and Janusz M Bujnicki. MemBlob database and server for identifying transmembrane regions using cryo-EM maps. *Bioinformatics*, 35(19):3582–3584, 2019. doi: 10.1093/bioinformatics/btz539.
18. Jorge Roel-Touris, Heidi Koldsoe, and Manuel N. Melo. BioDolphin: an open-source computational platform for the analysis of lipid-protein interactions in the PDB. *Communications Chemistry*, 7:Article 308, 2024. doi: 10.1038/s42004-024-01384-z.
19. Melissa F Adasme, Katja L Linnemann, Sarah Naomi Bolz, et al. PLIP 2021: expanding the scope of the protein–ligand interaction profiler to DNA and RNA. *Nucleic Acids Research*, 49(W1):W530–W534, 2021. doi: 10.1093/nar/gkab294.
20. Goran Neshich, Roberto C Togawa, Adauto L Mancini, Paula R Kuser, Michel EB Yamagishi, Georgios Pappas Jr, Wandré V Torres, Thalita Fonseca e Campo, Leandro L Ferreira, Fabio M Luna, et al. STING Millennium: a web-based suite of programs for comprehensive and simultaneous analysis of protein structure and sequence. *Nucleic Acids Research*, 31(13):3386–3392, 2003. doi: 10.1093/nar/gkg578.
21. Goran Neshich, Ivan Mazoni, Sandro Roberto Oliveira, Michel EB Yamagishi, Paula R Kuser-Falcão, Leandro C Borro, Dag H Morita, Katlin Rabelo Souza, Vagner M Gonçalves-Almeida, et al. STING Report: convenient web-based application for graphic and tabular presentations of protein sequence, structure and function descriptors from the STING database. *Nucleic Acids Research*, 34(suppl_2): W209–W213, 2006. doi: 10.1093/nar/gkl226.
22. Alexios Chatzigoulas and Zoe Cournia. Predicting protein–membrane interfaces of peripheral membrane proteins using ensemble machine learning. *Briefings in Bioinformatics*, 23(2):bbab518, 2022. doi: 10.1093/bib/bbab518.
23. Niek van Hilten, Nino Verwei, Jeroen Methorst, Carsten Nase, Andrius Bernatavicius, and Herre Jelger Risselada. PMIpred: a physics-informed web server for quantitative protein–membrane interaction prediction. *Bioinformatics*, 40(2):btae069, 2024. doi: 10.1093/bioinformatics/btae069.
24. Dimitra Paranou, Alexios Chatzigoulas, and Zoe Cournia. Using deep learning and large protein language models to predict protein–membrane interfaces of peripheral membrane proteins. *Bioinformatics Advances*, 4(1): vbae078, 2024. doi: 10.1093/bioadv/vbae078.
25. Ahmed Elnaggar, Michael Heinzinger, Christian Dallago, et al. ProtTrans: toward understanding the language of life through self-supervised learning. *IEEE Transactions on Pattern Analysis and Machine Intelligence*, 44(10):7112–7127, 2022. doi: 10.1109/TPAMI.2021.3095381.
26. Helen M Berman, John Westbrook, Zukang Feng, et al. The Protein Data Bank. *Nucleic Acids Research*, 28(1): 235–242, 2000. doi: 10.1093/nar/28.1.235.
27. Stephen K Burley, Charmi Bhikadiya, Chunxiao Bi, et al. RCSB Protein Data Bank (rcsb.org): delivery of experimentally-determined PDB structures alongside one million computed structure models of proteins from artificial intelligence/machine learning. *Nucleic Acids Research*, 51(D1):D488–D508, 2023. doi: 10.1093/nar/gkac1077.
28. Zeming Lin, Halil Akin, Roshan Rao, et al. Evolutionary-scale prediction of atomic-level protein structure with a language model. *Science*, 379(6637):1123–1130, 2023. doi: 10.1126/science.ade2574.
29. Martin Steinegger and Johannes Söding. MMseqs2 enables sensitive protein sequence searching for the analysis of massive data sets. *Nature Biotechnology*, 35(11):1026–1028, 2017. doi: 10.1038/nbt.3988.
30. Ian Sillitoe, Nicola Bordin, Natalie Dawson, et al. CATH: increased structural coverage of functional space. *Nucleic Acids Research*, 49(D1):D266–D273, 2021. doi: 10.1093/nar/gkaa1079.
31. Gunnar von Heijne. Membrane protein structure prediction: hydrophobicity analysis and the positive-inside rule. *Journal of Molecular Biology*, 225(2):487–494, 1992. doi: 10.1016/0022-2836(92)90934-C.
32. William P Russ and Donald M Engelman. The GxxxG motif: a framework for transmembrane helix-helix association. *Journal of Molecular Biology*, 296(3):911–919, 2000. doi: 10.1006/jmbi.1999.3489.
33. Anna L Duncan, Jonathan J Ruprecht, Edmund RS Kunji, and Alan J Robinson. Cardiolipin binds selectively but transiently to conserved lysine residues in the rotor of metazoan ATP synthases. *Science Advances*, 7(34): eabh2217, 2021. doi: 10.1126/sciadv.abh2217.
34. Peter JA Cock, Tiago Antao, Jeffrey T Chang, et al. Biopython: freely available Python tools for computational molecular biology and bioinformatics. *Bioinformatics*, 25 (11):1422–1423, 2009. doi: 10.1093/bioinformatics/btp163.
35. Wes McKinney. Data structures for statistical computing in Python. In *Proceedings of the 9th Python in Science Conference*, pages 56–61, 2010. doi: 10.25080/

Majora-92bf1922-00a.
36. Fabian Pedregosa, Gaël Varoquaux, Alexandre Gramfort, et al. Scikit-learn: machine learning in Python. *Journal of Machine Learning Research*, 12:2825–2830, 2011.
37. Nitesh V Chawla, Kevin W Bowyer, Lawrence O Hall, and W Philip Kegelmeyer. SMOTE: synthetic minority over-sampling technique. *Journal of Artificial Intelligence Research*, 16:321–357, 2002. doi: 10.1613/jair.953.
38. Yong Han, Shao-Wu Zhang, Qing-Qing Zhang, and Ming-Hui Shi. MGMA-PPIS: Predicting the protein-protein interaction site with multiview graph embedding and multiscale attention fusion. *GigaScience*, 14:giaf114, 2025. doi: 10.1093/gigascience/giaf114.
39. Shizhuo Zhang, Jiyun Han, and Juntao Liu. Protein-protein and protein-nucleic acid binding site prediction via interpretable hierarchical geometric deep learning. *GigaScience*, 13:giae080, 2024. doi: 10.1093/gigascience/giae080.
40. Yu-Hsin Chen, Chien-Fu Liu, Jun-Yi Leu, and Huai-Kuang Tsai. Complete end-to-end learning from protein feature representation to protein interactome inference. *GigaScience*, 14:giaf122, 2025. doi: 10.1093/gigascience/giaf122.
41. Grace Brannigan, Jérôme Hénin, Richard Law, et al. Embedded cholesterol in the nicotinic acetylcholine receptor. *Proceedings of the National Academy of Sciences*, 105(38):14418–14423, 2008. doi: 10.1073/pnas.0803029105.
42. Jacques Fantini and Francisco J Barrantes. How cholesterol interacts with membrane proteins: an exploration of cholesterol-binding sites including CRAC, CARC, and tilted domains. *Frontiers in Physiology*, 4:31, 2013. doi: 10.3389/fphys.2013.00031.
43. Folorunsho Bright Omage, Jorge A. Salim, Ivan Mazoni, Inácio Henrique Yano, Leandro Borro, Juan E. H. Gonzalez, Monica Pickholz, I. A. P. Neshich, I. P. Sousa, K. Santana, A. L. Rochetti, J. Fernandes, J. G. Jardine, G. T. Valente, A. L. Mancini, L. Tasic, R. K. Arni, R. Parise Filho, and Goran Neshich. Protein allosteric site identification using machine learning. *Computational and Structural Biotechnology Journal*, 23:3907–3919, 2024. doi: 10.1016/j.csbj.2024.10.036.
44. Folorunsho Bright Omage, Jorge A. Salim, Ivan Mazoni, Inácio Henrique Yano, Juan E. H. González, I. A. P. Neshich, Leandro C. Borro, Monica Pickholz, J. Fernandes, J. G. Jardine, A. L. Mancini, R. K. Arni, L. Tasic, and Goran Neshich. STINGAllo: a web server for high-throughput prediction of allosteric site-forming residues. *Briefings in Bioinformatics*, 26(4):bbaf424, 2025. doi: 10.1093/bib/bbaf424.
45. Folorunsho Bright Omage, Ivan Mazoni, Inácio Henrique Yano, and Goran Neshich. Protein exosites as drug targets: from structural topology to therapeutic modality. *Drug Discovery Today*, 31(3):104662, 2026. doi: 10.1016/j.drudis.2026.104662.
46. Folorunsho Bright Omage and Goran Neshich. MPLID: Membrane protein-lipid interface dataset, 2026. URL `https://doi.org/10.5281/zenodo.18487584`. Version 2.0.0.
47. Mark D Wilkinson, Michel Dumontier, IJsbrand Jan Aalbersberg, et al. The FAIR guiding principles for scientific data management and stewardship. *Scientific Data*, 3(1):160018, 2016. doi: 10.1038/sdata.2016.18.

Tracked changes manuscript

Click here to access/download;Manuscript;main_gigascience_tracked.pdf

GigaScience, 2026, pp. 1–16

doi:

Data Note

DATA NOTE

MPLID (Membrane Protein–Lipid Interaction Database): A Large-Scale Experimental Resource of Residue-Level Protein–Lipid Contacts

Folorunsho Bright Omage[1,2,3,*] and Goran Neshich[2,*]

[1]Department of Computer Science, University of Oxford, Wolfson Building, Parks Road, OX1 3QD, Oxford, United Kingdom, [2]Computational Biology Research Group, Embrapa Digital Agriculture, Av. André Tosello 209, 13083-886, Campinas, São Paulo, Brazil and [3]Biological Chemistry Laboratory, Department of Organic Chemistry, Institute of Chemistry, University of Campinas (UNICAMP), R. Monteiro Lobato 270, 13083-970, Campinas, São Paulo, Brazil

*Corresponding authors. folorunsho.bright.omage@cs.ox.ac.uk; goran.neshich@embrapa.br

Abstract

Background: Membrane proteins constitute approximately 20–30% of all proteomes and represent over 60% of current drug targets. Although protein–lipid interactions play important structural and regulatory roles in membrane-associated proteins, most existing structural resources focus on identifying whether a residue lies within a membrane region, typically inferred from computational hydrophobicity-based positioning algorithms. This approach does not directly address a distinct biological question: which residues at the protein surface make direct physical contact with lipid molecules? Answering this question from experimental data is critical for understanding lipid-mediated allostery, designing lipid-mimetic therapeutics, and training accurate machine learning models for lipid binding site prediction. **Findings:** We present MPLID (Membrane Protein-Lipid Interaction Database), a curated residue-level dataset comprising 4,704 membrane proteins representing 813 sequence clusters at 30% identity, 8,055,325 residues, and 80,439 experimentally validated lipid contact annotations (1.00% observed positive rate). Labels are derived exclusively from crystallized lipid molecules resolved in Protein Data Bank structures using a 4.0 Å all-atom heavy-atom distance cutoff. Because most native lipid interactions are lost during purification and crystallization, this observed rate represents a lower bound, and the non-contact class inevitably contains false negatives. The dataset recognizes 117 lipid codes across ten functional categories (cross-validated against the RCSB Chemical Component Dictionary), spanning phospholipids, cardiolipin, sphingolipids, sterols, fatty acids, glycerolipids, detergent mimetics (explicitly flagged), lipid A components, and CHARMM simulation nomenclature. To prevent data leakage, proteins are clustered at 30% sequence identity using MMseqs2, yielding 813 clusters partitioned into training (2,578), validation (1,051), and test (1,075) splits. Amino acid composition analysis reveals biologically consistent enrichment at lipid contact sites: tryptophan (1.88×), arginine (1.44×), glycine (1.36×), lysine (1.33×), and phenylalanine (1.23×) are enriched, while proline (0.51×), isoleucine (0.57×), and aspartate (0.59×) are depleted. **Conclusions:** MPLID addresses a distinct biological question compared to existing resources (OPM, MemBlob, BioDolphin/PLIP): identifying residues that directly contact experimentally resolved lipid molecules rather than those positioned within computationally defined membrane boundaries. With 4,704 proteins and over 8 million annotated residues, MPLID provides the scale needed for training deep learning models for lipid contact prediction, with direct applications in structure-guided drug design and membrane protein engineering. The dataset adheres to FAIR principles and is freely available under a CC0 public domain dedication. Structurally resolved contacts represent only a subset of the full biological lipid interaction landscape, and MPLID is intended as a ground-truth resource rather than a complete catalog of lipid binding sites.

Key words: membrane proteins, lipid-protein interactions, protein structure, machine learning dataset, structural biology, experimental validation, residue-level classification, FAIR data

Background

Membrane proteins are central to virtually all cellular processes, mediating transport of ions and solutes, signal transduction, energy conversion, and cell adhesion [1]. Genomic analyses consistently estimate that 20–30% of open reading frames in any given proteome encode membrane proteins [1], and pharmacological surveys have established that these proteins account for more than 60% of all current drug targets [2, 3]. Despite their biomedical importance, membrane proteins remain among the most challenging targets for structural

© The Author 2026. Published by Oxford University Press. All rights reserved. For permissions, please e-mail: journals.permissions@oup.com

and computational biology, in large part because their native functional context, the lipid bilayer, is difficult to recapitulate experimentally and model computationally.

A growing body of evidence demonstrates that lipid-protein interactions are not passive consequences of membrane embedding but rather active modulators of protein structure, dynamics, and function [4, 5]. Specific lipid binding events regulate ion channel gating [6], G protein-coupled receptor (GPCR) signaling [4], and transporter conformational cycling [7]. Cholesterol, for example, occupies defined binding sites on GPCRs that are structurally resolved at atomic resolution [8], and cardiolipin binding is essential for the function of mitochondrial respiratory chain complexes [9]. The diversity of lipid species in biological membranes, encompassing phospholipids, sphingolipids, sterols, and glycolipids [10, 11], creates a complex interaction landscape that shapes the behavior of membrane-embedded proteins.

Membrane zone versus lipid contact: two distinct questions

A fundamental distinction exists between two related but different biological questions in membrane protein structural biology. The first question, "Is this residue located within the membrane zone?", has been addressed by computational databases such as the Orientations of Proteins in Membranes (OPM) database [12, 13]. OPM positions membrane proteins in a hydrophobic slab model using energy minimization against experimentally calibrated transfer free energy parameters, producing membrane boundaries for over 15,000 protein entries. This approach is invaluable for understanding membrane protein topology, but the resulting labels reflect a coarse spatial localization: typically 20–40% of residues in a transmembrane protein fall within the OPM-defined membrane zone.

The second question, "Does this residue make direct physical contact with a lipid molecule?", requires experimental structural evidence. Crystallographic and cryo-electron microscopy structures frequently resolve lipid molecules at specific protein surface sites, revealing native lipid interaction partners at atomic resolution. These experimental contacts represent a far more stringent and specific annotation: in our dataset, only 1.00% of all residues in membrane proteins (including both surface-exposed and buried residues) are observed to make lipid contacts within 4.0 Å (using an all-atom distance criterion), a rate . However, this rate represents a lower bound on the true frequency of lipid contacts in biological membranes, because only a subset of native lipid interactions survive protein purification and are resolved in crystal or cryo-EM structures. Nonetheless, even as a lower bound, this rate is approximately 20- to 40-fold lower than the membrane zone positive rate, underscoring the specificity of the experimental lipid contact annotation.

This distinction has profound consequences for both biological interpretation and machine learning model development (Table 1). Membrane zone labels identify the general region of a protein that is embedded in the bilayer, while lipid contact labels pinpoint specific binding sites that may mediate functionally important lipid-protein interactions. For drug discovery, the latter is far more actionable: lipid contact sites represent potential targets for allosteric modulation by lipid-mimetic compounds [14], while membrane zone annotations provide no such site-level resolution. We emphasize that no OPM-derived residue labels were used at any stage of the MPLID construction pipeline; all residue annotations derive exclusively from experimentally resolved lipid molecules in PDB structures. OPM is discussed here solely to distinguish the complementary biological questions of membrane positioning versus direct lipid contact.

Table 1. Comparison of membrane zone and lipid contact prediction tasks. These represent fundamentally different biological questions with distinct label sources, positive rates, and downstream applications.

Aspect	Membrane zone	Lipid contact
Label source	Computational (OPM)	Experimental (PDB)
Biological question	In membrane slab?	Contacts lipid?
Positive Observed positive rate	20–40%	∼1.00%[†]
Spatial resolution	Zone-level (∼30 Å)	Atomic (4.0 Å)
Validation	In silico energy	X-ray/cryo-EM
Key application	Topology prediction	Binding site identification

[†]Lower bound; the true lipid contact rate in vivo is likely substantially higher due to lipid loss during purification and incomplete resolution.

Existing resources and their limitations

Several resources provide information about membrane protein-lipid interactions, each with specific scope and limitations. The OPM database [12] remains the definitive resource for membrane protein positioning, providing computationally derived membrane boundaries for transmembrane and peripheral proteins. However, OPM labels represent theoretical membrane zones rather than experimentally resolved lipid interaction sites.

The MemProtMD database [15, 16] complements OPM by providing coarse-grained molecular dynamics (CG-MD) simulations of membrane proteins embedded in explicit phospholipid bilayers. While MemProtMD offers valuable insights into lipid organization around proteins, its contacts derive from simulations rather than experimental structures, and the database focuses on integral membrane proteins in a single lipid type (dipalmitoylphosphatidylcholine).

The MemBlob database and server [17] takes a complementary approach by analyzing cryo-EM density maps to computationally identify membrane-facing regions of transmembrane proteins. MemBlob classifies residues as solvent-facing, lipid-facing, at the lipid interface, or buried, providing a membrane environment annotation that bridges between the coarse OPM slab model and direct experimental contact data. While MemBlob provides valuable residue-level environment classification, its labels are derived from density map analysis rather than from resolved lipid molecules, and the database is limited to proteins with available cryo-EM reconstructions.

BioDolphin [18] identifies and catalogs all protein-lipid interactions across the PDB using the PLIP (Protein-Ligand Interaction Profiler) framework [19], providing detailed interaction-type annotations (hydrogen bonds, hydrophobic contacts, salt bridges) at the residue and atom level. BioDolphin offers API access and covers a broad range of interaction types, making it a powerful tool for detailed mechanistic analysis of individual protein-lipid complexes. MPLID differs from BioDolphin in its focus on providing pre-computed, ML-ready binary contact labels with

cluster-aware train/validation/test splits, continuous distance values, and FAIR-compliant dataset deposition, rather than on-demand interaction profiling.

The STING Relational DataBase (RDB) [20, 21] provides a comprehensive structural analysis platform that computes and stores inter-atomic contacts, surface accessibility, and residue-level descriptors for all PDB structures. While STING RDB does not specifically label lipid contacts, its contact calculation infrastructure and residue-level annotation framework informed the design philosophy of MPLID, particularly regarding the use of distance-based contact definitions and the importance of providing both binary labels and continuous distance values for downstream analysis.

On the prediction methodology side, the DREAMM method [22] demonstrated that ensemble machine learning can achieve a Matthews correlation coefficient (MCC) of 0.84 for predicting protein-membrane interfaces, using a curated set of 54 peripheral membrane proteins (later expanded to 65) with experimentally characterized membrane-penetrating residues. However, this training data was distributed as part of the prediction tool rather than as a standalone, documented dataset resource. More recently, PMIpred introduced a physics-informed transformer approach trained on molecular dynamics data for peptide-membrane interactions [23], and protein language model approaches have been explored using ProtTrans embeddings [24, 25]. While these studies have advanced prediction methodology, none provides a large-scale, publicly deposited, and documented dataset covering the full diversity of membrane protein classes.

The need for large-scale experimental lipid contact data

The convergence of three trends motivates the creation of MPLID. First, the rapid growth of the Protein Data Bank [26, 27], which now contains more than 230,000 structures, means that thousands of membrane protein structures with resolved lipid molecules are available for systematic analysis. Second, advances in protein language models such as ESM-2 [28] and ProtTrans [25] have demonstrated that per-residue embeddings trained on large-scale protein sequence data can capture structural and functional properties, but training supervised models on top of these representations requires large labeled datasets. Third, structure-guided drug discovery increasingly recognizes lipid binding sites as druggable targets for allosteric modulation [14, 6], creating demand for systematic catalogs of experimentally validated lipid interaction sites.

MPLID addresses these needs by providing experimentally validated lipid contact annotations for 4,704 membrane proteins containing 8,055,325 residues. All labels derive from crystallized lipid molecules in PDB structures, ~~ensuring~~ providing experimental ground truth at atomic resolution. ~~Unlike prior resources that are limited in scale or scope, MPLID covers~~ While existing resources offer related information, including membrane environment classification from cryo-EM density (MemBlob [17]) and detailed interaction-type profiling (BioDolphin/PLIP [18, 19]), none provides a pre-computed, ML-ready residue-level dataset with cluster-aware evaluation splits and FAIR-compliant deposition. MPLID fills this gap by covering integral, peripheral, and single-pass membrane proteins across 813 sequence-diverse clusters.

Data Description

Dataset overview

MPLID provides residue-level binary annotations indicating whether each residue in a membrane protein makes direct physical contact with ~~a crystallized~~ an experimentally resolved lipid molecule. The complete dataset encompasses 4,704 membrane proteins with a total of 8,055,325 residues, of which 80,439 (1.00%) are labeled as lipid contacts (Figure 1). This ~~positive rate reflects the biological reality that~~ observed positive rate should be interpreted as a lower bound on the true frequency of protein-lipid contacts in biological membranes: many native lipid interactions are lost during protein purification and crystallization, and most PDB structures resolve only a small ~~fraction of residues in membrane proteins make direct lipid contacts within 4.0 Å, primarily at protein surfaces, interfacial regions, and lipid-binding pockets~~number of tightly bound or structurally ordered lipids. Consequently, the non-contact class (99.00%) inevitably contains a substantial proportion of false negatives, residues that contact lipids in vivo but lack a resolved lipid partner in the crystal or cryo-EM structure. The remaining residues ~~are located in~~ classified as non-contacts include solvent-exposed domains, protein-protein interfaces, ~~or~~ interior regions of multi-subunit complexes, and an unknown fraction of unresolved lipid-binding sites.

Proteins in the dataset range from 22 to 15,280 residues (median 1,038; mean 1,712), reflecting the diversity of membrane protein sizes from small single-pass transmembrane peptides to large multi-subunit complexes. The number of lipid contacts per protein ranges from 1 to 4,820 (median 4; mean 17), with the highly skewed distribution reflecting that most proteins have few resolved lipid contacts while a small number of well-characterized complexes have extensive lipid interactions. The maximum value (4,820 contacts for PDB 6W4F, an NMR-resolved structure of the KRAS4B homodimer on a lipid bilayer nanodisc) arises because the current pipeline counts residue-lipid contacts across all NMR conformational models deposited in the PDB coordinate file; 6W4F contains 20 NMR models, each with approximately 240 contact residues. A total of 41 NMR multi-model structures in the dataset are affected by this counting convention, contributing approximately 24,000 excess contact annotations (see Limitations). Every protein in the final dataset has at least one lipid contact, as proteins with zero contacts were removed during quality filtering (see Methods). This removal excludes membrane proteins whose lipids were stripped during purification, meaning that MPLID does not represent the complete membrane proteome and cannot serve as a source of proteome-wide negative controls.

Data schema and format

Each record in MPLID contains ten fields that provide complete information for each annotated residue. The `pdb_id` field contains the four-character PDB accession code, and `chain_id` specifies the polypeptide chain within the structure. The `residue_number` gives the residue's position in the chain, while `residue_name` provides the three-letter amino acid code. The binary label `is_contact` takes a value of 1 for residues where any heavy atom is within 4.0 Å of any lipid heavy atom and 0 otherwise. The `label_source` field is set to "EXPERIMENTAL" for all entries, confirming that labels derive from experimentally resolved lipid molecules in PDB structures rather than computational predictions. The `confidence` field is set to "high"

for all entries in the current release because all labels derive from experimentally resolved structures; this field is retained as a placeholder for future quality scoring that may incorporate structure resolution and B-factor data. The `min_distance` field records the minimum distance in Angstroms between any heavy atom of the residue and the nearest lipid heavy atom. Finally, `cluster_id` assigns each protein to a sequence cluster, and `split` indicates assignment to training, validation, or test partitions.

Data are distributed as gzip-compressed comma-separated values (CSV) files partitioned by split (train, validation, test), along with a protein-level metadata file containing per-protein summary statistics including residue counts, contact counts, contact rates, cluster assignments, and split assignments.

Train/validation/test splits

The dataset is partitioned into training (2,578 proteins; 4,907,696 residues), validation (1,051 proteins; 1,403,838 residues), and test (1,075 proteins; 1,743,791 residues) splits (Figure 1). Critically, splitting is performed at the protein level with cluster-aware stratification: proteins within the same sequence cluster are always assigned to the same split, preventing data leakage from sequence-similar proteins appearing in both training and evaluation partitions. Contact rates vary across splits (training 1.16%, validation 0.69%, test 0.79%) because stratification is performed at the cluster level rather than the residue level, and clusters with higher contact rates may be unevenly distributed. This variation is expected and should be accounted for when comparing model performance across splits. This design choice is essential for meaningful generalization assessment, as protein-level leakage can dramatically inflate performance estimates in structural bioinformatics tasks.

The cluster-aware splitting strategy uses MMseqs2 [29] with a 30% sequence identity threshold and 80% alignment coverage, producing 813 clusters. Entire clusters are assigned to splits to ensure that no protein in the validation or test sets shares more than 30% sequence identity with any training protein. This threshold is substantially more conservative than the 40% identity commonly used in protein function prediction benchmarks [30], further reducing the risk of overestimating generalization performance.

Sequence clustering analysis

The 813 sequence clusters exhibit a highly skewed size distribution (Figure 2), with 373 singletons (45.9%) and a largest cluster of 173 proteins, reflecting the sequence diversity of membrane proteins across different families and superfamilies. This clustering pattern indicates substantial structural and functional diversity in the dataset, as singleton clusters represent unique membrane protein families without close homologs.

Analyses

Amino acid composition at lipid contact sites

Analysis of amino acid enrichment at lipid contact sites reveals biologically meaningful and interpretable patterns (Figure 3). Enrichment ratios were calculated as the fraction of each amino acid among contact residues divided by its fraction in the complete dataset. Tryptophan (1.88×) shows the strongest enrichment, consistent with the well-established role of aromatic residues in anchoring transmembrane proteins at the membrane-water interface through favorable interactions with lipid headgroups via cation-π and hydrogen bonding interactions [4]. Notably, positively charged residues are strongly enriched: arginine (1.44×) and lysine (1.33×) rank among the top five most enriched amino acids, consistent with the "positive-inside rule" [31] and with the electrostatic attraction of cationic side chains to anionic phospholipid headgroups. Glycine (1.36×) is enriched, reflecting its prevalence in GxxxG motifs that mediate transmembrane helix-helix packing and create lipid-accessible surfaces at helix interfaces [32]. Phenylalanine (1.23×) and tyrosine (1.08×) are moderately enriched, contributing to the aromatic belt. Among other residues, cysteine (1.15×), serine (1.13×), and histidine (1.11×) show mild enrichment, with histidine functioning as an interfacial anchoring residue through its amphipathic imidazole ring, and cysteine enrichment likely reflecting palmitoylation sites and disulfide-constrained loop regions at lipid interfaces. Leucine (1.08×) is slightly enriched, reflecting its involvement in direct side-chain contacts with lipid acyl chains.

Among depleted residues, proline (0.51×) and isoleucine (0.57×) show the strongest depletion. Proline disrupts regular secondary structure and is underrepresented at smooth lipid-protein interfaces. The depletion of isoleucine, a β-branched hydrophobic residue, may reflect its preference for buried core positions rather than surface lipid contacts. Aspartate (0.59×) is depleted, consistent with the electrostatic incompatibility of negatively charged side chains with the lipid bilayer interior. Asparagine (0.71×) and valine (0.73×) are moderately depleted. Threonine (0.87×) and glutamine (0.89×) show mild depletion, while alanine (0.94×) and glutamate (0.97×) are near neutral. Overall, the enrichment pattern strongly supports the biological validity of the all-atom contact definition: residues capable of electrostatic interactions with phospholipid headgroups (arginine, lysine) and aromatic anchoring at the interface (tryptophan, phenylalanine) are enriched, while conformationally constrained (proline) and β-branched (isoleucine, valine) residues are depleted. These patterns are consistent with previous analyses of cardiolipin binding site composition [33] and likely reflect, in part, the overrepresentation of anionic lipids (particularly cardiolipin and phosphatidylglycerol) among structurally resolved lipid species. Because anionic lipids are more readily co-purified and crystallized with membrane proteins than zwitterionic species such as phosphatidylcholine and phosphatidylethanolamine, the observed enrichment of positively charged residues may be amplified relative to the in vivo membrane environment, where the majority of lipids are zwitterionic. Given the large sample sizes (80,439 contact residues among 8,055,325 total residues), these enrichment and depletion patterns are robust and unlikely to arise from sampling variation, but their magnitude may be influenced by the lipid composition bias inherent to crystallographic datasets.

Distance distribution analysis

The distribution of minimum distances between protein residues and lipid molecules provides independent validation of the 4.0 Å contact cutoff (Figure 4). Among contact residues, the distance distribution shows a clear peak at approximately 3.5–4.0 Å, corresponding to typical van der Waals contact distances between heavy atoms. The sharp boundary at 4.0 Å indicates that the cutoff effectively captures the transition from direct physical contact to non-contact. For non-contact residues, the distribution is broadly shifted toward larger distances, with a

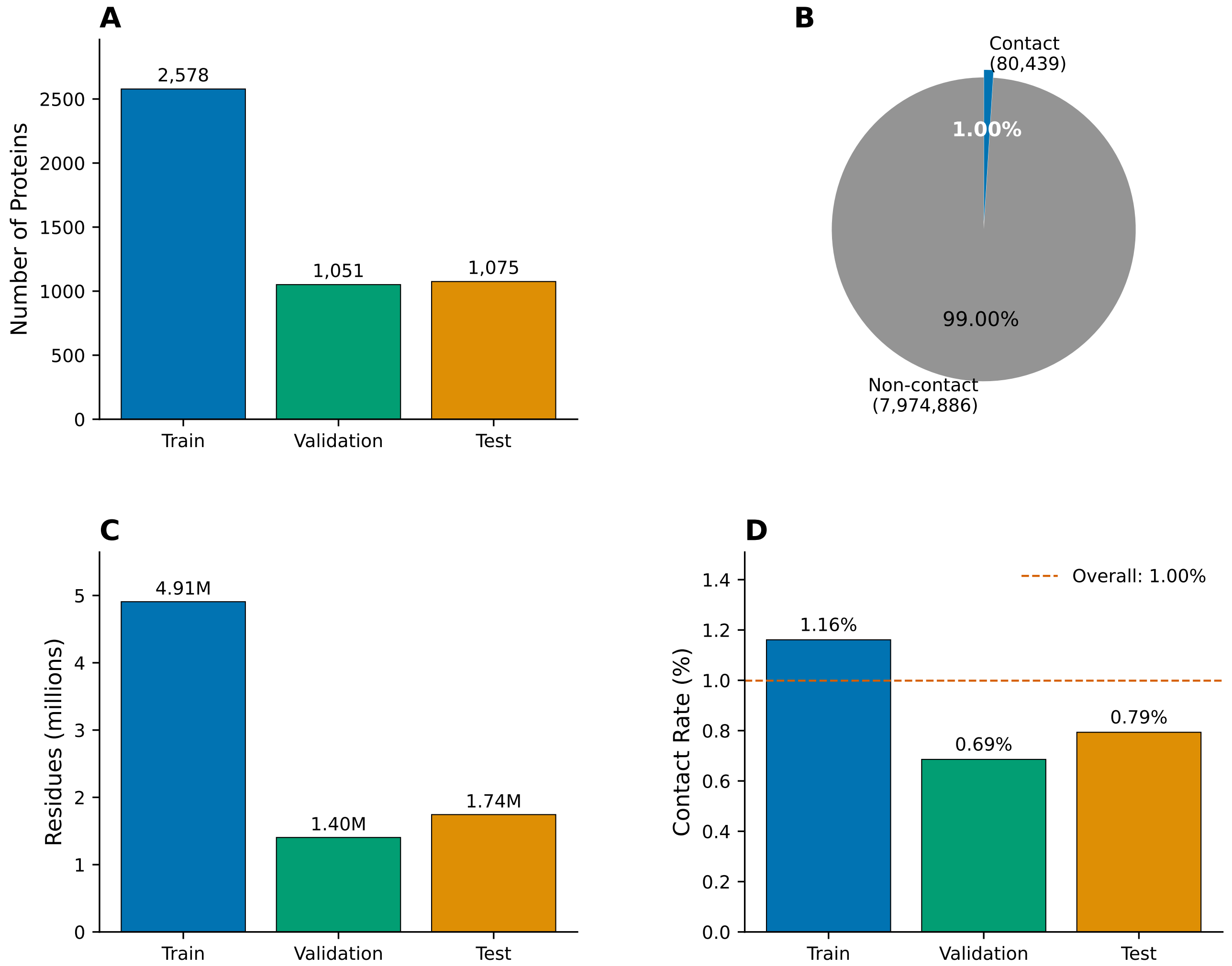

Fig. 1. MPLID dataset overview. (A) Distribution of proteins across training (54.8%), validation (22.3%), and test (22.9%) splits. (B) Class distribution showing the imbalance between contact (1.00%) and non-contact (99.00%) residues. (C) Total residues per split. (D) Contact rates across splits, showing variation due to cluster-level stratification: training (1.16%), validation (0.69%), and test (0.79%).

peak near 8–12 Å and a long tail extending to distances greater than 30 Å for residues in soluble domains far from any lipid molecule.

Protein size and contact density distributions

The distribution of protein sizes in MPLID spans three orders of magnitude, reflecting the diversity of membrane protein families represented (Figure 5). Contact density, defined as the fraction of residues making lipid contacts, varies substantially across proteins, with a per-protein median contact rate of 0.45% and a mean of 0.99%. The ~~overall~~ observed contact rate of 1.00% reflects ~~a~~ both the sparse resolution of lipid molecules in PDB structures and the inherent specificity of direct van der Waals contacts. This rate should be interpreted as a lower bound; the severe class imbalance (approximately 100:1 negative-to-positive ratio) ~~inherent to this prediction task, which~~ includes a substantial fraction of false negatives in the non-contact class, where residues that contact lipids in vivo lack a resolved lipid partner in the experimental structure. This imbalance requires specialized machine learning approaches such as class weighting or focal loss.

Comparison with existing resources

Table 2 places MPLID in the context of existing membrane protein-lipid interaction resources (Figure 6). Each resource addresses a different aspect of the problem. OPM [12] provides computationally derived membrane zone boundaries for topology analysis. MemProtMD [15] offers coarse-grained simulation data for lipid organization. The DREAMM prediction method [22] used a curated set of 54 peripheral membrane proteins with experimentally characterized membrane-penetrating residues as training data, though this data was not released as a standalone documented dataset. MPLID fills this gap by providing the first large-scale, publicly deposited, and fully documented dataset of experimental lipid contact labels, covering 4,704 proteins across all membrane protein classes with FAIR-compliant metadata, a data dictionary, and pre-defined evaluation splits. The diversity of membrane protein families represented in MPLID, spanning 813 sequence clusters at 30% identity, ensures broad coverage of the membrane protein structural landscape. These resources are complementary rather than competing: researchers should select the appropriate resource based on their specific biological

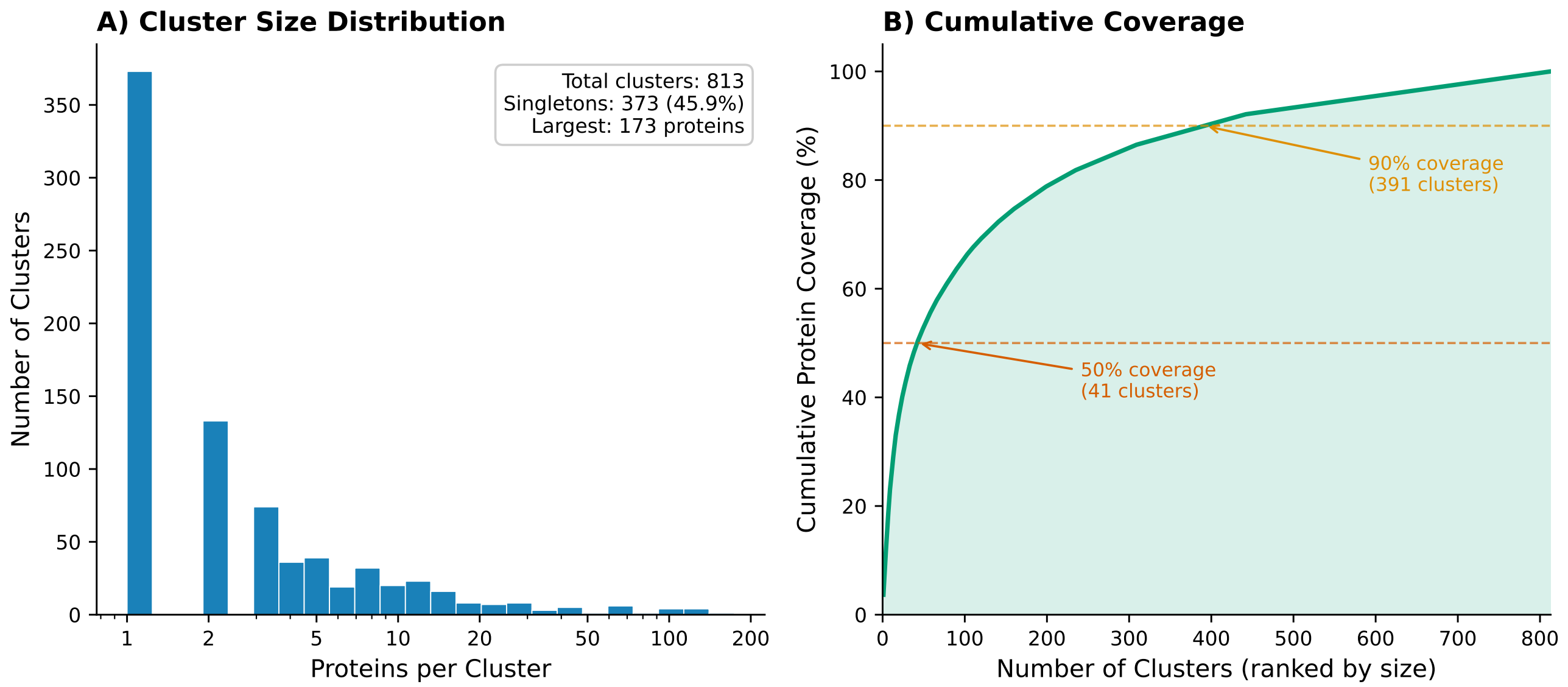

Fig. 2. Sequence cluster analysis. (A) Distribution of cluster sizes at 30% sequence identity on a log scale, showing a highly skewed size distribution with many singletons and a small number of large clusters containing many homologous structures. (B) Cumulative protein coverage ranked by cluster size, illustrating the concentration of proteins in a few large families.

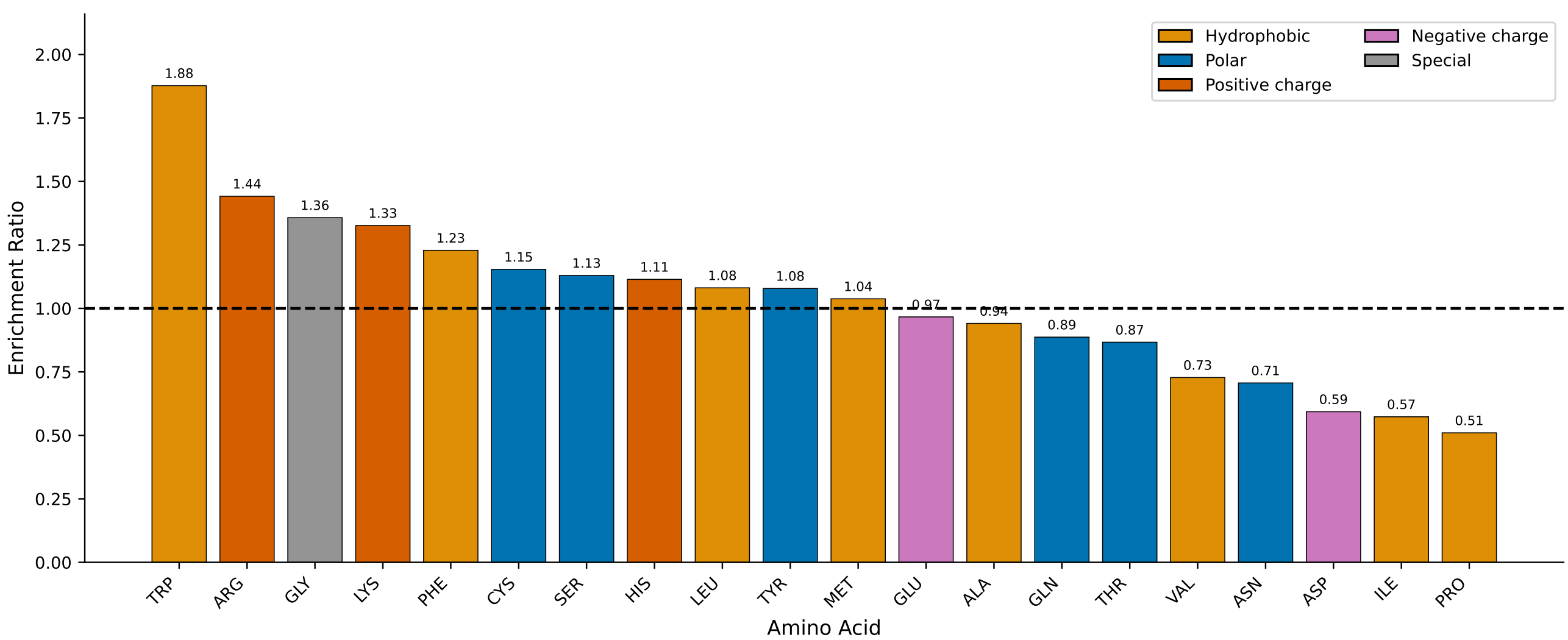

Fig. 3. Amino acid enrichment at lipid contact sites. Enrichment ratios are calculated as the frequency of each amino acid among contact residues divided by its overall frequency in the dataset. Colors indicate amino acid physicochemical properties: hydrophobic (orange), polar (blue), positive charge (red), negative charge (purple), and special (gray). The dashed line marks the expected frequency (1.0). Values above 1.0 indicate enrichment; values below 1.0 indicate depletion.

question, and models trained on one type of label may not transfer directly to another without domain adaptation.

~~MPLID and OPM address different biological questions~~ These resources form a complementary hierarchy addressing different aspects of membrane protein-lipid biology, and should be viewed as complementary rather than competing~~resources.~~ ~~OPM~~ : OPM [12] provides membrane localization and hydrophobic slab positioning; MemBlob [17] infers membrane-facing environment from cryo-EM density; BioDolphin/PLIP [18, 19] provides detailed interaction-type profiling across all PDB ligands; and MPLID provides ~~membrane zone annotations that are useful for topology prediction and membrane protein classification, while MPLID provides lipid contact annotations that are useful for binding site identification and lipid-mediated function analysis~~pre-computed, ML-ready residue-level lipid contact labels with evaluation splits. Models trained on one type of label cannot be directly evaluated on ~~the other~~another, and researchers should select the appropriate ~~dataset~~ resource based on their specific biological question.

Lipid type diversity

MPLID recognizes 117 lipid codes organized into ten functional categories, comprising 90 PDB Chemical Component Dictionary codes and 27 CHARMM simulation lipid identifiers commonly found in cryo-EM depositions. All 90 PDB codes

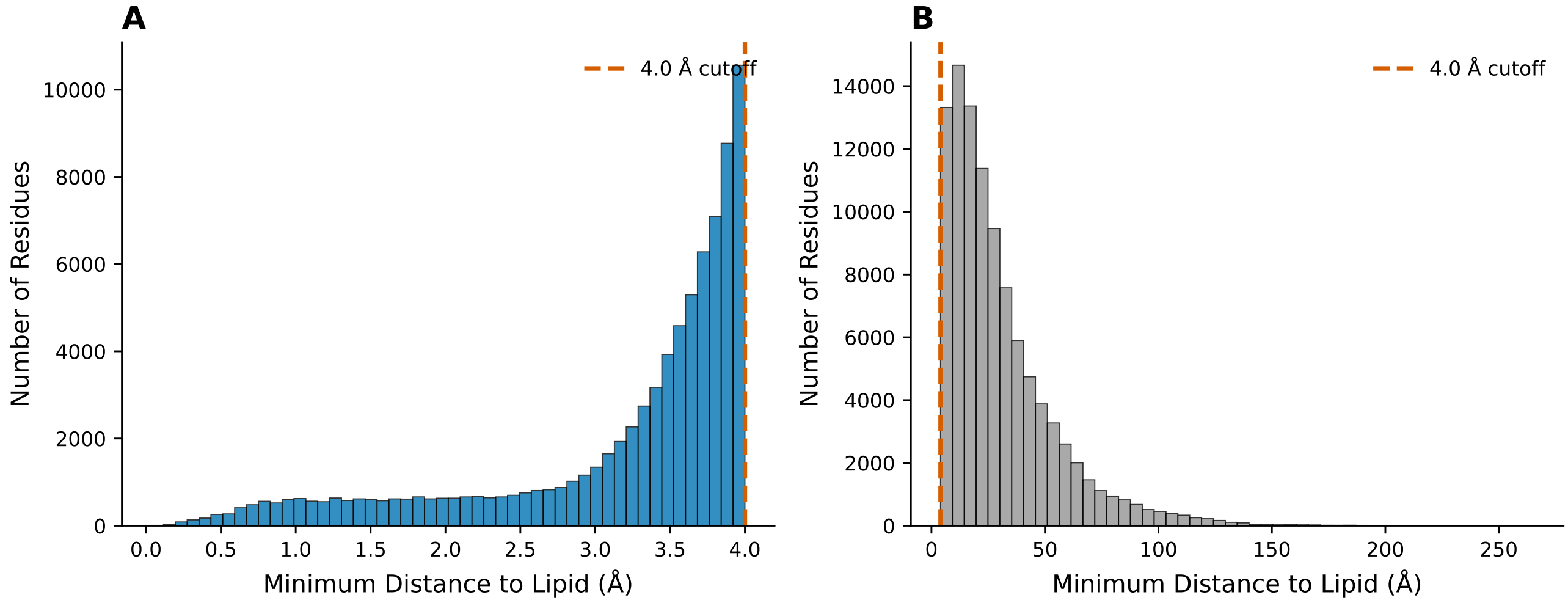

Fig. 4. Distribution of minimum residue-to-lipid distances. (A) Contact residues (≤4.0 Å) show a peak near 3.5–4.0 Å, consistent with van der Waals contact distances. (B) Non-contact residues display a broad distribution with distances extending well beyond 30 Å, confirming clear separation between contacting and non-contacting populations.

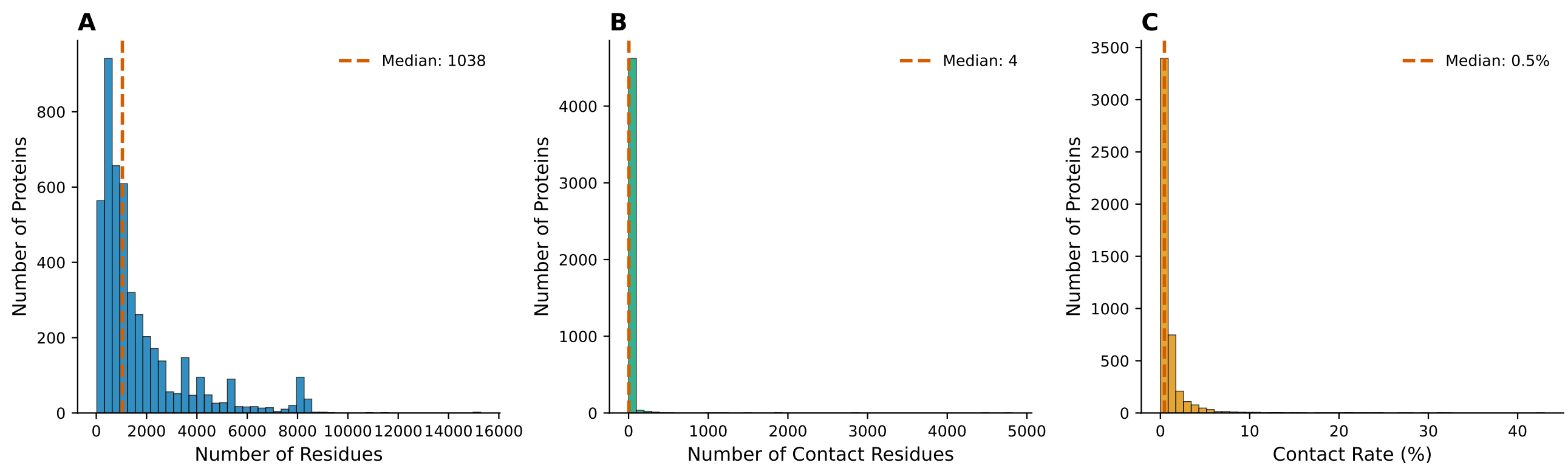

Fig. 5. Protein-level statistics. Distribution of protein sizes and contact densities across the MPLID dataset, illustrating the diversity of membrane protein families represented.

were cross-validated against the RCSB Chemical Component Dictionary API during revision; the complete lipid ontology with RCSB verification status, official chemical names, and category assignments is provided in Supplementary Table S1. Phospholipids include cardiolipin (CDL, found in 675 structures), phosphocholine variants (PCW, 196 structures), phosphoethanolamine (PEE, 273 structures), and phosphoglycerol (PGV, 144 structures). Sterols are represented by cholesterol (CLR, 1,119 structures) and derivatives including cholesteryl hemisuccinate cholic acid (CHD, 151 structures; a bile acid sterol derivative) and cholesterol hemisuccinate (Y01). Fatty acids include palmitate (PLM, 734 structures), oleic acid (OLA, 379 structures), myristate (MYR, 426 structures), and stearate (STE, 94 structures). Sphingolipids

Table 2. Comparison of MPLID with existing membrane protein-lipid interaction resources. MPLID is the first large-scale, publicly deposited dataset providing experimentally validated residue-level lipid contact labels with FAIR-compliant documentation.

Resource	Proteins	Residues	Label type	Label source
MPLID (this work)	4,704	8,055,325	Lipid contact	Experimental (PDB)
BioDolphin [18][b]	PDB-wide	–	Interaction type	Experimental (PLIP)
MemBlob [17][c]	∼500	–	Membrane environment	Cryo-EM density
DREAMM [22][a]	54	∼15,000	Membrane interface	Experimental (curated)
OPM [12]	15,096	–	Membrane zone	Computational
MemProtMD [15]	∼3,500	–	Lipid contact	Simulated (CG-MD)

[a]Training data distributed within the DREAMM prediction tool repository; not released as a standalone documented dataset. [b]On-demand interaction profiling via PLIP; not pre-computed ML-ready dataset with splits. [c]Residue-level membrane environment classification from cryo-EM density maps.

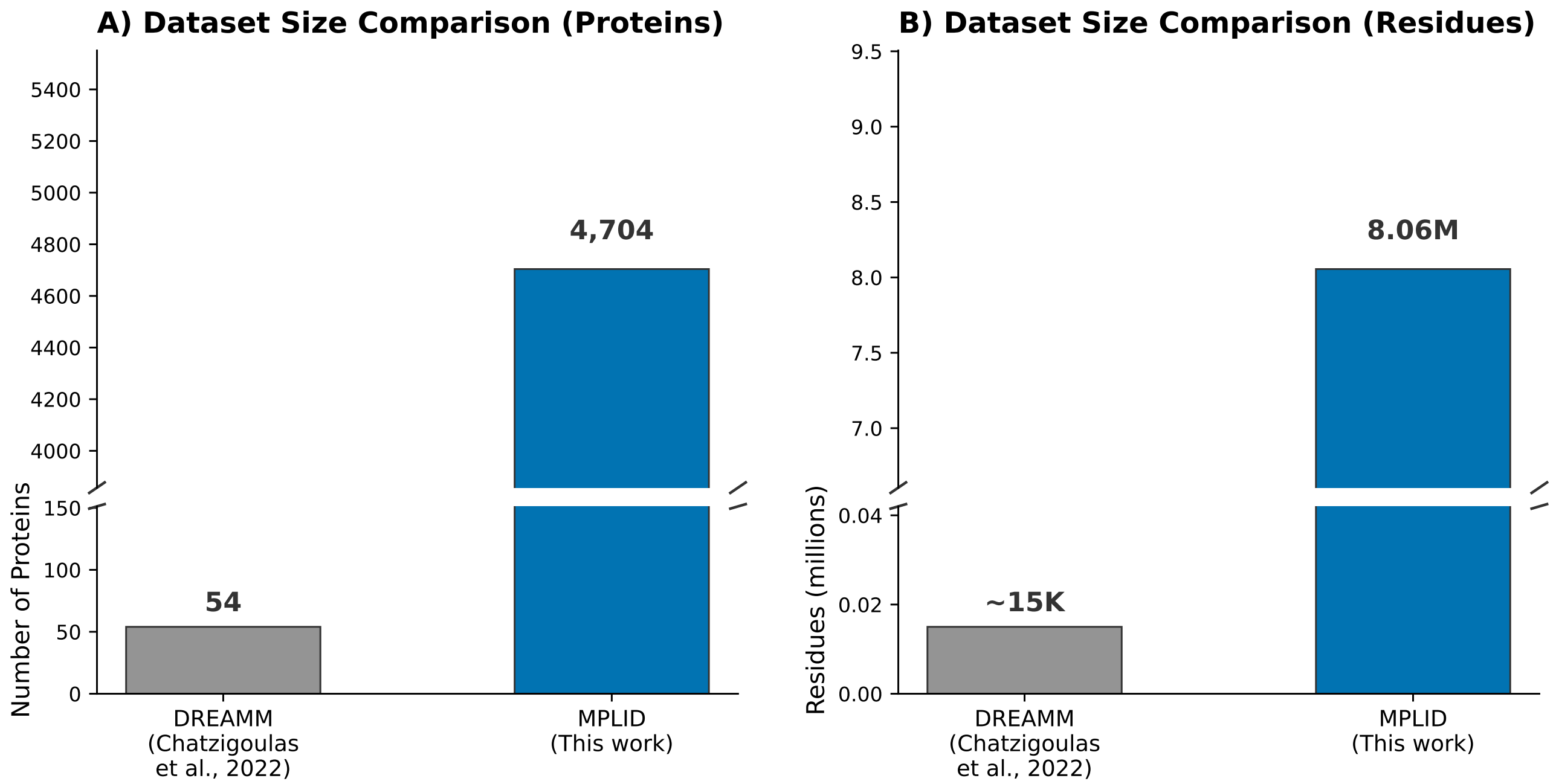

Fig. 6. Scale comparison between MPLID and the training data used by the DREAMM prediction method [22], the largest prior collection of experimentally annotated membrane protein-lipid interactions. (A) Number of proteins (4,704 versus 54). (B) Number of annotated residues (8,055,325 versus ∼15,000). Note that these resources differ in scope: DREAMM focuses on peripheral membrane proteins, while MPLID covers all membrane protein classes.

include sphingosine (SPH, 102 structures) and sphingosine-1-phosphate (S1P, 16 structures). Detergent mimetics used in crystallization, which often occupy native lipid binding sites, include ~~lauryl maltose neopentyl glycol~~ dodecyl β-D-maltoside (LMT, 677 structures), octyl β-D-glucopyranoside (BOG, 510 structures), and lauryldimethylamine oxide (LDA, 263 structures). ~~This comprehensive lipid recognition ensures that MPLID captures the full diversity of protein-lipid interactions observed in experimental structures.~~ The protein-level metadata includes an explicit `has_detergent_only` flag indicating whether all lipid contacts for a given protein derive exclusively from detergent mimetics rather than physiological lipid species, enabling users to filter detergent-only entries if desired.

Notably, several biologically important lipid classes are absent or underrepresented in the current MPLID release. Phosphatidic acid (PA), phosphatidylserine (PS), and phosphoinositides (PI, PIP, PIP_2, PIP_3) are not represented among the recognized lipid codes, despite their well-established roles in membrane protein regulation. This absence reflects both the difficulty of resolving these lipids crystallographically (owing to their flexible headgroups and transient binding modes) and the focus of MPLID on lipid species that are currently represented in PDB HETATM records. Similarly, polyunsaturated fatty acids (e.g., docosahexaenoic acid, arachidonic acid) are rarely resolved in crystal structures. Future releases will expand lipid coverage as more structures with these lipid types become available in the PDB.

Structural visualization of lipid contacts

To illustrate the nature of the lipid contact annotations in MPLID, representative membrane protein structures with their resolved lipid molecules are shown in Figure 7. These examples demonstrate that lipid contacts occur at specific, well-defined sites on the protein surface rather than uniformly across the membrane-exposed region. Contact residues (highlighted in red/orange) are interspersed with non-contact residues even in transmembrane regions, confirming that lipid contact prediction is a more specific task than membrane zone classification.

Computational label comparison studies

The availability of MPLID alongside OPM membrane zone annotations enables systematic comparison of experimental lipid contacts with computational membrane boundaries. We compared MPLID contact labels with OPM-derived membrane zone assignments across proteins present in both resources (Figure 8). The number of overlapping proteins varies by analysis: 2,383 proteins contributed to precision estimates, 1,685 to recall estimates, and 2,457 to contact distribution analysis, reflecting differences in the availability of OPM membrane zone annotations and non-zero lipid contacts across the shared set. Of all MPLID lipid contacts in the shared set (n = 28,497 residues), 51.2% fell within the OPM membrane zone while 48.8% were located outside the computationally defined boundaries, likely reflecting interfacial contacts, re-entrant loops, or amphipathic helices that extend beyond the hydrophobic core. Conversely, 98.8% of OPM membrane zone residues (n = 1,170,590) showed no direct lipid contact within 4.0 Å, and the per-protein median was only 0.7% of membrane zone residues having lipid contacts. The OPM membrane zone captured a median of 50.0% of lipid contacts per protein. These dataset-wide statistics highlight the fundamental difference between membrane zone classification and lipid contact prediction: being embedded in the membrane is neither sufficient nor necessary for direct lipid interaction. Researchers working with MemProtMD [15]

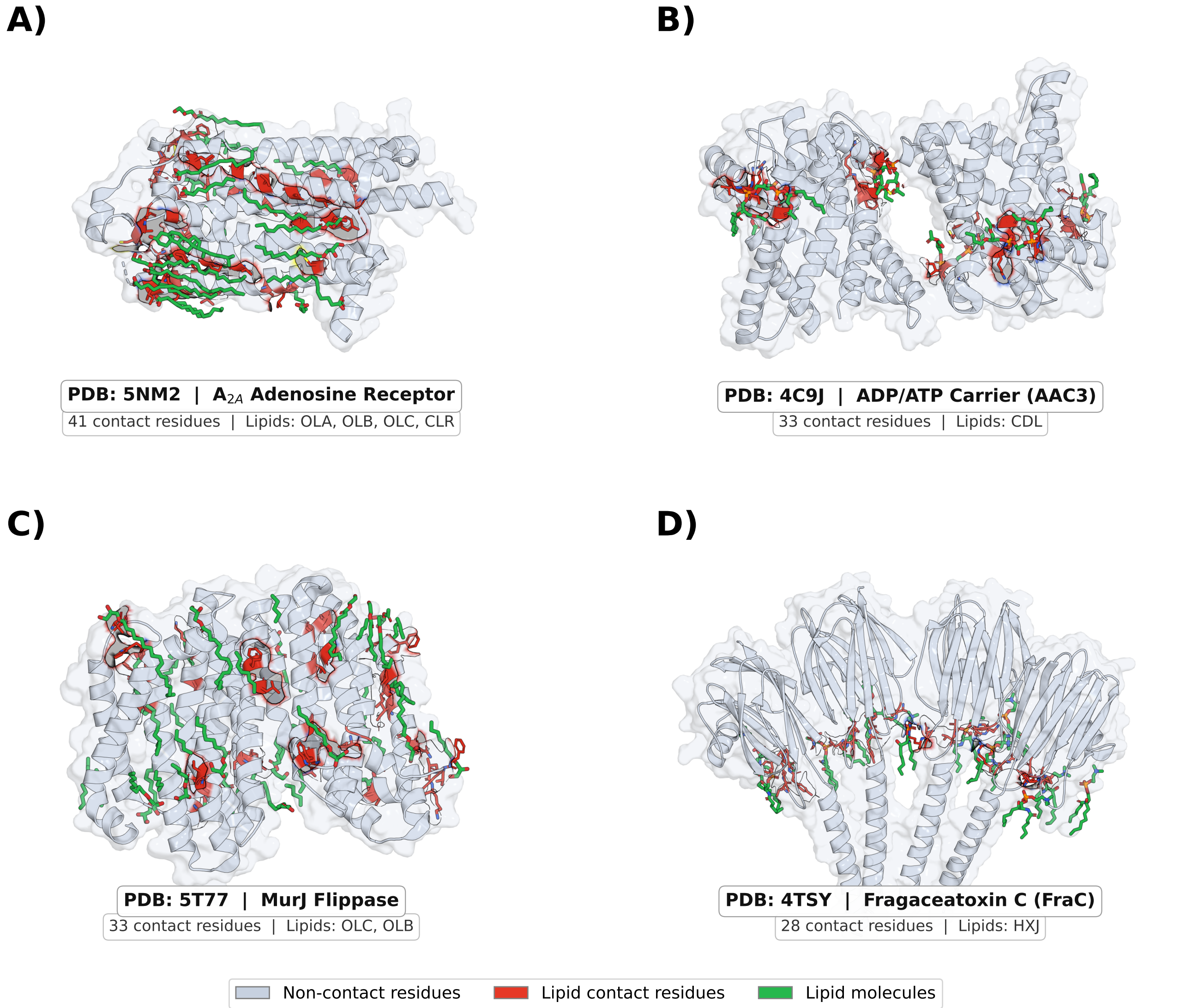

Fig. 7. Structural visualization of MPLID lipid contact annotations across four representative membrane protein classes. (A) A_{2A} adenosine receptor (PDB: 5NM2), a GPCR with multiple lipid types including oleic acid and cholesterol. (B) ADP/ATP carrier AAC3 (PDB: 4C9J), a mitochondrial transporter with cardiolipin contact residues across two chains. (C) MurJ flippase (PDB: 5T77), a lipid II flippase with contacts distributed across its transmembrane helices. (D) Fragaceatoxin C (PDB: 4TSY), a tetrameric pore-forming toxin with sphingolipid contacts at the membrane insertion interface. These examples demonstrate that lipid contacts are site-specific rather than uniformly distributed across membrane-exposed surfaces.

simulations can similarly compare simulated lipid contacts with the experimental contacts provided by MPLID.

Methods

Data source integration

MPLID was constructed using a purely experimental approach with no dependence on computational membrane positioning databases. OPM data were not used at any stage of the MPLID construction pipeline; OPM is discussed in this paper solely to contextualize the complementary biological questions of membrane zone positioning versus direct lipid contact annotation. All residue annotations in MPLID derive exclusively from experimentally resolved lipid molecules in PDB structures. The RCSB Protein Data Bank [26, 27] was queried programmatically for all structures containing recognized lipid chemical components, identifying 8,221 candidate structures with lipid ligands. This query-first strategy ensures that every protein in the dataset has at least one experimentally resolved lipid molecule, providing an unbiased sampling of protein-lipid interactions across all structural classes. The identified structures were downloaded as original PDB coordinate files (9,796 files total, as some entries required retrieval of biological assembly files or alternate depositions). After parsing, 5,723 unique proteins yielded valid structures with recognized HETATM lipid records; the remainder comprised duplicate chains, structures that failed parsing, or entries lacking standard amino acid residues. These were processed through the contact calculation and quality filtering pipeline described in the following subsections.

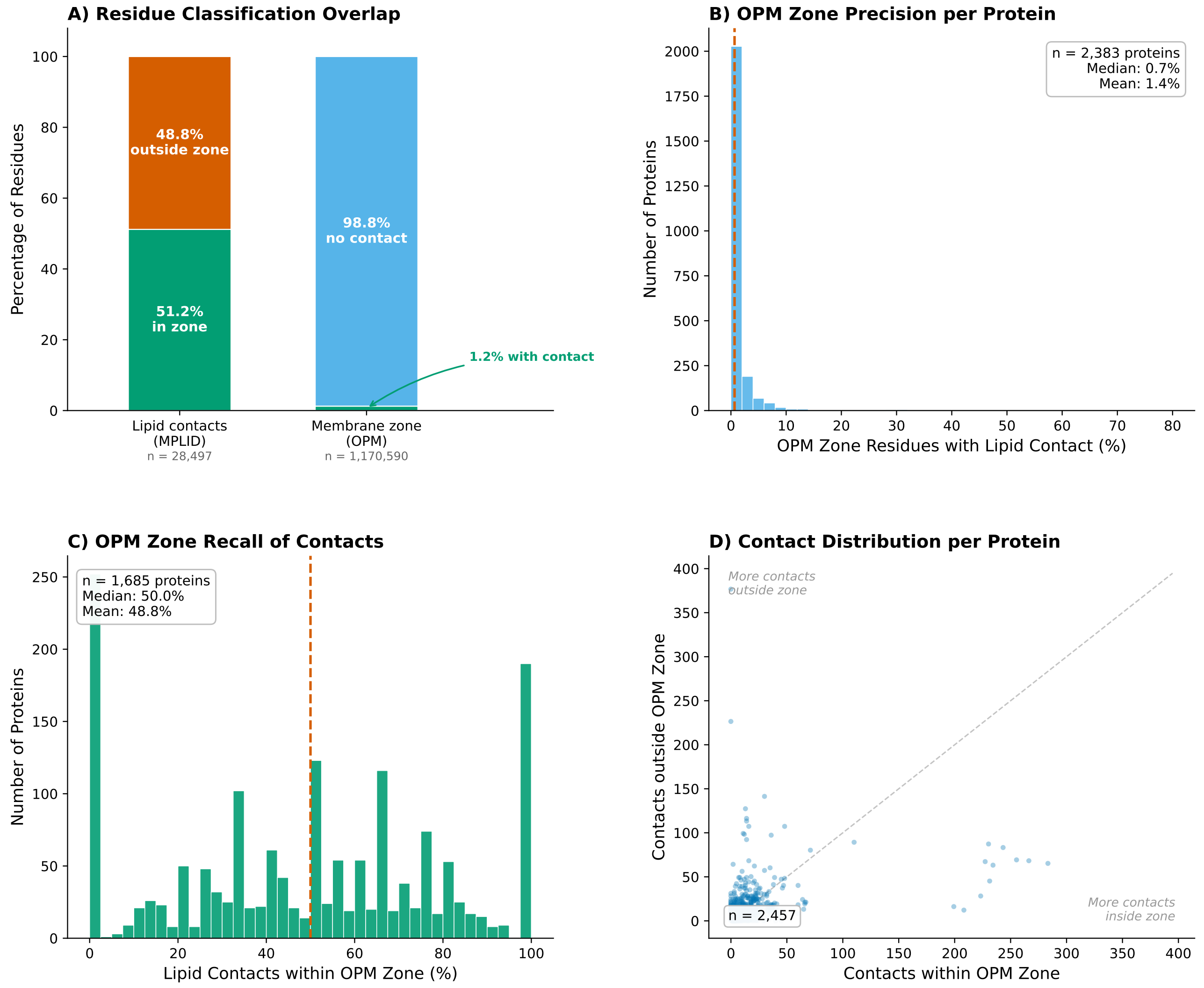

Fig. 8. Quantitative comparison of MPLID lipid contacts versus OPM membrane zone annotations. (A) Residue classification overlap: 48.8% of lipid contacts (n = 28,497) fall outside the OPM membrane zone, while 98.8% of OPM zone residues (n = 1,170,590) lack direct lipid contact. (B) Per-protein OPM precision (n = 2,383 proteins): median 0.7% of membrane zone residues have lipid contacts. (C) Per-protein OPM recall (n = 1,685 proteins): the membrane zone captures a median of 50.0% of lipid contacts. (D) Per-protein scatter of contacts inside versus outside the OPM zone (n = 2,457 proteins). These results demonstrate that membrane zone classification and lipid contact annotation address fundamentally different biological questions.

Lipid molecule identification

Lipid molecules in PDB structures were identified using their Chemical Component Dictionary codes. We compiled a list of 117 lipid codes organized into ten functional categories: phospholipids (CDL, POV, PCW, PEE, PGV, and others), cardiolipin (CDL, C9V, 18W, LCL), sphingolipids (SPH, S1P, HXJ, CRT), sterols (CLR, CHD, Y01, ERG), fatty acids (PLM, MYR, OLA, STE, ARA, DHA), glycerolipids (TGL, DAG, MAG), detergent mimetics (LDA, LMT, BOG, OLC, DPC), lipid A components (LPA, KDO, 6LP), and CHARMM simulation nomenclature (POPC, POPE, POPG, and others commonly found in cryo-EM depositions). This list was developed iteratively by cross-referencing the PDB Chemical Component Dictionary with lipid classification databases and manually curating entries to exclude non-lipid small molecules that might share similar chemical codes. Detergent mimetics were included because they frequently occupy native lipid binding sites in membrane protein crystal structures and provide relevant information about lipid-protein interaction geometry [7].

Structure processing and contact calculation

For each candidate protein, the original PDB coordinate file was downloaded from RCSB to preserve HETATM records containing lipid molecule coordinates. Structure parsing was performed using BioPython [34], extracting all standard amino acid residues (ATOM records) and all recognized lipid molecules (HETATM records). Non-standard residues, water molecules, metal ions, and non-lipid ligands were excluded.

Lipid contacts were defined using an all-atom distance criterion. For each protein residue R and each lipid molecule L in the structure, the minimum distance $d_{\min}(R, L)$ between any heavy (non-hydrogen) atom of R and any heavy atom of L was calculated. A residue was labeled as a lipid contact if:

$$d_{\min}(R, L) = \min_{a \in R,\, b \in L} \|\mathbf{r}_a - \mathbf{r}_b\| \leq 4.0 \text{ Å} \tag{1}$$

where $\mathbf{r}_a$ denotes the Cartesian coordinates of heavy atom a in residue R and $\mathbf{r}_b$ denotes the coordinates of heavy atom b in lipid L. The use of all heavy atoms (backbone and side chain) as residue representatives captures direct van der Waals contacts that would be missed by a Cα-only criterion, particularly for residues whose side chains extend into lipid-binding pockets while their backbone remains distant from lipid molecules. The 4.0 Å cutoff was chosen to capture direct van der Waals contacts between heavy atoms, consistent with standard distance thresholds used in protein-ligand interaction analysis [19], protein contact databases [20], and previous membrane protein-lipid interaction studies [22]. Typical van der Waals radii for carbon, nitrogen, and oxygen atoms sum to contact distances of 3.2–3.8 Å, and the 4.0 Å threshold provides a small margin to accommodate thermal displacement while remaining well below the threshold for indirect (water-mediated or electrostatic) interactions. The distance distribution analysis (Figure 4) independently validates this choice, showing a clear peak at 3.5–4.0 Å corresponding to van der Waals contacts. We note that this cutoff does not distinguish between contacts with lipid headgroups and acyl chains; both are captured by the all-atom criterion. Researchers interested in headgroup-specific or acyl-chain-specific contacts can use the continuous `min_distance` values together with the lipid atom identities available in the original PDB coordinate files. For each residue, the minimum all-atom distance to the nearest lipid heavy atom was recorded regardless of contact status, providing a continuous distance metric that enables researchers to evaluate alternative cutoff values.

Quality filtering

Of the 5,723 proteins with parseable lipid-containing structures, 1,019 were removed because they contained only non-lipid molecules misannotated as lipids (such as HEPES buffer and bacteriochlorophyll) or had all lipid atoms beyond the 4.0 Å contact threshold, leaving 4,704 proteins with at least one genuine lipid contact. This filtering ensures that every protein in the final dataset has genuine, experimentally resolved lipid contacts.

Sequence clustering

To prevent data leakage between training, validation, and test splits, all 4,704 proteins were clustered using MMseqs2 [29] with a 30% sequence identity threshold and 80% bidirectional alignment coverage. These stringent thresholds ensure that proteins assigned to different splits are sufficiently divergent in sequence to represent independent test cases. The clustering produced 813 clusters.

Split strategy

Clusters were assigned to training, validation, and test splits using stratified random sampling at the cluster level, with stratification based on the average contact rate per cluster. This approach ensures that each split contains a representative distribution of high-contact and low-contact proteins. Entire clusters were assigned to a single split, guaranteeing that no two proteins sharing more than 30% sequence identity appear in different partitions. The resulting splits contain 2,578 training proteins (54.8%), 1,051 validation proteins (22.3%), and 1,075 test proteins (22.9%).

Software and computational environment

The MPLID construction pipeline was implemented in Python 3.11.8 using BioPython 1.83 [34] for PDB structure parsing, pandas 2.2.2 [35] for data manipulation, NumPy 1.26.4 for numerical computation, SciPy 1.13 for distance calculations, and scikit-learn 1.7.2 [36] for data splitting. MMseqs2 version 15 [29] was used for sequence clustering. The RCSB PDB search API was used for programmatic identification of structures containing lipid chemical components.

Discussion

MPLID represents the first large-scale, publicly deposited dataset of experimentally validated residue-level lipid contacts for membrane proteins, complementing existing resources such as OPM (membrane zone positioning), MemBlob (membrane environment inference from cryo-EM density), and BioDolphin/PLIP (generalized structural interaction profiling). The dataset addresses a biological question that is fundamentally distinct from membrane zone classification: rather than identifying residues located within a computationally defined hydrophobic slab, MPLID annotates residues that make direct physical contact with ~~crystallized~~ experimentally resolved lipid molecules at atomic resolution. Importantly, MPLID captures only the subset of protein-lipid interactions that survive purification and are resolved in crystal or cryo-EM structures; the full biological lipid interaction landscape is substantially richer. The analyses presented here validate this annotation approach through multiple independent lines of evidence. The amino acid enrichment patterns at lipid contact sites are biologically consistent, with aromatic anchoring residues (tryptophan, phenylalanine) and positively charged residues (arginine, lysine) enriched at contact sites, while conformationally constrained (proline) and negatively charged (aspartate) residues are depleted, consistent with independent analyses of cardiolipin binding sites [33]. The distance distribution analysis confirms that the 4.0 Å cutoff effectively captures the van der Waals contact boundary. The quantitative comparison with OPM membrane zone annotations demonstrates that nearly half of experimentally observed lipid contacts fall outside computationally predicted membrane boundaries, reinforcing the complementary nature of these ~~two~~ resources.

The scale of MPLID, comprising 4,704 proteins across 813 sequence clusters, provides substantially greater coverage than prior experimental lipid contact datasets. The DREAMM training set [22], the largest previously available collection, contained 54 peripheral membrane proteins. MPLID expands this by nearly two orders of magnitude while covering all membrane protein classes, including integral, peripheral, and single-pass proteins. The cluster-aware splitting strategy, using a conservative 30% sequence identity threshold, ensures that evaluation on the test set reflects genuine generalization rather than sequence memorization.

Limitations

Several limitations should be noted when using MPLID. ~~First, lipid~~

False negatives in the non-contact class.

Lipid contact labels depend on the crystallographic or cryo-EM resolution and completeness of resolved lipid molecules; many native lipid binding events are not captured because lipids are

disordered or stripped during purification and crystallization. Consequently, the false negative rate in the non-contact class is likely substantial, ~~and the true positive rate~~ potentially exceeding the number of true positive contacts by an order of magnitude. The true frequency of lipid contacts in membrane proteins ~~is probably~~ in vivo is almost certainly higher than the 1.00% observed in MPLID. Moreover, many structures contain only one or a few resolved lipid molecules, meaning that the observed contacts represent a sparse snapshot of the full lipid-binding landscape rather than a comprehensive map of all possible interaction sites. ~~Second, the~~ Users training machine learning models on MPLID should be aware that the non-contact class is noisy and that model performance may be limited by false negative labels rather than by model capacity.

Detergent mimetics.
The inclusion of detergent mimetics as proxy lipids introduces potential noise, as detergent binding sites may not perfectly recapitulate native lipid interactions. Analysis of HETATM records across all 4,704 processed PDB files shows that ~~approximately 17% of proteins with recognized lipid molecules~~ 1,039 proteins (22.1%) contain exclusively detergent mimetics (e.g., dodecyl maltoside, octyl glucoside, lauryldimethylamine oxide) without any physiological lipid species, while an additional ~~10%~~685 proteins (14.6%) contain both detergent and physiological lipids. This means that for roughly one in ~~six~~ five proteins, all contact labels derive from crystallization detergents rather than native lipid species. ~~Third, the dataset~~ Detergents are retained in the dataset because they occupy membrane-associated structural environments and are part of the experimental structural context; however, the protein-level metadata includes an explicit detergent-only flag to enable filtering. The cholesterol derivative CHD (cholic acid, a bile acid sterol derivative) and Y01 (cholesterol hemisuccinate) are distinct compounds with different PDB Chemical Component Dictionary identities ($C_{24}H_{40}O_5$ and $C_{31}H_{50}O_4$, respectively), though both are classified as sterols in MPLID. Users should note that cholesterol hemisuccinate is an artificial detergent commonly added during membrane protein solubilization and is not a native membrane lipid.

Lipid code verification.
During revision, all 90 PDB Chemical Component Dictionary codes were cross-validated against the RCSB Chemical Component Dictionary API (Supplementary Table S1). This audit confirmed 45 codes as correctly classified, identified 34 codes with incorrect or ambiguous category assignments, and found 11 codes absent from the dictionary. Of the 34 misclassified codes, 15 appear in PDB HETATM records within the dataset, but most have low occurrence. The codes with the largest impact are CHD (cholic acid, present in 150 structures; name corrected from "cholesteryl hemisuccinate"), CRT (spirilloxanthin, a bacterial carotenoid incorrectly classified as ceramide, present in 22 structures), and ARA (alpha-L-arabinopyranose, a sugar incorrectly classified as arachidonic acid, present in 18 structures). These misclassified contacts collectively represent fewer than 300 protein entries and do not materially alter the dataset statistics. The three codes specifically flagged by reviewers (LNS, LNN, GLF) are confirmed as misclassified but do not appear in any PDB structure in the dataset (zero impact). The complete verification results are provided in Supplementary Table S1 for transparency.

NMR multi-model structures.
The current pipeline counts residue-lipid contacts across all conformational models in NMR-derived PDB coordinate files rather than extracting contacts from a single representative model. This convention affects 41 NMR structures in the dataset, contributing approximately 133,000 excess residue entries (1.65% of total residues) and approximately 24,000 excess contact annotations. The most extreme case is PDB 6W4F (KRAS4B homodimer on a lipid bilayer nanodisc), which contains 20 NMR models and reports 4,820 contacts across all models, compared to approximately 240 unique contact residues per individual model. Users working with NMR structures should be aware of this multi-model counting convention. Future releases will adopt a single-model extraction strategy for NMR structures.

PDB and lipid composition biases.
The dataset inherits biases from the PDB toward well-studied, readily crystallizable membrane protein families, leaving many therapeutically relevant but structurally uncharacterized families underrepresented. ~~Finally, the~~ Several biologically important lipid classes are absent, including phosphatidic acid (PA), phosphatidylserine (PS), phosphoinositides (PI, PIP, PIP_2, PIP_3), and polyunsaturated fatty acids. This absence reflects the difficulty of resolving these lipids crystallographically rather than a deliberate exclusion. Anionic lipids (particularly cardiolipin and phosphatidylglycerol) are overrepresented among structurally resolved lipid contacts relative to their abundance in biological membranes, which has implications for machine learning models trained on MPLID: such models may learn structural detectability biases rather than purely biological binding affinity patterns. Additionally, the removal of 1,019 proteins with zero contacts during quality filtering means that MPLID does not represent the complete membrane proteome and cannot serve as a source of proteome-wide negative controls.

Distance cutoff and static snapshots.
The 4.0 Å distance cutoff is a single threshold that may not be optimal for all lipid types; researchers are encouraged to explore alternative cutoffs using the continuous distance values provided in the dataset. ~~Additionally, because~~ The cutoff does not distinguish between headgroup and acyl-chain contacts, both of which are captured by the all-atom criterion. Because MPLID annotations are derived from static crystallographic and cryo-EM snapshots, they may not fully represent the dynamic nature of ~~protein lipid~~ protein-lipid interactions in native membrane environments, where lipids undergo lateral diffusion and exchange on nanosecond-to-microsecond timescales. Crystal packing artifacts may also contribute false positive contacts, as lipid molecules resolved in crystal structures can occupy lattice contact interfaces that do not reflect native membrane binding.

Additional technical notes.
No structure resolution cutoff was applied during dataset construction: all PDB structures with recognized lipid molecules were included regardless of crystallographic resolution. While this maximizes dataset size, low-resolution structures (worse than 3.5 Å) may have less reliable lipid positions, potentially introducing noise into contact labels. Researchers working with resolution-sensitive applications are encouraged to filter by PDB resolution using the provided `pdb_id` field.

The current release does not include per-residue lipid chemical component codes, meaning that users cannot directly determine which lipid type ~~(e.g., cholesterol versus cardiolipin)~~ contacts a given residue~~. However, the continuous min_distance values enable evaluation of alternative distance cutoffs, and~~ ; however, the pdb_id field allows users to retrieve lipid identities from the original PDB structures. Lipid type stratification at the residue level is planned for a future release.

Four selenocysteine (SEC) residues in the dataset are all annotated as lipid contacts (4/4, 100% contact rate)~~. Given~~ ; given the extremely small sample size, this observation is not statistically meaningful and these residues were excluded from enrichment ratio analysis.

Cross-validation of MPLID labels against BioDolphin [18] interaction annotations and the curated dataset used by the DREAMM prediction method [22] is planned as future work to assess concordance between ~~the two~~ independently derived annotation approaches.

Conclusions

In summary, MPLID provides a large-scale, experimentally grounded resource that fills a critical gap in the membrane protein structural biology landscape. By distinguishing direct lipid contact from membrane zone localization, and by positioning itself as complementary to existing resources including OPM, MemBlob, and BioDolphin/PLIP, the dataset enables a new class of residue-level prediction tasks with direct relevance to drug discovery, protein engineering, and mechanistic understanding of lipid-mediated regulation. Users should interpret MPLID annotations as representing structurally resolved lipid contacts rather than a complete inventory of biological protein-lipid interactions, and should account for the substantial false negative rate inherent to crystallographic and cryo-EM datasets. The dataset adheres to FAIR principles and is freely available under a CC0 public domain dedication. ~~MPLID is intended as a ground-truth resource for residue-level lipid contact annotation and does not aim, by itself, to provide an optimized predictive solution to the full experimental-only classification problem.~~ As the PDB continues to grow and cryo-EM structures increasingly resolve lipid molecules at higher resolution, future releases of MPLID will expand both the coverage and the annotation granularity of this resource, including NMR single-model extraction, per-residue lipid type annotations, and expanded lipid ontology coverage.

Potential implications

MPLID is designed to serve as a large-scale training and evaluation resource for multiple research applications in structural bioinformatics, drug discovery, and membrane biology.

Machine learning for lipid contact prediction

The principal application of MPLID is as a training and evaluation dataset for developing machine learning models that predict lipid contact residues from protein sequence or structure. The pre-defined, cluster-aware train/validation/test splits enable standardized model comparison, and the class imbalance (1.00% observed positive rate, approximately 100:1 negative-to-positive ratio) presents a realistic and challenging binary classification task that requires specialized approaches such as class weighting, oversampling (SMOTE) [37], or focal loss functions. Recent advances in geometric deep learning for protein interaction site prediction [38, 39] and end-to-end protein interactome modeling [40] demonstrate the growing potential of graph-based and attention-based architectures for residue-level prediction tasks. While these methods have been developed primarily for protein-protein interaction sites, their hierarchical geometric representations and multiscale attention mechanisms are architecturally suitable for the protein-lipid contact prediction task addressed by MPLID, particularly given the spatial nature of lipid binding sites. The scale of the dataset is sufficient for training deep neural networks, including architectures that combine structural descriptors with protein language model embeddings from ESM-2 [28] or ProtTrans [25]. We note that the Matthews correlation coefficient should be used as the primary evaluation metric for this task due to the substantial class imbalance, as accuracy and even F1 scores can be misleading when the negative class dominates. Users should also be aware that the substantial false negative rate in the non-contact class (see Limitations) may impose an inherent ceiling on achievable performance metrics.

Drug discovery applications

Lipid binding sites on membrane proteins represent an emerging class of druggable targets. General anesthetics, neurosteroids, and endocannabinoids modulate ion channels and GPCRs through specific lipid binding sites [6, 41], and cholesterol binding sites on GPCRs are increasingly recognized as allosteric modulatory sites [8, 42]. This interpretation is consistent with recent work highlighting exosites and allosteric site-forming residues as therapeutically actionable protein-surface regions [43, 44, 45]. MPLID can inform the identification of lipid-mimetic drug binding sites by providing a catalog of experimentally validated lipid interaction residues across thousands of membrane proteins. Predicted lipid contact sites on therapeutically relevant proteins can guide the design of lipid-conjugated prodrugs, membrane-anchored therapeutics, or allosteric modulators that exploit lipid binding pockets.

Structural biology and protein engineering

Understanding which residues contact lipids is essential for rational membrane protein engineering. Mutations at lipid contact sites can destabilize membrane proteins, alter their trafficking, or modify their functional properties. MPLID provides data to train models that predict which residues are critical for lipid-mediated stability, enabling more informed design of stabilizing mutations for membrane protein crystallization, cryo-EM, or functional studies. Additionally, the dataset supports comparative analysis of lipid contact patterns across protein families, enabling identification of conserved lipid binding motifs and family-specific interaction signatures. Future work incorporating cavity-based structural analysis could further support the observation that many residues located within the membrane region do not form direct lipid contacts, reinforcing the conceptual separation between membrane localization and residue-level interaction. These interpretations are based on static structural snapshots. Incorporation of dynamic information may alter residue–lipid proximity patterns, as conformational fluctuations could transiently modify contact definitions.

Integration with protein language models

The residue-level format of MPLID is directly compatible with per-residue embeddings from protein language models. Each residue's 1,280-dimensional ESM-2 embedding [28] or 1,024-dimensional ProtTrans embedding [25] can be concatenated with structural descriptors and used as input to supervised classifiers. The 813 sequence clusters provide sufficient diversity to evaluate whether language model representations generalize across membrane protein families with limited sequence similarity. Alternatively, MPLID can be combined with interpretable structural descriptor frameworks, such as STING RDB-derived nanoenvironment descriptors [20, 21, 43, 44]. In this setting, residue-level lipid contacts may be analyzed in terms of explicit physicochemical and geometric properties, enabling mechanistic interpretation of the nanoenvironment characteristics associated with lipid interaction. This dual compatibility supports both high-capacity representation learning approaches and descriptor-based, interpretable modeling strategies.

Availability of supporting source code and data

The complete MPLID dataset is deposited at Zenodo under a CC0 public domain dedication with persistent DOI: `https://doi.org/10.5281/zenodo.18487584` [46]. Upon acceptance, the dataset will also be deposited in GigaDB in accordance with journal policy. The Zenodo archive contains the full residue-level annotations (train, validation, and test splits as gzip-compressed CSV files), protein-level metadata, amino acid composition statistics, and a data dictionary describing all fields.

All code for reproducing the dataset from raw PDB structures is available in a public GitHub repository: `https://github.com/omagebright/MPLID`. The repository includes the complete processing pipeline (structure downloading, lipid identification, contact calculation, quality filtering, and split generation), analysis scripts for generating figures and tables, and documentation of the data schema and methodology.

The computational workflow is registered in WorkflowHub (`https://workflowhub.eu/workflows/2176`). MPLID is registered in the bio.tools registry (biotoolsID: `MPLID`; `https://bio.tools/MPLID`). MPLID is registered in the SciCrunch Resource Registry as Membrane Protein-Lipid Interaction Database, RRID:`SCR_028506`.

The dataset and code are organized following FAIR principles [47]: data are Findable via persistent DOIs and indexed metadata; Accessible through standard HTTPS download from Zenodo and GitHub; Interoperable using standard CSV format with documented column definitions; and Reusable under permissive open licenses (CC0 for data, MIT for code).

Conflicts of interest

The authors declare that they have no competing interests.

Funding

This research was funded by the São Paulo Research Foundation (FAPESP) (grant nos. 2020/08615-8, 2023/02691-2, 2025/23708-6).

Data availability

The data underlying this article are available in Zenodo at `https://doi.org/10.5281/zenodo.18487584`, and in a public GitHub repository at `https://github.com/omagebright/MPLID`.

Author contributions statement

Author contributions follow the CRediT taxonomy. F.B.O.: Conceptualization, Methodology, Software, Formal Analysis, Data Curation, Writing (Original Draft), Visualization. G.N.: Conceptualization (dataset definition and biological framework), Supervision, Writing (Original Draft, Review & Editing), Funding Acquisition. Both authors read and approved the final manuscript.

Ethics approval and consent to participate

Not applicable. This study uses publicly available structural data from the Protein Data Bank and does not involve human subjects, animal experiments, or clinical data.

Acknowledgments

The authors thank Ivan Mazoni and Inácio Henrique Yano for their contributions to data validation and resource management during the early stages of this work. The authors thank Embrapa Digital Agriculture (Embrapa Agricultura Digital) for computational resources and infrastructure support. The authors acknowledge the RCSB Protein Data Bank [26, 27] and the OPM database [12] teams for maintaining the essential structural biology resources upon which this work depends. The authors thank the developers of MMseqs2 [29], BioPython [34], and the broader open-source scientific computing community for the software tools used in this study.

Abbreviations

MPLID: Membrane Protein-Lipid Interaction Database; **PDB**: Protein Data Bank; **OPM**: Orientations of Proteins in Membranes; **GPCR**: G protein-coupled receptor; **MCC**: Matthews correlation coefficient; **CG-MD**: Coarse-grained molecular dynamics; **FAIR**: Findable, Accessible, Interoperable, Reusable; **CDL**: Cardiolipin; **CLR**: Cholesterol; **PLM**: Palmitic acid; **DREAMM**: Detection of Residues on Exposed Areas of Membrane-associated Macromolecules; **ESM-2**: Evolutionary Scale Modeling 2; **SMOTE**: Synthetic Minority Over-sampling Technique; **PLIP**: Protein-Ligand Interaction Profiler; **RCSB**: Research Collaboratory for Structural Bioinformatics; **NMR**: Nuclear magnetic resonance; **CSV**: Comma-separated values.

References

1. Erik Wallin and Gunnar von Heijne. Genome-wide analysis of integral membrane proteins from eubacterial, archaean, and eukaryotic organisms. *Protein Science*, 7(4):1029–1038, 1998. doi: 10.1002/pro.5560070420.
2. John P Overington, Bissan Al-Lazikani, and Andrew L Hopkins. How many drug targets are there? *Nature Reviews Drug Discovery*, 5(12):993–996, 2006. doi: 10.1038/nrd2199.

3. Rita Santos, Oleg Ursu, Anna Gaulton, et al. A comprehensive map of molecular drug targets. *Nature Reviews Drug Discovery*, 16(1):19–34, 2017. doi: 10.1038/nrd.2016.230.
4. Valentina Corradi, Besian I Sejdiu, Haydee Mesa-Galloso, et al. Emerging diversity in lipid–protein interactions. *Chemical Reviews*, 119(9):5775–5848, 2019. doi: 10.1021/acs.chemrev.8b00451.
5. Arthur Laganowsky, Eamonn Reading, Timothy M Allison, et al. Membrane proteins bind lipids selectively to modulate their structure and function. *Nature*, 510(7503):172–175, 2014. doi: 10.1038/nature13419.
6. Anna L Duncan, Wanling Song, and Mark SP Sansom. Lipid-dependent regulation of ion channels and G protein-coupled receptors: insights from structures and simulations. *Annual Review of Pharmacology and Toxicology*, 60:31–50, 2020. doi: 10.1146/annurev-pharmtox-010919-023411.
7. Anthony G Lee. How lipids affect the activities of integral membrane proteins. *Biochimica et Biophysica Acta - Biomembranes*, 1666(1-2):62–87, 2004. doi: 10.1016/j.bbamem.2004.05.012.
8. Michael A Hanson, Vadim Cherezov, Mark T Griffith, et al. A specific cholesterol binding site is established by the 2.8 å structure of the human β2-adrenergic receptor. *Structure*, 16(6):897–905, 2008. doi: 10.1016/j.str.2008.05.001.
9. Felix-Xavier Contreras, Andreas M Ernst, Felix Wieland, and Britta Brügger. Specificity of intramembrane protein–lipid interactions. *Cold Spring Harbor Perspectives in Biology*, 3(6):a004705, 2011. doi: 10.1101/cshperspect.a004705.
10. Takeshi Harayama and Howard Riezman. Understanding the diversity of membrane lipid composition. *Nature Reviews Molecular Cell Biology*, 19(5):281–296, 2018. doi: 10.1038/nrm.2017.138.
11. Gerrit van Meer, Dennis R Voelker, and Gerald W Feigenson. Membrane lipids: where they are and how they behave. *Nature Reviews Molecular Cell Biology*, 9(2): 112–124, 2008. doi: 10.1038/nrm2330.
12. Mikhail A Lomize, Irina D Pogozheva, Hyeon Joo, Henry I Mosberg, and Andrei L Lomize. OPM database and PPM web server: resources for positioning of proteins in membranes. *Nucleic Acids Research*, 40(D1):D370–D376, 2012. doi: 10.1093/nar/gkr703.
13. Andrei L Lomize, Irina D Pogozheva, Mikhail A Lomize, and Henry I Mosberg. Positioning of proteins in membranes: a computational approach. *Protein Science*, 15(6):1318–1333, 2006. doi: 10.1110/ps.062126106.
14. Alexios Chatzigoulas and Zoe Cournia. DREAMM: a web-based server for drugging protein-membrane interfaces as a novel workflow for targeted drug design. *Bioinformatics*, 38 (24):5449–5451, 2022. doi: 10.1093/bioinformatics/btac680.
15. Thomas D Newport, Mark SP Sansom, and Phillip J Stansfeld. The MemProtMD database: a resource for membrane-embedded protein structures and their lipid interactions. *Nucleic Acids Research*, 47(D1):D390–D397, 2019. doi: 10.1093/nar/gky1047.
16. Phillip J Stansfeld, Joseph E Goose, Martin Caffrey, et al. MemProtMD: automated insertion of membrane protein structures into explicit lipid membranes. *Structure*, 23(7): 1350–1361, 2015. doi: 10.1016/j.str.2015.05.006.
17. Lukasz P Kozłowski and Janusz M Bujnicki. MemBlob database and server for identifying transmembrane regions using cryo-EM maps. *Bioinformatics*, 35(19):3582–3584, 2019. doi: 10.1093/bioinformatics/btz539.
18. Jorge Roel-Touris, Heidi Koldsoe, and Manuel N. Melo. BioDolphin: an open-source computational platform for the analysis of lipid-protein interactions in the PDB. *Communications Chemistry*, 7:Article 308, 2024. doi: 10.1038/s42004-024-01384-z.
19. Melissa F Adasme, Katja L Linnemann, Sarah Naomi Bolz, et al. PLIP 2021: expanding the scope of the protein–ligand interaction profiler to DNA and RNA. *Nucleic Acids Research*, 49(W1):W530–W534, 2021. doi: 10.1093/nar/gkab294.
20. Goran Neshich, Roberto C Togawa, Adauto L Mancini, Paula R Kuser, Michel EB Yamagishi, Georgios Pappas Jr, Wandré V Torres, Thalita Fonseca e Campo, Leandro L Ferreira, Fabio M Luna, et al. STING Millennium: a web-based suite of programs for comprehensive and simultaneous analysis of protein structure and sequence. *Nucleic Acids Research*, 31(13):3386–3392, 2003. doi: 10.1093/nar/gkg578.
21. Goran Neshich, Ivan Mazoni, Sandro Roberto Oliveira, Michel EB Yamagishi, Paula R Kuser-Falcão, Leandro C Borro, Dag H Morita, Katlin Rabelo Souza, Vagner M Gonçalves-Almeida, et al. STING Report: convenient web-based application for graphic and tabular presentations of protein sequence, structure and function descriptors from the STING database. *Nucleic Acids Research*, 34(suppl_2): W209–W213, 2006. doi: 10.1093/nar/gkl226.
22. Alexios Chatzigoulas and Zoe Cournia. Predicting protein–membrane interfaces of peripheral membrane proteins using ensemble machine learning. *Briefings in Bioinformatics*, 23(2):bbab518, 2022. doi: 10.1093/bib/bbab518.
23. Niek van Hilten, Nino Verwei, Jeroen Methorst, Carsten Nase, Andrius Bernatavicius, and Herre Jelger Risselada. PMIpred: a physics-informed web server for quantitative protein–membrane interaction prediction. *Bioinformatics*, 40(2):btae069, 2024. doi: 10.1093/bioinformatics/btae069.
24. Dimitra Paranou, Alexios Chatzigoulas, and Zoe Cournia. Using deep learning and large protein language models to predict protein–membrane interfaces of peripheral membrane proteins. *Bioinformatics Advances*, 4(1): vbae078, 2024. doi: 10.1093/bioadv/vbae078.
25. Ahmed Elnaggar, Michael Heinzinger, Christian Dallago, et al. ProtTrans: toward understanding the language of life through self-supervised learning. *IEEE Transactions on Pattern Analysis and Machine Intelligence*, 44(10):7112–7127, 2022. doi: 10.1109/TPAMI.2021.3095381.
26. Helen M Berman, John Westbrook, Zukang Feng, et al. The Protein Data Bank. *Nucleic Acids Research*, 28(1): 235–242, 2000. doi: 10.1093/nar/28.1.235.
27. Stephen K Burley, Charmi Bhikadiya, Chunxiao Bi, et al. RCSB Protein Data Bank (rcsb.org): delivery of experimentally-determined PDB structures alongside one million computed structure models of proteins from artificial intelligence/machine learning. *Nucleic Acids Research*, 51(D1):D488–D508, 2023. doi: 10.1093/nar/gkac1077.
28. Zeming Lin, Halil Akin, Roshan Rao, et al. Evolutionary-scale prediction of atomic-level protein structure with a language model. *Science*, 379(6637):1123–1130, 2023. doi: 10.1126/science.ade2574.
29. Martin Steinegger and Johannes Söding. MMseqs2 enables sensitive protein sequence searching for the analysis of massive data sets. *Nature Biotechnology*, 35(11):1026–1028, 2017. doi: 10.1038/nbt.3988.

30. Ian Sillitoe, Nicola Bordin, Natalie Dawson, et al. CATH: increased structural coverage of functional space. *Nucleic Acids Research*, 49(D1):D266–D273, 2021. doi: 10.1093/nar/gkaa1079.
31. Gunnar von Heijne. Membrane protein structure prediction: hydrophobicity analysis and the positive-inside rule. *Journal of Molecular Biology*, 225(2):487–494, 1992. doi: 10.1016/0022-2836(92)90934-C.
32. William P Russ and Donald M Engelman. The GxxxG motif: a framework for transmembrane helix-helix association. *Journal of Molecular Biology*, 296(3):911–919, 2000. doi: 10.1006/jmbi.1999.3489.
33. Anna L Duncan, Jonathan J Ruprecht, Edmund RS Kunji, and Alan J Robinson. Cardiolipin binds selectively but transiently to conserved lysine residues in the rotor of metazoan ATP synthases. *Science Advances*, 7(34): eabh2217, 2021. doi: 10.1126/sciadv.abh2217.
34. Peter JA Cock, Tiago Antao, Jeffrey T Chang, et al. Biopython: freely available Python tools for computational molecular biology and bioinformatics. *Bioinformatics*, 25 (11):1422–1423, 2009. doi: 10.1093/bioinformatics/btp163.
35. Wes McKinney. Data structures for statistical computing in Python. In *Proceedings of the 9th Python in Science Conference*, pages 56–61, 2010. doi: 10.25080/Majora-92bf1922-00a.
36. Fabian Pedregosa, Gaël Varoquaux, Alexandre Gramfort, et al. Scikit-learn: machine learning in Python. *Journal of Machine Learning Research*, 12:2825–2830, 2011.
37. Nitesh V Chawla, Kevin W Bowyer, Lawrence O Hall, and W Philip Kegelmeyer. SMOTE: synthetic minority over-sampling technique. *Journal of Artificial Intelligence Research*, 16:321–357, 2002. doi: 10.1613/jair.953.
38. Yong Han, Shao-Wu Zhang, Qing-Qing Zhang, and Ming-Hui Shi. MGMA-PPIS: Predicting the protein-protein interaction site with multiview graph embedding and multiscale attention fusion. *GigaScience*, 14:giaf114, 2025. doi: 10.1093/gigascience/giaf114.
39. Shizhuo Zhang, Jiyun Han, and Juntao Liu. Protein-protein and protein-nucleic acid binding site prediction via interpretable hierarchical geometric deep learning. *GigaScience*, 13:giae080, 2024. doi: 10.1093/gigascience/giae080.
40. Yu-Hsin Chen, Chien-Fu Liu, Jun-Yi Leu, and Huai-Kuang Tsai. Complete end-to-end learning from protein feature representation to protein interactome inference. *GigaScience*, 14:giaf122, 2025. doi: 10.1093/gigascience/giaf122.
41. Grace Brannigan, Jérôme Hénin, Richard Law, et al. Embedded cholesterol in the nicotinic acetylcholine receptor. *Proceedings of the National Academy of Sciences*, 105(38):14418–14423, 2008. doi: 10.1073/pnas.0803029105.
42. Jacques Fantini and Francisco J Barrantes. How cholesterol interacts with membrane proteins: an exploration of cholesterol-binding sites including CRAC, CARC, and tilted domains. *Frontiers in Physiology*, 4:31, 2013. doi: 10.3389/fphys.2013.00031.
43. Folorunsho Bright Omage, Jorge A. Salim, Ivan Mazoni, Inácio Henrique Yano, Leandro Borro, Juan E. H. Gonzalez, Monica Pickholz, I. A. P. Neshich, I. P. Sousa, K. Santana, A. L. Rochetti, J. Fernandes, J. G. Jardine, G. T. Valente, A. L. Mancini, L. Tasic, R. K. Arni, R. Parise Filho, and Goran Neshich. Protein allosteric site identification using machine learning. *Computational and Structural Biotechnology Journal*, 23:3907–3919, 2024. doi: 10.1016/j.csbj.2024.10.036.
44. Folorunsho Bright Omage, Jorge A. Salim, Ivan Mazoni, Inácio Henrique Yano, Juan E. H. González, I. A. P. Neshich, Leandro C. Borro, Monica Pickholz, J. Fernandes, J. G. Jardine, A. L. Mancini, R. K. Arni, L. Tasic, and Goran Neshich. STINGAllo: a web server for high-throughput prediction of allosteric site-forming residues. *Briefings in Bioinformatics*, 26(4):bbaf424, 2025. doi: 10.1093/bib/bbaf424.
45. Folorunsho Bright Omage, Ivan Mazoni, Inácio Henrique Yano, and Goran Neshich. Protein exosites as drug targets: from structural topology to therapeutic modality. *Drug Discovery Today*, 31(3):104662, 2026. doi: 10.1016/j.drudis.2026.104662.
46. Folorunsho Bright Omage and Goran Neshich. MPLID: Membrane protein-lipid interface dataset, 2026. URL `https://doi.org/10.5281/zenodo.18487584`. Version 2.0.0.
47. Mark D Wilkinson, Michel Dumontier, IJsbrand Jan Aalbersberg, et al. The FAIR guiding principles for scientific data management and stewardship. *Scientific Data*, 3(1):160018, 2016. doi: 10.1038/sdata.2016.18.

Manuscript

Click here to access/download;Manuscript;MPLID_main_gigascience_UPD

GigaScience, 2026, pp. 1–16

doi:
Data Note

DATA NOTE

MPLID (Membrane Protein–Lipid Interaction Database): A Large-Scale Experimental Resource of Residue-Level Protein–Lipid Contacts

Folorunsho Bright Omage[1,2,3,*] and Goran Neshich[2,*]

[1]Department of Computer Science, University of Oxford, Wolfson Building, Parks Road, OX1 3QD, Oxford, United Kingdom, [2]Computational Biology Research Group, Embrapa Digital Agriculture, Av. André Tosello 209, 13083-886, Campinas, São Paulo, Brazil and [3]Biological Chemistry Laboratory, Department of Organic Chemistry, Institute of Chemistry, University of Campinas (UNICAMP), R. Monteiro Lobato 270, 13083-970, Campinas, São Paulo, Brazil

*Corresponding authors. folorunsho.bright.omage@cs.ox.ac.uk; goran.neshich@embrapa.br

Abstract

Background: Membrane proteins constitute approximately 20–30% of all proteomes and represent over 60% of current drug targets. Although protein–lipid interactions play important structural and regulatory roles in membrane-associated proteins, most existing structural resources focus on identifying whether a residue lies within a membrane region, typically inferred from computational hydrophobicity-based positioning algorithms. This approach does not directly address a distinct biological question: which residues at the protein surface make direct physical contact with lipid molecules? Answering this question from experimental data is critical for understanding lipid-mediated allostery, designing lipid-mimetic therapeutics, and training accurate machine learning models for lipid binding site prediction. **Findings:** We present MPLID (Membrane Protein-Lipid Interaction Database), a curated residue-level dataset comprising 4,704 membrane proteins representing 813 sequence clusters at 30% identity, 8,055,325 residues, and 80,439 experimentally validated lipid contact annotations (1.00% observed positive rate). Labels are derived exclusively from crystallized lipid molecules resolved in Protein Data Bank structures using a 4.0 Å all-atom heavy-atom distance cutoff. Because most native lipid interactions are lost during purification and crystallization, this observed rate represents a lower bound, and the non-contact class inevitably contains false negatives. The dataset uses a curated list of 117 candidate lipid identifiers across ten functional categories, including 90 PDB-derived ligand codes audited against the RCSB Chemical Component Dictionary and 27 CHARMM-style lipid identifiers encountered in cryo-EM depositions. These identifiers span phospholipids, cardiolipin, sphingolipids, sterols, fatty acids, glycerolipids, detergent mimetics (explicitly flagged), lipid A components, and CHARMM simulation nomenclature. To prevent data leakage, proteins are clustered at 30% sequence identity using MMseqs2, yielding 813 clusters partitioned into training (2,578), validation (1,051), and test (1,075) splits. Amino acid composition analysis reveals biologically consistent enrichment at lipid contact sites: tryptophan (1.88×), arginine (1.44×), glycine (1.36×), lysine (1.33×), and phenylalanine (1.23×) are enriched, while proline (0.51×), isoleucine (0.57×), and aspartate (0.59×) are depleted. **Conclusions:** MPLID addresses a distinct biological question compared to existing resources (OPM, MemBlob, BioDolphin/PLIP): identifying residues that directly contact experimentally resolved lipid molecules rather than those positioned within computationally defined membrane boundaries. With 4,704 proteins and over 8 million annotated residues, MPLID provides the scale needed for training deep learning models for lipid contact prediction, with direct applications in structure-guided drug design and membrane protein engineering. The dataset adheres to FAIR principles and is freely available under a CC0 public domain dedication. Structurally resolved contacts represent only a subset of biological protein-lipid interactions, and MPLID is intended as an experimentally grounded resource rather than a complete catalog of lipid binding sites.

Key words: membrane proteins, lipid-protein interactions, protein structure, machine learning dataset, structural biology, experimental validation, residue-level classification, FAIR data

Background

Membrane proteins are central to virtually all cellular processes, mediating transport of ions and solutes, signal transduction, energy conversion, and cell adhesion [1]. Genomic analyses consistently estimate that 20–30% of open reading frames in any given proteome encode membrane proteins [1], and pharmacological surveys have established that these

© The Author 2026. Published by Oxford University Press. All rights reserved. For permissions, please e-mail: journals.permissions@oup.com

proteins account for more than 60% of all current drug targets [2, 3]. Despite their biomedical importance, membrane proteins remain among the most challenging targets for structural and computational biology, in large part because their native functional context, the lipid bilayer, is difficult to recapitulate experimentally and model computationally.

A growing body of evidence demonstrates that lipid-protein interactions are not passive consequences of membrane embedding but rather active modulators of protein structure, dynamics, and function [4, 5]. Specific lipid binding events regulate ion channel gating [6], G protein-coupled receptor (GPCR) signaling [4], and transporter conformational cycling [7]. Cholesterol, for example, occupies defined binding sites on GPCRs that are structurally resolved at atomic resolution [8], and cardiolipin binding is essential for the function of mitochondrial respiratory chain complexes [9]. The diversity of lipid species in biological membranes, encompassing phospholipids, sphingolipids, sterols, and glycolipids [10, 11], creates a complex interaction landscape that shapes the behavior of membrane-embedded proteins.

Membrane zone versus lipid contact: two distinct questions

A fundamental distinction exists between two related but different biological questions in membrane protein structural biology. The first question, "Is this residue located within the membrane zone?", has been addressed by computational databases such as the Orientations of Proteins in Membranes (OPM) database [12, 13]. OPM positions membrane proteins in a hydrophobic slab model using energy minimization against experimentally calibrated transfer free energy parameters, producing membrane boundaries for over 15,000 protein entries. This approach is invaluable for understanding membrane protein topology, but the resulting labels reflect a coarse spatial localization: typically 20–40% of residues in a transmembrane protein fall within the OPM-defined membrane zone.

The second question, "Does this residue make direct physical contact with a lipid molecule?", requires experimental structural evidence. Crystallographic and cryo-electron microscopy structures frequently resolve lipid molecules at specific protein surface sites, revealing native lipid interaction partners at atomic resolution. These experimental contacts represent a far more stringent and specific annotation: in our dataset, only 1.00% of all residues in membrane proteins (including both surface-exposed and buried residues) are observed to make lipid contacts within 4.0 Å (using an all-atom distance criterion). However, this rate represents a lower bound on the true frequency of lipid contacts in biological membranes, because only a subset of native lipid interactions survive protein purification and are resolved in crystal or cryo-EM structures. Nonetheless, even as a lower bound, this rate is approximately 20- to 40-fold lower than the membrane zone positive rate, underscoring the specificity of the experimental lipid contact annotation.

This distinction matters for both biological interpretation and machine learning model development (Table 1). Membrane zone labels identify the general region of a protein that is embedded in the bilayer, while lipid contact labels pinpoint specific binding sites that may mediate functionally important lipid-protein interactions. For drug discovery, the latter is far more actionable: lipid contact sites represent potential targets for allosteric modulation by lipid-mimetic compounds

Table 1. Comparison of membrane zone and lipid contact prediction tasks. These represent fundamentally different biological questions with distinct label sources, positive rates, and downstream applications.

Aspect	Membrane zone	Lipid contact
Label source	Computational (OPM)	Experimental (PDB)
Biological question	In membrane slab?	Contacts lipid?
Observed positive rate	20–40%	∼1.00%†
Spatial resolution	Zone-level (∼30 Å)	Atomic (4.0 Å)
Validation	In silico energy	X-ray/cryo-EM
Key application	Topology prediction	Binding site identification

†Lower bound; the true lipid contact rate in vivo is likely substantially higher due to lipid loss during purification and incomplete resolution.

[14], while membrane zone annotations provide no such site-level resolution. We emphasize that no OPM-derived residue labels were used at any stage of the MPLID construction pipeline; all residue annotations derive exclusively from experimentally resolved lipid molecules in PDB structures. OPM is discussed here solely to distinguish the complementary biological questions of membrane positioning versus direct lipid contact.

Existing resources and their limitations

Several resources provide information about membrane protein-lipid interactions, each with specific scope and limitations. The OPM database [12] remains the definitive resource for membrane protein positioning, providing computationally derived membrane boundaries for transmembrane and peripheral proteins. However, OPM labels represent theoretical membrane zones rather than experimentally resolved lipid interaction sites.

The MemProtMD database [15, 16] complements OPM by providing coarse-grained molecular dynamics (CG-MD) simulations of membrane proteins embedded in explicit phospholipid bilayers. While MemProtMD offers valuable insights into lipid organization around proteins, its contacts derive from simulations rather than experimental structures, and the database focuses on integral membrane proteins in a single lipid type (dipalmitoylphosphatidylcholine).

The MemBlob database and server [17] takes a complementary approach by analyzing cryo-EM density maps to computationally identify membrane-facing regions of transmembrane proteins. MemBlob classifies residues as solvent-facing, lipid-facing, at the lipid interface, or buried, providing a membrane environment annotation that bridges between the coarse OPM slab model and direct experimental contact data. While MemBlob provides valuable residue-level environment classification, its labels are derived from density map analysis rather than from resolved lipid molecules, and the database is limited to proteins with available cryo-EM reconstructions.

BioDolphin [18] identifies and catalogs all protein-lipid interactions across the PDB using the PLIP (Protein-Ligand Interaction Profiler) framework [19], providing detailed interaction-type annotations (hydrogen bonds, hydrophobic contacts, salt bridges) at the residue and atom level. BioDolphin offers API access and covers a broad range of

interaction types, making it useful for detailed mechanistic analysis of individual protein-lipid complexes. MPLID differs from BioDolphin in its focus on providing pre-computed, ML-ready binary contact labels with cluster-aware train/validation/test splits, continuous distance values, and FAIR-compliant dataset deposition, rather than on-demand interaction profiling.

The STING Relational DataBase (RDB) [20, 21] provides a comprehensive structural analysis platform that computes and stores inter-atomic contacts, surface accessibility, and residue-level descriptors for all PDB structures. While STING RDB does not specifically label lipid contacts, its contact calculation infrastructure and residue-level annotation framework informed the design philosophy of MPLID, particularly regarding the use of distance-based contact definitions and the importance of providing both binary labels and continuous distance values for downstream analysis.

On the prediction methodology side, the DREAMM method [22] demonstrated that ensemble machine learning can achieve a Matthews correlation coefficient (MCC) of 0.84 for predicting protein-membrane interfaces, using a curated set of 54 peripheral membrane proteins (later expanded to 65) with experimentally characterized membrane-penetrating residues. However, this training data was distributed as part of the prediction tool rather than as a standalone, documented dataset resource. More recently, PMIpred introduced a physics-informed transformer approach trained on molecular dynamics data for peptide-membrane interactions [23], and protein language model approaches have been explored using ProtTrans embeddings [24, 25]. While these studies have advanced prediction methodology, none provides a large-scale, publicly deposited, and documented dataset covering the full diversity of membrane protein classes.

The need for large-scale experimental lipid contact data

The convergence of three trends motivates the creation of MPLID. First, the rapid growth of the Protein Data Bank [26, 27], which now contains more than 230,000 structures, means that thousands of membrane protein structures with resolved lipid molecules are available for systematic analysis. Second, advances in protein language models such as ESM-2 [28] and ProtTrans [25] have demonstrated that per-residue embeddings trained on large-scale protein sequence data can capture structural and functional properties, but training supervised models on top of these representations requires large labeled datasets. Third, structure-guided drug discovery increasingly recognizes lipid binding sites as druggable targets for allosteric modulation [14, 6], creating demand for systematic catalogs of experimentally validated lipid interaction sites.

MPLID addresses these needs by providing experimentally validated lipid contact annotations for 4,704 membrane proteins containing 8,055,325 residues. All labels derive from crystallized lipid molecules in PDB structures, providing experimental ground truth at atomic resolution. While existing resources offer related information, including membrane environment classification from cryo-EM density (MemBlob [17]) and detailed interaction-type profiling (BioDolphin/PLIP [18, 19]), none provides a pre-computed, ML-ready residue-level dataset with cluster-aware evaluation splits and FAIR-compliant deposition. MPLID fills this gap by covering integral, peripheral, and single-pass membrane proteins across 813 sequence-diverse clusters.

Data Description

Dataset overview

MPLID provides residue-level binary annotations indicating whether each residue in a membrane protein makes direct physical contact with an experimentally resolved lipid molecule. The complete dataset encompasses 4,704 membrane proteins with a total of 8,055,325 residues, of which 80,439 (1.00%) are labeled as lipid contacts (Figure 1). This observed positive rate should be interpreted as a lower bound on the true frequency of protein-lipid contacts in biological membranes: many native lipid interactions are lost during protein purification and crystallization, and most PDB structures resolve only a small number of tightly bound or structurally ordered lipids. Consequently, the non-contact class (99.00%) inevitably contains a substantial proportion of false negatives, residues that contact lipids in vivo but lack a resolved lipid partner in the crystal or cryo-EM structure. The remaining residues classified as non-contacts include solvent-exposed domains, protein-protein interfaces, interior regions of multi-subunit complexes, and an unknown fraction of unresolved lipid-binding sites.

Proteins in the dataset range from 22 to 15,280 residues (median 1,038; mean 1,712), reflecting the diversity of membrane protein sizes from small single-pass transmembrane peptides to large multi-subunit complexes. The number of lipid contacts per protein ranges from 1 to 4,820 (median 4; mean 17), with the highly skewed distribution reflecting that most proteins have few resolved lipid contacts while a small number of well-characterized complexes have extensive lipid interactions. The maximum value (4,820 contacts for PDB 6W4F, an NMR-resolved structure of the KRAS4B homodimer on a lipid bilayer nanodisc) arises because the current pipeline counts residue-lipid contacts across all NMR conformational models deposited in the PDB coordinate file; 6W4F contains 20 NMR models, each with approximately 240 contact residues. A total of 41 NMR multi-model structures in the dataset are affected by this counting convention, contributing approximately 24,000 excess contact annotations (see Limitations). Every protein in the final dataset has at least one lipid contact, as proteins with zero contacts were removed during quality filtering (see Methods). This removal excludes membrane proteins whose lipids were stripped during purification, meaning that MPLID does not represent the complete membrane proteome and cannot serve as a source of proteome-wide negative controls.

Data schema and format

Each record in MPLID contains ten fields that provide complete information for each annotated residue. The `pdb_id` field contains the four-character PDB accession code, and `chain_id` specifies the polypeptide chain within the structure. The `residue_number` gives the residue's position in the chain, while `residue_name` provides the three-letter amino acid code. The binary label `is_contact` takes a value of 1 for residues where any heavy atom is within 4.0 Å of any lipid heavy atom and 0 otherwise. The `label_source` field is set to "EXPERIMENTAL" for all entries, confirming that labels derive from experimentally resolved lipid molecules in PDB structures rather than computational predictions. The `confidence` field is set to "high" for all entries in the current release because all labels derive from experimentally resolved structures; this field is retained as a placeholder for future quality scoring that may incorporate

structure resolution and B-factor data. The `min_distance` field records the minimum distance in Angstroms between any heavy atom of the residue and the nearest lipid heavy atom. Finally, `cluster_id` assigns each protein to a sequence cluster, and `split` indicates assignment to training, validation, or test partitions.

Data are distributed as gzip-compressed comma-separated values (CSV) files partitioned by split (train, validation, test), along with a protein-level metadata file containing per-protein summary statistics including residue counts, contact counts, contact rates, cluster assignments, and split assignments.

Train/validation/test splits

The dataset is partitioned into training (2,578 proteins; 4,907,696 residues), validation (1,051 proteins; 1,403,838 residues), and test (1,075 proteins; 1,743,791 residues) splits (Figure 1). Critically, splitting is performed at the protein level with cluster-aware stratification: proteins within the same sequence cluster are always assigned to the same split, preventing data leakage from sequence-similar proteins appearing in both training and evaluation partitions. Contact rates vary across splits (training 1.16%, validation 0.69%, test 0.79%) because stratification is performed at the cluster level rather than the residue level, and clusters with higher contact rates may be unevenly distributed. This variation is expected and should be accounted for when comparing model performance across splits. This design choice is essential for meaningful generalization assessment, as protein-level leakage can dramatically inflate performance estimates in structural bioinformatics tasks.

The cluster-aware splitting strategy uses MMseqs2 [29] with a 30% sequence identity threshold and 80% bidirectional alignment coverage, producing 813 clusters. Entire clusters are assigned to splits to ensure that no protein in the validation or test sets shares more than 30% sequence identity with any training protein. This threshold is substantially more conservative than the 40% identity commonly used in protein function prediction benchmarks [30], further reducing the risk of overestimating generalization performance.

Sequence clustering analysis

The 813 sequence clusters exhibit a highly skewed size distribution (Figure 2), with 373 singletons (45.9%) and a largest cluster of 173 proteins, reflecting the sequence diversity of membrane proteins across different families and superfamilies. This clustering pattern indicates substantial structural and functional diversity in the dataset, as singleton clusters represent unique membrane protein families without close homologs.

Analyses

Amino acid composition at lipid contact sites

Analysis of amino acid enrichment at lipid contact sites reveals biologically meaningful and interpretable patterns (Figure 3). Enrichment ratios were calculated as the fraction of each amino acid among contact residues divided by its fraction in the complete dataset. Tryptophan (1.88×) shows the strongest enrichment, consistent with the well-established role of aromatic residues in anchoring transmembrane proteins at the membrane-water interface through favorable interactions with lipid headgroups via cation-π and hydrogen bonding interactions [4]. Notably, positively charged residues are strongly enriched: arginine (1.44×) and lysine (1.33×) rank among the top five most enriched amino acids, consistent with the "positive-inside rule" [31] and with the electrostatic attraction of cationic side chains to anionic phospholipid headgroups. Glycine (1.36×) is enriched, reflecting its prevalence in GxxxG motifs that mediate transmembrane helix-helix packing and create lipid-accessible surfaces at helix interfaces [32]. Phenylalanine (1.23×) and tyrosine (1.08×) are moderately enriched, contributing to the aromatic belt. Among other residues, cysteine (1.15×), serine (1.13×), and histidine (1.11×) show mild enrichment, with histidine functioning as an interfacial anchoring residue through its amphipathic imidazole ring, and cysteine enrichment likely reflecting palmitoylation sites and disulfide-constrained loop regions at lipid interfaces. Leucine (1.08×) is slightly enriched, reflecting its involvement in direct side-chain contacts with lipid acyl chains.

Among depleted residues, proline (0.51×) and isoleucine (0.57×) show the strongest depletion. Proline disrupts regular secondary structure and is underrepresented at smooth lipid-protein interfaces. The depletion of isoleucine, a β-branched hydrophobic residue, may reflect its preference for buried core positions rather than surface lipid contacts. Aspartate (0.59×) is depleted, consistent with the electrostatic incompatibility of negatively charged side chains with the lipid bilayer interior. Asparagine (0.71×) and valine (0.73×) are moderately depleted. Threonine (0.87×) and glutamine (0.89×) show mild depletion, while alanine (0.94×) and glutamate (0.97×) are near neutral. Overall, the enrichment pattern strongly supports the biological validity of the all-atom contact definition: residues capable of electrostatic interactions with phospholipid headgroups (arginine, lysine) and aromatic anchoring at the interface (tryptophan, phenylalanine) are enriched, while conformationally constrained (proline) and β-branched (isoleucine, valine) residues are depleted. These patterns are consistent with previous analyses of cardiolipin binding site composition [33] and likely reflect, in part, the overrepresentation of anionic lipids (particularly cardiolipin and phosphatidylglycerol) among structurally resolved lipid species. Because anionic lipids are more readily co-purified and crystallized with membrane proteins than zwitterionic species such as phosphatidylcholine and phosphatidylethanolamine, the observed enrichment of positively charged residues may be amplified relative to the in vivo membrane environment, where the majority of lipids are zwitterionic. Given the large sample sizes (80,439 contact residues among 8,055,325 total residues), these enrichment and depletion patterns are robust and unlikely to arise from sampling variation, but their magnitude may be influenced by the lipid composition bias inherent to crystallographic datasets. A sensitivity analysis excluding all 46 NMR multi-model structures confirms that the core enrichment pattern is preserved (Pearson $r = 0.86$; Spearman $\rho = 0.80$, $p = 2.3 \times 10^{-5}$; Supplementary Analysis S2).

Distance distribution analysis

The distribution of minimum distances between protein residues and lipid molecules provides independent validation of the 4.0 Å contact cutoff (Figure 4). Among contact residues, the distance distribution shows a clear peak at approximately 3.5–4.0 Å, corresponding to typical van der Waals contact distances between heavy atoms. The sharp boundary at 4.0 Å indicates that the cutoff effectively captures the transition from direct physical contact to non-contact. For non-contact residues, the

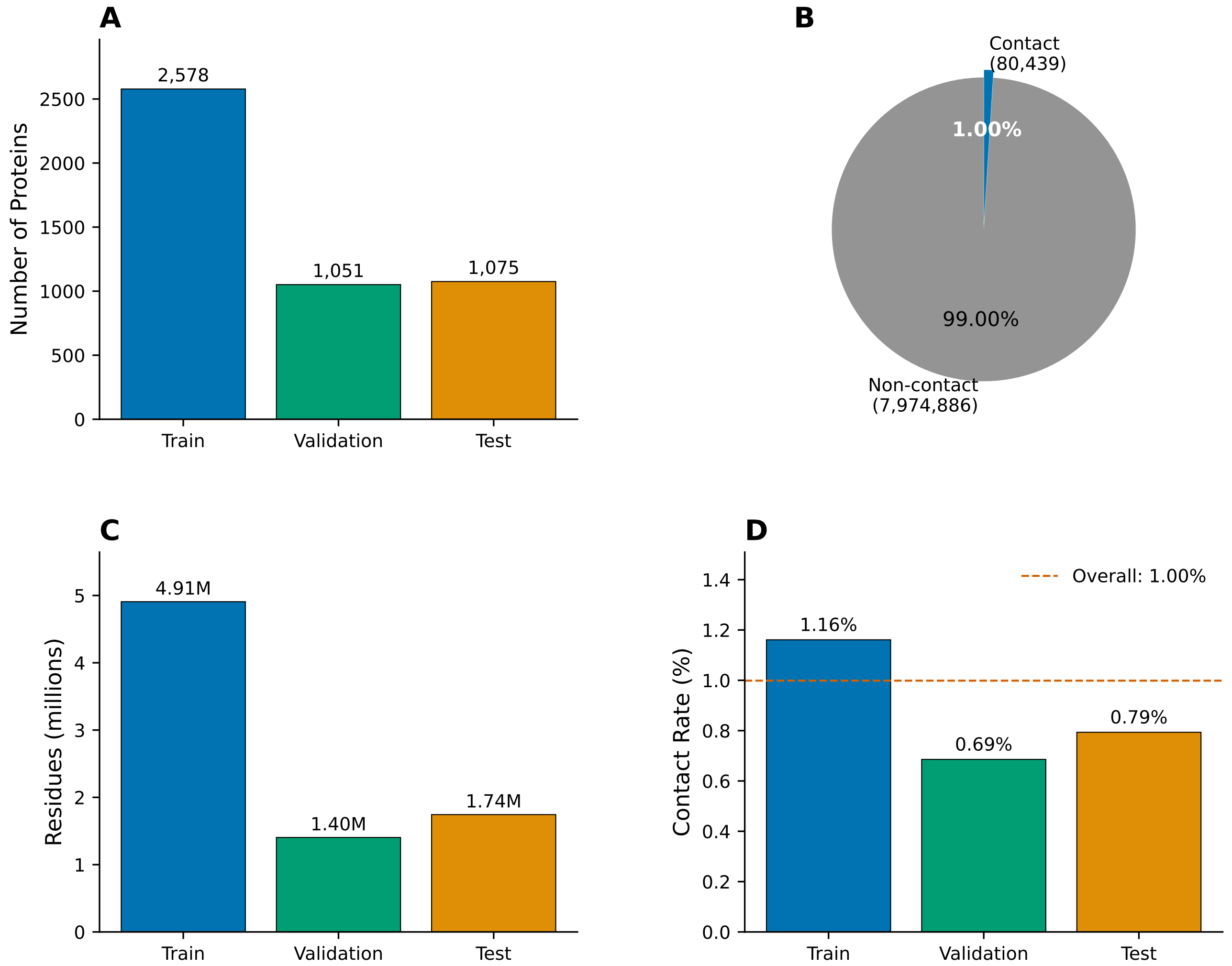

Fig. 1. MPLID dataset overview. (A) Distribution of proteins across training (54.8%), validation (22.3%), and test (22.9%) splits. (B) Class distribution showing the imbalance between contact (1.00%) and non-contact (99.00%) residues. (C) Total residues per split. (D) Contact rates across splits, showing variation due to cluster-level stratification: training (1.16%), validation (0.69%), and test (0.79%).

distribution is broadly shifted toward larger distances, with a peak near 8–12 Å and a long tail extending to distances greater than 30 Å for residues in soluble domains far from any lipid molecule.

Protein size and contact density distributions

The distribution of protein sizes in MPLID spans three orders of magnitude, reflecting the diversity of membrane protein families represented (Figure 5). Contact density, defined as the fraction of residues making lipid contacts, varies substantially across proteins, with a per-protein median contact rate of 0.45% and a mean of 0.99%. The observed contact rate of 1.00% reflects both the sparse resolution of lipid molecules in PDB structures and the inherent specificity of direct van der Waals contacts. This rate should be interpreted as a lower bound; the severe class imbalance (approximately 100:1 negative-to-positive ratio) includes a substantial fraction of false negatives in the non-contact class, where residues that contact lipids in vivo lack a resolved lipid partner in the experimental structure. This imbalance requires specialized machine learning approaches such as class weighting or focal loss.

Comparison with existing resources

Table 2 places MPLID in the context of existing membrane protein-lipid interaction resources (Figure 6). Each resource addresses a different aspect of the problem. OPM [12] provides computationally derived membrane zone boundaries for topology analysis. MemProtMD [15] offers coarse-grained simulation data for lipid organization. The DREAMM prediction method [22] used a curated set of 54 peripheral membrane proteins with experimentally characterized membrane-penetrating residues as training data, though this data was not released as a standalone documented dataset. MPLID fills this gap by providing the first large-scale, publicly deposited, and fully documented dataset of experimental lipid contact labels, covering 4,704 proteins across all membrane protein classes with FAIR-compliant metadata, a data dictionary, and pre-defined evaluation splits. The diversity of membrane protein families represented in MPLID, spanning 813 sequence clusters at 30% identity, ensures broad coverage of the membrane protein structural landscape. These resources are complementary rather than competing, each addressing a different level of the membrane protein-lipid interaction hierarchy.

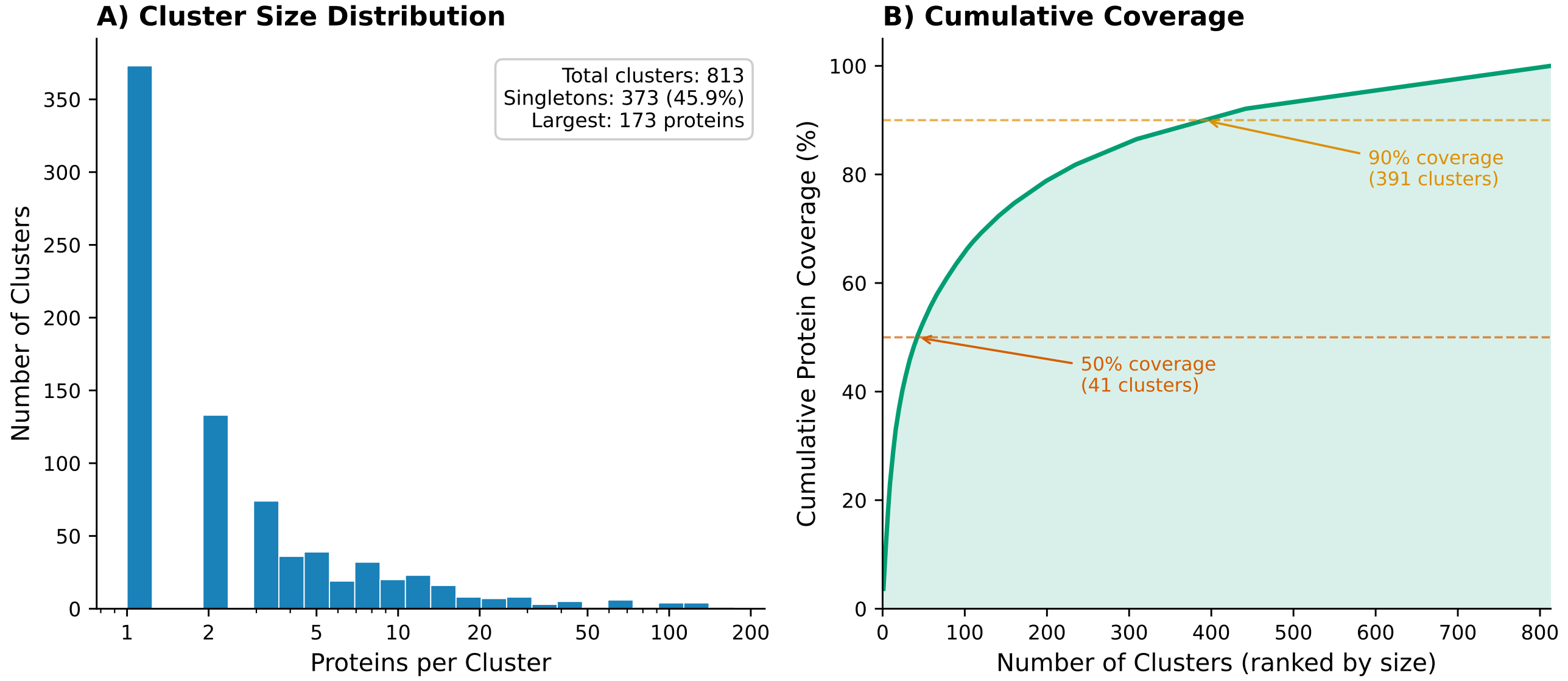

Fig. 2. Sequence cluster analysis. (A) Distribution of cluster sizes at 30% sequence identity on a log scale, showing a highly skewed size distribution with many singletons and a small number of large clusters containing many homologous structures. (B) Cumulative protein coverage ranked by cluster size, illustrating the concentration of proteins in a few large families.

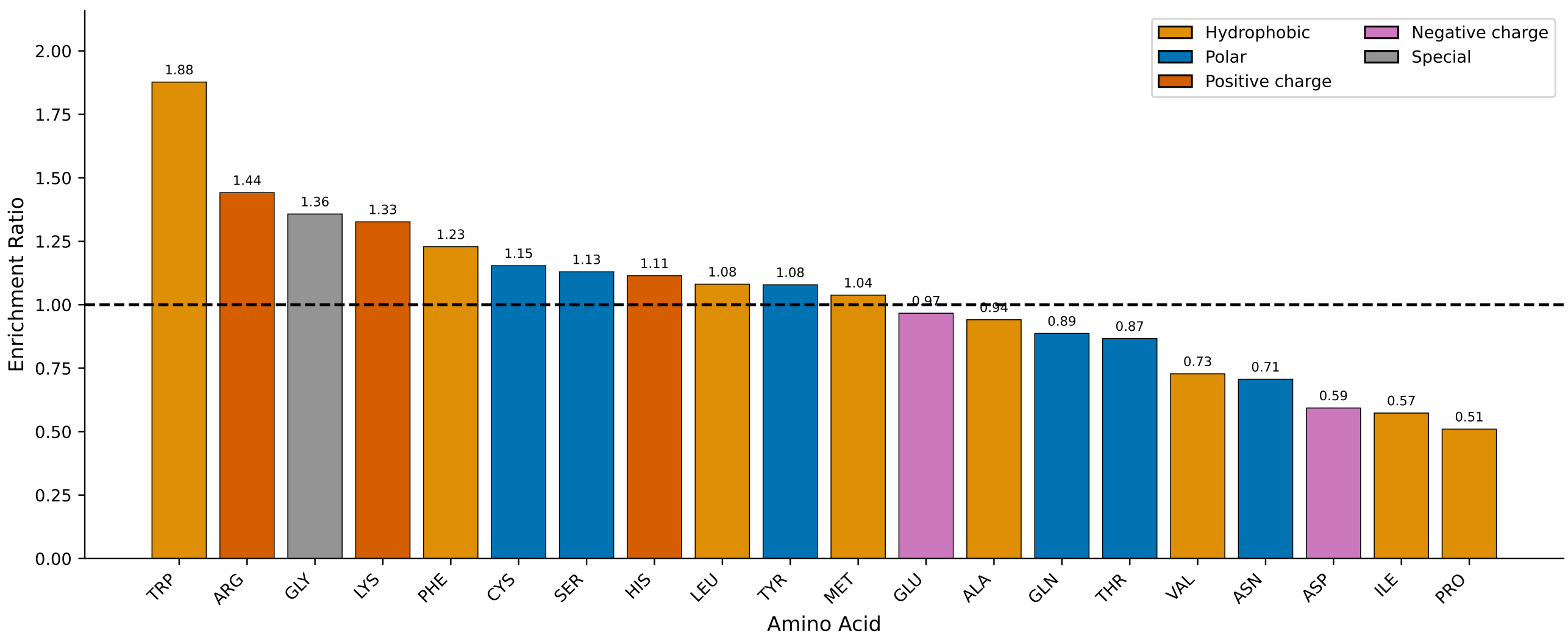

Fig. 3. Amino acid enrichment at lipid contact sites. Enrichment ratios are calculated as the frequency of each amino acid among contact residues divided by its overall frequency in the dataset. Colors indicate amino acid physicochemical properties: hydrophobic (orange), polar (blue), positive charge (red), negative charge (purple), and special (gray). The dashed line marks the expected frequency (1.0). Values above 1.0 indicate enrichment; values below 1.0 indicate depletion.

Table 2. Comparison of MPLID with existing membrane protein-lipid interaction resources. MPLID is the first large-scale, publicly deposited dataset providing experimentally validated residue-level lipid contact labels with FAIR-compliant documentation.

Resource	Proteins	Residues	Label type	Label source
MPLID (this work)	4,704	8,055,325	Lipid contact	Experimental (PDB)
BioDolphin [18][b]	PDB-wide	–	Interaction type	Experimental (PLIP)
MemBlob [17][c]	∼500	–	Membrane environment	Cryo-EM density
DREAMM [22][a]	54	∼15,000	Membrane interface	Experimental (curated)
OPM [12]	15,096	–	Membrane zone	Computational
MemProtMD [15]	∼3,500	–	Lipid contact	Simulated (CG-MD)

[a]Training data distributed within the DREAMM prediction tool repository; not released as a standalone documented dataset. [b]On-demand interaction profiling via PLIP; not pre-computed ML-ready dataset with splits. [c]Residue-level membrane environment classification from cryo-EM density maps.

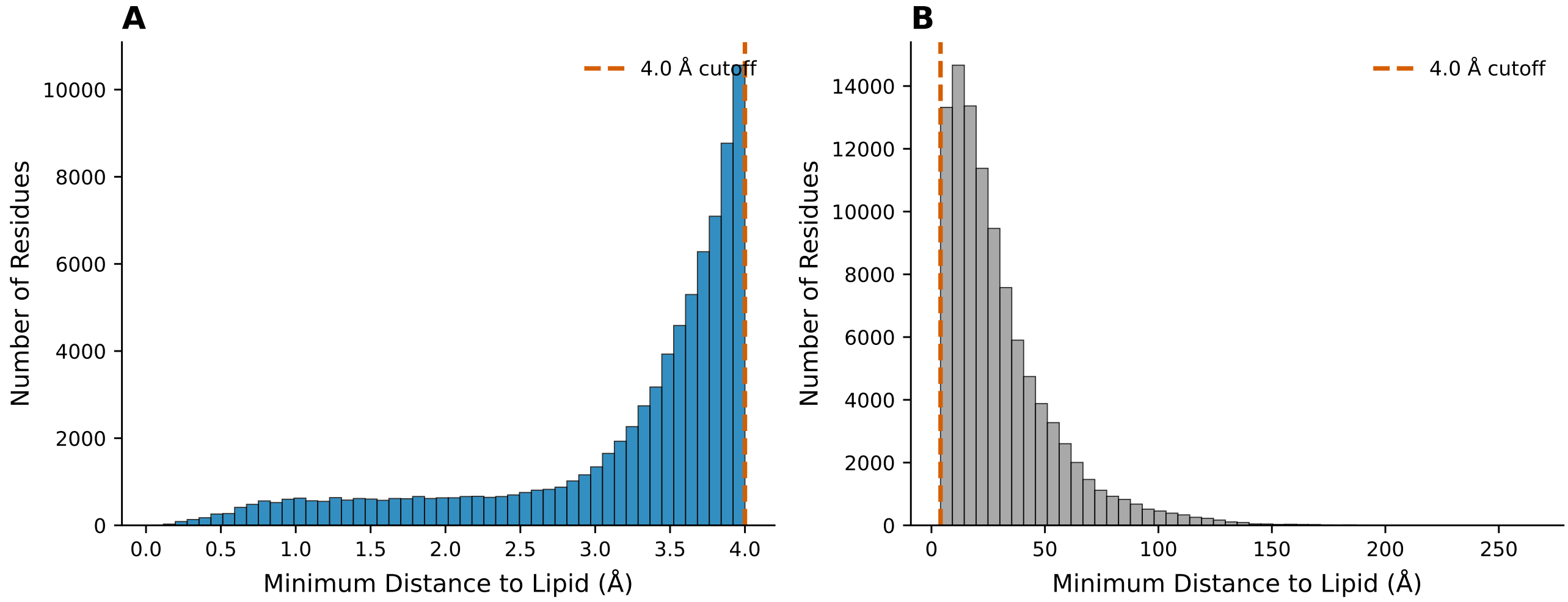

Fig. 4. Distribution of minimum residue-to-lipid distances. (A) Contact residues (≤4.0 Å) show a peak near 3.5–4.0 Å, consistent with van der Waals contact distances. (B) Non-contact residues display a broad distribution with distances extending well beyond 30 Å, confirming clear separation between contacting and non-contacting populations.

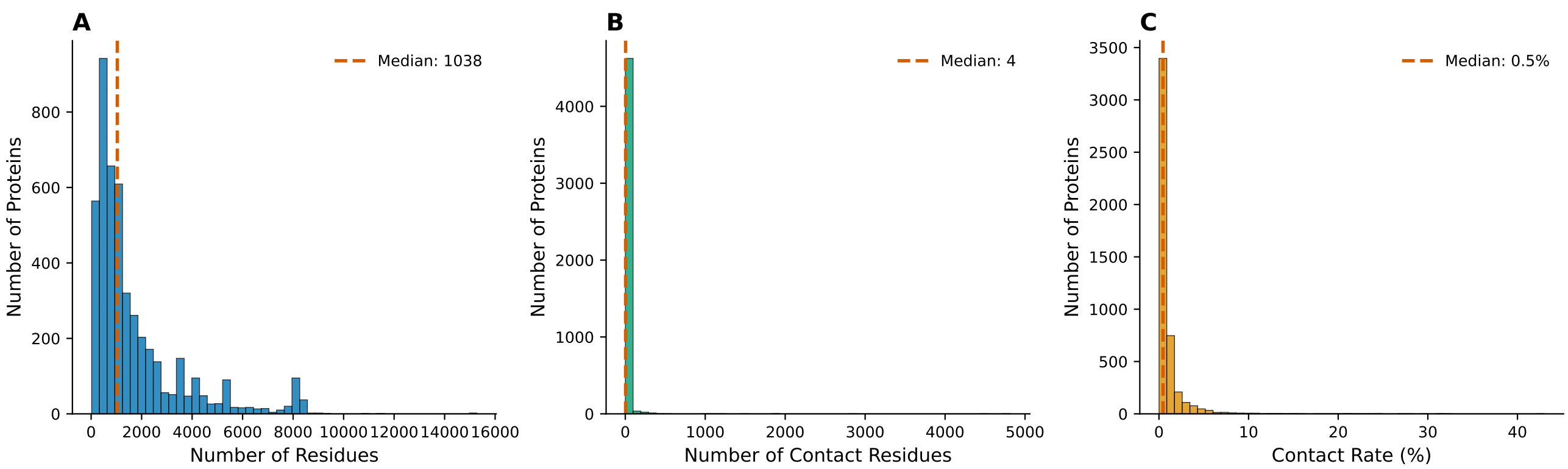

Fig. 5. Protein-level statistics. Distribution of protein sizes and contact densities across the MPLID dataset, illustrating the diversity of membrane protein families represented.

In practice, models trained on one type of label (e.g., OPM membrane zone) cannot be directly evaluated on another (e.g., MPLID lipid contact), and researchers should select the appropriate resource based on their specific biological question.

Lipid type diversity

MPLID uses a curated list of 117 candidate lipid identifiers organized into ten functional categories, comprising 90 PDB-derived ligand codes and 27 CHARMM-style lipid identifiers commonly found in cryo-EM depositions. All 90 PDB-derived codes were audited against the RCSB Chemical Component Dictionary API during revision; the complete lipid ontology with RCSB verification status, official chemical names, and category assignments is provided in Supplementary Table S1. Phospholipids include cardiolipin (CDL, found in 675 structures), phosphocholine variants (PCW, 196 structures), phosphoethanolamine (PEE, 273 structures), and phosphoglycerol (PGV, 144 structures). Sterols are represented by cholesterol (CLR, 1,119 structures) and derivatives including cholic acid (CHD, 150 structures; a bile acid sterol derivative) and cholesterol hemisuccinate (Y01). Fatty acids include palmitate (PLM, 734 structures), oleic acid (OLA, 379 structures), myristate (MYR, 426 structures), and stearate (STE, 94 structures). Sphingolipids include sphingosine (SPH, 102 structures) and sphingosine-1-phosphate (S1P, 16 structures). Detergent mimetics used in crystallization, which often occupy native lipid binding sites, include dodecyl β-D-maltoside (LMT, 677 structures), octyl β-D-glucopyranoside (BOG, 510 structures), and lauryldimethylamine oxide (LDA, 263 structures). The protein-level metadata includes an explicit `has_detergent_only` flag indicating whether all lipid contacts for a given protein derive exclusively from detergent mimetics rather than physiological lipid species, enabling users to filter detergent-only entries if desired.

Notably, several biologically important lipid classes are absent or underrepresented in the current MPLID release. Phosphatidic acid (PA), phosphatidylserine (PS), and phosphoinositides (PI, PIP, PIP_2, PIP_3) are not represented among the recognized lipid codes, despite their well-established roles in membrane protein regulation. This absence reflects both the difficulty of resolving these lipids crystallographically (owing to their flexible headgroups and transient binding modes) and the focus of MPLID on lipid species that are

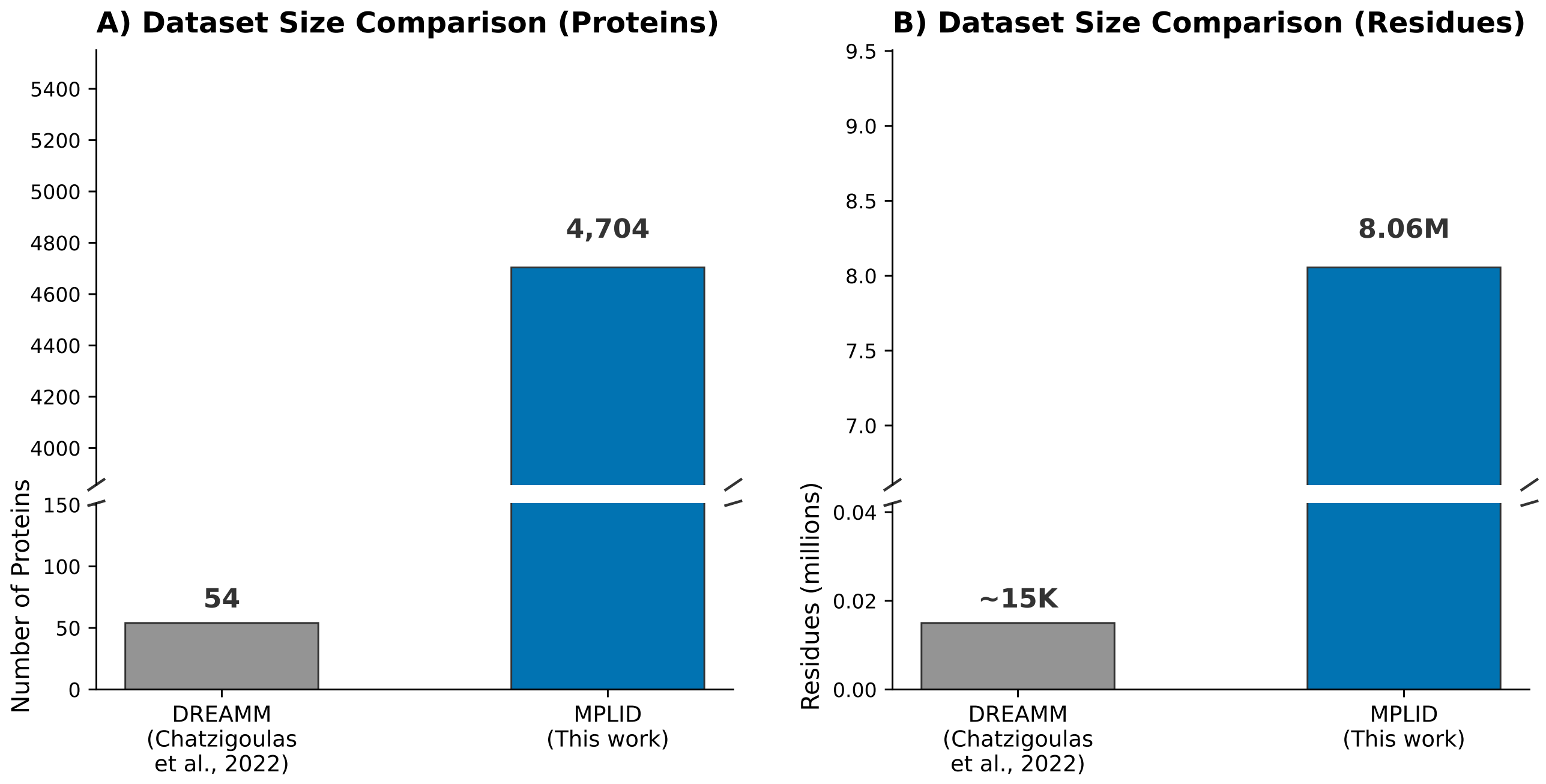

Fig. 6. Scale comparison between MPLID and the training data used by the DREAMM prediction method [22], the largest prior collection of experimentally annotated membrane protein-lipid interactions. (A) Number of proteins (4,704 versus 54). (B) Number of annotated residues (8,055,325 versus ∼15,000). Note that these resources differ in scope: DREAMM focuses on peripheral membrane proteins, while MPLID covers all membrane protein classes.

currently represented in PDB HETATM records. Similarly, polyunsaturated fatty acids (e.g., docosahexaenoic acid, arachidonic acid) are rarely resolved in crystal structures. Future releases will expand lipid coverage as more structures with these lipid types become available in the PDB.

Structural visualization of lipid contacts

To illustrate the nature of the lipid contact annotations in MPLID, representative membrane protein structures with their resolved lipid molecules are shown in Figure 7. These examples demonstrate that lipid contacts occur at specific, well-defined sites on the protein surface rather than uniformly across the membrane-exposed region. Contact residues (highlighted in red/orange) are interspersed with non-contact residues even in transmembrane regions, confirming that lipid contact prediction is a more specific task than membrane zone classification.

Computational label comparison studies

The availability of MPLID alongside OPM membrane zone annotations enables systematic comparison of experimental lipid contacts with computational membrane boundaries. We compared MPLID contact labels with OPM-derived membrane zone assignments across proteins present in both resources (Figure 8). The number of overlapping proteins varies by analysis: 2,383 proteins contributed to precision estimates, 1,685 to recall estimates, and 2,457 to contact distribution analysis, reflecting differences in the availability of OPM membrane zone annotations and non-zero lipid contacts across the shared set. Of all MPLID lipid contacts in the shared set (n = 28,497 residues), 51.2% fell within the OPM membrane zone while 48.8% were located outside the computationally defined boundaries, likely reflecting interfacial contacts, re-entrant loops, or amphipathic helices that extend beyond the hydrophobic core. Conversely, 98.8% of OPM membrane zone residues (n = 1,170,590) showed no direct lipid contact within 4.0 Å, and the per-protein median was only 0.7% of membrane zone residues having lipid contacts. The OPM membrane zone captured a median of 50.0% of lipid contacts per protein. These dataset-wide statistics highlight the fundamental difference between membrane zone classification and lipid contact prediction: being embedded in the membrane is neither sufficient nor necessary for direct lipid interaction. Researchers working with MemProtMD [15] simulations can similarly compare simulated lipid contacts with the experimental contacts provided by MPLID.

Methods

Data source integration

MPLID was constructed using a purely experimental approach with no dependence on computational membrane positioning databases. OPM data were not used at any stage of the MPLID construction pipeline; OPM is discussed in this paper solely to contextualize the complementary biological questions of membrane zone positioning versus direct lipid contact annotation. All residue annotations in MPLID derive exclusively from experimentally resolved lipid molecules in PDB structures. The RCSB Protein Data Bank [26, 27] was queried programmatically for all structures containing recognized lipid chemical components, identifying 8,221 candidate structures with lipid ligands. This query-first strategy ensures that every protein in the dataset has at least one experimentally resolved lipid molecule, providing broad structural coverage of experimentally resolved protein-lipid interactions across diverse membrane protein classes, while still reflecting the

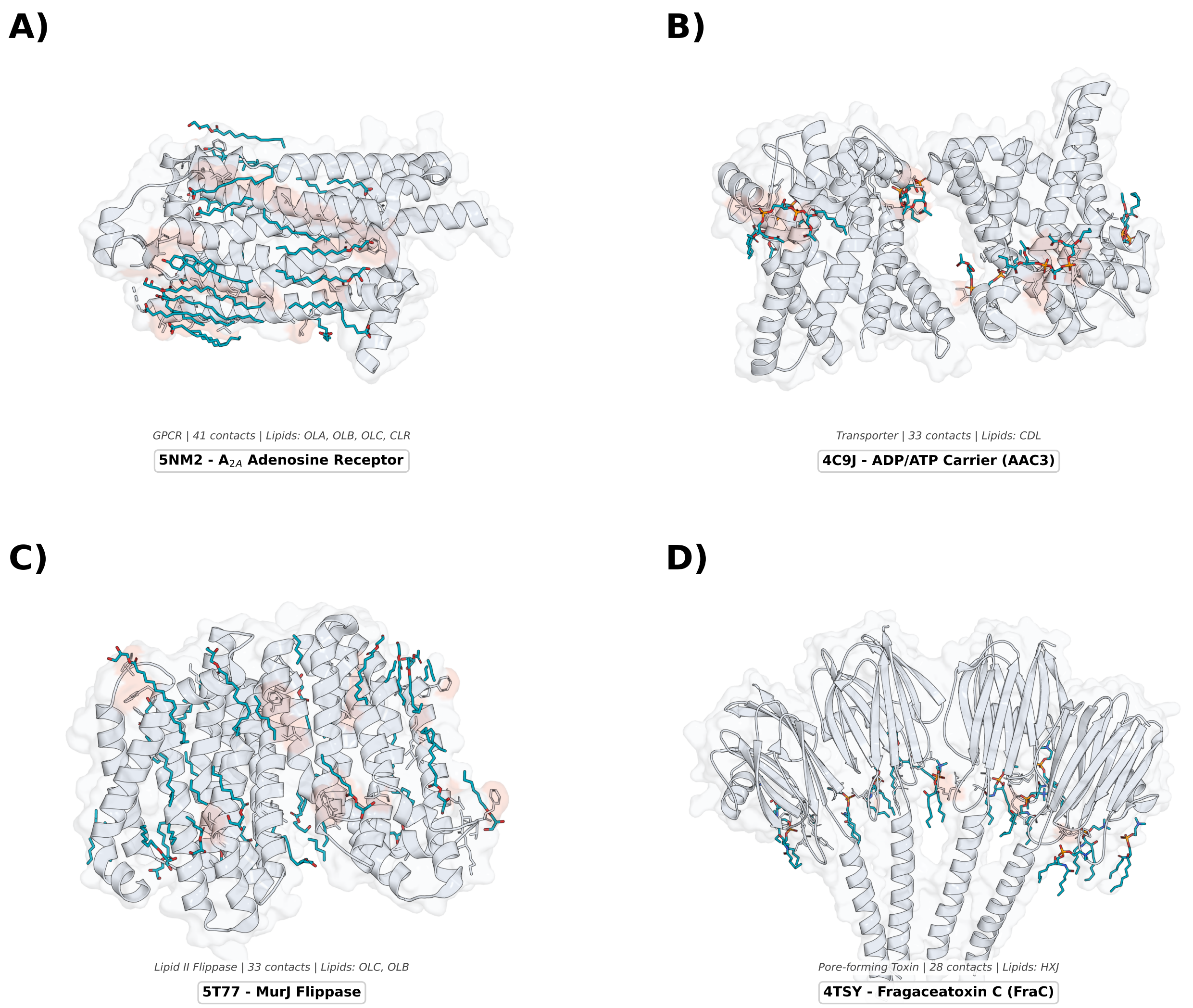

Fig. 7. Structural visualization of MPLID lipid contact annotations across four representative membrane protein classes. (A) A_{2A} adenosine receptor (PDB: 5NM2), a GPCR with multiple lipid types including oleic acid and cholesterol. (B) ADP/ATP carrier AAC3 (PDB: 4C9J), a mitochondrial transporter with cardiolipin contact residues across two chains. (C) MurJ flippase (PDB: 5T77), a lipid II flippase with contacts distributed across its transmembrane helices. (D) Fragaceatoxin C (PDB: 4TSY), a tetrameric pore-forming toxin with sphingolipid contacts at the membrane insertion interface. These examples demonstrate that lipid contacts are site-specific rather than uniformly distributed across membrane-exposed surfaces.

known experimental biases of structural biology datasets. The identified structures were downloaded as original PDB coordinate files (9,796 files total, as some entries required retrieval of biological assembly files or alternate depositions). After parsing, 5,723 unique proteins yielded valid structures with recognized HETATM lipid records; the remainder comprised duplicate chains, structures that failed parsing, or entries lacking standard amino acid residues. These were processed through the contact calculation and quality filtering pipeline described in the following subsections.

Lipid molecule identification

Lipid molecules in PDB structures were identified using candidate ligand codes that were subsequently audited against the RCSB Chemical Component Dictionary. We compiled a list of 117 candidate lipid identifiers organized into ten functional categories: phospholipids (POV, PCW, PEE, PGV, and others), cardiolipin (CDL, C9V, 18W, LCL), sphingolipids (SPH, S1P, HXJ, and others), sterols (CLR, CHD, Y01, ERG), fatty acids (PLM, MYR, OLA, STE, DHA, and others), glycerolipids (TGL, DAG, MAG), detergent mimetics (LDA, LMT, BOG, OLC, DPC), lipid A components (LPA, KDO, 6LP), and CHARMM simulation nomenclature (POPC, POPE, POPG, and others commonly found in cryo-EM depositions). This list was developed iteratively by cross-referencing the PDB Chemical Component Dictionary with lipid classification databases and manually curating entries to exclude non-lipid small molecules that might share similar chemical codes. Detergent mimetics were included because they frequently occupy native lipid binding sites in membrane protein crystal structures and provide relevant information about lipid-protein interaction geometry [7].

Structure processing and contact calculation

For each candidate protein, the original PDB coordinate file was downloaded from RCSB to preserve HETATM records

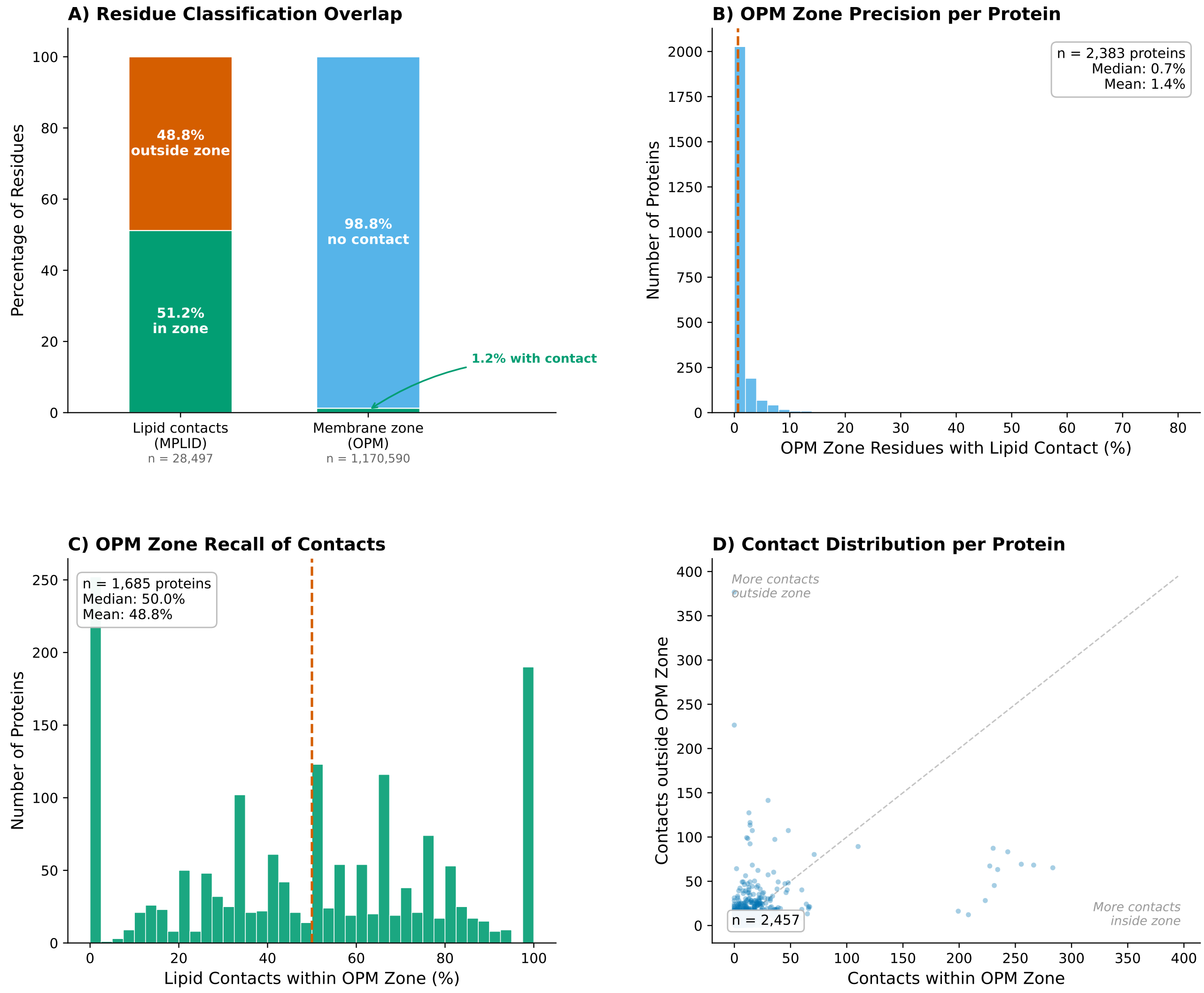

Fig. 8. Quantitative comparison of MPLID lipid contacts versus OPM membrane zone annotations. (A) Residue classification overlap: 48.8% of lipid contacts (n = 28,497) fall outside the OPM membrane zone, while 98.8% of OPM zone residues (n = 1,170,590) lack direct lipid contact. (B) Per-protein OPM precision (n = 2,383 proteins): median 0.7% of membrane zone residues have lipid contacts. (C) Per-protein OPM recall (n = 1,685 proteins): the membrane zone captures a median of 50.0% of lipid contacts. (D) Per-protein scatter of contacts inside versus outside the OPM zone (n = 2,457 proteins). These results demonstrate that membrane zone classification and lipid contact annotation address fundamentally different biological questions.

containing lipid molecule coordinates. Structure parsing was performed using BioPython [34], extracting all standard amino acid residues (ATOM records) and all recognized lipid molecules (HETATM records). Non-standard residues, water molecules, metal ions, and non-lipid ligands were excluded.

Lipid contacts were defined using an all-atom distance criterion. For each protein residue R and each lipid molecule L in the structure, the minimum distance $d_{\min}(R, L)$ between any heavy (non-hydrogen) atom of R and any heavy atom of L was calculated. A residue was labeled as a lipid contact if:

$$d_{\min}(R, L) = \min_{a \in R,\, b \in L} \|\mathbf{r}_a - \mathbf{r}_b\| \leq 4.0 \text{ Å} \qquad (1)$$

where $\mathbf{r}_a$ denotes the Cartesian coordinates of heavy atom a in residue R and $\mathbf{r}_b$ denotes the coordinates of heavy atom b in lipid L. The use of all heavy atoms (backbone and side chain) as residue representatives captures direct van der Waals contacts that would be missed by a Cα-only criterion, particularly for residues whose side chains extend into lipid-binding pockets while their backbone remains distant from lipid molecules. The 4.0 Å cutoff was chosen to capture direct van der Waals contacts between heavy atoms, consistent with standard distance thresholds used in protein-ligand interaction analysis [19], protein contact databases [20], and previous membrane protein-lipid interaction studies [22]. Typical van der Waals radii for carbon, nitrogen, and oxygen atoms sum to contact distances of 3.2–3.8 Å, and the 4.0 Å threshold provides a small margin to accommodate thermal displacement while remaining well below the threshold for indirect (water-mediated or electrostatic) interactions. The distance distribution analysis (Figure 4) independently validates this choice, showing a clear peak at 3.5–4.0 Å corresponding to van der Waals contacts. We note that this cutoff does not distinguish between contacts with lipid headgroups and acyl chains; both are captured by the all-atom criterion. Researchers interested in headgroup-specific or acyl-chain-specific contacts can use the continuous `min_distance`

values together with the lipid atom identities available in the original PDB coordinate files. For each residue, the minimum all-atom distance to the nearest lipid heavy atom was recorded regardless of contact status, providing a continuous distance metric that enables researchers to evaluate alternative cutoff values.

Quality filtering

Of the 5,723 proteins with parseable lipid-containing structures, 1,019 were removed because they contained only non-lipid molecules misannotated as lipids (such as HEPES buffer and bacteriochlorophyll) or had all lipid atoms beyond the 4.0 Å contact threshold, leaving 4,704 proteins with at least one genuine lipid contact. This filtering ensures that every protein in the final dataset has genuine, experimentally resolved lipid contacts.

Sequence clustering

To prevent data leakage between training, validation, and test splits, all 4,704 proteins were clustered using MMseqs2 [29] with a 30% sequence identity threshold and 80% bidirectional alignment coverage. These stringent thresholds ensure that proteins assigned to different splits are sufficiently divergent in sequence to represent independent test cases. The clustering produced 813 clusters.

Split strategy

Clusters were assigned to training, validation, and test splits using stratified random sampling at the cluster level, with stratification based on the average contact rate per cluster. This approach ensures that each split contains a representative distribution of high-contact and low-contact proteins. Entire clusters were assigned to a single split, guaranteeing that no two proteins sharing more than 30% sequence identity appear in different partitions. The resulting splits contain 2,578 training proteins (54.8%), 1,051 validation proteins (22.3%), and 1,075 test proteins (22.9%).

Software and computational environment

The MPLID construction pipeline was implemented in Python 3.11.8 using BioPython 1.83 [34] for PDB structure parsing, pandas 2.2.2 [35] for data manipulation, NumPy 1.26.4 for numerical computation, SciPy 1.13 for distance calculations, and scikit-learn 1.7.2 [36] for data splitting. MMseqs2 version 15 [29] was used for sequence clustering. The RCSB PDB search API was used for programmatic identification of structures containing lipid chemical components.

Discussion

MPLID represents the first large-scale, publicly deposited dataset of experimentally validated residue-level lipid contacts for membrane proteins, complementing existing resources such as OPM (membrane zone positioning), MemBlob (membrane environment inference from cryo-EM density), and BioDolphin/PLIP (generalized structural interaction profiling). The dataset addresses a biological question that is fundamentally distinct from membrane zone classification: rather than identifying residues located within a computationally defined hydrophobic slab, MPLID annotates residues that make direct physical contact with experimentally resolved lipid molecules at atomic resolution. Importantly, MPLID captures only the subset of protein-lipid interactions that survive purification and are resolved in crystal or cryo-EM structures; biological protein-lipid interactions are therefore broader than the structurally resolved set. The analyses presented here validate this annotation approach through multiple independent lines of evidence. The amino acid enrichment patterns at lipid contact sites are biologically consistent, with aromatic anchoring residues (tryptophan, phenylalanine) and positively charged residues (arginine, lysine) enriched at contact sites, while conformationally constrained (proline) and negatively charged (aspartate) residues are depleted, consistent with independent analyses of cardiolipin binding sites [33]. The distance distribution analysis confirms that the 4.0 Å cutoff effectively captures the van der Waals contact boundary. The quantitative comparison with OPM membrane zone annotations demonstrates that nearly half of experimentally observed lipid contacts fall outside computationally predicted membrane boundaries, reinforcing the complementary nature of these resources.

The scale of MPLID, comprising 4,704 proteins across 813 sequence clusters, provides substantially greater coverage than prior experimental lipid contact datasets. The DREAMM training set [22], the largest previously available collection, contained 54 peripheral membrane proteins. MPLID expands this by nearly two orders of magnitude while covering all membrane protein classes, including integral, peripheral, and single-pass proteins. The cluster-aware splitting strategy, using a conservative 30% sequence identity threshold, ensures that evaluation on the test set reflects genuine generalization rather than sequence memorization.

Limitations

Several limitations should be noted when using MPLID.

False negatives in the non-contact class.

Lipid contact labels depend on the crystallographic or cryo-EM resolution and completeness of resolved lipid molecules; many native lipid binding events are not captured because lipids are disordered or stripped during purification and crystallization. Consequently, the false negative rate in the non-contact class is likely substantial, potentially exceeding the number of true positive contacts by an order of magnitude. The true frequency of lipid contacts in membrane proteins in vivo is almost certainly higher than the 1.00% observed in MPLID. Moreover, many structures contain only one or a few resolved lipid molecules, meaning that the observed contacts represent a sparse snapshot of the full lipid-binding landscape rather than a comprehensive map of all possible interaction sites. Users training machine learning models on MPLID should be aware that the non-contact class is noisy and that model performance may be limited by false negative labels rather than by model capacity.

Detergent mimetics.

The inclusion of detergent mimetics as proxy lipids introduces potential noise, as detergent binding sites may not perfectly recapitulate native lipid interactions. Analysis of HETATM records across all 4,704 processed PDB files shows that 1,039 proteins (22.1%) contain exclusively detergent mimetics (e.g., dodecyl maltoside, octyl glucoside, lauryldimethylamine oxide) without any physiological lipid species, while an additional 685 proteins (14.6%) contain both detergent and physiological lipids. This means that for roughly one in five proteins, all contact labels derive from crystallization detergents rather

than native lipid species. The protein-level metadata includes an explicit detergent-only flag to enable filtering. These compounds were retained because they frequently occupy membrane-associated binding environments and preserve experimentally observable lipid-protein interaction geometries, even when they do not represent native physiological lipids. The cholesterol derivative CHD (cholic acid, a bile acid sterol derivative) and Y01 (cholesterol hemisuccinate) are distinct compounds with different PDB Chemical Component Dictionary identities ($C_{24}H_{40}O_5$ and $C_{31}H_{50}O_4$, respectively), though both are classified as sterols in MPLID. Users should note that cholesterol hemisuccinate is an artificial detergent commonly added during membrane protein solubilization and is not a native membrane lipid.

Lipid code verification.

During revision, all 90 PDB-derived candidate ligand codes were audited against the RCSB Chemical Component Dictionary API (Supplementary Table S1). This audit confirmed 45 codes as correctly classified, identified 34 codes with incorrect or ambiguous category assignments, and found 11 candidate codes absent from the dictionary. Of the 34 misclassified codes, 15 appear in PDB HETATM records within the dataset, but most have low occurrence. The codes with the largest impact are CHD (cholic acid, present in 150 structures; name corrected from "cholesteryl hemisuccinate"), CRT (spirilloxanthin, a bacterial carotenoid incorrectly classified as ceramide, present in 22 structures), and ARA (alpha-L-arabinopyranose, a sugar incorrectly classified as arachidonic acid, present in 18 structures). These misclassified contacts collectively represent fewer than 300 protein entries and do not materially alter the dataset statistics. The three codes specifically flagged by reviewers (LNS, LNN, GLF) are confirmed as misclassified but do not appear in any PDB structure represented in MPLID and therefore do not affect residue-level contact annotations. The complete verification results are provided in Supplementary Table S1 for transparency.

NMR multi-model structures.

The current pipeline counts residue-lipid contacts across all conformational models in NMR-derived PDB coordinate files rather than extracting contacts from a single representative model. Empirical detection of duplicated residue positions identified 46 multi-model structures in the dataset, contributing approximately 144,000 excess residue entries (1.8% of total residues) and approximately 29,000 excess contact annotations. Although substantial in relative terms (~36% of all contact annotations), these excess contacts derive from a small subset of multi-model structures and can be readily excluded through metadata filtering. The most extreme case is PDB 6W4F (KRAS4B homodimer on a lipid bilayer nanodisc), which contains 20 NMR models and reports 4,820 contacts across all models, compared to approximately 240 unique contact residues per individual model. A sensitivity analysis excluding all 46 multi-model structures confirms that the principal enrichment patterns (aromatic belt, arginine, glycine, proline depletion) are preserved (Spearman $\rho = 0.80$, $p = 2.3 \times 10^{-5}$), although lysine enrichment is sensitive to NMR inclusion (Supplementary Analysis S2). The multi-model convention also introduces artifactual sub-2.0 Å contact distances from cross-model atom overlaps; these effectively vanish upon NMR exclusion (Supplementary Analysis S2). Users working with NMR structures should be aware of this multi-model counting convention. Future releases will adopt a single-model extraction strategy for NMR structures.

PDB and lipid composition biases.

The dataset inherits biases from the PDB toward well-studied, readily crystallizable membrane protein families, leaving many therapeutically relevant but structurally uncharacterized families underrepresented. As detailed in the Lipid type diversity section, several biologically important lipid classes (PA, PS, phosphoinositides, polyunsaturated fatty acids) are absent, reflecting the difficulty of resolving these lipids crystallographically rather than a deliberate exclusion. Anionic lipids (particularly cardiolipin and phosphatidylglycerol) are overrepresented among structurally resolved lipid contacts relative to their abundance in biological membranes, which has implications for machine learning models trained on MPLID: such models may learn structural detectability biases rather than purely biological binding affinity patterns. Additionally, the removal of 1,019 proteins with zero contacts during quality filtering (see Dataset overview) further limits proteome-wide negative control inference.

Distance cutoff and static snapshots.

The 4.0 Å distance cutoff is a single threshold that may not be optimal for all lipid types; researchers are encouraged to explore alternative cutoffs using the continuous distance values provided in the dataset. As noted in Methods, the cutoff does not distinguish between headgroup and acyl-chain contacts. Because MPLID annotations are derived from static crystallographic and cryo-EM snapshots, they may not fully represent the dynamic nature of protein-lipid interactions in native membrane environments, where lipids undergo lateral diffusion and exchange on nanosecond-to-microsecond timescales. Crystal packing artifacts may also contribute false positive contacts, as lipid molecules resolved in crystal structures can occupy lattice contact interfaces that do not reflect native membrane binding.

Additional technical notes.

No structure resolution cutoff was applied during dataset construction: all PDB structures with recognized lipid molecules were included regardless of crystallographic resolution. While this maximizes dataset size, low-resolution structures (worse than 3.5 Å) may have less reliable lipid positions, potentially introducing noise into contact labels. Researchers working with resolution-sensitive applications are encouraged to filter by PDB resolution using the provided `pdb_id` field. The current release does not include per-residue lipid chemical component codes, meaning that users cannot directly determine which lipid type contacts a given residue; however, the `pdb_id` field allows users to retrieve lipid identities from the original PDB structures. Lipid type stratification at the residue level is planned for a future release. Four selenocysteine (SEC) residues in the dataset are all annotated as lipid contacts (4/4, 100% contact rate); given the extremely small sample size, this observation is not statistically meaningful and these residues were excluded from enrichment ratio analysis.

Cross-validation of MPLID labels against BioDolphin [18] interaction annotations and the curated dataset used by the DREAMM prediction method [22] is planned as future work to assess concordance between independently derived annotation approaches.

Conclusions

In summary, MPLID provides a large-scale, experimentally grounded resource for membrane protein structural biology. By distinguishing direct lipid contact from membrane zone localization, and by positioning itself as complementary to existing resources including OPM, MemBlob, and BioDolphin/PLIP, the dataset enables residue-level prediction tasks with direct relevance to drug discovery, protein engineering, and mechanistic understanding of lipid-mediated regulation. Users should interpret MPLID annotations as representing structurally resolved lipid contacts rather than a complete inventory of biological protein-lipid interactions, and should account for the substantial false negative rate inherent to crystallographic and cryo-EM datasets. The dataset adheres to FAIR principles and is freely available under a CC0 public domain dedication. As the PDB continues to grow and cryo-EM structures increasingly resolve lipid molecules at higher resolution, future releases of MPLID will expand both the coverage and the annotation granularity of this resource, including NMR single-model extraction, per-residue lipid type annotations, and expanded lipid ontology coverage.

Potential implications

MPLID is designed to serve as a large-scale training and evaluation resource for multiple research applications in structural bioinformatics, drug discovery, and membrane biology.

Machine learning for lipid contact prediction

The principal application of MPLID is as a training and evaluation dataset for developing machine learning models that predict lipid contact residues from protein sequence or structure. The pre-defined, cluster-aware train/validation/test splits enable standardized model comparison, and the class imbalance (1.00% observed positive rate, approximately 100:1 negative-to-positive ratio) presents a realistic and challenging binary classification task that requires specialized approaches such as class weighting, oversampling (SMOTE) [37], or focal loss functions. Recent advances in geometric deep learning for protein interaction site prediction [38, 39] and end-to-end protein interactome modeling [40] demonstrate the growing potential of graph-based and attention-based architectures for residue-level prediction tasks. While these methods have been developed primarily for protein-protein interaction sites, their hierarchical geometric representations and multiscale attention mechanisms are architecturally suitable for the protein-lipid contact prediction task addressed by MPLID, particularly given the spatial nature of lipid binding sites. The scale of the dataset is sufficient for training deep neural networks, including architectures that combine structural descriptors with protein language model embeddings from ESM-2 [28] or ProtTrans [25]. We note that the Matthews correlation coefficient should be used as the primary evaluation metric for this task due to the substantial class imbalance, as accuracy and even F1 scores can be misleading when the negative class dominates. Users should also be aware that the substantial false negative rate in the non-contact class (see Limitations) may impose an inherent ceiling on achievable performance metrics.

Drug discovery applications

Lipid binding sites on membrane proteins represent an emerging class of druggable targets. General anesthetics, neurosteroids, and endocannabinoids modulate ion channels and GPCRs through specific lipid binding sites [6, 41], and cholesterol binding sites on GPCRs are increasingly recognized as allosteric modulatory sites [8, 42]. This interpretation is consistent with recent work highlighting exosites and allosteric site-forming residues as therapeutically actionable protein-surface regions [43, 44, 45]. MPLID can inform the identification of lipid-mimetic drug binding sites by providing a catalog of experimentally validated lipid interaction residues across thousands of membrane proteins. Predicted lipid contact sites on therapeutically relevant proteins can guide the design of lipid-conjugated prodrugs, membrane-anchored therapeutics, or allosteric modulators that exploit lipid binding pockets.

Structural biology and protein engineering

Understanding which residues contact lipids is essential for rational membrane protein engineering. Mutations at lipid contact sites can destabilize membrane proteins, alter their trafficking, or modify their functional properties. MPLID provides data to train models that predict which residues are critical for lipid-mediated stability, enabling more informed design of stabilizing mutations for membrane protein crystallization, cryo-EM, or functional studies. Additionally, the dataset supports comparative analysis of lipid contact patterns across protein families, enabling identification of conserved lipid binding motifs and family-specific interaction signatures. These interpretations are based on static structural snapshots. Incorporation of dynamic information may alter residue–lipid proximity patterns, as conformational fluctuations could transiently modify contact definitions.

Integration with protein language models

The residue-level format of MPLID is directly compatible with per-residue embeddings from protein language models. Each residue's 1,280-dimensional ESM-2 embedding [28] or 1,024-dimensional ProtTrans embedding [25] can be concatenated with structural descriptors and used as input to supervised classifiers. The 813 sequence clusters provide sufficient diversity to evaluate whether language model representations generalize across membrane protein families with limited sequence similarity. Alternatively, MPLID can be combined with interpretable structural descriptor frameworks, such as STING RDB-derived nanoenvironment descriptors [20, 21, 43, 44]. In this setting, residue-level lipid contacts may be analyzed in terms of explicit physicochemical and geometric properties, enabling mechanistic interpretation of the nanoenvironment characteristics associated with lipid interaction. This dual compatibility supports both high-capacity representation learning approaches and descriptor-based, interpretable modeling strategies.

Availability of Source Code and Requirements

The source code implementing the complete MPLID construction and analysis pipeline is openly available, with project details as follows:

- Project name: MPLID
- Project home page: `https://github.com/omagebright/MPLID`
- License: MIT license

- Operating system(s): Platform independent (Linux, macOS, Windows)
- Programming language: Python 3.11
- Other requirements: BioPython 1.83, pandas 2.2.2, NumPy 1.26.4, SciPy 1.13, scikit-learn 1.7.2, MMseqs2 v15
- RRID:SCR_028506
- biotools: MPLID
- WorkflowHub: `https://workflowhub.eu/workflows/2176`

The repository includes the complete processing pipeline (structure downloading, lipid identification, contact calculation, quality filtering, and split generation), the analysis scripts that generate the figures and tables in this article together with their underlying numerical data, and documentation of the data schema and methodology.

Conflicts of interest

The authors declare that they have no competing interests.

Funding

This research was funded by the São Paulo Research Foundation (FAPESP) (grant nos. 2023/02691-2, 2025/23708-6). Open access publication charges are expected to be covered through the University of Oxford institutional open access agreement with Oxford University Press, subject to publisher eligibility confirmation. For the purpose of open access, the authors have applied a Creative Commons Attribution (CC BY) license to any Author Accepted Manuscript version arising.

Data availability

The complete MPLID dataset is deposited at Zenodo under a CC0 public domain dedication [46]. Upon acceptance, the dataset will also be deposited in the GigaScience GigaDB repository in accordance with journal policy. The Zenodo archive contains the full residue-level annotations (train, validation, and test splits as gzip-compressed CSV files), protein-level metadata, amino acid composition statistics, the underlying numerical data for Figures 2, 4, 5, and 8, and a data dictionary describing all fields. Snapshots of the data and code are additionally available in the GitHub repository described in the Availability of Source Code and Requirements section. The dataset and code follow the FAIR principles [47], with data released under a CC0 public domain dedication and code under the MIT license.

Author contributions statement

Author contributions follow the CRediT taxonomy. F.B.O.: Conceptualization, Methodology, Software, Formal Analysis, Data Curation, Writing (Original Draft), Visualization. G.N.: Conceptualization (dataset definition and biological framework), Supervision, Writing (Original Draft, Review & Editing), Funding Acquisition. Both authors read and approved the final manuscript.

Ethics approval and consent to participate

Not applicable. This study uses publicly available structural data from the Protein Data Bank and does not involve human subjects, animal experiments, or clinical data.

Acknowledgments

The authors thank Ivan Mazoni and Inácio Henrique Yano for their contributions to data validation and resource management during the early stages of this work. The authors thank Embrapa Digital Agriculture (Embrapa Agricultura Digital) for computational resources and infrastructure support. The authors acknowledge the RCSB Protein Data Bank [26, 27] and the OPM database [12] teams for maintaining the essential structural biology resources upon which this work depends. The authors thank the developers of MMseqs2 [29], BioPython [34], and the broader open-source scientific computing community for the software tools used in this study.

Abbreviations

MPLID: Membrane Protein-Lipid Interaction Database; **PDB**: Protein Data Bank; **OPM**: Orientations of Proteins in Membranes; **GPCR**: G protein-coupled receptor; **MCC**: Matthews correlation coefficient; **CG-MD**: Coarse-grained molecular dynamics; **FAIR**: Findable, Accessible, Interoperable, Reusable; **CDL**: Cardiolipin; **CLR**: Cholesterol; **PLM**: Palmitic acid; **DREAMM**: Detection of Residues on Exposed Areas of Membrane-associated Macromolecules; **ESM-2**: Evolutionary Scale Modeling 2; **SMOTE**: Synthetic Minority Over-sampling Technique; **PLIP**: Protein-Ligand Interaction Profiler; **RCSB**: Research Collaboratory for Structural Bioinformatics; **NMR**: Nuclear magnetic resonance; **CSV**: Comma-separated values.

References

1. Erik Wallin and Gunnar von Heijne. Genome-wide analysis of integral membrane proteins from eubacterial, archaean, and eukaryotic organisms. *Protein Science*, 7(4):1029–1038, 1998. doi: 10.1002/pro.5560070420.
2. John P Overington, Bissan Al-Lazikani, and Andrew L Hopkins. How many drug targets are there? *Nature Reviews Drug Discovery*, 5(12):993–996, 2006. doi: 10.1038/nrd2199.
3. Rita Santos, Oleg Ursu, Anna Gaulton, et al. A comprehensive map of molecular drug targets. *Nature Reviews Drug Discovery*, 16(1):19–34, 2017. doi: 10.1038/nrd.2016.230.
4. Valentina Corradi, Besian I Sejdiu, Haydee Mesa-Galloso, et al. Emerging diversity in lipid–protein interactions. *Chemical Reviews*, 119(9):5775–5848, 2019. doi: 10.1021/acs.chemrev.8b00451.
5. Arthur Laganowsky, Eamonn Reading, Timothy M Allison, et al. Membrane proteins bind lipids selectively to modulate their structure and function. *Nature*, 510(7503):172–175, 2014. doi: 10.1038/nature13419.
6. Anna L Duncan, Wanling Song, and Mark SP Sansom. Lipid-dependent regulation of ion channels and G protein-coupled receptors: insights from structures and simulations. *Annual Review of Pharmacology and Toxicology*, 60:31–50, 2020. doi: 10.1146/annurev-pharmtox-010919-023411.
7. Anthony G Lee. How lipids affect the activities of integral membrane proteins. *Biochimica et Biophysica Acta - Biomembranes*, 1666(1-2):62–87, 2004. doi: 10.1016/j.bbamem.2004.05.012.
8. Michael A Hanson, Vadim Cherezov, Mark T Griffith, et al. A specific cholesterol binding site is established by the 2.8 å

structure of the human β2-adrenergic receptor. *Structure*, 16(6):897–905, 2008. doi: 10.1016/j.str.2008.05.001.

9. Felix-Xavier Contreras, Andreas M Ernst, Felix Wieland, and Britta Brügger. Specificity of intramembrane protein–lipid interactions. *Cold Spring Harbor Perspectives in Biology*, 3(6):a004705, 2011. doi: 10.1101/cshperspect.a004705.
10. Takeshi Harayama and Howard Riezman. Understanding the diversity of membrane lipid composition. *Nature Reviews Molecular Cell Biology*, 19(5):281–296, 2018. doi: 10.1038/nrm.2017.138.
11. Gerrit van Meer, Dennis R Voelker, and Gerald W Feigenson. Membrane lipids: where they are and how they behave. *Nature Reviews Molecular Cell Biology*, 9(2): 112–124, 2008. doi: 10.1038/nrm2330.
12. Mikhail A Lomize, Irina D Pogozheva, Hyeon Joo, Henry I Mosberg, and Andrei L Lomize. OPM database and PPM web server: resources for positioning of proteins in membranes. *Nucleic Acids Research*, 40(D1):D370–D376, 2012. doi: 10.1093/nar/gkr703.
13. Andrei L Lomize, Irina D Pogozheva, Mikhail A Lomize, and Henry I Mosberg. Positioning of proteins in membranes: a computational approach. *Protein Science*, 15(6):1318–1333, 2006. doi: 10.1110/ps.062126106.
14. Alexios Chatzigoulas and Zoe Cournia. DREAMM: a web-based server for drugging protein-membrane interfaces as a novel workflow for targeted drug design. *Bioinformatics*, 38 (24):5449–5451, 2022. doi: 10.1093/bioinformatics/btac680.
15. Thomas D Newport, Mark SP Sansom, and Phillip J Stansfeld. The MemProtMD database: a resource for membrane-embedded protein structures and their lipid interactions. *Nucleic Acids Research*, 47(D1):D390–D397, 2019. doi: 10.1093/nar/gky1047.
16. Phillip J Stansfeld, Joseph E Goose, Martin Caffrey, et al. MemProtMD: automated insertion of membrane protein structures into explicit lipid membranes. *Structure*, 23(7): 1350–1361, 2015. doi: 10.1016/j.str.2015.05.006.
17. Lukasz P Kozłowski and Janusz M Bujnicki. MemBlob database and server for identifying transmembrane regions using cryo-EM maps. *Bioinformatics*, 35(19):3582–3584, 2019. doi: 10.1093/bioinformatics/btz539.
18. Jorge Roel-Touris, Heidi Koldsoe, and Manuel N. Melo. BioDolphin: an open-source computational platform for the analysis of lipid-protein interactions in the PDB. *Communications Chemistry*, 7:Article 308, 2024. doi: 10.1038/s42004-024-01384-z.
19. Melissa F Adasme, Katja L Linnemann, Sarah Naomi Bolz, et al. PLIP 2021: expanding the scope of the protein–ligand interaction profiler to DNA and RNA. *Nucleic Acids Research*, 49(W1):W530–W534, 2021. doi: 10.1093/nar/gkab294.
20. Goran Neshich, Roberto C Togawa, Adauto L Mancini, Paula R Kuser, Michel EB Yamagishi, Georgios Pappas Jr, Wandré V Torres, Thalita Fonseca e Campo, Leandro L Ferreira, Fabio M Luna, et al. STING Millennium: a web-based suite of programs for comprehensive and simultaneous analysis of protein structure and sequence. *Nucleic Acids Research*, 31(13):3386–3392, 2003. doi: 10.1093/nar/gkg578.
21. Goran Neshich, Ivan Mazoni, Sandro Roberto Oliveira, Michel EB Yamagishi, Paula R Kuser-Falcão, Leandro C Borro, Dag H Morita, Katlin Rabelo Souza, Vagner M Gonçalves-Almeida, et al. STING Report: convenient web-based application for graphic and tabular presentations of protein sequence, structure and function descriptors from the STING database. *Nucleic Acids Research*, 34(suppl_2): W209–W213, 2006. doi: 10.1093/nar/gkl226.
22. Alexios Chatzigoulas and Zoe Cournia. Predicting protein–membrane interfaces of peripheral membrane proteins using ensemble machine learning. *Briefings in Bioinformatics*, 23(2):bbab518, 2022. doi: 10.1093/bib/bbab518.
23. Niek van Hilten, Nino Verwei, Jeroen Methorst, Carsten Nase, Andrius Bernatavicius, and Herre Jelger Risselada. PMIpred: a physics-informed web server for quantitative protein–membrane interaction prediction. *Bioinformatics*, 40(2):btae069, 2024. doi: 10.1093/bioinformatics/btae069.
24. Dimitra Paranou, Alexios Chatzigoulas, and Zoe Cournia. Using deep learning and large protein language models to predict protein–membrane interfaces of peripheral membrane proteins. *Bioinformatics Advances*, 4(1): vbae078, 2024. doi: 10.1093/bioadv/vbae078.
25. Ahmed Elnaggar, Michael Heinzinger, Christian Dallago, et al. ProtTrans: toward understanding the language of life through self-supervised learning. *IEEE Transactions on Pattern Analysis and Machine Intelligence*, 44(10):7112–7127, 2022. doi: 10.1109/TPAMI.2021.3095381.
26. Helen M Berman, John Westbrook, Zukang Feng, et al. The Protein Data Bank. *Nucleic Acids Research*, 28(1): 235–242, 2000. doi: 10.1093/nar/28.1.235.
27. Stephen K Burley, Charmi Bhikadiya, Chunxiao Bi, et al. RCSB Protein Data Bank (rcsb.org): delivery of experimentally-determined PDB structures alongside one million computed structure models of proteins from artificial intelligence/machine learning. *Nucleic Acids Research*, 51(D1):D488–D508, 2023. doi: 10.1093/nar/gkac1077.
28. Zeming Lin, Halil Akin, Roshan Rao, et al. Evolutionary-scale prediction of atomic-level protein structure with a language model. *Science*, 379(6637):1123–1130, 2023. doi: 10.1126/science.ade2574.
29. Martin Steinegger and Johannes Söding. MMseqs2 enables sensitive protein sequence searching for the analysis of massive data sets. *Nature Biotechnology*, 35(11):1026–1028, 2017. doi: 10.1038/nbt.3988.
30. Ian Sillitoe, Nicola Bordin, Natalie Dawson, et al. CATH: increased structural coverage of functional space. *Nucleic Acids Research*, 49(D1):D266–D273, 2021. doi: 10.1093/nar/gkaa1079.
31. Gunnar von Heijne. Membrane protein structure prediction: hydrophobicity analysis and the positive-inside rule. *Journal of Molecular Biology*, 225(2):487–494, 1992. doi: 10.1016/0022-2836(92)90934-C.
32. William P Russ and Donald M Engelman. The GxxxG motif: a framework for transmembrane helix-helix association. *Journal of Molecular Biology*, 296(3):911–919, 2000. doi: 10.1006/jmbi.1999.3489.
33. Anna L Duncan, Jonathan J Ruprecht, Edmund RS Kunji, and Alan J Robinson. Cardiolipin binds selectively but transiently to conserved lysine residues in the rotor of metazoan ATP synthases. *Science Advances*, 7(34): eabh2217, 2021. doi: 10.1126/sciadv.abh2217.
34. Peter JA Cock, Tiago Antao, Jeffrey T Chang, et al. Biopython: freely available Python tools for computational molecular biology and bioinformatics. *Bioinformatics*, 25 (11):1422–1423, 2009. doi: 10.1093/bioinformatics/btp163.
35. Wes McKinney. Data structures for statistical computing in Python. In *Proceedings of the 9th Python in Science Conference*, pages 56–61, 2010. doi: 10.25080/

Majora-92bf1922-00a.

36. Fabian Pedregosa, Gaël Varoquaux, Alexandre Gramfort, et al. Scikit-learn: machine learning in Python. *Journal of Machine Learning Research*, 12:2825–2830, 2011.
37. Nitesh V Chawla, Kevin W Bowyer, Lawrence O Hall, and W Philip Kegelmeyer. SMOTE: synthetic minority over-sampling technique. *Journal of Artificial Intelligence Research*, 16:321–357, 2002. doi: 10.1613/jair.953.
38. Yong Han, Shao-Wu Zhang, Qing-Qing Zhang, and Ming-Hui Shi. MGMA-PPIS: Predicting the protein-protein interaction site with multiview graph embedding and multiscale attention fusion. *GigaScience*, 14:giaf114, 2025. doi: 10.1093/gigascience/giaf114.
39. Shizhuo Zhang, Jiyun Han, and Juntao Liu. Protein-protein and protein-nucleic acid binding site prediction via interpretable hierarchical geometric deep learning. *GigaScience*, 13:giae080, 2024. doi: 10.1093/gigascience/giae080.
40. Yu-Hsin Chen, Chien-Fu Liu, Jun-Yi Leu, and Huai-Kuang Tsai. Complete end-to-end learning from protein feature representation to protein interactome inference. *GigaScience*, 14:giaf122, 2025. doi: 10.1093/gigascience/giaf122.
41. Grace Brannigan, Jérôme Hénin, Richard Law, et al. Embedded cholesterol in the nicotinic acetylcholine receptor. *Proceedings of the National Academy of Sciences*, 105(38):14418–14423, 2008. doi: 10.1073/pnas.0803029105.
42. Jacques Fantini and Francisco J Barrantes. How cholesterol interacts with membrane proteins: an exploration of cholesterol-binding sites including CRAC, CARC, and tilted domains. *Frontiers in Physiology*, 4:31, 2013. doi: 10.3389/fphys.2013.00031.
43. Folorunsho Bright Omage, Jorge A. Salim, Ivan Mazoni, Inácio Henrique Yano, Leandro Borro, Juan E. H. Gonzalez, Monica Pickholz, I. A. P. Neshich, I. P. Sousa, K. Santana, A. L. Rochetti, J. Fernandes, J. G. Jardine, G. T. Valente, A. L. Mancini, L. Tasic, R. K. Arni, R. Parise Filho, and Goran Neshich. Protein allosteric site identification using machine learning. *Computational and Structural Biotechnology Journal*, 23:3907–3919, 2024. doi: 10.1016/j.csbj.2024.10.036.
44. Folorunsho Bright Omage, Jorge A. Salim, Ivan Mazoni, Inácio Henrique Yano, Juan E. H. González, I. A. P. Neshich, Leandro C. Borro, Monica Pickholz, J. Fernandes, J. G. Jardine, A. L. Mancini, R. K. Arni, L. Tasic, and Goran Neshich. STINGAllo: a web server for high-throughput prediction of allosteric site-forming residues. *Briefings in Bioinformatics*, 26(4):bbaf424, 2025. doi: 10.1093/bib/bbaf424.
45. Folorunsho Bright Omage, Ivan Mazoni, Inácio Henrique Yano, and Goran Neshich. Protein exosites as drug targets: from structural topology to therapeutic modality. *Drug Discovery Today*, 31(3):104662, 2026. doi: 10.1016/j.drudis.2026.104662.
46. Folorunsho Bright Omage and Goran Neshich. MPLID: Membrane protein-lipid interface dataset, 2026. URL `https://doi.org/10.5281/zenodo.18487584`. Version 2.0.0.
47. Mark D Wilkinson, Michel Dumontier, IJsbrand Jan Aalbersberg, et al. The FAIR guiding principles for scientific data management and stewardship. *Scientific Data*, 3(1):160018, 2016. doi: 10.1038/sdata.2016.18.
