# Supplementary material for: MPLID (Membrane Protein–Lipid Interaction Database): A Large-Scale Experimental Resource of Residue-Level Protein–Lipid Contacts
Source: Gigascience. 2026 Jul 27;15:giag080. doi: 10.1093/gigascience/giag080 (PMC13435803; doi:10.1093/gigascience/giag080)
Supplement: Supplementary file 4 [file giag080_reviewer_1_report_original_submission.pdf]

Reviewer Report

Title: MPLID (Membrane Protein-Lipid Interaction Database): A Large-Scale Experimental Resource of Residue-Level Protein-Lipid Contacts

Version: Original Submission **Date:** 3/19/2026

Reviewer name: Reviewer 1

Reviewer Comments to Author:

The MPLID project aims to identify protein-lipid interactions at a residue-by-residue level. They build a database from the entire PDB and thus examines a wide range of lipids and proteins, producing a huge amount of data (>8 million residues identified). I think this resource is potentially of considerable value for the training of models for downstream analysis.

The study appears well carried out and the paper well written. That said, I have a few issues with the framing of the study, which I think need to be addressed before publication.

Chiefly, it should be made much clearer in the paper that their approach simply looks at protein-lipid interactions as found in the PDB. This is a tiny fraction of all protein-lipid interactions. The PDB is a very specific and somewhat artificial environment - only certain protein-lipid interactions survive the process of purification (often using harsh detergents). Their much-repeated statement that 1% of all residues are lipid contacting is interesting, but this will of course not reflect biological reality. So upfront it should be made clear that this is for "structural" protein-lipid interactions only, which represent only a small amount of the total. Claims that their data "reflects the biological reality that only a small fraction of residues in membrane proteins make direct lipid contacts" should be dropped. The 1% residue claim should be strongly reconsidered or reframed, as their "non-contact" data will have huge amounts of false-negatives, likely exceeding the "contact" data by an order of magnitude. This flavours much of the analysis in the paper. To reiterate, the PDB only contains a very specific subset of protein-lipid interactions.

The authors highlight the novelty of their approach, however in their discussion of other methods they omit to mention MemBlob (https://doi.org/10.1093/bioinformatics/btz539). This work takes cryo-EM maps from the PDB and computationally screen the data to identify the membrane and solvent facing regions. They provide the data online, which includes a residue-level description of whether residues face solvent, lipid, lipid interface, or are buried. This tool/repo somewhat overlaps with MPLID and should be cited and described appropriately.

Similarly, BioDolphin (https://doi.org/10.1038/s42004-024-01384-z) which identifies and labels all protein-lipid interactions from the PDB, as defined with PLIP https://plip-tool.biotec.tu-dresden.de/plip-web/plip/index. It provides full information on interactions at a residue level, and is accessible using an API, so overlaps considerably with the MPLID work. It should be cited and described appropriately.

Due to the focus on structural lipids only, there will be a strong favouring for anionic PIP and CDL lipids (hence the increased abundance of Arg and Lys in the data set). Most lipids in biological membranes are not anionic however - this should be mentioned.

They also see enrichment of certain polar (Ser/His) and aromatic (Trp/Phe/Tyr). This matches previous

analyses on CDL binding (10.1126/sciadv.abh2217), perhaps again reflecting the abundance of anionic lipids in the dataset. This should be discussed.
In terms of the lipid chemical codes used for MPLID in the zenodo link, if these are the codes used for the RSCB PDB then these should be double checked. I looked at a few and got varied results. E.g., LNS appears to be 6-(methylcarbamoyl)pyridine-2-carboxylic acid, which is not a sterol. LNN appears to be (6-hydroxybiphenyl-3-yl)acetic acid, which is not a sterol. GLF is alpha-D-glucopyranosyl fluoride, which is not a sphingolipid. Etc. Perhaps this is my misunderstanding in what these codes represent.
Further, if I understand correctly, they've included detergent interactions in their dataset. I find this a very odd choice, and either suggest these are removed or that this is made much clearer up top.
I'm curious about the protein with 4,820 lipid contacts. 'Lipid contacts' are defined in this study as residues that contact a lipid (within 4 A). This PDB (6W4F) has 810 residues, so far fewer than 4,820. Further, the majority of the protein residues are not in contact with the lipids. Could it be that all 20 states in the PDB file have been accounted for by accident? The number would still be too low, unless it's actually atoms that are being counted rather than whole residues? In which case a median of 4 is incredibly low. In general this is very confusing, and needs to be made more clear.

Methods

Are the methods appropriate to the aims of the study, are they well described, and are necessary controls included? Choose an item.

Conclusions

Are the conclusions adequately supported by the data shown? Choose an item.

Reporting Standards

Does the manuscript adhere to the journal's guidelines on [minimum standards of reporting?]() Choose an item.

Choose an item.

Statistics

Are you able to assess all statistics in the manuscript, including the appropriateness of statistical tests used? Choose an item.

Quality of Written English

Please indicate the quality of language in the manuscript: Choose an item.

Declaration of Competing Interests

Please complete a declaration of competing interests, considering the following questions:

- Have you in the past five years received reimbursements, fees, funding, or salary from an organisation that may in any way gain or lose financially from the publication of this manuscript, either now or in the future?
- Do you hold any stocks or shares in an organisation that may in any way gain or lose financially from the publication of this manuscript, either now or in the future?
- Do you hold or are you currently applying for any patents relating to the content of the manuscript?
- Have you received reimbursements, fees, funding, or salary from an organization that holds or has applied for patents relating to the content of the manuscript?
- Do you have any other financial competing interests?
- Do you have any non-financial competing interests in relation to this paper?

If you can answer no to all of the above, write 'I declare that I have no competing interests' below. If your reply is yes to any, please give details below.

No to all

I agree to the open peer review policy of the journal. I understand that my name will be included on my report to the authors and, if the manuscript is accepted for publication, my named report including any attachments I upload will be posted on the website along with the authors' responses. I agree for my report to be made available under an Open Access Creative Commons CC-BY license (http://creativecommons.org/licenses/by/4.0/). I understand that any comments which I do not wish to be included in my named report can be included as confidential comments to the editors, which will not be published.

I agree to the open peer review policy of the journal

To further support our reviewers, we have joined with Publons, where you can gain additional credit to further highlight your hard work (see: https://publons.com/journal/530/gigascience). On publication of this paper, your review will be automatically added to Publons, you can then choose whether or not to claim your Publons credit. I understand this statement.

Yes Choose an item.
