# Supplementary material for: MPLID (Membrane Protein–Lipid Interaction Database): A Large-Scale Experimental Resource of Residue-Level Protein–Lipid Contacts
Source: Gigascience. 2026 Jul 27;15:giag080. doi: 10.1093/gigascience/giag080 (PMC13435803; doi:10.1093/gigascience/giag080)
Supplement: Supplementary file 6 [file giag080_reviewer_2_report_original_submission.pdf]

Reviewer Report

Title: MPLID (Membrane Protein-Lipid Interaction Database): A Large-Scale Experimental Resource of Residue-Level Protein-Lipid Contacts

Version: Original Submission **Date:** 4/29/2026

Reviewer name: Reviewer 2

Reviewer Comments to Author:

This manuscript by Omage and Neshich presents a new tool, MPLID, a large-scale, experimentally derived dataset of residue-level protein-lipid contacts extracted from PDB structures.
The manuscript addresses a conceptual gap between membrane zone annotations (e.g., OPM) and direct lipid-contact residues which is biologically important. The dataset is carefully curated and well documented. This makes the work potentially valuable for machine learning, membrane protein biology, and drug discovery. The manuscript is generally well motivated and appropriate as a Data Note, but there are important methodological and interpretational limitations that should be more clearly acknowledged and, where possible, addressed.
- Lipid contacts resolved in PDB structures are not a random sample of biologically relevant lipid interactions. Certain lipids and proteins are overrepresented. While this is inevitable, the manuscript should include the discussion on the consequences of this such as the bias in training ML models (learning structural detectability rather than biological affinity).
- If I understood correct, although the dataset includes detergent mimetics and CHARMM lipid codes, the biological interpretation of these contacts differs substantially from native lipid binding. This might create some problems. For example, detergent contacts may reflect solubilization artifacts, not functional lipid sites. This is a significant limitation for users and should be explicitly flagged.
- How did the authors determine the single distance cutoff to be 4.0 Ã…? Does this distance sufficiently distinguish headgroup vs acyl-chain contacts? Or takes orientation into account?
- Does the removal of zero-contact proteins introduce selection bias? If that is the case, the dataset cannot support true proteome-wide negative controls and the manuscript should discuss that MPLID is not representative of the full membrane proteome.

Methods

Are the methods appropriate to the aims of the study, are they well described, and are necessary controls included? Choose an item.

Conclusions

Are the conclusions adequately supported by the data shown? Choose an item.

Reporting Standards

Does the manuscript adhere to the journal's guidelines on minimum standards of reporting? Choose an item.

Choose an item.

Statistics

Are you able to assess all statistics in the manuscript, including the appropriateness of statistical tests used? Choose an item.

Quality of Written English

Please indicate the quality of language in the manuscript: Choose an item.

Declaration of Competing Interests

Please complete a declaration of competing interests, considering the following questions:

- Have you in the past five years received reimbursements, fees, funding, or salary from an organisation that may in any way gain or lose financially from the publication of this manuscript, either now or in the future?
- Do you hold any stocks or shares in an organisation that may in any way gain or lose financially from the publication of this manuscript, either now or in the future?
- Do you hold or are you currently applying for any patents relating to the content of the manuscript?
- Have you received reimbursements, fees, funding, or salary from an organization that holds or has applied for patents relating to the content of the manuscript?
- Do you have any other financial competing interests?
- Do you have any non-financial competing interests in relation to this paper?

If you can answer no to all of the above, write 'I declare that I have no competing interests' below. If your reply is yes to any, please give details below.

None

I agree to the open peer review policy of the journal. I understand that my name will be included on my report to the authors and, if the manuscript is accepted for publication, my named report including any attachments I upload will be posted on the website along with the authors' responses. I agree for my report to be made available under an Open Access Creative Commons CC-BY license (http://creativecommons.org/licenses/by/4.0/). I understand that any comments which I do not wish to be included in my named report can be included as confidential comments to the editors, which will not be published.

I agree to the open peer review policy of the journal

To further support our reviewers, we have joined with Publons, where you can gain additional credit to further highlight your hard work (see: https://publons.com/journal/530/gigascience). On publication of this paper, your review will be automatically added to Publons, you can then choose whether or not to claim your Publons credit. I understand this statement.

Yes Choose an item.
