# Supplementary material for: MPLID (Membrane Protein–Lipid Interaction Database): A Large-Scale Experimental Resource of Residue-Level Protein–Lipid Contacts
Source: Gigascience. 2026 Jul 27;15:giag080. doi: 10.1093/gigascience/giag080 (PMC13435803; doi:10.1093/gigascience/giag080)
Supplement: Supplementary file 8 [file giag080_reviewer_3_report_original_submission.pdf]

Reviewer Report

Title: MPLID (Membrane Protein-Lipid Interaction Database): A Large-Scale Experimental Resource of Residue-Level Protein-Lipid Contacts

Version: Original Submission **Date:** 4/30/2026

Reviewer name: Reviewer 3

Reviewer Comments to Author:

Lipids are known to interact with membrane proteins and regulate their structure and function; however, because the number of documented interaction examples is limited, the detailed mechanisms of these interactions remain insufficiently understood. The authors provide a comprehensive database of membrane protein-lipid interactions derived from PDB data. This effort is of extremely high importance for research in this field.
On the other hand, as noted in the Limitations section, there are several issues: the analysis depends on the quality of the data deposited in the PDB; the amount of data is limited because lipids may dissociate during purification; and detergents are also counted as lipids. In particular, the assignment of lipids appears to be rather ambiguous. For example, lipid species such as PA and PS are not included, and phosphoinositides (PIPs), which are known to bind to various membrane proteins, are also absent. In addition, polyunsaturated fatty acids have not been detected. Cholesterol hemisuccinate and cholesteryl hemisuccinate appear to be the same compound. Although these are cholesterol derivatives, they are in fact a type of detergent added artificially. If possible, it would be desirable to revise these points or conduct additional analyses.
In any case, this manuscript contains important findings and is considered worthy of publication in GigaScience.

Methods

Are the methods appropriate to the aims of the study, are they well described, and are necessary controls included? Choose an item.

Conclusions

Are the conclusions adequately supported by the data shown? Choose an item.

Reporting Standards

Does the manuscript adhere to the journal's guidelines on [minimum standards of reporting?] Choose an item.

Choose an item.

Statistics

Are you able to assess all statistics in the manuscript, including the appropriateness of statistical tests used? Choose an item.

Quality of Written English

Please indicate the quality of language in the manuscript: Choose an item.

Declaration of Competing Interests

Please complete a declaration of competing interests, considering the following questions:

- Have you in the past five years received reimbursements, fees, funding, or salary from an organisation that may in any way gain or lose financially from the publication of this manuscript, either now or in the future?
- Do you hold any stocks or shares in an organisation that may in any way gain or lose financially from the publication of this manuscript, either now or in the future?
- Do you hold or are you currently applying for any patents relating to the content of the manuscript?
- Have you received reimbursements, fees, funding, or salary from an organization that holds or has applied for patents relating to the content of the manuscript?
- Do you have any other financial competing interests?
- Do you have any non-financial competing interests in relation to this paper?

If you can answer no to all of the above, write 'I declare that I have no competing interests' below. If your reply is yes to any, please give details below.

I declare that I have no competing interests

I agree to the open peer review policy of the journal. I understand that my name will be included on my report to the authors and, if the manuscript is accepted for publication, my named report including any attachments I upload will be posted on the website along with the authors' responses. I agree for my report to be made available under an Open Access Creative Commons CC-BY license (http://creativecommons.org/licenses/by/4.0/). I understand that any comments which I do not wish to be included in my named report can be included as confidential comments to the editors, which will not be published.

I agree to the open peer review policy of the journal

To further support our reviewers, we have joined with Publons, where you can gain additional credit to further highlight your hard work (see: https://publons.com/journal/530/gigascience). On publication of this paper, your review will be automatically added to Publons, you can then choose whether or not to claim your Publons credit. I understand this statement.

Yes Choose an item.
