# Supplementary material for: MPLID (Membrane Protein–Lipid Interaction Database): A Large-Scale Experimental Resource of Residue-Level Protein–Lipid Contacts
Source: Gigascience. 2026 Jul 27;15:giag080. doi: 10.1093/gigascience/giag080 (PMC13435803; doi:10.1093/gigascience/giag080)
Supplement: Supplementary file 9 [file giag080_supplemental_file.pdf]

Supplementary Table S1: MPLID Lipid Code Verification Against RCSB PDB Chemical Component Dictionary

Folorunsho Bright Omage and Goran Neshich

All 90 non-CHARMM lipid codes in MPLID were cross-validated against the RCSB PDB Chemical Component Dictionary API (`https://data.rcsb.org/rest/v1/core/chemcomp/`). The 27 CHARMM simulation nomenclature codes (POPC, POPE, etc.) are excluded as they are not PDB Chemical Component Dictionary entries.

Status key: Verified = RCSB identity matches MPLID category; Misclassified = RCSB identity does not match; Not found = code absent from RCSB dictionary.

Table 1: Cross-validation of MPLID lipid codes against RCSB PDB Chemical Component Dictionary.

Code	MPLID Category	RCSB Name	Status	PDBs	Notes
BOG	Detergent	Octyl beta-D-glucopyranoside	Verified	510	Common detergent
CDL	Cardiolipin	CARDIOLIPIN	Verified	675	Confirmed
CLR	Sterol	CHOLESTEROL	Verified	1119	Confirmed
DGA	Glycerolipid	DIACYL GLYCEROL	Verified	–	Confirmed
DPG	Phospholipid	Archaeal diether PG	Verified	–	Confirmed
EPA	Fatty acid	Eicosapentaenoic acid	Verified	–	Confirmed
ERG	Sterol	ERGOSTEROL	Verified	–	Confirmed
HTG	Detergent	Heptyl 1-thio-glucoside	Verified	–	Confirmed
HXJ	Sphingolipid	Sphingomyelin variant	Verified	1	Confirmed
KDO	Lipid A	3-Deoxy-manno-octulosonate	Verified	–	Confirmed
LDA	Detergent	Lauryldimethylamine oxide	Verified	263	Confirmed
LHG	Phospholipid	Dipalmitoyl-PG	Verified	–	Confirmed
LMT	Detergent	Dodecyl maltoside	Verified	677	Confirmed
LPS	Phospholipid	Lysophosphatidylserine	Verified	–	Confirmed
MYR	Fatty acid	MYRISTIC ACID	Verified	426	Confirmed
OLA	Fatty acid	OLEIC ACID	Verified	379	Confirmed
OLC	Detergent	Monoolein	Verified	548	Confirmed
PAM	Fatty acid	PALMITOLEIC ACID	Verified	–	Confirmed
PC1	Phospholipid	Diacyl-phosphocholine	Verified	–	Confirmed
PCW	Phospholipid	DOPC	Verified	196	Confirmed
PEE	Phospholipid	DOPE	Verified	273	Confirmed
PGV	Phospholipid	Phosphatidylglycerol	Verified	144	Confirmed
PLC	Phospholipid	Diundecyl-PC	Verified	–	Confirmed
PLM	Fatty acid	PALMITIC ACID	Verified	734	Confirmed
POV	Phospholipid	POPC	Verified	344	Confirmed
PTY	Phospholipid	Phosphatidylethanolamine	Verified	–	Confirmed
S1P	Sphingolipid	Sphingosine-1-phosphate	Verified	16	Confirmed
SDS	Detergent	DODECYL SULFATE	Verified	–	Confirmed
SPH	Sphingolipid	SPHINGOSINE	Verified	102	Confirmed
STE	Fatty acid	STEARIC ACID	Verified	94	Confirmed
TGL	Glycerolipid	TRISTEAROYLGLYCEROL	Verified	–	Confirmed
Y01	Sterol	Cholesterol hemisuccinate	Verified	483	Artificial derivative
C10	Detergent	HexaEG monodecyl ether	Verified	–	Confirmed
C8E	Detergent	TetraEG octyl ether	Verified	63	Confirmed

Code	MPLID Category	RCSB Name	Status	PDBs	Notes
DCR	Fatty acid	Icosanoic acid (C20:0)	Verified	1	C20:0 not C10:0
BGC	Detergent	Beta-D-glucopyranose	Verified	–	Sugar/detergent
CHD	Sterol	CHOLIC ACID	Misclassified	150	Bile acid, not CHS
CRT	Sphingolipid	SPIRILLOXANTHIN	Misclassified	22	Carotenoid pigment
ARA	Fatty acid	Alpha-L-arabinopyranose	Misclassified	18	Sugar, not arachidonate
DHA	Fatty acid	2-Amino-acrylic acid	Misclassified	11	Dehydroalanine
UND	Fatty acid	UNDECANE	Misclassified	20	Hydrocarbon, not acid
STI	Sterol	STI-571 (Imatinib)	Misclassified	3	Tyrosine kinase inhibitor
GSP	Sphingolipid	GDP-monothiophosphate	Misclassified	3	Nucleotide analog
DPC	Detergent	Oseltamivir-like compound	Misclassified	2	Drug molecule
LNL	Fatty acid	Linolenic acid C18:3	Misclassified	3	C18:3 not C18:2
PGR	Phospholipid	1,2-Propanediol	Misclassified	2	Small diol
C9V	Cardiolipin	Boron-CDL complex	Misclassified	1	Complex product
LNS	Sterol	Pyridine carboxylic acid	Misclassified	0	Not lanosterol
LNN	Fatty acid	Biphenyl acetic acid	Misclassified	0	Not linolenic acid
GLF	Sphingolipid	Glucopyranosyl fluoride	Misclassified	0	Not galactosylceramide
SIT	Sterol	Fusicoccin derivative	Misclassified	0	Not beta-sitosterol
GCR	Sphingolipid	GALLICHROME	Misclassified	0	Gallium siderophore
DPS	Phospholipid	Indolyl-benzenesulfonyl	Misclassified	0	Drug fragment
IPC	Phospholipid	Thiophene carboxylic acid	Misclassified	0	Not inositol-PC
MPE	Phospholipid	Various	Misclassified	0	Not PE derivative
OHC	Sterol	Various	Misclassified	0	Not hydroxycholesterol
P34	Phospholipid	Various	Misclassified	0	Not PIP3
PI3	Phospholipid	Various	Misclassified	0	Not PI(3)P
PI4	Phospholipid	Various	Misclassified	0	Not PI(4)P
PIP	Phospholipid	Various	Misclassified	0	Not PIP
PTI	Phospholipid	Various	Misclassified	0	Not PI
18W	Cardiolipin	Pyrrolone derivative	Misclassified	0	Not CDL variant
1PL	Phospholipid	Cyclopentapentalene deriv.	Misclassified	0	Not phospholipid
6LP	Lipid A	Pyrimidine derivative	Misclassified	0	Not lipid A
DAG	Glycerolipid	Aminodideoxy-glucopyranose	Misclassified	0	Not diacylglycerol
MAG	Glycerolipid	Various	Misclassified	0	Not monoacylglycerol
TAG	Glycerolipid	Various	Misclassified	0	Not triacylglycerol
FC6	Detergent	Hexacyanoferrate	Misclassified	0	Inorganic complex
FC8	Detergent	Purine diamine	Misclassified	0	Drug molecule
LPA	Lipid A	Various	Misclassified	0	Not lysophosphatidic acid
LIP	Phospholipid	Various	Misclassified	0	Generic lipid
2PL	Phospholipid	–	Not found	–	CHARMM-style?
DM	Detergent	–	Not found	–	CHARMM abbrev.
FC10	Detergent	–	Not found	–	4-char code
FC12	Detergent	–	Not found	–	4-char code
FC14	Detergent	–	Not found	–	4-char code
FC16	Detergent	–	Not found	–	4-char code
LAU	Fatty acid	–	Not found	–	Lauric acid
NG	Detergent	–	Not found	–	2-char, ambiguous
NM	Detergent	–	Not found	–	2-char, ambiguous
OCL	Sterol	–	Not found	–	Cholesteryl oleate
OG	Detergent	–	Not found	–	2-char, ambiguous

Summary: Of 90 non-CHARMM codes, 45 (50.0%) are correctly classified, 34 (37.8%) are misclassified (RCSB identity does not match MPLID category), and 11 (12.2%) are absent from the RCSB dictionary. Of the 34 misclassified codes, 15 appear in PDB structures within the MPLID dataset. The misclassified codes with the largest dataset impact are CHD (150 PDBs), CRT (22 PDBs), UND (20 PDBs), ARA (18 PDBs), and DHA (11 PDBs).

Codes with zero PDB occurrence (19 misclassified) serve as preventative inclusions that do not affect any dataset annotations.
